# Advancing dementia-friendly hospital care: a systematic review of healthcare professionals’ reported barriers and facilitators using the theoretical domains framework

**DOI:** 10.1093/ageing/afag285

**Published:** 2026-09-22

**Authors:** Michael Shimelash, Jinjing Fu, Annette Plantinga, Sven J G Geelen, Cynthia Hofman, Jurgen A H R Claassen, Barbara C van Munster

**Affiliations:** University Medical Centre Groningen, Department of Internal Medicine, Alzheimer Centre Groningen, Groningen, Netherlands; University of Groningen, Faculty of Medical Sciences, Groningen, GR, Netherlands; University of Groningen, Faculty of Medical Sciences, Groningen, GR, Netherlands; University Medical Centre Groningen, Department of Epidemiology, Groningen, Netherlands; University Medical Centre Groningen, Department of Internal Medicine, Alzheimer Centre Groningen, Groningen, Netherlands; NHL Stenden University of Applied Sciences, Health and Wellbeing research group, Leeuwarden, Netherlands; University of Groningen, Faculty of Medical Sciences, Groningen, GR, Netherlands; University Medical Centre Groningen, Department of Primary and Long-Term Care, Cure and Care in the Community Context (FOUR-C) Research Program, Groningen, GR, Netherlands; Hanze University of Applied Sciences, Research Group Healthy Ageing, Allied Health Care and Nursing, Groningen, Netherlands; Vilans National Centre of Expertise in Long Term Care, Department of Research and Validation, Utrecht, Netherlands; Radboud University Medical Center, Department of Geriatrics, Nijmegen, Netherlands; University of Leicester, Department of Cardiovascular Sciences, Cerebral Haemodynamics in Ageing and Stroke Medicine (CHiASM) Research Group, Leicester, England, United Kingdom of Great Britain and Northern Ireland; University Medical Centre Groningen, Department of Internal Medicine, Alzheimer Centre Groningen, Groningen, Netherlands; University of Groningen, Faculty of Medical Sciences, Groningen, GR, Netherlands; Martini Hospital, Department of Geriatric Medicine, Groningen, Netherlands

**Keywords:** dementia, dementia-friendly hospital care, implementation, person-centred care, systematic review, older people

## Abstract

**Background:**

People living with dementia are common in hospitals and often experience poorer outcomes, yet dementia-friendly hospital care (DFHC) is not consistently embedded in routine practice. A theory-informed synthesis of healthcare professionals’ reported barriers and facilitators is needed to guide implementation.

**Objectives:**

To synthesise healthcare professionals’ reported barriers and facilitators to DFHC using the Theoretical Domains Framework (TDF).

**Methods:**

We conducted a systematic review of peer-reviewed studies published between 2015 and 2025. MEDLINE, CINAHL, PsycINFO and EMBASE were searched, with citation tracking. Eligible studies reported staff-reported barriers and/or facilitators relevant to DFHC in hospital settings. Two reviewers independently screened, extracted and appraised studies using the Mixed Methods Appraisal Tool. Determinants were classified as barriers or facilitators and mapped to TDF domains; recurrent cross-domain bundles were identified inductively. The review was registered in PROSPERO (CRD420251085854).

**Results:**

Sixty-five studies were included. Methodological quality was generally high. We coded 856 determinants: 455 barriers and 401 facilitators. Barriers were most commonly mapped to Environmental Context and Resources (14.1%), Knowledge (12.5%) and Social Influences (11.0%). Facilitators clustered mainly in Social Influences (15.2%), Environmental Context and Resources (15.0%), and Knowledge (14.2%). Recurrent barrier bundles reflected system pressures, capability gaps and social and cultural barriers; recurrent facilitator bundles reflected leadership, experiential education, interprofessional teamwork and workflow conditions that support person-centred care.

**Conclusion:**

DFHC is shaped by interacting structural, social, staff-capability and workflow conditions that do not operate in isolation. Progress will likely require multi-level, multi-component implementation strategies embedded across hospital systems, not stand-alone interventions.

## Key Points

- Barriers and facilitators to dementia-friendly hospital care mapped mainly to environmental, social, knowledge and skill domains.
- Determinants were commonly linked rather than isolated, especially around system pressures, capability gaps and workflow fit.
- Leadership, experiential education, teamwork and practical workflow supports were recurrent facilitators across studies.
- Single-component initiatives are unlikely to be sufficient; multi-level strategies are likely to support dementia-friendly care.
- Hospitals may need to combine training with workflow, communication and organisational supports for dementia-friendly care.

## Introduction

Dementia is a growing global public health challenge and places a significant burden on healthcare systems [1, 2]. In hospital settings, approximately one in four older adults has dementia, yet nearly half remain unrecognised by staff [3–5]. Hospitalisation can be particularly challenging for people with dementia, with higher risks of complications, distress, communication difficulties and poorer outcomes, including prolonged stays and negative care experiences [6–8]. Healthcare professionals, therefore, play a central role in shaping care experiences and outcomes, but delivering dementia-sensitive care in busy hospital environments remains challenging [9–12].

Dementia-friendly hospital care (DFHC) is an approach to hospital care that includes recognising dementia and adapting care processes, environments and staff practices to meet the needs of people living with dementia. It encompasses continuity and person-centred care, dementia-specific knowledge and skills, involvement of relatives, clear communication and non-pharmacological responses to distress [13, 14]. These principles may be embedded in routine care or supported through specific interventions, tools, service models or implementation strategies [14, 15].

Despite growing consensus on DFHC components, these practices are not consistently embedded in routine hospital care [9, 14]. Implementing DFHC requires understanding the determinants (barriers and facilitators) that shape whether healthcare professionals can deliver dementia-friendly practices in daily care. A realist review by Handley et al. suggests that DFHC interventions achieve benefits through hospital staff’s actions and reasoning in routine care, and that adoption depends on staff having the capability, resources and permission to work differently [15]. This highlights the need to synthesise staff-reported barriers and facilitators to inform tailored DFHC implementation strategies. To our knowledge, no comprehensive, theory-informed synthesis of these determinants of DFHC implementation currently exists. Although DFHC implementation is also influenced by broader organisational conditions, focusing on healthcare professionals is important because their practices play a central role in day-to-day care delivery.

We therefore conducted a systematic review using the Theoretical Domains Framework (TDF), a behaviour-change framework that groups determinants of practice into 14 domains to support systematic identification and synthesis of barriers and facilitators [16]. This review aimed to synthesise and categorise healthcare professionals’ reported barriers and facilitators to delivering or implementing DFHC using the TDF.

## Methods

This systematic review followed PRISMA 2020 [17] and was registered in PROSPERO (CRD420251085854).

### Search strategy and selection criteria

With support from an information specialist, we searched MEDLINE (PubMed), CINAHL, PsycINFO and EMBASE using controlled vocabulary and keywords related to DFHC, barriers/facilitators and implementation. The databases were last searched on 4 July 2025. Searches were limited to English-language publications (2015–25); full strategies are provided in Appendix 1 (Tables A1.1–A1.4). Backward and forward citation tracking was performed.

Eligible studies were peer-reviewed empirical studies (qualitative, quantitative or mixed-methods) that examined the delivery or implementation of dementia-relevant hospital care for people with dementia or cognitive impairment across hospital settings (e.g. inpatient wards, emergency departments (EDs), outpatient services, geriatric units, specialist dementia units). Studies were included if they reported at least one implementation determinant (barrier and/or facilitator) relevant to delivering or implementing DFHC and if these findings could be extracted separately when multiple perspectives were reported.

We excluded reviews (systematic/scoping), editorials, commentaries and purely theoretical papers (reference lists were checked for eligible primary studies). We excluded studies conducted solely in non-hospital settings (e.g. nursing homes, hospices, home care, rehabilitation centres, memory clinics). Studies focusing only on delirium were excluded unless dementia-specific findings were clearly extractable. We excluded pharmacological-only studies without relevance to care delivery processes or implementation determinants, and studies reporting only patient or informal caregiver perspectives.

### Operationalisation of DFHC and study focus

In this review, DFHC referred to hospital care that recognises dementia and adapts care processes, environments and staff practices to the needs of people with dementia. Eligible determinants could arise from routine/current care delivery or from implementation of DFHC-relevant interventions or strategies. We classified studies as routine/current care when no distinct initiative was described, and as intervention/tool/strategy-specific when a distinct initiative was described. Determinants from the latter studies were included because they concerned attempts to implement dementia-relevant hospital practices, but findings tied to a particular tool or programme, such as tool usability or documentation design, were treated as context for that initiative rather than as general DFHC barriers. This classification was used to contextualise interpretation, not to conduct separate syntheses by study focus.

### Study selection and screening

Records were exported to EndNote for de-duplication and then imported into ASReview for machine-learning–assisted screening prioritisation [18]. ASReview ranks citations by estimated relevance based on reviewer-labelled examples; reviewers make all inclusion/exclusion decisions.

**Figure 1:**
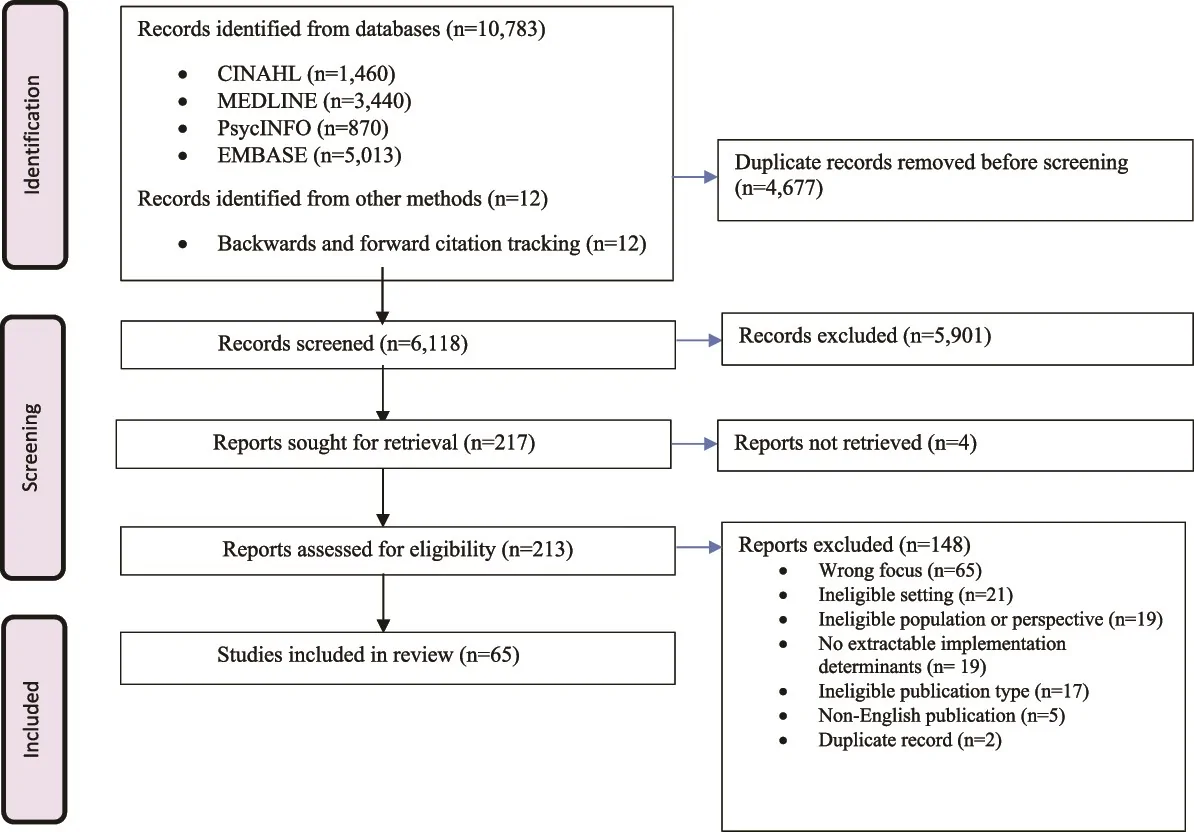
PRISMA 2020 flow diagram of study selection.

Two reviewers (M.S., J.F.) independently screened titles and abstracts in ASReview, starting with the highest-ranked records (i.e. most likely relevant). Screening continued iteratively as the model updated and was stopped after 150 consecutively screened records were judged irrelevant (heuristic stopping rule), consistent with simulation evidence that active-learning screening can reduce workload while maintaining high sensitivity [18, 19]. This stopping rule was applied only at the title/abstract stage; all records labelled potentially eligible proceeded to full-text assessment. Full texts were obtained for potentially eligible records and assessed independently by M.S. and J.F. Discrepancies were resolved through discussion with a third reviewer (A.P.). Study selection is presented in a PRISMA flow diagram (Figure 1). Inter-rater agreement for both stages was assessed using percent agreement and Cohen’s kappa.

### Data extraction

A standardised extraction form was piloted and refined. M.S. extracted study characteristics and text describing staff- or organisation/system-level determinants (barriers and facilitators) relevant to DFHC (Table A1.5). Only determinants reported from the perspective of healthcare professionals (including organisational or system-level factors described in staff data) were extracted for synthesis; patient and caregiver determinants were not coded. J.F. checked each extraction against the full text for completeness and accuracy; disagreements were resolved through discussion.

Where studies reported quantitative results, relevant items and findings were extracted and interpreted as barriers/facilitators based on item framing and direction of results (Table A1.6). Where reported, frequencies/proportions or effect estimates were recorded to preserve context. Open-ended responses were extracted as qualitative text. Where a discrete DFHC intervention or programme was described, we additionally extracted a brief description of intervention content and delivery, guided by the TIDieR checklist items (e.g. what was delivered, by whom and in which setting) [20]. Implementation strategies were recorded when reported and grouped, with reference to Proctor *et al.* [21], into broad educational, organisational and policy-level approaches. Implementation outcomes, including acceptability, feasibility, fidelity, adoption and sustainability, were recorded separately. Extracted items are provided in Appendix 2.

### Quality appraisal

Two reviewers (M.S., J.F.) appraised each included study using the Mixed Methods Appraisal Tool (MMAT) [22]. Appraisal was used to describe methodological strengths and limitations and to contextualise the interpretation of the synthesised determinants.

### Data synthesis

We conducted a TDF-guided synthesis of hospital staff-reported implementation determinants (barriers and facilitators) for delivering DFHC. M.S. identified determinants from the extracted text, classified each as a barrier or facilitator based on the reported direction of influence on DFHC delivery (decision rules: Table A1.6), and linked determinants to professional groups where reported. J.F. verified determinant identification and professional group assignment against the source texts; discrepancies were resolved through discussion and reference to the original reports (Appendixes 3 and 4).

Determinants were then deductively mapped to one or more of the 14 TDF domains using domain definitions and component constructs (Appendix 5). Barriers and facilitators were coded separately, and multi-domain coding was permitted where warranted. M.S. completed TDF coding, and J.F. verified domain assignments against the extracted text and TDF definitions; disagreements were resolved through discussion, with adjudication by a third reviewer (A.P.) if needed. We summarised the frequency of barriers and facilitators within each TDF domain and examined patterns by professional group, describing setting-specific patterns narratively where reported. Frequency counts were used descriptively to show how often determinants were reported and mapped; they were not interpreted as measures of importance, effect size or causal influence. Full coding matrices are provided in Appendixes 3 and 4.

To complement domain-level TDF mapping, we inductively grouped determinants that were reported as linked within individual studies into cross-domain bundles. A bundle was retained if it spanned ≥2 TDF domains, recurred in ≥3 studies and was reported across ≥2 professional groups and/or ≥ 2 hospital settings. M.S. developed candidate bundles and labels, and J.F. verified bundle membership against the extracted text; disagreements were resolved by discussion (A.P. adjudication if needed).

Given heterogeneity in study designs, outcomes and implementation-focused evidence, no meta-analysis or formal certainty assessment was undertaken. Quantitative findings were used to support determinant identification and were synthesised alongside qualitative findings within the TDF mapping. Potential reporting bias was considered narratively from study characteristics and reporting completeness.

## Results

### Study selection and characteristics

The search retrieved 10 795 records, of which 6118 unique records remained after deduplication and were screened in ASReview. 213 reports were assessed for eligibility, and 65 studies were included (Figure 1) [23–87]. Inter-rater agreement was high for both stages (title/abstract: 97.3% agreement, κ = 0.64; full text: 87.8% agreement, κ = 0.70). Study-level characteristics are presented in Table 1 and Appendix 2. Included studies varied in focus: 34 examined routine/current care without a distinct DFHC intervention, while 31 were intervention/tool/strategy-specific.

**Table 1:** Study-level characteristics of included studies, grouped by study design (*n* = 65).

Panel A. Qualitative studies (*n* = 42)					
Study	Country/setting	Staff sample/professional groups	Study focus/intervention or care topic	Data collection	MMAT
Adlbrecht *et al.* [23]	Switzerland; acute geriatric specialised unit	*n* = 27 staff; doctors, nurses, AHPs	**I:** specialised cognitive impairment unit	Interviews, focus groups, documents	5/5
Ashley *et al.* [25]	UK; outpatient oncology services	*n* = 19 staff; doctors, nurses, AHPs	**R:** routine cancer care for people with dementia	Interviews, observations, record review	5/5
Burn *et al.* [28]	UK; acute hospital admission pathways	*n* = 23 staff; doctors, nurses, managers	**I:** dementia case-finding policy/process	Interviews	5/5
Cheong *et al.* [29]	Singapore; specialised acute dementia unit	*n* = 10 nurses	**I:** CAMIE person-centred dementia unit	Interviews	5/5
Cross *et al.* [31]	UK; orthogeriatric wards and EDs	Staff interviews and 204 h observation	**R:** orthopaedic hip-fracture care with cognitive impairment	Ethnographic observation, interviews	5/5
Dadich *et al.* [32]	Australia; geriatric evaluation/management unit	*n* = 50 participants; clinical and non-clinical staff	**I:** unit-wide person-centred dementia safety model	Video-reflexive ethnography, interviews, observations	5/5
Deeks *et al.* [33]	Australia; hospital discharge/transitions	*n* = 51 participants; hospital and community professionals	**R:** medication and transitions of care	Interviews	5/5
Dowding *et al.* [34]	UK; acute hospital wards	*n* = 52 staff; doctors, nurses, AHPs	**R:** pain recognition, assessment and management	Ethnography, interviews, audits	5/5
Dunkle *et al.* [35]	USA; ED and inpatient units	*n* = 17 staff; nurses, social workers	**R:** BPSD care in acute settings	Interviews	5/5
Featherstone *et al.* [36]	UK; medical assessment and orthopaedic wards	684 h observation; staff interviews/groups	**I:** visual signs/symbols and dementia identifiers	Ethnography, interviews, documents	5/5
Fukuda *et al.* [38]	Japan; acute medical/surgical wards	*n* = 50 nurses	**R:** acute nursing care for patients with dementia	Focus groups	5/5
Griffiths *et al.* [42]	UK; oncology/radiotherapy outpatient departments	*n* = 19 staff; doctors, nurses, AHPs	**R:** cancer treatment and service adaptation	Ethnography, interviews, record review	5/5
Handley *et al.* [43]	UK; general hospital wards	*n* = 36 staff; doctors, nurses, AHPs	**I:** dedicated dementia ward/mobile one-to-one team	Interviews, observation, record review	5/5
Harkin *et al.* [44]	UK; ED, acute medical and surgical wards	Focus groups *n* = 14; pathway observations/interviews	**I:** participatory pain-management tools/prompts	Audit, observation, focus groups, interviews	5/5
Heward *et al.* [45]	England; acute hospital training settings	*n* = 17 trainers	**I:** DEALTS2 simulation-based train-the-trainer programme	Interviews	5/5
Higgins *et al.* [46]	UK; acute medical, elderly medicine and orthopaedic wards	*n* = 37 staff/leaders; 132 h observation	**R:** person-centred oral hydration care	Observation, interviews, document review	5/5
Ingelson *et al.* [48]	USA; medicine, surgery and telemetry units	*n* = 12 nurses	**R:** pain-management knowledge and beliefs	Interviews	5/5
Jelinski *et al.* [50]	Canada; emergency departments	*n* = 11 staff; doctors, nurses, AHPs	**R:** dementia care in ED	Interviews	5/5
Jensen *et al.* [51]	Denmark; orthopaedic hip-fracture unit	*n* = 8 staff; nurses, managers	**R:** acute orthopaedic care	Paired group interviews	5/5
Kable *et al.* [52]	Australia; acute discharge planning	*n* = 33 staff	**R:** transitional care systems	Focus groups	5/5
Kjelle *et al.* [54]	Norway; imaging departments	*n* = 8 radiographers	**R:** radiography/imaging care	Interviews	5/5
Kovaleva *et al.* [55]	USA; perioperative units	*n* = 20 nurses	**R:** perioperative dementia care	Interviews	5/5
LaMantia *et al.* [57]	USA; hospital outpatient/geriatrics clinics	*n* = 22 staff; doctors, nurses, AHPs	**R:** acute-care redesign and hospital-clinic interface	Focus groups	5/5
Leggett *et al.* [58]	USA; ED, inpatient and outpatient hospital settings	*n* = 49 stakeholders	**R:** existing dementia care across a health system	Interviews	5/5
Lennox *et al.* [59]	Australia; acute geriatrics/ACE unit	*n* = 11 staff; doctors, nurses, AHPs	**R:** needs/preferences in inpatient care	Interviews	5/5
Lichtner *et al.* [60]	UK; mixed acute wards	*n* = 52 staff; doctors, nurses, AHPs	**R:** pain assessment and management	Observation, interviews, audits	5/5
Longley *et al.* [61]	UK; acute stroke and rehabilitation units	*n* = 23 staff; doctors, nurses, AHPs	**R:** stroke rehabilitation decisions	Interviews	5/5
Minaya-Freire *et al.* [63]	Spain; acute geriatric unit	*n* = 10 nurses	**R:** pain management in acute geriatric care	Questionnaires, focus groups	5/5
Mwale *et al.* [64]	UK; general medical wards/assessment units	3 hospitals, 6 wards	**R:** cultures of control in acute care	Ethnographic observation, documents	5/5
O’Brien *et al.* [65]	UK; older people’s acute wards	*n* = 26 HCPs; doctors, nurses, AHPs	**R:** negotiating refusal of care	Video-recorded observation	5/5
Osuoha *et al.* [66]	USA; medicine, rehabilitation and neuroscience units	*n* = 49 staff; nurses and care technicians	**R:** person-centred acute care	Focus groups	5/5
Petty *et al.* [67]	UK; long-stay dementia wards	*n* = 12 staff	**R:** emotional wellbeing in hospital-based dementia care	Interviews	5/5
Plantinga *et al.* [68]	Netherlands; mixed acute hospital wards	*n* = 14 registered nurses	**R:** shared decision-making in daily care	Interviews	5/5
Ren *et al.* [70]	Canada; hospital dementia unit and long-term care homes	*n* = 22 staff; nurses, AHPs, managers	**I:** telepresence robot	Interviews, focus groups, observation	5/5
Scerri *et al.* [72]	Malta; acute medical wards	*n* = 16 nurse managers	**R:** nurse-manager challenges and solutions	Focus groups	5/5
Shadarevian *et al.* [73]	Canada; older adult mental health and acute medical units	*n* = 41 staff	**I:** tablet-based family video toolkit	Interviews, focus groups	5/5
Skingley *et al.* [74]	UK; 10 acute wards across five NHS trusts	Ward implementation teams	**I:** PIE practice-improvement programme	Interviews, observation, documents	5/5
Sutton *et al.* [77]	England; acute hospitals	*n* = 21 healthcare professionals plus carers/patients	**I:** visual identifier systems	Interviews, documents	5/5
Taneichi *et al.* [78]	Japan; acute general hospitals	*n* = 10 certified dementia nurses	**I:** certified dementia nurses as educators/specialists	Focus groups	5/5
Teodorczuk *et al.* [79]	UK; acute non-psychiatric wards	*n* = 25 staff; doctors, nurses, AHPs, managers	**R:** dementia/delirium practice and education needs	Interviews, focus groups	5/5
Turner *et al.* [83]	UK; older people’s wards	*n* = 12 staff	**R:** truth and deception in dementia care	Interviews	5/5
Yous *et al.* [86]	Canada; acute medical units/cardiology	*n* = 15 staff; nurses, AHPs	**R:** responsive behaviours in acute care	Interviews, field notes	5/5
Panel B. Mixed-methods studies (*n* = 16)					
Study	Country/setting	Staff sample/professional groups	Study focus/intervention or care topic	Data collection	MMAT
Backhouse *et al.* [26]	UK; acute hip-fracture wards	*n* = 63 staff; doctors, nurses, AHPs, managers	**I:** PERFECT-ER checklist intervention	Interviews, focus groups, documents	3/5
Coker *et al.* [30]	Canada; post-acute specialised dementia unit	*n* = 26 staff/core team; survey samples	**I:** Meaningful Moments initiative	Focus groups, surveys, audits	4/5
Flessa *et al.* [37]	USA; ACE unit	*n* = 27 stakeholders	**R:** co-design of life-story intervention	Interviews, surveys	5/5
Gkioka *et al.* [39]	Greece; mixed acute hospital wards	*n* = 242 trained staff	**I:** person-centred dementia training	Surveys, open-ended responses	3/5
Godfrey *et al.* [40]	England; 10 acute wards in five NHS trusts	*n* = 10 wards; staff interviews/surveys	**I:** PIE programme	Interviews, observation, surveys, documents	4/5
Goodwin *et al.* [41]	UK; emergency departments	*n* = 52 survey; *n* = 13 interviews	**R:** ED responses to behaviour that challenges	Survey, interviews	4/5
Hung *et al.* [47]	Canada; medicine and mental health units	*n* = 310 trained; *n* = 297 survey; *n* = 24 focus groups	**I:** Gentle Persuasive Approaches workshop	Surveys, focus groups	4/5
Innes *et al.* [49]	Malta; two hospital wards	*n* = 69 staff; *n* = 16 patients observed	**I:** feedback package, lecture/mentoring and environmental cues	Observation, questionnaires	4/5
Kurrle *et al.* [56]	Australia; multiple acute wards across six hospitals	Staff surveys, audits and clinical lead interviews	**I:** CHOPs programme	Audits, surveys, interviews	2/5
Redley *et al.* [69]	Australia; general medicine wards	*n* = 25 nurses; nurse/manager survey samples	**I:** BRAIN-TRK app	Interviews, focus groups, surveys	4/5
Sampson *et al.* [71]	UK; older adult medical wards	*n* = 20 patients observed; staff included	**R:** agitation near end of life	Observation, interviews	4/5
Surr *et al.* [75]	UK; mixed health and social care services including hospitals	Audit and survey samples; interviews/focus groups	**I:** dementia education/training programmes	Audits, surveys, interviews, focus groups	4/5
Surr *et al.* [76]	UK; three NHS acute/general hospital trusts	*n* = 49 staff; ward observations/surveys	**I:** impactful dementia training programmes	Interviews, focus groups, observations, surveys	4/5
Toubøl *et al.* [80]	Denmark; general hospital departments	*n* = 849 baseline staff survey; follow-up surveys	**I:** whole-workforce dementia education	Questionnaires, interviews	4/5
Walzer *et al.* [84]	Germany; neurosurgery, surgery and radiology wards	Nurse surveys/focus groups	**I:** bed-exit information system	Questionnaires, focus groups, documents	4/5
Wilkinson *et al.* [85]	UK; district general hospital	*n* = 6 junior doctor champions; *n* = 26 doctors	**I:** junior doctor dementia champions	Focus groups, questionnaires	4/5
Panel C. Quantitative studies (*n* = 7)					
Study	Country/setting	Staff sample/professional groups	Study focus/intervention or care topic	Data collection	MMAT
Bashian *et al.* [27]	USA; acute medical telemetry unit	*n* = 42 nursing staff; nurse champions, managers, psychologist	**I:** STAR-VA behavioural programme	Questionnaire, programme monitoring data	3/5
Keuning-Plantinga *et al.* [53]	Netherlands; mixed acute hospital wards	*n* = 229 nurses	**R:** nurses’ perceptions of acute dementia care	Online questionnaire	2/5
Tropea *et al.* [82]	Australia; ED and general medical wards	*n* = 112 staff; doctors, nurses, AHPs	**R:** barriers/facilitators to best-practice dementia care	Survey	2/5
Zygouris *et al.* [87]	Greece; public general hospitals	*n* = 161 nurses	**I:** computerised dementia screening	Questionnaire	3/5
Akrour *et al.* [24]	Switzerland; acute geriatric care unit	*n* = 34 nursing staff	**I:** non-pharmacological BPSD/CMAI programme	Clinical audits, observation, EHR review	2/5
McCorkell *et al.* [62]	Northern Ireland; acute trauma/orthopaedics unit	*n* = 20 patient audit	**I:** dementia toolkit with purple folder	Audit	3/5
Travers *et al.* [81]	Australia; acute intervention/control wards	*n* = 34 RN CogChamps plus ward data	**I:** CogChamps dementia/delirium practice-change intervention	Audits, observation, records	4/5

**Abbreviations:** ACE, Acute Care for Elders; AHP, allied health professional; BPSD, behavioural and psychological symptoms of dementia; CAMIE, Care for Acute Mentally Infirm Elders; CHOPs, Confused Hospitalised Older Persons program; CMA, Cohen-Mansfield Agitation Inventory; GEM, geriatric evaluation and management; IDT, interdisciplinary team; NHS, National Health Service; NR, not reported; RN, registered nurse; DFHC, dementia-friendly hospital care; ED, emergency department; EHR, electronic health record; HCP, healthcare professional; MMAT, Mixed Methods Appraisal Tool; PIE, Person, Interactions and Environment. BRAIN-TRK, CogChamps, DEALTS2, PERFECT-ER and STAR-VA are names of the corresponding interventions or programmes.

**Study-focus categories:** I = intervention/tool/strategy-specific study; R = routine/current-care. Thirty-four studies were classified as R and 31 as I.

Professional-group categories were not mutually exclusive: nurses were represented in 41 studies, allied health professionals in 25, doctors in 19, and managers in 16. Thirty-five studies reported a named theoretical or methodological framework. For studies that also included patients, carers or non-hospital settings, only healthcare-professional and hospital-relevant findings were included in the synthesis. MMAT descriptive ratings show the proportion of applicable criteria rated ‘Yes’ on a 0–5 scale, rounded to the nearest whole number. Ratings were not used to exclude, weight or rank studies. Criterion-level judgements are provided in Appendix 6.

### Intervention components, implementation strategies and outcomes

Among the 31 intervention/tool/strategy-specific studies, intervention components and implementation outcomes were heterogeneous (Table 2). Commonly described components included education or training, practical tools or documentation supports, champions or specialist roles, environmental or technological adaptations and policy-level or external levers. Reported implementation outcomes were also variable, with most studies describing acceptability, perceived usefulness or feasibility and fewer reporting adoption, fidelity, reach or sustainability.

**Table 2:** Intervention components, implementation strategies and outcomes reported in intervention/tool/strategy-specific studies (*n* = 31).

Category	*n*/31 (%)	What was included	Examples reported	Implementation relevance	Studies reporting category
**Education or training**	24 (77%)	Dementia-specific education, role-specific training, simulation, workshops, train-the-trainer models, bedside or ward-based education	Simulation-based dementia care, Gentle Persuasive Approaches, DEALTS2 train-the-trainer package, junior-doctor dementia champion training, hospital-wide dementia education, dementia care nurse-led bedside education	Mainly addressed Knowledge, Skills, Beliefs about Capabilities and Social Influences by increasing staff confidence, awareness and practical capability	23–24, 26–30, 32, 39–40, 43–45, 47, 56, 62, 69, 74–76, 78, 80–81, 85
**Practical tools or documentation supports**	19 (61%)	Checklists, care bundles, visual identifiers, behaviour plans, biographical or life-story tools, screening templates, electronic flags or point-of-care tools	PERFECT-ER checklists, delirium/BPSD bundles, purple-folder or purple-dot toolkits, Forget-me-not identifiers, pain/cognitive assessment templates, BRAIN-TRK app, computerised dementia screening	Mainly supported Behavioural Regulation, Memory, Attention and Decision Processes and Environmental Context and Resources by prompting, standardising or embedding DFHC tasks into workflow	23–24, 26–28, 30, 36, 40, 43–44, 56, 62, 69, 73–74, 77, 81, 85, 87
**Champions, specialist roles or implementation teams**	17 (55%)	Dementia nurse specialists, dementia care nurses, ward champions, doctor champions, implementation teams, expert support teams, technology champions	RN CogChamps, dementia care nurses, dementia nurse specialists, junior-doctor dementia champions, PIE implementation teams, dementia training leads, staff support for technology-supported care	Mainly addressed Social Influences, Social/Professional Role and Identity and Goals by legitimising DFHC, supporting staff and creating local ownership	23–24, 26–27, 30, 40, 43–45, 70, 73–76, 78, 81, 85
**Environmental or technological adaptations**	9 (29%)	Physical environmental adaptations, low-stimulation spaces, signage, life-story displays, sensor systems, tablet/video tools, telepresence robots	Dementia-friendly signage, low-stimulation lounges, life-story posters, tablet-delivered family videos, telepresence robots, bed-exit sensor systems, visual identifier systems	Mainly addressed Environmental Context and Resources and Behavioural Regulation by making dementia-sensitive care more feasible within hospital routines	24, 32, 36, 56, 69–70, 73, 77, 84
**Policy-level or external levers**	5 (16%)	National/regional dementia-friendly hospital programmes, accreditation initiatives, incentivised case-finding, organisational education policies	Dementia-friendly hospital programmes, CQUIN-linked dementia case-finding, hospital-wide dementia training strategies, accreditation-linked initiatives	Mainly addressed Goals, Environmental Context and Resources and Social Influences by aligning DFHC with organisational priorities and accountability structures	28, 43, 56, 80, 85
**Acceptability, perceived usefulness or feasibility reported**	23 (74%)	Staff views on whether the intervention, tool or program was acceptable, useful or feasible in routine practice	Positive views about training, tools or specialist support, often alongside concerns about workload, competing demands or workflow fit	Showed that implementation was often shaped by perceived fit with routine care, not only by intervention content	23–24, 26–27, 29–30, 32, 36, 40, 43, 45, 56, 62, 69–70, 74, 76, 78, 80–81, 84–85, 87
**Adoption or penetration reported**	15 (48%)	Quantitative indicators of uptake, spread or coverage	Use of screening tools, care bundles, identifiers, training uptake, digital tools, sensor systems or documentation templates	Provided evidence on whether interventions were taken up in practice, although reporting was inconsistent	24, 26–28, 30, 40, 44, 56, 62, 69, 74, 80–81, 84–85
**Fidelity, dose or reach reported**	10 (32%)	Audit data, usage data or documentation showing whether intervention components were delivered as intended	Audits of screening, care-bundle completion, documentation, training coverage, usage of digital tools or sensor systems	Often showed partial implementation, inconsistent documentation or variable delivery across settings	24, 26–27, 30, 40, 56, 69, 74, 81, 84
**Sustainability beyond initial implementation reported**	4 (13%)	Evidence of continuation, embedding, spread or maintenance after the initial project period	Longer-term continuation of training, practice-improvement models, dementia-friendly initiatives or champion roles	Sustainability was rarely evaluated, limiting conclusions about longer-term embedding of intervention/tool-specific approaches	40, 56, 76, 85

**Abbreviations:** BPSD, behavioural and psychological symptoms of dementia; DFHC, dementia-friendly hospital care; TDF, Theoretical Domains Framework.

Categories were not mutually exclusive; studies could contribute to more than one category when multiple implementation strategies or implementation outcomes were reported. Percentages use 31 intervention/tool/strategy-specific studies as the denominator. Intervention/tool/strategy-specific studies were those describing a distinct DFHC intervention, tool, training package, policy, service model or implementation strategy. Study numbers correspond to the included-study reference list. Study-level intervention details are provided in Appendix 2.

### Methodological quality

MMAT findings differed by study design. Qualitative studies generally met the applicable criteria, with a mean of 100% of applicable criteria rated ‘Yes’. Mixed-methods studies had a mean of 78%, with limitations mainly related to integration of qualitative and quantitative components. Quantitative non-randomised and descriptive studies had lower mean proportions of criteria rated ‘Yes’ (60% and 50%, respectively), mainly due to limited reporting of confounding, representativeness and nonresponse bias. Criterion-level MMAT findings are shown in Figure A1, and study-level assessments are provided in Table 1 and Appendix 6.

### Identified barriers and facilitators

Across the 65 included studies, we descriptively mapped **856** determinants related to delivering or implementing DFHC, comprising **455** barriers (53.2%) and **401** facilitators (46.8%). Most codes were linked to nurses (*n* = 543), followed by allied health professionals (AHPs) (*n* = 176), managers (*n* = 160), and doctors (*n* = 136); codes were linked to more than one professional group when findings referred to multidisciplinary teams (Appendixes 3 and 4).

#### Barrier TDF domains

**Figure 2:**
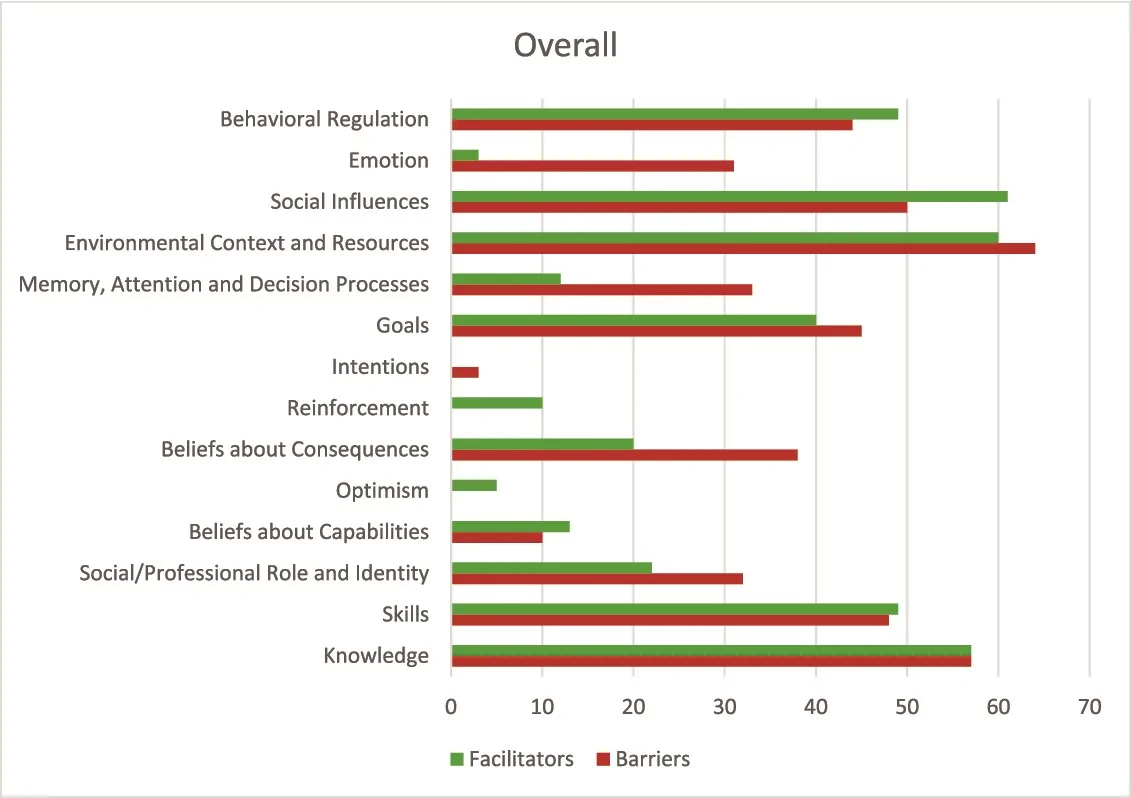
Overall distribution of barriers and facilitators across the TDF domains.

All 455 barriers were mapped to 12 of the 14 TDF domains (Figure 2). 76% clustered in seven domains: Environmental Context and Resources (ECR) (14.1%), Knowledge (12.5%), Social Influences (11.0%), Skills (10.5%), Goals (9.9%), Behavioural Regulation (9.7%) and Beliefs about Consequences (8.4%); none were coded to Optimism or Reinforcement. Examples of barrier content within the most frequently represented domains are summarised in Table 3.

**Table 3:** Summary of commonly reported barriers and facilitators within the most frequently represented TDF domains.

Domain (high frequency)	Common barriers (what staff reported)	Common facilitators (what enabled DFHC)
Environmental context and resources	Workload/time pressure; staffing shortages; competing priorities; dementia-unfriendly ward environment; fragmented information systems	Adequate staffing/skill mix; protected time; supportive environment (orientation cues/quiet spaces); improved information flow
Knowledge	Limited dementia knowledge; uncertainty about best approaches; poor awareness of DFHC principles	Dementia education/training; accessible guidance/resources
Skills	Communication difficulties; limited non-pharmacological de-escalation skills; challenges managing responsive behaviours	Role-relevant skills training; coaching/modelling; practice-based learning
Social influences	Task-driven culture; weak interdisciplinary collaboration; hierarchy; limited leadership engagement	Leadership support/visibility; champions/specialist roles; supportive team culture; MDT working
Behavioural regulation	Lack of routines/pathways; inconsistent documentation/prompts; unclear processes for escalation/management	Care pathways; checklists/prompts; documentation tools; structured routines
Goals	Patient flow priorities overriding DFHC; unclear ownership; misaligned expectations across disciplines	Organisational prioritisation of DFHC; shared goals; clarified responsibility/accountability
Beliefs about consequences	Concerns about safety/risk; fear of adverse events; perceived inefficiency of person-centred care	Confidence in benefits; perceived safety/value of DFHC practices

**Abbreviations:** DFHC = dementia-friendly hospital care.

Domains shown are those most frequently mapped in Figure 2; counts are descriptive mapping frequencies. Items are synthesised examples of commonly reported determinants across included studies.

#### Barriers by professional group

Across professional groups, ECR and Goals were among the most frequently coded domains for nurses, AHPs and managers, whereas Beliefs about Consequences was most frequently coded among doctors. Detailed frequencies by professional group are presented in Table A1.7.

#### Facilitator TDF domains

All 401 facilitators were mapped to 13 of the 14 TDF domains (Figure 2), with none coded to Intentions. Most clustered in five domains: Social Influences (15.2%), ECR (15.0%), Knowledge (14.2%), Skills (12.2%) and Behavioural Regulation (12.2%), followed by Goals (10.0%). Each remaining domain accounted for fewer than 6% of facilitators. Common facilitator content within dominant domains is summarised in Table 3.

#### Facilitators by professional group

Across professions, facilitators clustered in Social Influences, ECR, Knowledge, Skills and Behavioural Regulation, although the emphasis varied by role (Table A1.7). For example, nurses’ facilitators commonly reflected practical supports that make DFHC doable at the bedside (e.g. workflow tools, staffing flexibility), whereas doctors’ facilitators more often related to senior endorsement and prompts such as standardised documentation. AHP facilitators emphasised interdisciplinary working, while managers’ facilitators centred on leadership and resource allocation.

### Cross-domain determinant bundles

We identified recurrent cross-domain bundles of determinants that were reported as linked within studies and that spanned multiple TDF domains (Table A1.8). Three barrier bundles and four facilitator bundles met the retention criteria.

Barrier bundles included system pressures and feasibility constraints (e.g. workload, competing priorities, dementia-unfriendly environments, fragmented information systems); capability gaps (limited dementia-specific knowledge and skills); and social and cultural barriers (task-driven cultures, role ambiguity, hierarchy, emotional burden and stigma).

Facilitator bundles included visible leadership and champions; experiential and role-relevant education; interprofessional teamwork and shared ownership; and workflow fit for person-centred practice, including person-centred information, family involvement, environmental or workflow adaptations and practical tools or technologies that supported routine care.

## Discussion

This review synthesised healthcare professionals’ reported barriers and facilitators to implementing DFHC in hospitals using the TDF. Determinants were most commonly mapped to the domains of Environmental Context and Resources, Knowledge, Skills, Social Influences, Goals and Behavioural Regulation. The recurrent cross-domain bundles extended these domain-level findings by showing that determinants were seldom reported as isolated influences and instead appeared as interrelated patterns embedded within routine hospital practice. These findings suggest that implementing DFHC depends not on a single determinant, but on the interaction of structural, capability-related, social and workflow conditions within routine hospital practice.

### Environmental and social determinants

ECR was the most frequently coded domain, although the difference from other leading domains was modest. This suggests that staff often described dementia-unfriendly care as a system issue rather than solely an attitude or knowledge problem. Across the included studies, staffing levels, workload, ward layout, noise, documentation systems and competing institutional priorities were closely linked to the ability to deliver DFHC practices consistently. This aligns with prior research on dementia care and person-centred practice in hospitals, which similarly identifies staffing levels, workload, the physical environment and organisational priorities as key determinants of care feasibility [14, 15, 88]. Our review adds to this literature by suggesting that these determinants were repeatedly described as shaping whether DFHC can be delivered in routine hospital practice.

Social Influences emerged as another prominent domain. Where leaders, peers and interprofessional teams normalised dementia-sensitive care, staff described these practices as embedded in routine work; where leadership was less supportive and team cultures were task-driven or hierarchical, staff reported reverting to sedation, restraint or task-focused routines. The influence of leadership, team norms and interdisciplinary collaboration on sustaining practice change is well established in implementation literature [89]. Our review extends this literature by indicating that these influences were reported as shaping whether dementia-friendly care was prioritised, supported and sustained in everyday hospital work. Together, these findings indicate that DFHC is influenced by both the structural conditions that determine whether care is feasible and the social context that enables teams to adapt, prioritise and sustain it in routine practice.

### Role-specific manifestations of shared domains

Although similar TDF domains were identified across professional groups, they were often expressed differently according to staff roles and positions within hospital workflows. Nurses most often described barriers related to direct bedside care, such as time pressure, competing tasks, emotional burden and difficulties maintaining person-centred care during routine ward work. By contrast, doctors and managers appeared more often in relation to decision-making authority, prioritisation, leadership and the support, legitimisation and embedding of dementia-friendly practices within service routines. AHPs frequently highlighted issues of coordination, access, communication and the practical fit of interventions within existing care pathways.

These differences should be interpreted cautiously, because the evidence base was dominated by nursing perspectives. This likely reflects both the composition of the literature and the fact that barriers to dementia-friendly care are often most visible in bedside nursing work, where competing demands are encountered most directly. At the same time, previous hospital dementia literature has similarly argued that implementation cannot be understood only at the level of frontline care delivery, because organisational priorities, interprofessional relationships and leadership structures shape whether person-centred practices can be sustained in routine care [15, 90]. Our review findings therefore suggest that DFHC is not simply a nursing issue, but a broader organisational and interprofessional one, even if its practical tensions were most often described through nursing work.

### Setting-specific manifestations of shared domains

The same underlying determinants were identified across settings, but they manifested differently. On inpatient wards, the key challenge was sustaining DFHC within continuous, busy routines, which could limit time for engagement and family conversations. In outpatient and diagnostic services, barriers are more often related to brief encounters and poor coordination, in which dementia-relevant information may not be consistently identified or carried forward. In EDs, pace and frequent transitions could constrain orientation, explanation, and de-escalation and increase the risk of losing dementia-related information during handovers. Notably, outpatient care was comparatively underrepresented in the included studies, despite its role in continuity, recognition of dementia-related needs and coordination across hospital pathways, suggesting that the current literature may under-capture an important part of DFHC implementation [91, 92].

These differences suggest that while the domains were shared, the most realistic ‘entry points’ for change may be setting-specific, for example, ward routines that support continuity, outpatient systems that improve identification and information transfer, and ED processes that protect communication during transitions. This reflects previous work showing that dementia-friendly care depends on its fit with organisational routines and context, while ED-focused literature similarly highlights the importance of dementia-sensitive workflows and safer transitions. It also points to the need for greater attention to outpatient and cross-pathway implementation in future research [15, 91–94].

### Cross-domain bundles in hospital practice

A key finding of this review was that determinants repeatedly appeared as linked cross-domain bundles within hospital care. These bundles were especially evident around system pressures and feasibility constraints, capability gaps, social and cultural barriers affecting teamwork and ownership, and the extent to which person-centred practice fits routine workflows. This suggests that implementation problems in DFHC arise through the interaction of organisational demands, staff knowledge and skills, social processes and everyday care routines, rather than from any single deficit alone.

This interpretation is consistent with previous hospital dementia literature showing that dementia-friendly care depends on how well person-centred practices fit existing organisational routines, care processes and ward contexts, rather than on individual staff knowledge or motivation alone. Earlier reviews have similarly highlighted the importance of feasibility, coordination and organisational support in sustaining dementia-sensitive care in acute settings [14, 15, 88]. Viewed through this lens, when organisational priorities, ward routines and documentation systems emphasise patient flow and risk management, staff may experience dementia-friendly adaptations as extra work that competes with task-oriented routines. Conversely, when knowing the person is structurally supported through leadership, adequate resources, shared accountability and accessible person-centred information, staff more often report greater confidence and more consistent person-centred care.

A further implication is that determinants rarely operate in isolation. Time pressure, for example, was often reported alongside documentation burden, unclear ownership and reduced opportunities for communication or reflection. Likewise, facilitators such as leadership, team routines, family input and accessible person-centred information often appeared as reinforcing conditions rather than separate influences. Seen in this way, the cross-domain bundles help explain why single-component initiatives may have limited impact when wider workflow conditions, team processes and service priorities remain unchanged. They therefore extend the TDF findings by showing not only which determinants were commonly reported, but also how those determinants were configured in practice. This also aligns with recent implementation literature arguing that barriers and facilitators are better understood as interacting conditions than as isolated factors [95]. In this sense, the cross-domain bundles identified in our review add interpretive value by showing how determinants were configured in routine hospital practice, rather than only how they were categorised analytically. This supports the use of multi-level implementation strategies that address linked conditions together rather than targeting single determinants in isolation [15].

### Implications for practice and policy

These findings suggest that DFHC is unlikely to improve through single-component initiatives. Because barriers and facilitators clustered across knowledge and skills, structural conditions and team processes, implementation efforts are likely to require strategies that address these linked determinants together.

In practice, this points to multi-component strategies delivered across different levels of the hospital system. Staff may benefit from role-specific, experiential training that is reinforced in daily practice through coaching, modelling and feedback rather than one-off teaching. At the team and ward level, hospitals should reduce recurring sources of workflow friction by addressing time pressure, environmental stressors, unclear roles and responsibilities and weak communication or handover processes. Leadership visibility and shared ownership may also help sustain dementia-friendly practices in routine care. At the organisational level, DFHC is more likely to be sustained when it is embedded within existing governance, quality improvement and accountability structures, with standards and resourcing aligned to support dementia-sensitive care.

### Strengths and limitations

This review has several strengths. We used a theory-informed approach, applying the TDF to guide the extraction, synthesis and interpretation of findings. We included multiple professional groups across several countries and hospital settings and systematically mapped both barriers and facilitators to TDF domains, with additional descriptions by profession. Despite heterogeneity in study designs and contexts, the findings converged around a core set of determinants across studies, which strengthens the credibility of the synthesis.

However, several limitations should be considered. First, the evidence base was concentrated in high-income countries and acute inpatient settings, with fewer studies from outpatient or diagnostic services and limited representation from low- and middle-income contexts; transferability to other health-system contexts may therefore be limited. Second, perspectives were unevenly represented across professional groups, with nursing data most common and fewer studies capturing determinants from doctors, AHPs and managers. Third, reporting of contextual features and study details was heterogeneous and often incomplete, which limited cross-study comparability. Included studies differed in focus, ranging from routine/current care to specific interventions, tools, training packages and implementation strategies. As a result, some determinants from intervention/tool/strategy-specific studies may reflect features of the specific initiative being implemented, rather than DFHC delivery more broadly. Fourth, because determinants were mapped from healthcare professionals’ reports, patient and caregiver perspectives were not included; therefore, the conclusions reflect the implementation landscape primarily from a staff perspective. In addition, risk of bias due to missing results was not formally assessed, and selective reporting cannot be ruled out. Finally, the cross-domain bundles were developed through an inductive interpretive synthesis rather than a formal configurational approach; therefore, they should be interpreted as recurring patterns across studies, rather than as empirically tested configurations of determinants.

## Conclusion

DFHC is shaped by whether hospital systems make such care feasible, supported and sustainable in routine practice, and by staff capability to deliver it. This review showed that barriers and facilitators clustered around structural pressures, dementia-specific knowledge and skills, leadership and team culture and the fit of person-centred care within everyday workflows. Across studies, these determinants were more often reported as interacting conditions than as isolated influences.

Accordingly, DFHC is unlikely to be advanced through single-component approaches. Progress will require multi-level implementation strategies that combine experiential training with workflow and environmental adaptation, leadership visibility, shared ownership and organisational support for person-centred practice. Advancing DFHC requires treating it as a system-level priority and embedding that priority in the workflows, team processes and organisational conditions of routine hospital care.

## Supplementary Material

Supplementary_material_afag285

## Research Data Transparency and Availability:

No primary datasets were generated for this review. Review materials supporting the findings, including extraction tables and coding matrices, are provided in the supplementary appendices.
