# Supplementary material for: Advancing dementia-friendly hospital care: a systematic review of healthcare professionals’ reported barriers and facilitators using the theoretical domains framework
Source: Age Ageing. 2026 Sep 22;55(9):afag285. doi: 10.1093/ageing/afag285 (PMC13596736; doi:10.1093/ageing/afag285)
Supplement: Supplementary file 1 [file supplementary_material_afag285.zip › aa-26-1308-File003.pdf]

Advancing Dementia-Friendly Hospital Care: A Systematic Review of Healthcare Professionals' Reported Barriers and Facilitators Using the Theoretical Domains Framework

Appendix 2. Extracted study characteristics and implementation details

Supplementary Data

Study characteristics

Title	Author(s)	Year	Country and setting	Sample and professional groups	Study design	Theoretical framework	Data collection	Study aim and key findings
Facilitators and barriers to implementing a specialized care unit for persons with cognitive impairment in an acute geriatric hospital: a process evaluation	Adlbrecht L; Karrer M; Helfenberger N; Ziegler E; Zeller A	2024	Switzerland; Acute geriatric hospital; Specialized 24 bed unit for persons with cognitive impairment within a 90 bed geriatric acute hospital in the German speaking part of Switzerland; other geriatric wards involved via focus groups	n=27 staff (15 individual interviewees from the specialized unit; 12 focus group participants from other geriatric wards); Doctors (3); Nurses (16); AHPs (8); Managers (NR)	Qualitative	i-PARIHS framework; Kuckartz content structuring qualitative content analysis; participatory action research	Semi structured individual interviews with staff from the specialized unit; interprofessional focus groups with staff from other geriatric wards; documentation from implementation workshops including protocols, observations, milestone plans and presentations	Aim was to identify facilitators and barriers to implementing an interprofessional multicomponent specialized unit for persons with cognitive impairment in an acute geriatric hospital. The process evaluation showed that uncontrollable contextual changes such as the COVID 19 pandemic, staff turnover and nursing team fluctuation, a traditional task and efficiency oriented hospital culture, rigid organisational structures, economic billing pressures, staffing shortages, conceptual ambiguity about the specialized unit and triage, and scepticism about benefits created major barriers. Project related communication was unsystematic and roles, processes and facilitation responsibilities were poorly clarified, contributing to fragmented interprofessional cooperation and variable change competence. Facilitators included an established culture of interprofessionalism in daily clinical work, the interprofessionally composed project group and cooperation with researchers, as well as the project groups motivation and change management skills in some members. Overall, incomplete organisational development, limited system responsiveness and fragmented interprofessionalism meant that the multicomponent intervention could only be partially implemented within the project timeframe
Person-Centred Dementia Care in an Acute Hospital: Experiences from Nurses Working in a Specialized Dementia Unit	Cheong CY; Tay FHE; Choo J; Yap PLK	2021	Singapore; Acute tertiary hospital; Specialized 10 bed dementia unit CAMIE within tertiary acute hospital geriatric service, caring for patients with dementia with or without delirium and acute medical problems	n=10 nurses (from 15 CAMIE nurses invited); Doctors (0); Nurses (10); AHPs (0); Managers (0)	Qualitative	None	Individual face to face semi structured interviews with CAMIE nurses, audio recorded and transcribed for inductive thematic analysis	Aim was to explore nurses experiences of implementing person centred dementia care in a specialized acute hospital dementia unit. Nurses initially struggled with limited dementia knowledge, fear and emotional burden when managing aggression within a restraint free ethos, and language barriers with dialect speaking patients. Formal PCC training, experiential simulation, on the job learning, adequate staffing, a designated social nurse, strong teamwork, and supportive leadership enabled nurses to gain confidence, deliver meaningful one to one engagement and restraint free care, and view PCC as both satisfying and high quality, while stigma from other wards and concerns about sustaining passion and staffing remained ongoing challenges
Prevention and management of behavioural and psychological symptoms in patients with dementia in acute care: a best practice implementation project	Rachid Akrour; Catherine Courret-Gilgen; Beatrice Perrenoud	2022	Switzerland; tertiary teaching; acute geriatric; Acute Geriatric Care Unit (AGCU), Lausanne University Hospital; 28 beds; acute geriatric ward; pre/post audits	n=34 staff (nurses and Community Health and Care Assistants); 28 patients; 1 unit (28 beds); Doctors (0); Nurses (34); AHPs (0); Managers (0)	Quantitative non-randomised	None/NR	baseline and follow-up clinical audits; bedside observation; EHR review via grids; attendance sheets; descriptive statistics from JBI PACES	Implement evidence-based recommendations to prevent/manage agitation/aggression (BPSD) in an acute geriatric unit and improve nursing compliance. Post-implementation, organizational processes and staff training improved; nurses more often identified triggers and completed risk assessments. Individualized care planning/documentation lagged due to EHR design, CMAI e-format usability, training timing, communication gaps, and perceived mental workload. Interprofessional collaboration and formalized processes were strengthened; sustainability actions (ongoing training/mentorship) initiated.
Understanding and identifying ways to improve hospital-based cancer care and treatment for people with dementia: an ethnographic study	Laura Ashley; Rachael Kelley; Alys Griffiths; Fiona Cowdell; Ann Henry; Hayley Inman; June Hennell; Margaret Ogden; Maria Walsh; Liz Jones; Ellen Mason; Michelle Collinson; Amanda Farrin; Claire Surr	2021	UK (England); teaching; acute; Outpatient oncology services across two NHS Trusts: large regional cancer centre (e.g., radiotherapy) and a smaller city cancer unit (e.g., chemotherapy clinics: lung; breast; prostate); multiple departments and sites	n=19 staff; Doctors (2); Nurses (8); AHPs (9); Managers (0)	Qualitative	None/NR	Interviews; observations; accompanying conversations; medical record review	To examine staff-experienced challenges and support needs when delivering hospital-based cancer care to people with dementia and to identify ways to address them. Key staff findings: dementia often undocumented or hard to locate in records; oncology teams report limited dementia training; service pressures and targets constrain flexibility for person-centred adjustments; need for longer/consistent appointments, clearer flags, and practical tools; cross-pathway coordination gaps (e.g., who escorts/sits with patients) leave staff "working without the full picture."
Implementing an intervention to enhance care delivery and consistency for people with hip fracture and cognitive impairment in acute hospital wards: a mixed methods process evaluation of a randomised controlled feasibility trial (PERFECTED)	Backhouse T; Fox C; Hammond SP; Poland F; McDermott-Thompson V; Penhale B; Cross JL	2023	UK; acute; Acute NHS hospital wards providing hip fracture care; five wards across England and Scotland; ward-level implementation over seven PDSA cycles	n=63 staff; 5 acute wards; Doctors (6); Nurses (37); AHPs (9); Managers (8)	Mixed methods	CFIR; MRC Process Evaluation guidance; PDSA	Interviews; focus groups; SIL reflective reports; action plans; checklist scores (documentary evidence)	Aim: determine how/under what circumstances PERFECT-ER was implemented and how it impacted staff practices/perceptions. Key findings: implementation occurred on all wards but was vulnerable under staffing/pressure; adaptations (targeting opinion leaders) reduced whole-ward engagement; dedicated SIL time, audit/feedback, champions, and endorsement by senior influencers facilitated uptake; implementation can proceed without cognitive engagement from all staff in hierarchical settings, but broader engagement may improve reach and ethos change.
Implementation of STAR-VA for behavioral symptoms of dementia in acute care: Lessons learned	Hannah M. Bashian; Julia T. Boyle; Seneca Correa; Jane Driver; Caroline Madrigal; Isabel Desroches; Mackenzie Farrell; Olivia Eiten; Katie Flanagan; Talya Shahal; Kelly A. O'Malley	2024	United States; acute; tertiary; government (VA); veterans hospital; 22-bed inpatient medical telemetry unit with two hospice rooms at an urban VA medical center (VA Boston Healthcare System)	n=42 nursing staff on unit (25 survey respondents); 4 Nurse Champions; 1 Behavioral Coordinator (psychologist); 2 nurse managers; 1 pilot acute unit; Doctors (NR); Nurses (42); AHPs (1); Managers (2)	Quantitative descriptive	QUERI implementation roadmap; Pathways to Excellence framework; person–environment fit and social learning theory	Staff questionnaire (Microsoft Forms/paper); attendance/education logs; implementation records (behavior plan counts, timelines); routine program monitoring meetings	To implement and adapt STAR-VA for acute care BPSD management. Staff reported limited skills and high BPSD burden. With leadership support, multiple nurse champions, and an embedded psychologist, STAR-VA education and workflows were delivered across shifts. Rapid identification and tailored behavior plans were feasible, education reached most staff, and processes for sustainment (rounds, check-ins) were established; minor adaptations (multi-NC model, electronic communication, brief in-services) improved fit for the fast-paced acute setting
Dementia case-finding in hospitals: a qualitative study exploring the views of healthcare professionals in English primary care and secondary care	Anne-Marie Burn; Jane Fleming; Carol Brayne; Chris Fox; Frances Bunn	2018	United Kingdom; acute; NHS teaching/ mixed acute; Acute hospitals across six counties (East of England); unplanned admissions ≥75 years; ward and ED admission workflows	n=23 hospital staff from 12 acute hospitals (dementia specialist nurses=12; non-dementia nurses=3; consultants=6; commissioner=1; dementia case-finding coordinator=1); Doctors (6); Nurses (15); AHPs (0); Managers (2)	Qualitative	None/NR (exploratory qualitative; thematic content analysis)	Telephone interviews with hospital staff; (focus groups/interviews with GPs excluded per setting filter)	To understand how acute hospitals implement dementia case-finding and staff views on benefits/challenges. Approaches varied in tools, responsible actors, IT capture, and reporting. Barriers included specialty buy-in, IT inflexibility, and poor cross-setting communication; facilitators included dedicated staff, prompts, and increased awareness. Concerns persisted about appropriateness (delirium vs dementia) and unclear roles for post-discharge pathways
An Action Research Approach to Implementing a Program for Sharing Meaningful Moments With Hospitalized Patients Living With Dementia	Esther Coker; Joanna Sue; Liz Mersereau; Patricia Hewston; Caroline Marr; Sophia Matarazzo; Afeef Vehra; Alexandra Papaioannou	2025	Canada; post-acute; specialized dementia unit; academic health system; 63-bed specialized dementia care unit in a post-acute hospital (Hamilton Health Sciences, St. Peter's Hospital); patients admitted for BPSD assessment/treatment	n=26 staff (focus groups/core team); "Let's Plan" survey n=20; "What Do You Think?" survey n=24 (non-core staff); "Meaningful Moments Outcomes " survey n=38; Doctors (0); Nurses (12); AHPs (13); Managers (1)	Mixed methods	Implementation Research Logic Model; Action Research; Iowa Implementation for Sustainability Framework	Focus groups (observational notes); staff surveys (three waves); audits/feedback; descriptive summaries	To identify and implement hospital-suitable elements of a long-term-care innovation to facilitate meaningful moments. Knowing the person plus simple bedside tools enabled integration into daily care; champions, clear roles, and leadership drove adoption; audit/reminders supported use; EMR documentation of non-pharmacological care remained low; music and brief interactions during care were most used moments

Title	Author(s)	Year	Country and setting	Sample and professional groups	Study design	Theoretical framework	Data collection	Study aim and key findings
Disjunctures in practice: ethnographic observations of orthopaedic ward practices in the care of older adults with hip fracture and presumed cognitive impairment	Jane L. Cross; Tamara Backhouse; Simon P. Hammond; Bridget Penhale; Fiona Scheibl; Nigel Lambert; Anna Varley; Chris Fox; Fiona M. Poland	2022	United Kingdom; acute; teaching; Three NHS teaching hospitals; acute ortho-geriatric wards and associated Emergency Departments (East Anglia, Midlands, Yorkshire); public ward spaces; older adults with hip fracture and presumed cognitive impairment	48 observation sessions; 204 hours; 3 wards; staff interviews (NR count); Doctors (NR); Nurses (NR); AHPs (NR); Managers (NR)	Qualitative	Institutional Ethnography (IE); None/NR for TDF/COM-B/CFIR/NPT/RE-AIM	Ethnographic non-participant observations; ethnographic interviews with consenting staff; field notes; document/context notes	aim: explore how interruptions/disjunctures between staff experience and institutional protocols shape care on acute ortho-geriatric wards for patients with hip fracture and cognitive impairment. key findings: pervasive workflow interruptions categorized as disruptions (unscheduled needs/resistance; toileting; pain; sleep), discontinuities (professional role demarcations; jargon/abbreviation barriers; inconsistent handovers), and dispersions (misplaced notes/meds; hybrid paper–digital mismatches). Organisational targets and standardised sequences objectified patients, constrained autonomy, and reduced capacity for person-centred care; enabling autonomy to manage inevitable interruptions is essential.
Patient safety for people experiencing advanced dementia in hospital: A video reflexive ethnography	Ann Dadich; Jade Rodrigues; Anita De Bellis; Annmarie Hosie; Tamsin Symonds; Justin Prendergast; Alan Bevan; Aileen Collier	2023	Australia; Acute geriatric specialist unit (GEM); Inpatient 11-bed GEM unit within an acute hospital (South Australia); multidisciplinary ward with daily huddles, case conferences, nurse-led code black coordination, and security interface	n=50 participants (patients=3; family=5; manager s=2; clinicians=35; non-clinical staff=5); Doctors (NR); Nurses (NR); AHPs (NR); Managers (2)	Qualitative	Safety-II; Positive Organisational Scholarship; Video-Reflexive Ethnography (VRE)	Observations/fieldnotes; video-recordings of practice; semi-structured interviews; reflexive group sessions viewing clips; field interviews; observations of meetings/handovers	To clarify how staff enact patient safety in a specialist acute unit for advanced dementia. Safety was negotiated through transparent leadership and teamwork; practice-based learning and VRE enhanced early detection and non-pharmacological responses; personhood sometimes superseded rigid protocols (e.g., nurse-led code black, flexible routines); a shared purpose fostered joy and help-seeking; family engagement supported safety (excluded from staff-only mapping).
Dementia, medication and transitions of care	Louise S. Deeks; Gabrielle M. Cooper; Brian Draper; Susan Kurrle; Diane M. Gibson	2016	Australia; Mixed (acute hospitals; geriatric/a ged care interface); Acute hospital admissions/disc harges and ED with transitions to primary care and RACFs; multi-site urban/rural NSW/ACT focus	n=51 participants (carers; health professionals incl. hospital doctors, nurses, pharmacists, OT; GPs; Alzheimer 's Australia staff); Doctors (NR); Nurses (NR); AHPs (NR); Managers (NR)	Qualitative	None/NR	Individual semi-structured interviews (face-to-face/phon e) with hospital and community professionals; thematic analysis	To explore medication processes for people with dementia during acute episodes and transitions. Key findings: medication reconciliation is difficult and inconsistently resourced; discharge communication is error-prone (often drafted by least-informed junior doctors); after-hours discharges and short stays reduce counseling; IT systems are fragmented and access-restricted; multiple prescribers and RACF documentation requirements delay/compromise continuity; pharmacist roles are variably used; DAAs and self-administration can help but require assessment and oversight; cognitive identification and targeted training are needed.
Using sense-making theory to aid understanding of the recognition, assessment and management of pain in patients with dementia in acute hospital settings	Dawn Dowding; Valentina Lichtner; Nick Allcock; Michelle Briggs; Kirstin James; John Keady; Reena Lasrado; Elizabeth L. Sampson; Caroline Swarbrick; S. José Closs	2016	UK; Acute medicine; elderly medicine; vascular; stroke rehab; orthopaedic; elective/emergency surgery; continuing care; acute admissions (11 wards across 4 NHS hospitals); Acute hospital wards caring for older adults with dementia; bedside care, ward routines, MDT interactions	n=52 staff; 11 wards; 4 hospitals; Doctors (NR); Nurses (NR); HCAs (NR); AHPs incl. physiotherapists (NR); Specialist nurses (NR); Managers (NR)	Qualitative	None/NR	Ethnographic bedside observation; interviews; field notes; audits; thematic analysis	To revise the decision model for pain in dementia in acute care. Key findings: assessment is dynamic and contextual; clinicians use intuition plus selective analytic checks; "trial-and-error" management common; routines and staffing shape opportunities to notice/receive pain reports; tools alone don't trigger action without shared narratives and cumulative cues
Exploring dementia care in acute care settings: Perspectives of nurses and social workers on caring for patients with behavioral and psychological symptoms	Ruth E. Dunkle; Katherine Cavignini; Joonyoung Cho; Laura Sutherland; Helen Kales; Cathleen Connell; Amanda Leggett	2022	USA; Teaching; Tertiary; Acute; ED and inpatient units within a major academic tertiary acute-care hospital	n=17 staff (nurses=5; social workers=12); Doctors (0); Nurses (5); AHPs (12); Managers (0)	Qualitative	None/NR	Individual in-depth interviews; thematic analysis using RADaR	Qualitative comparison of nurses and social workers in ED/IP on managing BPSD. Both prioritize safety/medical needs while attempting person-centered care. Non-pharmacological strategies (redirection, de-escalation, groups, sitters, family involvement) are commonly used; medications sometimes used by nurses due to time/skill constraints. Key barriers: limited time/staff, lack of protected spaces, training gaps, discharge resource constraints, and coordination challenges. Facilitators: patient story–based tailoring, family engagement, team coordination, private rooms/1:1 sitters, group activities, and non-pharmacological calming strategies.
Using Signs and Symbols to Label Hospital Patients with a Dementia Diagnosis: Help or Hindrance to Care?	Katie Featherstone; Paula Boddington; Andy Northcott	2020	UK; Acute; Teaching; Medical Assessment Units and Trauma & Orthopaedic wards across five acute hospitals in England and Wales	Observed care over 684 hours; interviews: 192 ethnographic + 22 group (Trauma & Orthopaedics); 222 ethnographic (MAU); Doctors (NR); Nurses (NR); AHPs (NR); Managers (NR)	Qualitative	Symbolic interactionism; Grounded theory ethnography	Ethnographic observation; informal staff interviews; group interviews; review of ward boards/records	examine how bedside signs/symbols and profile forms are used to identify and support people with dementia on acute wards. Key findings: Signage schemes are common and publicly promoted, but are frequently inaccurate, left in place after transfers, or conflated with unrelated aids (e.g., red trays), narrowing attention to diagnosis and presumed dependency and obscuring individual needs. "This is me" forms exist but are rarely consulted. "Dementia-friendly" labels signal image rather than substantive practice change. Consequences include misclassification, dehumanizing assumptions (e.g., automatic "feeding"), reduced dialogue with patients, and missed rehabilitation opportunities. Accurate, person-focused assessment and training are needed; current technologies of attention risk invisibility and stigma.
Developing a Life Story Intervention for Older Adults With Dementia or at Risk of Delirium Who Were Hospitalized: Multistage, Stakeholder-Engaged Co-Design Study	Sarah J. Flessa; James D. Harrison; Roniela Turnigan; Megan Rathfon; Michael Chandler; Jay Newton-Small; Stephanie E. Rogers	2024	USA; Acute; Teaching; Acute Care for Elders (ACE) Unit, UCSF Medical Center; EHR integration context	n=27 stakeholders (Interdisciplinary (or Integrated) Care Team(IDT)=18; patients=3; care partners=6); Doctors (NR); Nurses (NR); AHPs (NR); Managers (NR)	Mixed methods	None/NR	Interviews; surveys	Co-designed an acute-care–adapted life story intervention with IDT to humanize patients and support tailored care. Staff anticipated benefits for rapport/orientation and discharge planning. Key implementation issues: need easy EHR access (Daily Rounds shortcut + Social History full text), concise bullet summaries, team training, champions, and privacy safeguards. Barriers include short length of stay, time pressure, competing priorities, variable specialty buy-in, and capacity/consent concerns.
Issues experienced while administering care to patients with dementia in acute care hospitals: A study based on focus group interviews	Risa Fukuda; Yasuko Shimizu; Natsuko Seto	2015	Japan; Acute; Six acute hospitals in western Japan, surgical and internal medicine wards, non ICU, mixed wards	n=50 nurses, eight focus groups with 6-10 per group; Doctors (0); Nurses (50); AHPs (0); Managers (0)	Qualitative	None or NR	Focus groups, audio recorded; brief participant questionnaire; field notes	Exploratory qualitative study with nurses only identified an interacting burdensome cycle, problematic behaviors in unfamiliar environments, recurrent need for family assistance often unavailable, and spillover effects on others, exacerbated by limited dementia training and experience, staffing and time pressure, infrastructure gaps, and weak interprofessional cooperation such as difficulty securing psychiatric input; nurses implement practical workarounds like sensor mats and bringing patients to the station while experiencing ethical tension regarding restraints and intensive treatments; Fig 2 schematizes theme interactions and shows emphasis on early detection but responses remaining reactive
Effects of a Person Centered Dementia Training Program in Greek Hospital Staff: Implementation and Evaluation	Mara Gkioka; Birgit Teichmann; Despina Moraitou; Sotirios Papagiannopoulos; Magda Tsolaki	2020	Greece; mixed: general hospital and university teaching hospital, acute; mixed acute wards across two metropolitan hospitals: surgery clinics, internal medicine, cardiology, neurology, psychiatry, ED, ICU, outpatient clinics, laboratories	n=242 staff trained; n=232 pre and post measures; n=103 three month follow up; Doctors (NR); Nurses (220); AHPs (6); Managers (10)	Mixed methods	Holton's evaluation model; Person centered care orientation	surveys with validated questionnaires DAS, CODE, STAI, DKAT2 at T1 T2 T3; open ended questions with thematic analysis	Training improved attitudes, confidence and knowledge with partial maintenance at three months; many staff integrated PCC communication and selected tools; implementation was challenged by staffing shortages, time pressure, physical environment and hierarchical culture; organizational support and train the trainer were positioned as key enablers for spread and sustainability; Table 2 reports mean scores, Figures 3 to 6 display longitudinal changes

Title	Author(s)	Year	Country and setting	Sample and professional groups	Study design	Theoretical framework	Data collection	Study aim and key findings
The Person, Interactions and Environment Programme to improve care of people with dementia in hospital: a multisite study	Godfrey M; Young J; Shannon R; Skingley A; Woolley R; Arrojo F; Brooker D; Manley K; Surr C	2018	England; Acute, teaching NHS hospitals; Acute wards across 10 wards in 5 NHS trusts: care of older people, trauma orthopaedics, dementia and rehabilitation wards	mixed units: n=10 wards; 56 staff interviews; 104 staff survey respondents; Doctors (NR); Nurses (NR); AHPs (NR); Managers (NR)	Mixed methods	Normalisation Process Theory (NPT)	Interviews; ethnographic observation; staff questionnaires; process documentation; implementation meeting observation; events log	Goal was to evaluate PIE implementation and identify what shapes practice change. Implementation depended on micro leadership constellation driver plus facilitator plus team, and meso macro fit with trust priorities and resourcing. Full adoption occurred on two wards with strong dementia specialist leadership and practice development support; elsewhere, staffing pressures, ward closures, winter pressures, and absence of drivers stalled work. Observation and reflective cycles improved visibility and coherence when enacted.
How do emergency department staff respond to behaviour that challenges displayed by people living with dementia? A mixed-methods study	Goodwin L, Liddiard C, Manning S, Benger JR, Carlton E, Cheston R, Hoskins R, Taylor H, Voss S	2023	UK; acute; EDs of three NHS Hospital Trusts in Southwest England	n=52 staff survey; n=13 staff interviews; Doctors (22); Nurses (8); AHPs (NR); Managers (NR)	Mixed methods	None/NR	Online survey; semi structured telephone interviews; descriptive statistics; thematic analysis	ED described as a perfect storm of triggers and constraints; staff report frequent behaviour that challenges, limited ED-specific training, resource shortfalls, and inconsistent, improvised responses. One to one care, rapid assessment, distraction and quieter spaces perceived as helpful but hard to deliver. Staff favor an ED-specific, stepwise checklist and better cross service information to prevent escalation, with caution about added paperwork and feasibility under pressure
Balancing the needs of individuals and services in cancer treatment for people with dementia: A focused ethnographic study	Alys Wyn Griffiths; Laura Ashley; Rachael Kelley; Fiona Cowdell; Michelle Collinson; Ellen Mason; Hayley Inman; Ann Henry; Amanda Farrin; Claire Surr	2021	UK; acute, teaching; oncology and radiotherapy outpatient departments: reception, waiting areas, treatment rooms, two NHS hospital trusts	n=19 staff; Doctors (2); Nurses (8); AHPs (9); Managers (NR)	Qualitative	None/NR	participant observation; informal conversations; semi structured interviews; medical notes review	Aim: understand how oncology staff balance dementia related individual needs with service targets and processes in outpatient cancer pathways. Key findings: person centred practices improved perceived appropriateness and acceptability, but feasibility was constrained by time, staffing, target driven workflows, reactive adjustments, and inconsistent identification and documentation. Proactive planning, flexible appointments, continuity, tailored communication, and adapted ID procedures enabled better navigation.
Supporting general hospital staff to provide dementia sensitive care: A realist evaluation	Melanie Handley; Frances Bunn; Claire Goodman	2019	UK; acute, general hospital; Two NHS trusts, East of England, inpatient wards. Site 1: dedicated ward for patients with dementia addressing medical and mental health needs. Site 2: one-to-one healthcare assistant team with dementia specialist nurse support working across wards.	n=36 staff; Doctors (7); Nurses (14); AHPs (3); Managers (NR)	Qualitative	Realist evaluation	Interviews; non-participant observation; medical notes review; NPI-Q; documentary review	Aim: explain factors that support staff to provide dementia sensitive care and resulting outcomes. Key findings: six linked mechanisms supported better care when staff had knowledge and authority, role-relevant training with reflection, credible clinical experts and senior staff promoting patient-focused practice, endorsement to spend time with patients, person-centred risk management, and recognition of dementia care as skilled work. Effective interactions reduced distress and supported orientation. Training and extra time alone were insufficient when ward pressures and safety concerns dominated or when dementia care was not valued as skilled core work.
Exploring ways to enhance pain management for older people with dementia in acute care settings using a Participatory Action Research approach	Deirdre Harkin; Vivien Coates; Donna Brown	2022	UK; acute, general hospital; ED, Acute Medical Unit, Surgical Ward in a city-based acute general hospital	Focus groups n=14 staff; case study pathway observations involved staff: nurses n=20; AHPs n=8; pharmacists n=2; doctors n=5; staff interviews n=6; Doctors (NR); Nurses (10); AHPs (2); Managers (2)	Qualitative	PARiHS; Participatory Action Research	Exploratory audit; non-participant observation; focus groups; semi-structured interviews; nursing and medical notes review	To understand complexities of managing pain for older people with dementia across acute settings and assist staff to improve practice. Key findings: barriers included not knowing the patient, competing priorities and target-driven culture, limited knowledge of pain in dementia, and poor assimilation of available information. Facilitated reflection and practical tools enabled earlier identification, routine use of appropriate pain assessment, family engagement, and better information flow, supporting more person-centred pain management.
Barriers and enablers to implementing 'DEALTS2' simulation-based train-the–trainer dementia training programme in hospital settings across England: a qualitative study	Michelle Heward; Michele Board; Ashley Spriggs; Dina Blagden; Jane Murphy	2021	England; acute, NHS hospital trusts; Trainer-led dementia education for hospital staff in acute settings, national TTT workshops with local delivery	n=17 trainers interviewed (6–8 months post-TTT); context: TTT workshops n=199 trainers; Doctors (NR); Nurses (NR); AHPs (NR); Managers (NR)	Qualitative	Kirkpatrick model; Kolb experiential learning; Humanising Values Framework; Dementia Training Standards Framework (Tier 2)	semi-structured telephone interviews with trainers	To explore barriers and enablers trainers experienced when implementing DEALTS2 and perceived impacts on training delivery and staff practice. Key findings: implementation depended on flexible simulations, management support, adequate resources, and sufficient time; barriers included short training slots, difficulty releasing staff, and variable prioritisation of dementia training. Trainers reported enriched teaching practice and perceived improvements in staff empathy and application to care.
Person-centred oral hydration care for older people with dementia admitted to acute hospital wards: Empirical research qualitative	Shanlee Higgins; Lesley Baillie; Calvin Moorley; Fiona Nolan	2023	UK; teaching, acute; Three acute wards: acute medicine, elderly medicine, trauma and orthopaedics	Staff interviews: n=28 ward staff; n=4 ward leaders; n=5 organisational leaders; Observations: 132 h; Clinical records: n=26; Doctors (NR); Nurses (NR); AHPs (NR); Managers (NR)	Qualitative	Person-centred care framework, VIPS	Observation; semistructured interviews with staff, ward leaders, organisational leaders; documentary analysis of nursing and MDT records and policies	Person-centred hydration was obscured by organisational priorities and routinised, outsourced drink rounds. Roles were siloed, with variable hydration and dementia knowledge. Barriers clustered around communication gaps, limited direct actions outside routines, constrained resources and locked or poorly stocked areas, and unsupportive social and physical environments. Facilitators included person-centred communication and timely prompting, integrating hydration into other activities, clarifying team roles, involving managers to signal priority, and attention to oral hygiene and suitable cups.
The experience of hospital staff in applying the Gentle Persuasive Approaches to dementia care	Lillian Hung; Cathy Son; Rebecca Hung	2019	Canada; Teaching, acute; Three mental health units and four medicine units, inpatient wards, large metropolitan teaching hospital	Training n=310 staff; Survey n=297 staff; Focus groups n=24 staff; Doctors (3); Nurses (145); AHPs (10); Managers (2)	Mixed methods	Person centred nursing framework; Senses framework; PARiHS	Surveys; focus groups	Staff reported GPA shifted attitudes, built skills, and supported team based person centred practices. Joint training, shared language, and ongoing facilitation enabled application, while time pressure, short staffing, and variable leadership support hindered consistent implementation.
Nurses' knowledge and beliefs on pain management practices with hospitalised persons living with dementia: A qualitative descriptive study	Beverly Ingelson; Sherry Dahlke; Hannah O'Rourke; Kathleen F. Hunter	2024	USA; Acute, teaching; Single acute hospital, Southern California; general medicine; postoperative surgery; telemetry units	n=12 nurses; Doctors (NR); Nurses (12); AHPs (NR); Managers (NR)	Qualitative	None/NR	Semi structured interviews	Aims had two themes: improvising pain assessment and administration hesitancy. Nurses reported challenges assessing non verbal PLWD, inconsistent use of dementia specific tools, documentation gaps, and hesitancy to give analgesics due to fears of confusion or respiratory depression. Workload, communication barriers, and missing external records hindered timely pain control. Facilitators included nurse to nurse handoff, using multiple behavioral indicators, and involving family to interpret usual behaviors.
Living with dementia in hospital wards: a comparative study of staff perceptions of practice and observed patient experience	Innes A., Kelly F., Scerri C., Abela S.	2016	Malta; Acute general hospital; Two hospital wards caring for people with dementia, mixed acuity, in-hospital	n=69 staff; n=16 patients observed; Doctors (NR); Nurses (NR); AHPs (NR); Managers (NR)	Mixed methods	None/NR	Observation with Dementia Care Mapping; Environmental Audit Tool; Staff questionnaires: PCAT and PCQ-S	Two-wave ward evaluation found small environmental and practice improvements after feedback and mentoring, but care remained task-first and rushed with limited positive engagement. Restraint and routine pads persisted, communication barriers existed for Maltese-only speakers, and staff perceptions often conflicted with observed practice.
Barriers and facilitators to caring for patients living with dementia in the emergency department: a qualitative study	Jelinski D., Reich K., Lang E., Holroyd-Leduc J., Goodarzi Z.	2025	Canada; Acute, emergency department; EDs across four adult hospitals, Calgary Zone, Alberta	n=11 healthcare providers; Doctors (2); Nurses (5); AHPs (4); Managers (0)	Qualitative	TDF and Behavior Change Wheel (COM-B)	Semi-structured interviews	Providers described ED environment and resource constraints, time pressure, skills gaps and limited dementia-specific guidance as core barriers. Facilitators were collaborative team approaches, collateral information from care partners and EMS, access to geriatric staff, attention to non-medical needs, and person-centred care. Staffing initiatives and allied health pilots helped but were hard to sustain.

Title	Author(s)	Year	Country and setting	Sample and professional groups	Study design	Theoretical framework	Data collection	Study aim and key findings
Nurses' experiences of delivering acute orthopaedic care to patients with dementia	Jensen AM; Pedersen BD; Wilson RL; Olsen RB; Hounsgaard L	2019	Denmark; Teaching, acute; Acute orthopaedic hip fracture unit, university hospital; two four-bed and five single-bed rooms, 13 beds	n=8 staff; Doctors (0); Nurses (6); AHPs (0); Managers (2)	Qualitative	Kitwood person-centred care; VIPS; Interactional Nursing Practice	Small paired group interviews	Investigates nurses' experiences caring for people with dementia in an acute orthopaedic ward. Key findings: somatic priorities and task culture, information drowning in EHR, busy and noisy environment, and negative preconceptions hinder person-centred dementia care; nurses use ad hoc oral handover, pocket notes, single rooms, and allocating calm staff as pragmatic enablers; frustration, uncertainty, and limited dementia knowledge noted; call for person-centred training and EHR cueing.
Health professional perspectives on systems failures in transitional care for patients with dementia and their carers: a qualitative descriptive study	Kable A; Chenoweth L; Pond D; Hullick C	2015	Australia; Acute, tertiary; Acute tertiary hospital discharge planning with community GP and RACF interfaces, regional NSW	n=33 staff; Doctors (NR); Nurses (NR); AHPs (NR); Managers (NR)	Qualitative	None/NR	Focus groups	To describe health professionals' views on discharge and transitional care for people with dementia. Key findings: early discharge pressure, scarce community services, and incomplete or misdirected discharge documentation undermine continuity and safety; JMOs often draft summaries without full overview or guidance; medication reconciliation and rationale frequently missing; RACFs and GPs receive late or inadequate notification; community services have long waits and restrictive eligibility. Staff propose routine dose administration aids, home medicines reviews, clearer coordination with RACFs, and improved discharge content.
Nurses' perceptions in caring for people with dementia in Dutch acute hospitals	Annette Keuning-Plantinga; Petrie F. Roodbol; Wim P. Krijnen; Evelyn J. Finnema	2022	The Netherlands; Acute, university, nonuniversity teaching, general; Mixed acute hospital wards, including medical, surgical, geriatric, ICU and other wards across seven Dutch acute hospitals	n=229 nurses; Doctors (0); Nurses (229); AHPs (0); Managers (0)	Quantitative descriptive	None/NR	Online questionnaire survey using GerINCQ subscales and Hynninen subscales, plus two open-ended questions	Survey of Dutch hospital nurses found general preventive care common but dementia-specific interventions less used, frequent use of medical and physical restrictive measures, mixed attitudes and perceptions, and time and staffing pressures. Nurses who completed dementia training in the last year reported more positive attitudes, more responsibility for delirium and behavioural problems, and better communication practices.
Radiographers' experiences in working with patients living with dementia in Norway: A qualitative study	E. Kjelle; C. Eikefet; C.C. Chilanga	2024	Norway; Acute, university and regional hospitals; Imaging departments: MRI, CT, general imaging, nuclear medicine in three hospital trusts	n=8 radiographers; Doctors (0); Nurses (0); AHPs (8); Managers (0)	Qualitative	None/NR	Semi-structured interviews	Explores radiographers' experiences with PWD: overall examinations usually proceed, but communication, safety screening, missing dementia flags in referrals, and acute or advanced exams pose challenges. Facilitators include allocating time, calming environment, caregiver collaboration, and tailored communication; formal dementia-specific training and departmental protocols are lacking.
Perioperative Nurses' Experience of Working With Persons Living With Dementia	Mariya Kovaleva; Abigail C. Jones; Cornelia Frances Garrard; Jana Lauderdale; Mary S. Dietrich; Peace Johnson; Cathy Maxwell	2025	USA; Acute, tertiary academic medical center; Perioperative units in an academic hospital: trauma ICU and step-down, surgical step-down, surgical services including urology, orthopedics	n=20 nurses; Doctors (0); Nurses (20); AHPs (0); Managers (0)	Qualitative	Adaptive Leadership Framework	Individual semi-structured telephone interviews; conventional content analysis	Explores perioperative nurses' experiences caring for PLWD: work is complex and time-intensive with communication challenges, routine disruption, safety risks and pain assessment difficulties. Nurses rely on sitters, occasional restraints, proximity and observation, environmental calming and planning ahead. Family presence and tailored activities help. Gaps in dementia knowledge and skills and limited staffing and policies constrain feasibility; nurses recommend strengthened education, staffing and unit protocols.
Implementation of a model of care for hospitalised older persons with cognitive impairment (the Confused Hospitalised Older Persons program) in six New South Wales hospitals	Susan Kurrle; Sigrid Patterson; Cath Bateman; Anthea Temple; Anne Cumming; Glen Pang	2019	Australia; Acute, public, mixed metropolitan regional rural; Multiple acute wards across 6 NSW hospitals: ED, medical, surgical, aged care, subacute rehabilitation	Medical record audit n=743 records; Staff survey pre=432, post=283; Environ mental audits=6 sites; Clinical leads interviewed n=6; Doctors (NR); Nurses (NR); AHPs (NR); Managers (NR)	Mixed methods	None/NR	Medical record audits; staff knowledge and care confidence surveys; environmental audits; group interview with site clinical leads	Aim: evaluate CHOPs implementation to improve recognition, risk assessment and management of cognitive impairment in acute hospitals. Key findings: significant improvements in cognitive screening within 24 h, delirium risk identification, assessment, repeated screening, interactions with families, delirium coding at discharge, staff education and confidence, and environmental supports. Physical restraint use decreased; antipsychotic prescribing unchanged overall. Enablers: executive support, clinical leadership, education, environmental changes, prompts. Barriers: competing priorities, staff time/release, variable engagement, project lead capacity, IT changes, low profile of delirium/dementia.
Redesigning acute care for cognitively impaired older adults: Optimizing health care services	Michael A LaMantia; Malaz A Boustani; Shola Jhanji; Mungai Maina; Arif Nazir; Frank C Messina; Amie Frame; Catherine Alder; Joshua Chodosh	2016	USA; Mixed, teaching, public safety-net system; Hospital outpatient clinics attached to the university-affiliated safety-net hospital: geriatrics consultation clinics with social work support, EMR-linked to inpatient teams; interface with ACE inpatient consult service	n=22 staff, 3 focus groups; Doctors (2); Nurses (8); AHPs (11); Managers (NR)	Qualitative	None/NR	Focus groups, audio-recorded and transcribed; thematic analysis	Hospital outpatient clinic focus: physicians describe a persistent disconnect with inpatient teams that clinics must bridge, including contradictory discharge instructions and duplicated referrals. EMR alerts to clinics and standardized ACE packets enable timely specialist clinic follow-up. Clinic social work mitigates insurance and transport barriers. Education on delirium improves clinic staff capability. Time pressure and resource strain remain recurring impediments for clinic coordination and medication reconciliation.
Dementia care across a tertiary-care health system: What exists now and what needs to change	Amanda Leggett; Cathleen Connell; Leslie Dubin; Ruth Dunkle; Kenneth M. Langa; Donovan T. Maust; J. Scott Roberts; Beth Spencer; Helen C. Kales	2020	USA; Tertiary, academic health system; Mixed hospital settings: main hospital inpatient wards including AIP, Medical ED and Psychiatric Emergency Services, and hospital-attached geriatrics outpatient clinics	49 interviews with stakeholders across settings (staff and families); Doctors (NR); Nurses (NR); AHPs (NR); Managers (NR)	Qualitative	None/NR	Semi-structured qualitative interviews, recorded and transcribed; grounded/them atic analysis	map strengths and challenges of dementia care across a tertiary system and propose improvements. Key findings: hospital ED and inpatient environments are often chaotic and poorly suited for PLWD; dementia is inconsistently identified in records; competing demands, limited training, and turnover impede care; sitters and antipsychotics are frequently overused; placement after hospitalization is difficult. Facilitators include consult/liaison services, Elder Life volunteers, crisis lines, and multidisciplinary expertise. Recommendations: prepare for rising demand, implement web-based staff training, create a dementia care management program to link services, provide outpatient education, and repurpose sitters as elder life specialists.
Understanding and integrating the needs and preferences of people living with dementia in the inpatient setting: a qualitative study	Alyse Lennox; Denise Goodwin; Felicity Leavold; Renae Nicol; Velandai Srikanth; Darshini Ayton; Madeleine Berends; Myra Thiessen; Daphne Flynn; Chris Moran	2025	Australia; Acute, teaching, geriatrics-focused ward; Inpatient acute geriatrics unit, ACE Unit, university-affiliated tertiary metropolitan teaching hospital	n=11 staff interviews, single hospital site; Doctors (1); Nurses (9); AHPs (1); Managers (NR)	Qualitative	None/NR	Semi-structured interviews via phone or Zoom; audio-recorded, transcribed; thematic analysis with framework approach	Staff report that integrating needs and preferences requires early family input, clear ownership, and MDT communication. Barriers include unclear responsibility for gathering information, time and staffing constraints, surface-level or reactive tools, and difficulty accessing behaviour plans. Facilitators include MDT behaviour huddles, effective handover, visible bedside prompts, role modelling, and targeted training. A pre-emptive hospital admission kit is viewed as a promising solution if workflows, access, and updating are defined.
The assessment and management of pain in patients with dementia in hospital settings: a multi-case exploratory study from a decision making perspective	Valentina Lichtner; Dawn Dowding; Nick Allcock; John Keady; Elizabeth L. Sampson; Michelle Briggs; Anne Corbett; Kirstin James; Reena Lasrado; Caroline Swarbrick; S. José Closs	2016	UK; Acute, mixed teaching/secondary hospitals; Mixed acute wards: vascular surgery, surgery/orthopaedic, acute medical admissions, medicine for the elderly, care of the elderly, stroke rehabilitation	Staff interviews n=52; bedside observation 170 h across 4 hospitals and multiple wards; Doctors (8); Nurses (31); AHPs (2); Managers (NR)	Qualitative	Decision-making theory (judgement and decision making)	Non-participant observations; semi-structured staff interviews; audits of patient records; analysis of ward documents	Study aimed to understand how staff recognise, assess and manage pain in hospitalised patients with dementia. Key findings: staff rely on self-report but dementia-related communication limits, brief encounters, and ward routines hinder cue detection and reassessment; information is fragmented across paper silos so staff must reconstruct an overall picture using memory and time; pharmacological management dominates with trial-and-error titration and limited non-pharmacological options; PRN is ineffective when patients do not request; observational tools exist but are rarely used or become tick-box exercises; enablers include clinical knowledge, standard protocols, drug charts as shared reference and access to specialist pain teams, but require integration and time for interdisciplinary communication.

Title	Author(s)	Year	Country and setting	Sample and professional groups	Study design	Theoretical framework	Data collection	Study aim and key findings
What influences decisions about ongoing stroke rehabilitation for patients with pre-existing dementia or cognitive impairment: a qualitative study	Longley V; Bowen A; Peters S; Swarbrick C	2018	UK; acute and inpatient rehabilitation hospitals; Acute stroke unit; inpatient stroke rehabilitation units	n=23 staff; Doctors (2); Nurses (3); AHPs (18); Managers (NR)	Qualitative	None/NR	Semi-structured interviews (individual); thematic analysis	Clinicians weigh understanding the person, limited dementia knowledge, predicting rehab potential, organizational constraints, and perceived roles. Short timeframes and improvement-focused models often lead to ending rehab or shifting to compensatory management. Training gaps and role misunderstandings constrain decisions; MDT collaboration, tailored communication, environmental adjustments, family input, and expert consultation help accommodate cognitive impairment within rehab.
Facilitators, Barriers, and Solutions in Pain Management for Older Adults with Dementia	Alícia Minaya-Freire; Anna Ramon-Aribau; Gemma Pou-Pujol; Meritxell Fajula-Bonet; Mireia Subirana-Casacuberta	2020	Spain; acute, geriatric, teaching; Acute Geriatric Unit, inpatient acute geriatric ward, university hospital	n=10 nurses; Doctors (NR); Nurses (10); AHPs (NR); Managers (NR)	Qualitative	None/NR	questionnaire with open-ended items; focus groups	Qualitative PAR with AGU nurses identified facilitators: adapted communication and professional experience; barriers: time pressure and workload, knowledge and skills gaps, pain not prioritized as fifth constant, under-recording, and communication gaps across shifts. Nurses proposed a pragmatic training plan and an EHR pain register to standardize assessment and improve continuity.
Privileges, and Permissions: Theorising Intersectionality and Cultures of Control in the Care of People Living With Dementia in Acute Hospital Settings	Mwale S; Northcott A; Featherstone K	2025	UK; acute, teaching; general medical wards and medical assessment units (NHS acute hospitals), inpatient, mixed bays and single rooms	3 hospitals; 6 wards; ~30 observation days per ward (≈180 ward-days); Doctors (NR); Nurses (NR); AHPs (NR); Managers (NR)	Qualitative	Intersectionality (Collins, Crenshaw); Cultures of control (Garland); Power/discipline (Foucault)	non-participant ethnographic observation across shifts and weekends; field notes (~500,000 words); observation of handovers, MDT meetings, admissions boards and ward documents	Multi-sited hospital ethnography shows ward cultures of control shaping who may walk the ward and who is contained. Intersectional identities (ethnicity, social class, gender) contour staff responses: security-guard surveillance and tighter rules applied to some, while others receive conversational engagement and wider privileges. Control technologies (one-to-one observers, security, chair alarms, single rooms) support timetabled bedside work but intensify distress and may steer discharge pathways toward institutional settings. Staff sometimes use reassurance or activities to enable movement for privileged patients.
When people living with dementia say 'no': Negotiating refusal in the acute hospital setting	O'Brien R; Beeke S; Pilnick A; Goldberg SE; Harwood RH	2020	UK; acute, teaching; Healthcare of the Older Person wards, acute inpatient hospital	n=26 HCPs filmed across 41 encounters; Doctors (9); Nurses (11); AHPs (6); Managers (NR)	Qualitative	Conversation Analysis framework: entitlement and contingency	Video-recorded observations with transcripts; conversation analysis	To examine how HCP request formats precede PLWD refusal or acceptance in acute wards. Refusals were common, often following low or moderate entitlement requests that highlighted patient choice or ability. Requests showing higher entitlement, softened by lowering contingencies (e.g., minimisers like "just", invitations like "let's", statements of need plus permission checks), were more likely to precede acceptance and completion of care tasks.
"Getting to Know Them": Person-Centered Care for Patients With Dementia in Acute Care	Osuoha P; Masoud SS; Leibas M; Cleveland LM; Reed CC; Piernik-Yoder B; White CL	2021	USA; acute, teaching; Acute inpatient, medicine, rehabilitation, and neuroscience units	n=49 staff (24 RNs, 25 patient care technicians); Doctors (0); Nurses (49); AHPs (0); Managers (0)	Qualitative	None/NR	Focus groups (RN-only, PCT-only, mixed); moderator-led with observer notes	explored nurses' and patient care technicians' perspectives on delivering person-centered care in acute wards. Staff valued "getting to know" the person and described three determinant clusters: communication (handoff quality, team alignment, tone, redirection), education (limited dementia-specific training but strong motivation to learn, preference for practical, role-play-based sessions), and care environment (task-driven pace, staffing ratios, fall-risk placement, need for activity materials, consistent assignation, and family presence). Overall, PCC intent was strong but operationalization was constrained by time pressure, task-first routines, and inconsistent interprofessional communication, while practical workflow tweaks, environmental cues/activities, family involvement, and targeted education were seen as workable enablers.
Improving emotional wellbeing for hospital-based patients with dementia	Petty, S., Griffiths, A., Coleston, D.M. & Dening, T.	2021	UK; Geriatric, psychiatric, long-stay hospital; Two long-stay dementia wards in an independent UK hospital providing dementia care with mental and physical comorbidities; multidisciplinary support available by referral	n=12 staff; Doctors (NR); Nurses (12); AHPs (NR); Managers (NR)	Qualitative	None/NR	Semi-structured interviews	Qualitative interviews with nursing staff identified that emotion-focused care is achievable when embedded into routine tasks, supported by leaders, team learning and easy access to personal information; barriers included intense ward environments, competing priorities and time pressure, lack of a single method to calm distress, difficulty specifying and enforcing emotion-focused actions and the emotional toll on staff; facilitators included person knowledge, tailored communication, nonverbal presence and touch, family involvement, therapeutic resources, peer learning and leaders normalising "just sitting with" patients.
Nurses' perspectives on shared decision-making in the daily care of hospitalized patients with dementia: An exploratory qualitative study	Plantinga A; Roodbol PF; van Munster BC; Finnema EJ	2024	Netherlands; Acute, teaching, general hospitals; University, non-university teaching and regional hospitals; surgical and non-surgical wards; ICU and ED excluded	n=14 registered nurses; Doctors (NR); Nurses (14); AHPs (NR); Managers (NR)	Qualitative	Phenomenology, Colaizzi's seven-step method; none stated for implementation	In-depth semi-structured digital interviews	aim: SDM is used variably: nurses apply it unconsciously in routine care and more consciously for multidisciplinary decisions, but often without explicit goal-talk. Nurses report role ambiguity, time constraints, communication challenges and perceived low competence, while also acting as facilitators and advocates by organizing family meetings, timing conversations, adapting communication and informally assessing capacity. Relatives' involvement is crucial; triadic consultations and scheduled family meetings support SDM. Training on SDM steps, goal discussions and nuanced capacity assessment is needed.
Co-development of BRAIN-TRK: Qualitative examination of acceptability, usability and feasibility of an App to support nurses' care for patients with behavioural and psychological symptoms of neurocognitive disorders in hospital	Redley B; Richardson B; Peel C; Ockerby C; Rawson H; Tomlinson E; Hutchinson A	2019	Australia; acute public hospital; General medicine inpatient wards at two hospital sites in a large Australian public health service, acute hospital settings caring for older adults with neurocognitive disorders and behavioural and psychological symptoms	n=25 nurses in interviews and focus groups; 12 nurse facilitators; 2 nurse unit managers; 38 observed patients; 32 App patients; Doctors (0); Nurses (25); AHPs (0); Managers (2)	Mixed methods	Integrated knowledge translation approach; Kawamoto clinical decision support components; Sekhon acceptability construct and usability testing principles	naturalistic nonparticipant observations of nurse care; App usage logs; individual interviews; focus groups with ward nurses and nurse unit managers	Aim: to co-develop the BRAIN-TRK point-of-care App as part of a KT intervention and evaluate its acceptability, usability and feasibility for nurses caring for hospitalised patients with neurocognitive disorders and behavioural and psychological symptoms. The App enabled nurses to screen cognition and BPS, document preferences, and receive tailored evidence-based strategies, and was generally viewed as useful, easy to navigate and pitched at a basic practical level, especially after preparation and repeated use. However, perceived additional workload, time pressure, duplicate documentation, inconsistent use across shifts, resistance to technology and device issues limited uptake and completion of evaluation steps, indicating that stronger integration with routine documentation, clearer expectations, ongoing support and cultural change towards bedside technology are needed for sustainable dementia-friendly hospital care implementation.

Title	Author(s)	Year	Country and setting	Sample and professional groups	Study design	Theoretical framework	Data collection	Study aim and key findings
Working with a robot in hospital and long-term care homes: staff experience	Ren LH; Wong KLY; Wong J; Kleiss S; Berndt A; Mann J; Hussein A; Hu G; Wong L; Khong R; Fu J; Ahmed N; Nolte J; Hung L	2024	Canada; acute hospital; Public hospital dementia care unit using telepresence robots for older adults with dementia; long term care home data reported in paper but excluded from DFHC extraction	n=22 staff across one hospital and four LTC homes (hospital n=13; LTC n=9); four focus groups and 11 individual interviews; Doctors (0); Nurses (8); AHPs (8); Managers (6)	Qualitative	CFIR; Collaborative Action Research	focus groups; semi structured individual interviews; regular staff check ins; observations and field notes; research team meeting notes	Aim: to explore staff experiences and views on implementing telepresence robots in long term care and hospital settings for older adults with dementia, focusing on perceived facilitators and barriers. Key findings for the hospital dementia unit were that tailored training and ongoing support, including engaging demonstrations, staff champions and recognition, helped move staff from fear and uncertainty to confidence using the robot; convenient features such as simple operation, QR codes and direct family control meant calls could be set up in minutes without substantial impact on workload and supported independent, real time communication that staff felt aligned with person centred care. However, barriers such as uneven internal communication about the robot, limited in person training during pandemic periods, diverse learning needs not fully met by email based materials, unstable Wi Fi, staffing shortages, coordination demands for shared devices and safety and space constraints in small rooms limited consistent use and integration into routine workflows. Structural supports at micro, meso and macro levels were seen as necessary to sustain robot use as part of dementia friendly hospital care.
Agitation near the end of life with dementia: An ethnographic study of care	Sampson EL; Stringer A; La Frenais F; Higgins S; Doyle MJ; Laybourne A; Livingston G; Leavey G	2019	UK; acute, district general; acute, teaching; Older adult medical wards in an outer city district general hospital and older adult wards in a large teaching hospital, south east England, plus two care homes not included in DFHC hospital extraction	n=20 people with dementia observed (hospital and care homes); n=14 hospital staff interviewed or observed (Hospital A n=9, Hospital B n=5); Doctors (1); Nurses (8); AHPs (3); Managers (1)	Mixed methods	Ethnographic inductive analysis; explanatory models framework for interviews; positional theory used to interpret cultural and structural context of staff responses	Structured non participant observation with two minute interval behaviour coding; structured agitation checklist based on the Cohen Mansfield Agitation Inventory; detailed field notes on ward context and interactions; semi structured staff interviews	Aim was to explore how staff in hospitals and care homes understand and manage agitation in people with dementia nearing the end of life; in hospital wards, staff articulated and enacted two overlapping explanatory models, a moral judgement model where agitation was viewed as purposeless or bad behaviour and primarily as a threat to staff, and a needs based model where agitation was interpreted as meaningful distress linked to unmet physical, emotional or environmental needs; the moral judgement model was more common in the understaffed district general hospital with minimal dementia training and stigmatised older peoples wards, and was associated with fear of patients, avoidance, looking but not intervening, low levels of comforting responses, and reliance on security; the needs based model was more evident in the teaching hospital with a dementia lead, dementia training and environmental cues, and was associated with more empathic, investigative and tactile comforting responses, reframing agitation as distress behaviors, greater social time with patients and more consistent person centred care; organisational resources, staffing, training, leadership and ward culture strongly influenced which model predominated and thus the quality of agitation care
Dementia care in acute hospitals: A qualitative study on nurse managers' perceived challenges and solutions	Scerri A; Innes A; Scerri C	2020	Malta; acute general hospital; Acute medical wards in single public general hospital, nurse managers responsible for 11 adult medical wards admitting older adults with dementia and other acute conditions	n=16 nurse managers (two focus groups) covering 11 acute medical wards; Doctors (0); Nurses (9); AHPs (0); Managers (7)	Qualitative	Adapted McKinsey 7 S organisational model including Space, theoretically driven approach following Meyer and Ward	Focus groups	Aim was to explore nurse managers' perceived challenges when caring for patients with dementia in acute hospital wards and to identify possible solutions; nurse managers reported that hospital strategy remained focused on acute episodes and bed throughput despite high prevalence of dementia, with limited alignment to community and long term care and a perception that hospitals were being used as an access point to long term care; a rigid, medically dominated structure and task or staff centred organisational culture meant nurses' observations about social issues or cognitive status were often discounted and person centred dementia care was hard to prioritise; hospital systems such as fixed routines, repeated intra hospital transfers, poor coordination between wards and services, unclear cognitive assessment pathways and weak implementation of restraint guidelines were seen as creating confusion and risk for people with dementia; the physical environment lacked dementia friendly design features, such as colour coding and clear signage, making orientation and safe mobility difficult; shared values problems included ageist attitudes, labelling patients as confused or social cases and low interest in dementia training; autocratic leadership styles, chronic nurse shortages, fluctuating staffing levels, lack of support staff and limited dementia specific skills further constrained care; proposed facilitators included using local prevalence data and audits to influence strategy, reducing ward transfers by fast tracking people with dementia from emergency or admission units to medical wards, redesigning ward environments with colour coding, signage and safety technologies, adopting more democratic and transformational leadership styles, creating dementia practice nurse and champion roles, involving volunteers and transport aides, and providing continuous, empathy focused dementia and geriatric care training to shift shared values towards person centred care
Creating a toolkit with stakeholders for leveraging tablet computers to support person-centred dementia care in hospitals	Shadarevian J; Chan C; Berndt A; Son C; Gregorio M; Horne N; Mann J; Wallsworth C; Chow B; O'Neill R; Hung L	2020	Canada; acute, psychiatric; Older adult mental health unit (19 beds, 2–6 month stays) and two 20 bed acute medical units for older adults in two large urban hospitals	n=41 staff (35 nurses and care staff; 3 AHPs; 3 administrative or managerial staff); Doctors (0); Nurses (35); AHPs (3); Managers (3)	Qualitative	Participatory Action Research; ASK ME co production strategies	Staff focus groups; stakeholder interviews with nurse leader, patient and family partners; research team meetings with collaborative reflection and planning	Aim: to identify key barriers and enabling factors for implementing a tablet based family video intervention to support person centred dementia care in hospitals and to develop a practical toolkit for staff and families. Key findings: implementation hinged on engaging staff and stakeholders in co producing the toolkit and adapting the intervention to local ward workflows and infrastructure. Main barriers were inexperience with tablets among staff and families, anxiety about operating devices and finding videos, mechanical instability and awkward positioning of tablets at the bedside, information privacy and data access concerns, difficulties in personalising content within task focused routines, and technical problems such as Wi Fi connectivity, power demand and malfunctioning applications. Enabling factors included simple, co designed educational materials, on ward champions, hardware solutions such as a mechanical arm on a mobile stand, clear privacy procedures and data handling rules, and organisational readiness to invest in devices and staff capacity to deliver person centred dementia care using everyday technology

Title	Author(s)	Year	Country and setting	Sample and professional groups	Study design	Theoretical framework	Data collection	Study aim and key findings
Implementing the PIE (Person, Interaction and Environment) programme to improve person-centred care for people with dementia admitted to hospital wards: a qualitative evaluation	Ann Skingley; Mary Godfrey; Rosemary Henderson; Kim Manley; Rosie Shannon; John Young	2021	UK; Acute NHS general hospitals; 10 acute hospital wards across five NHS trusts: orthopaedic, frailty, step-down rehabilitation, acute medical ward for patients with dementia, older people or orthopaedic mixed wards, stroke unit, enhanced recovery ward for patients with dementia, care of older people wards	n=10 wards; number of participa ting staff NR; Doctors (NR); Nurses (NR); AHPs (NR); Managers (NR)	Qualitative	Normalisation Process Theory (NPT); stages of implementation (Fixsen et al.)	Observation of PIE action planning and review meetings; informant conversations and interviews with PIE team members; analysis of PIE observation, action-planning and review documentation; end-of-implementation interviews; contemporaneous events log	Aim was to describe and evaluate implementation of the PIE programme to improve person-centred care for people with dementia in acute hospital wards. Across 10 wards, PIE was fully implemented and sustained only in two, partially implemented in two and not implemented in six. Successful implementation was associated with staff perceiving PIE as relevant to their goals, collective team engagement in observation and action planning, alignment with trust dementia strategies, adequate staffing, time and physical space, strong clinical leadership and integrated facilitation from dementia specialists and ward managers, and relative organisational stability. Where demand pressures, staffing shortages, ward closures, competing initiatives or weak facilitation dominated, PIE activity stalled or never progressed beyond early exploration despite initial enthusiasm
The barriers and facilitators to implementing dementia education and training in health and social care services: a mixed-methods study	Surr CA; Parveen S; Smith SJ; Drury M; Sass C; Burden S; Oyebode J	2020	UK; mixed: acute hospitals; mental health trusts; care homes; primary care; mixed settings: acute hospital wards, mental health inpatient units, care homes, primary care services; DFHC extraction focuses on hospital and mental health inpatient data	n=436 audit respondents; n=576 trained staff survey respondents; n=75 Barriers and Facilitators Questionnaire respondents; n=153 interview and focus group staff (over 940 staff total, representing 386 training packages); Doctors (NR); Nurses (NR); AHPs (NR); Managers (NR)	Mixed methods	TDF; COM-B	online national audit survey of organisations and dementia training leads; online staff survey of trained staff; Barriers and Facilitators Questionnaire based on the TDF; individual interviews with dementia training leads, facilitators, managers and staff; focus groups with staff; descriptive quantitative analysis and template based thematic analysis	Aim was to use data from the What Works dementia training project to identify barriers and facilitators to implementing dementia education and training across UK health and social care using the TDF and COM-B; capability factors such as knowledge and skills were usually adequate and interactive, face to face and role relevant training built confidence to change practice; opportunity factors were the largest constraint, with lack of time, staffing, finance, dementia friendly ward environments and dedicated training space limiting behaviour change, while strong leadership, supportive ward or unit culture, peer support, mentorship and external support from families and agencies helped implementation; motivational factors such as skilled facilitation, mixed professional training groups, incentives and personal values around dementia care increased willingness to apply learning, whereas low morale, disinterest in dementia care and seeing training as irrelevant undermined change
Components of impactful dementia training for general hospital staff: a collective case study	Sass C; Surr CA; Burnley N; Drury M; Smith SJ; Parveen S; Burden S; Oyebode J	2018	UK; acute general hospital; Three NHS acute/general hospital Trusts in England, up to two general hospital wards per Trust with inpatients with dementia	n=49 staff participants across three sites; 6 wards observed with DCM; 24 patient/car er satisfaction cards; Doctors (NR); Nurses (NR); AHPs (NR); Managers (NR)	Mixed methods	Richards and DeVries Conceptual Model for Dynamic Evaluation of Learning Activities; Kirkpatrick four level training evaluation model	semi structured interviews; focus groups; observation of training sessions; Dementia Care Mapping ward observations; patient and carer satisfaction cards; document analysis of training materials; descriptive survey statistics	Aim was to identify which models of dementia education and training for general hospital staff are most likely to improve practice and care experiences and to understand barriers and facilitators to implementation across three positively deviant NHS acute Trusts. Training that was bespoke to the hospital, tailored to staff roles, delivered face to face in small interactive groups by skilled, enthusiastic facilitators, and that used experiential methods and the voices of people with dementia was perceived as relevant, engaging and confidence building, and staff reported concrete practice changes in communication, understanding behaviour, activity and family involvement. However, observational data showed wide variation in the extent to which person centred principles were enacted, with some wards demonstrating high levels of distress, low engagement and poor quality interactions, indicating that training alone did not guarantee consistent dementia friendly care. Key barriers included staffing shortages, lack of protected study time, difficulty engaging medics, untrained or negatively disposed agency staff, and non supportive physical environments, while key facilitators included proactive dementia training leads, supportive ward managers and senior leadership, organisational cultures valuing dementia care, and mechanisms such as mandatory training and champions to maintain focus and spread
Visual identifiers for people with dementia in hospitals: a qualitative study to unravel mechanisms of action for improving quality of care	Sutton E; Armstrong N; Locock L; Conroy S; Tarrant C	2023	England, UK; acute general hospital; Four acute care hospitals in England (two large, two smaller), mixed acute wards caring for inpatients with dementia	n=21 healthcare professionals; n=19 carers; n=2 people with dementia; 4 acute hospital trusts (case studies); Doctors (NR); Nurses (NR); AHPs (NR); Managers (NR)	Qualitative	Concept of classification and categorisation of patients; dark logic model of unintended consequence s; no explicit use of TDF, COM-B or CFIR	semi structured interviews; documentary review of dementia strategies and tools; narrative case studies across four hospitals	Using the classification lens, the study analysed staff experiences of visual identifiers for people with dementia in acute hospitals and unpacked mechanisms through which they can support or undermine dementia friendly care. Staff and dementia leads reported that identifiers can help coordinate care, signal eligibility for dementia specific initiatives, prioritise ward resources and cue dementia aware communication, but inconsistent use, lack of standardisation, weak coupling to personalised information, time pressure, staffing constraints and negative attitudes mean identifiers sometimes become mere labels that risk depersonalisation and unequal access to dementia support. Authors argue identifiers must be implemented with clear rules, accessible person specific information, adequate training and organisational support for dementia care
A qualitative focus group discussion study on the experiences of Certified Nurses in Dementia Nursing related to effective staff education	Taneichi H; Rokkaku R	2020	Japan; acute hospital; Ten acute general hospitals in central Japan, acute wards caring for inpatients with dementia	n=10 Certified Nurses in Dementia Nursing (DCNs); 10 acute hospitals; Doctors (0); Nurses (10); AHPs (0); Managers (0)	Qualitative	None/NR	focus group discussion with 10 DCNs; audio recorded, transcribed and thematically analysed using content analysis	Study explored how DCNs in Japanese acute hospitals educate staff in dementia care and what makes this education effective. Two overarching themes were identified: building a foundation for dementia care education (establishing systems with managers, understanding feelings of patients, families and staff, engaging volunteers and other professionals) and continuous support based on practical processes (on-the-job support, role modelling, jointly planning care, showing effects of dementia care and encouraging staff reflection). DCNs acted as mediators between managers and ward staff, provided concrete bedside support for challenging BPSD cases, mixed workshops with experiential learning and repeatedly questioned and guided nurses to reflect on their practice so they could eventually provide dementia care independently. Barriers included DCNs' limited authority and time, overworked nurses who saw dementia care as troublesome and tension between acute medical priorities and person-centred dementia care. Facilitators included managerial cooperation, multidisciplinary collaboration, visible positive effects of dementia care and using experiential learning cycles to support staff growth

Title	Author(s)	Year	Country and setting	Sample and professional groups	Study design	Theoretical framework	Data collection	Study aim and key findings
Deconstructing dementia and delirium hospital practice: using cultural historical activity theory to inform education approaches	Teodorczuk A; Mukaetova-Ladinska E; Corbett S; Welfare M	2015	UK (England); acute district general hospital; Acute non-psychiatric wards in a District General Hospital, with liaison old age psychiatry input, North East England	n=25 staff (15 individual staff interviews, 2 focus groups with 10 liaison mental health professionals); plus 2 carer focus groups and 1 patient focus group (not included in staff-only extraction); Doctors (5); Nurses (12); AHPs (6); Managers (2)	Qualitative	Social constructionism; Grounded theory (Charmaz); Cultural Historical Activity Theory (CHAT)	Semi-structured individual interviews; focus groups	Aim: to explore how practice gaps of hospital staff in relation to managing the confused older patient are constructed, and to use cultural historical activity theory to inform education approaches. Key findings: practice gaps in delirium and dementia care arise from interacting determinants at individual, team and organisational levels, including knowledge and skill deficits, personal philosophies and emotional avoidance, task based coping that prioritises medical tasks, time pressure and noisy ward environments, weak or inconsistent leadership, rigid professional and grade based role boundaries, medical dominance and fragmented physical and organisational separation of mental health and acute services. Socio cultural ward factors and power hierarchies mean that low status staff such as healthcare assistants and domestics often hold crucial knowledge about patients but feel unable to share it, while junior doctors experience pressure from nurses to sedate agitated patients despite awareness of risks. Applying cultural historical activity theory, the central object of the activity system is defined as learning about the patient, mediated by care relationships and ward processes, with rules and divisions of labour that often work against joining up patient knowledge. The authors argue that effective dementia friendly hospital practice requires structural change at individual, team and systems levels, including reducing ward moves, relaxing visiting, promoting horizontal sharing of patient knowledge and developing interprofessional education and knot working led by Liaison teams rather than relying solely on traditional training
The impact of an education intervention on the approach of hospital staff to patients with dementia in a Danish general hospital setting: An explanatory sequential mixed-methods study	Toubøl A; Moestrup L; Thomsen K; Ryg J; Hansen DL; Foldager M; Jakobsen S; Nielsen DS	2022	Denmark; acute, teaching, general hospital within university hospital; Smaller general hospital within Odense University Hospital, multiple departments including surgical, medical, para clinic and technical administrative services	n=849 staff at T0, n=618 at T1, n=468 at T2 (questionnaires); n=16 staff (qualitative interviews); Doctors (NR); Nurses (NR); AHPs (NR); Managers (NR)	Mixed methods	None/NR	Repeated self administered online questionnaires at three time points; semi structured individual interviews (mostly telephone, one face to face)	Aim was to investigate the impact of a dementia education intervention by examining self reported knowledge, attitude and competence of general hospital staff and exploring their experiences of these outcomes; mixed methods results indicated that whole workforce, interdisciplinary education produced modest but significant score increases and, more importantly, a shared knowledge base, inter occupational cooperation and shared proficiency that supported prioritising the person with dementia over tasks; remaining challenges concerned sustaining the dementia friendly focus and handling still challenging situations despite education
CogChamps: impact of a project to educate nurses about delirium and improve the quality of care for hospitalized patients with cognitive impairment	Travers C; Henderson A; Graham F; Beattie E	2018	Australia; acute, tertiary referral, teaching hospital; Two acute hospitals, six intervention wards (four medical, two surgical) and two control wards (one medical, one surgical), all general medical or surgical wards caring for older adults with cognitive impairment	n=34 RN Cognition Champions; n≈6 intervention wards with 30–40 nurses each (ward nursing workforce NR); n=181 patients observed (130 intervention hospital, 51 control hospital); Doctors (0); Nurses (34 CogChamps plus wider ward nurses NR); AHPs (0); Managers (NR)	Quantitative non-randomised	None/NR	Direct structured observations of nurse patient interactions on wards; chart and room audits using a purpose built Audit or Observational tool drawing on EHR documentation	Aim was to educate and empower experienced hospital nurses to lead ward level practice changes via CogChamps in order to improve delirium related assessment and care for hospitalised older patients with cognitive impairment; key findings were that collective social education of nurse champions plus ward specific Action Plans and facilitation increased nurses' use of cognitive assessments and influenced some medication practices, but had limited impact on broader non pharmacological care behaviours where tasks were diffuse, discretionary and not clearly owned, and that heavy workload, competing demands and variable leadership priority constrained sustained practice change
Care of patients with dementia in an acute trauma and orthopaedics unit	McCorkell G; Harkin D; McCrory V; Lafferty M; Coates V	2017	Northern Ireland; acute teaching hospital; acute trauma and orthopaedics unit	n=20 patient audit; Doctors (NR); Nurses (NR); AHPs (NR); Managers (NR)	Quantitative non-randomised	None	chart audit; action-research reflection; staff discussions	Study aimed to identify gaps in dementia care and collaborate with staff to implement a dementia toolkit. Key findings show initial low fidelity, staff stress, unclear behavioural expectations, competing priorities, and limited dementia-specific skills hindering implementation. Education, environmental cueing and champions improved adoption and consistency.
Caring for people with dementia in hospital: findings from a survey to identify barriers and facilitators to implementing best practice dementia care	Tropea J; LoGiudice D; Liew D; Roberts C; Brand C	2017	Australia; acute, teaching, regional; emergency departments and general medical wards in two Australian hospitals	n=112 staff; Doctors (29); Nurses (54); AHPs (17); Managers (NR)	Quantitative descriptive	KTAC	survey with Likert scales and open-ended items	study assessed staff knowledge, confidence, and perceived determinants of dementia care; key findings show major barriers (environment, staffing, time, knowledge) and system-level facilitators (specialist services, equipment, orientation cues). Results informed tailoring of a dementia care pathway.
The use of the truth and deception in dementia care amongst general hospital staff	Turner A; Eccles F; Keady J; Simpson J; Elvish R	2016	UK (England); acute, general hospital; Older people's wards in two general hospital NHS Trusts in north west England, caring for patients with dementia	n=12 general hospital staff across eight wards; Doctors (1); Nurses (7); AHPs (1); Managers (1)	Qualitative	Constructivist Grounded Theory; bioethics principles (beneficence, non maleficence, autonomy, justice)	Semi structured individual interviews; constructivist grounded theory analysis	Aim: to explore general hospital staff experiences and decision making when choosing whether to tell the truth or deceive patients with dementia. Key findings: staff identified three common triggers for considering deception (difficult questions about relatives and the past, managing behaviour or personal care, and sharing medical information). Decisions were shaped by mediating factors including poor communication and lack of guidance, non inclusion of many frontline staff in information sharing, tensions between job roles, responsibility and knowing the person, and reliance on personal versus professional ethical frameworks. Staff often felt paralysed, preferring distraction or passing responsibility to others rather than clearly telling the truth or lying. Qualified staff sometimes used deception pragmatically for medication administration or to reduce risk, justified through professional ethics and perceived best interests. Responses were adapted when relatives were present or when patients were highly distressed or agitated, further highlighting uncertainty and fear of complaints. Overall, dementia care communication around truth and deception in hospital was ad hoc, individually negotiated and weakly supported by policy, training or team discussion.

Title	Author(s)	Year	Country and setting	Sample and professional groups	Study design	Theoretical framework	Data collection	Study aim and key findings
Nurses' Perspectives and Experiences of Using a Bed-Exit Information System in an Acute Hospital Setting: Mixed Methods Study	Walzer S; Schön I; Pfeil J; Klemm S; Ziegler S; Schmoor C; Kunze C	2025	Germany; acute, teaching, university medical center; Three acute wards (neurosurgery, surgery, radiology) in a university medical center in Freiburg, caring for inpatients with cognitive impairment at risk of bed exits	Surveys: n=30 nurses at P0, n=24 nurses at P1, n=15 complete-case survey participants; Focus groups: n=11 nurses in 4 focus groups; Patients: 912 adults treated in P0, 1748 in P1, with cognitively impaired patients present on 199 of 256 days in P0 and 302 of 504 days in P1; Doctors (0); Nurses (30 survey P0; 24 survey P1; 15 survey complete cases; 11 focus group participants); AHPs (0); Managers (0)	Mixed methods	None/NR	Online questionnaires at P0 and P1; semi structured focus groups at P0 and P1; retrospective daily prevalence documentation of patients with cognitive impairment, bed leaving tendency, adverse events and BES use based on ward records.	Aim: to develop a better understanding of nurses' experiences, views and needs regarding a bed exit information system in an acute hospital setting and to explore its impact on strain and adverse events when caring for patients with cognitive impairment. Key findings: nurses reported high emotional and practical strain when caring for cognitively impaired inpatients, especially around unsupervised bed leaving, falls and device disconnections. They generally held positive attitudes toward IT and welcomed BES as an additional tool, particularly at night and when sitters were unavailable, but experienced a gap between high initial expectations and more modest benefits in practice. Qualitative data showed that deciding who should receive a mat, and in which situations, was complex because of heterogeneous symptoms and ward conditions; nurses developed informal criteria, prioritising cognitively impaired, mobile and lively patients at risk of wandering. BES was perceived to improve safety and feelings of security in specific scenarios by allowing faster response to bed exits, yet frequent alarms, undifferentiated signals through the call system and competing demands contributed to information overload and alarm fatigue, occasionally leading to removal of the system. Quantitatively, overall adverse event rates per cognitively impaired patient day decreased between P0 and P1, but effects differed across wards, and perceived strain remained high. Overall, BES was integrated pragmatically as one support within a wider bundle of strategies rather than a comprehensive solution, with its effectiveness shaped by staffing levels, ward layout, alarm design and nurses' expectations.
Junior doctor dementia champions in a district general hospital (innovative practice)	Wilkinson I; Coates A; Merrick S; Lee C	2016	UK (England); acute, district general hospital; Trust A: district general hospital in South London, various wards including elderly care and hip fracture; Trust B: two district general hospitals in south west London, junior doctors across wards caring for older inpatients with dementia or cognitive impairment	n=6 junior doctor dementia champions in focus group (Trust A); n=20 junior doctors responding to questionnaire (Trust B); Doctors (26); Nurses (0); AHPs (0); Managers (0)	Mixed methods	None/NR	Focus group with six FY1 dementia champions; electronic questionnaire with multiple choice and open questions sent to all junior doctors in neighbouring Trust B; qualitative interpretive phenomenological analysis of focus group and open questionnaire responses; basic descriptive analysis of questionnaire responses.	Aim: to explore what influenced junior doctors to become dementia champions and what impact being a champion had on them within district general hospitals implementing dementia CQUIN requirements. Key findings: three main drivers for volunteering were prior personal experiences with dementia, peer involvement and snowballing interest, and perceived knowledge gaps about assessing and managing cognitive impairment in acute settings. Champions described gaining practical skills in structured cognitive assessment, collateral history taking and communication with relatives, and felt more confident and holistic in their approach to older patients with dementia. They moved from seeing themselves mainly in a secretarial role to recognising a more active role in shaping care standards and directly supporting patients and families, supported by a motivated senior consultant who legitimised their leadership. Questionnaire data from a different trust showed many junior doctors felt dementia care for inpatients was worse than for other conditions and better on specialist units, and reported barriers to more hands on dementia care such as being too busy, limited team support and assumptions that some tasks were nursing roles. Overall, the dementia champion model functioned as a threshold experience for many participants, changing how they understood their professional role in dementia care and acting as a vehicle for leadership and career development while helping embed dementia friendly hospital practices.
Nurses' Experiences in Caring for Older Adults With Responsive Behaviors of Dementia in Acute Care	Yous ML; Ploeg J; Kaasalainen S; Schindel Martin L	2019	Canada; acute, teaching hospital; Four acute medical units three clinical teaching units and one cardiology unit in an urban teaching hospital in Southern Ontario	n=15 staff 10 nurses 5 allied health professionals; Doctors (0); Nurses (10); AHPs (5); Managers (0)	Qualitative	None/NR	Individual semistructured interviews with nurses and allied health professionals plus reflective field notes analysed using experiential thematic analysis	Aim was to explore how nurses in Canadian acute medical units experience caring for older adults with dementia who show responsive behaviours and to gather their recommendations to improve dementia care. Care delivery was described as complex emotionally taxing and ethically challenging, particularly when invasive procedures escalated behaviours or when nurses felt unsupported after physical aggression. Nurses often relied on pharmacological strategies such as PRN antipsychotics in desperate or proactive ways because of limited time resources and competing acute priorities, while using low investment person centred approaches such as music familiar television and simple activities when possible. Major barriers included time pressure focus on acute illness and discharge throughput, inadequate staffing and one to one support, understimulating ward environments and limited ongoing dementia specific education. Facilitators included shared care and strong interprofessional collaboration, involvement of specialty teams such as BSO, family input, continuity of care with familiar nurses, and structured behavioural rounds and team meetings to coordinate plans. Nurses recommended more trained staff and PSWs, increased BSO presence, regular refresher education and in services, more inclusive family and team meetings, and creation of a safe dementia focused acute unit.
Assessing the Attitudes of Greek Nurses Toward Computerized Dementia Screening	Stelios Zygouris; Mara Gkioka; Despina Moraitou; Birgit Teichmann; Thrasyvoulos Tsiatsos; Sotirios Papagiannopoulos; Magda Tsolaki	2020	Greece; acute general, public; Two urban public general hospitals in Thessaloniki, inpatient wards, nurses attending a dementia training program	n=161 nurses from two public general hospitals; Doctors (0); Nurses (161); AHPs (0); Managers (0)	Quantitative descriptive	NR	Self administered questionnaire survey about attitudes and perceived barriers, with confirmatory factor analysis of Likert scale items and descriptive frequency analysis of barrier items	Aim: To validate the factor structure of a questionnaire assessing nurses' attitudes toward computerized dementia screening, describe attitudes of Greek nurses, and identify perceived barriers to future implementation in hospitals. Confirmatory factor analysis in 161 nurses supported a two factor structure distinguishing feasibility and acceptability. Overall attitudes toward computerized dementia screening were positive with high feasibility and acceptability scores, and nurses reported they could devote 1–2 days to training and 10–20 minutes per older adult during shifts. Main perceived barriers to implementing computerized dementia screening in Greek public hospitals were cost of equipment, insufficient training and ICT skills, lack of a concrete plan to integrate computerized screening into daily hospital routines, and time needed for staff training.

Guide to the implementation-detail columns	
**Study-focus classification / DFHC intervention (TIDieR elements):**	Identifies whether the study is I or R. For I studies, describes the distinct DFHC initiative and how it was delivered. For R studies, describes the routine-care or pre-implementation focus and makes clear that no initiative was delivered.
**Implementation strategy (Proctor et al.):**	Describes strategies that were implemented, planned or recommended and clearly distinguishes between these.
**AACTT:**	Action: the behaviour being promoted; Actor: who performs it; Context: where it occurs; Target: to whom or what it is directed; Time: when, how often or for how long.
**Findings related to delivering or implementing DFHC:**	Summarises findings relevant to delivering DFHC in routine care or implementing a specific DFHC initiative.

I = intervention/tool/strategy-specific study; R = routine/current-care study. Thirty-four studies were classified as R and 31 as I.

Implementation details

Title	Study-focus classification / DFHC intervention (TIDieR elements)	Implementation strategy (Proctor et al.)	AACTT	Findings related to delivering or implementing DFHC
Facilitators and barriers to implementing a specialized care unit for persons with cognitive impairment in an acute geriatric hospital: a process evaluation	I — Interprofessional multicomponent specialized unit for persons with cognitive impairment, 24 beds, built within an acute geriatric hospital; includes an interprofessional care pathway, adapted interdisciplinary assessment and meetings, existing multicomponent delirium management, nursing process oriented to people with dementia, individualized daily structure and mealtimes, coordinated therapy, involvement of relatives, volunteers providing one to one support when needed, and environmental adaptations such as providing meaningful materials on the ward; delivered by geriatric physicians, nurses, therapists and social workers on the specialized unit during admission, with content iteratively refined during action research cycles; several intended elements, such as some trainings and meeting changes, remained only partially implemented by the end of the project	Implementation via a participatory action research project with an interprofessional project group (nursing scientist, geriatricians, unit manager, nurses, therapists, social worker, study nurse) acting as change agents; repeated action research cycles with workshops used to co design interventions, plan, observe and reflect; education and training on validation, basal stimulation and aggression management; planned but partly unrealized enhancements to nurse staffing; attempts to modify communication structures and interprofessional meetings; ongoing but mainly information focused contact with hospital management; cooperation with an external research partner; implementation duration extended from 8 to about 18 months due to contextual barriers	Action=implement and integrate the multicomponent specialized unit model and associated dementia friendly care processes into routine acute geriatric practice; Actor=Doctors, Nurses, AHPs, Managers working on and around the specialized unit; Context=acute geriatric hospital specialized 24 bed unit and other geriatric wards in German speaking Switzerland; Target=care and treatment of hospitalised older adults with cognitive impairment and the work processes of the interprofessional team; Time=during the 18 month action phase and as part of everyday work on each shift and admission	Implementation outcomes were mixed; some sets of interventions, such as delirium management and parts of the nursing process, were implemented, while others like interprofessional meeting changes, extended therapy services and some trainings remained partial or stalled; acceptability and perceived appropriateness varied, with motivation for change particularly high among nurses but scepticism about the specialized unit, triage criteria and workload among some staff; feasibility was constrained by COVID related disruptions, staff turnover, staffing shortages, rigid efficiency oriented processes and architectural constraints; fidelity to the intended individualized, person centred model was limited by economic billing requirements and entrenched workflows; long term sustainability was uncertain due to ongoing resource pressures, fragmented interprofessional ownership and only partial management engagement
Person-Centred Dementia Care in an Acute Hospital: Experiences from Nurses Working in a Specialized Dementia Unit	I — CAMIE specialized person centred dementia unit, delivered mainly by geriatric nurses within a multidisciplinary team, using enhanced delirium and psychosocial care protocols, Know me better bedside boards, music and activity resources, a designated social nurse role and restraint free approaches, provided face to face on the unit throughout admission, tailored to each patients biography, language and behavioural triggers, with content refined over time through staff learning and reflective sessions, fidelity supported through mandatory PCC workshop and ongoing supervision but not formally measured	Mandatory experiential PCC workshop with simulation of having dementia, in house and informal dementia teaching and role modelling by experienced CAMIE nurses, monthly sharing sessions for reflection and problem solving, enhanced nurse to patient ratios and a dedicated social nurse during day shifts, supportive geriatric leadership and involved doctors, team based peer support with social outings, and embedding delirium and psychosocial care protocols into everyday workflows	Action=deliver person centred, restraint minimising dementia care using PCC strategies and individualized engagement; Actor=Nurses; Context=CAMIE specialized dementia unit, tertiary acute hospital, Singapore; Target=patients with dementia with or without delirium and acute medical problems; Time=throughout each shift and across the hospital admission	Acceptability high as nurses valued PCC, meaningful relationships and support; adoption and fidelity to restraint free, needs based care increased over time as knowledge, skills and confidence grew; appropriateness perceived as high for acute dementia care; feasibility initially limited by lack of training, emotional burden, safety fears and language barriers but improved with training, staffing, teamwork and leadership; sustainability threatened by stigma from other wards, recruitment difficulties and fatigue despite strong internal culture
Prevention and management of behavioural and psychological symptoms in patients with dementia in acute care: a best practice implementation project	I — Nonpharmacological BPSD program: CMAI risk assessment; trigger identification; individualized care plans; routine EHR documentation; basket of interventions (spatial orientation; door security; music therapy; aromatherapy with essential oil diffusers; low-stimulation lounge; room personalization); provider=nursing team with head nurse/CNS support and geriatric psychologist input; mode=bedside care + team meetings + EHR entries; dose=on admission and throughout stay (per shift); tailoring=by risk/unmet needs; modifications=added aromatherapy; refurbished lounge; fidelity=planned audits + attendance tracking; actual=fidelity variable (documentation lag; low care-plan uptake)	Training (4×45 min by geriatric psychologist); audit & feedback (JBI PACES/GRiP); leadership support (Head Nurse, CNS, Nurse Manager); standardized protocols/workflow; adverse-event reporting for aggression; interdisciplinary meetings (nurses, physicians, psychologist); coaching/mentoring; environment redesign (low-stimulation lounge); EHR documentation process (attempted)	Action=systematic BPSD risk assessment, trigger identification, nonpharmacological management, and documentation via care plans; Actor=Nurses; Managers; AHPs; Context=acute geriatric care unit, tertiary teaching hospital, Switzerland; Target=patients with dementia at risk/of BPSD; Time=at admission and daily per shift; 4-month implementation window	Adoption/penetration: processes in place 100%; training reach 79%; identification of triggers and risk assessment ↑ to 46%. Fidelity/feasibility: individualized care plan development/implementation 21% (documentation barriers). Acceptability/feasibility: nursing mental workload; EHR constraints; training timing. Appropriateness/penetration: strengthened interprofessional collaboration; routine adverse-event reporting. Sustainability: plan for biannual training + mentorship; further audit planned but not completed.
Understanding and identifying ways to improve hospital-based cancer care and treatment for people with dementia: an ethnographic study	R — No distinct DFHC intervention delivered. Routine-care focus: Identify and clearly document dementia and tailor oncology workflows (appointment timing; communication).	No formal implementation strategy reported..	Action=identify and clearly document dementia and tailor oncology workflows (appointment timing; communication); Actor=Doctors; Nurses; AHPs; Context=outpatient oncology clinics in two NHS Trusts, North of England (regional cancer centre and local unit); Target=oncology records; workflows; staff practices; Time=at initial assessment and ongoing at each visit	Feasibility constrained by time/resource pressures and rigid scheduling; fidelity/penetration of dementia flagging/documentation low/variable; adoption of dementia-specific training limited; appropriateness of flexible appointments and tailored communication identified
Implementing an intervention to enhance care delivery and consistency for people with hip fracture and cognitive impairment in acute hospital wards: a mixed methods process evaluation of a randomised controlled feasibility trial (PERFECTED)	I — PERFECT-ER multicomponent ward-level checklist intervention: name=PERFECT-ER; provider=SIL (internal implementation lead) with PL (senior doctor); materials=68-item clinical/functional/psychosocial/cognitive checklist + 15 organisational items; procedures=PDSA cycles, action plans, documentation tools; mode=on-ward audits/spot checks, brief education, meetings/briefings; dose=average ~68% elements delivered (varied by ward); tailoring=aligned to existing pathways and ward processes; modifications=team action-planning meetings often replaced by targeted discussions with opinion leaders; fidelity=variable across wards (stronger where resources/engagement higher)	Internal facilitation (SIL) with opinion leaders (ward managers; orthogeriatricians as PLs); 3-day SIL training; audit & feedback via checklist scoring; reminders/“nagging”; local champions; workflow/documentation standardisation; networking/communications (emails, presentations, noticeboards); alignment with existing packs/policies	Action=implement and standardise checklist-based dementia-friendly practices in hip fracture care; Actor=Managers; Doctors; Nurses; AHPs; Context=acute hospital wards, NHS, England and Scotland; Target=ward staff practices/processes and care delivery; Time=7 PDSA cycles over 18 months	Acceptability (tools valued by staff); Adoption (elements implemented on all wards); Appropriateness (aligned with existing pathways); Feasibility (fragile under high pressures/limited SIL time); Fidelity (varied); Penetration (spread within wards though not all staff engaged); Sustainability (ethos change on some wards; limited where pressures high)
Implementation of STAR-VA for behavioral symptoms of dementia in acute care: Lessons learned	I — STAR-VA (interdisciplinary behavioral program for BPSD): provider=NCs (nurses) + Behavioral Coordinator (psychologist) with unit staff; materials=STAR-VA manuals; brief handouts; behavior plan templates; procedures=identify BPSD triggers; create individualized behavior plans; pleasant activities; communication skills; mode=in vivo coaching; huddles; brief in-services; dose=NC training (8h dementia course + 5-week STAR-VA modules); ongoing rounding and consults; tailoring=adapted for acute care (multiple NCs per shift; rapid documentation tool; electronic communication); modifications=education format, communication channels; fidelity=NC training completion; behavior plans uploaded to EHR and bedside copies to support consistent use	Training + education (8h dementia course; 5-week STAR-VA modules); nurse champions per shift; protected time; embedded psychologist (BC) one day/week; in vivo coaching; daily huddle education rounds; brief in-services; printed materials/behavior plan forms; weekly check-ins; electronic consult/feedback; leadership buy-in and shared governance (Pathways to Excellence); rapid identification workflow	Action=create and implement individualized behavior plans for BPSD; Actor=Nurses (NCs) with AHPs (psychologist) and Managers; Context=acute medical telemetry unit, VA Boston, USA; Target=patients with dementia exhibiting BPSD; Time=per shift and within 2–8 days of admission; 7-month implementation window	Acceptability (manager and staff buy-in); adoption (8 plans for 7 veterans; 83% staff reached by education); feasibility (rapid identification; plans created quickly; workflows functioned across shifts); fidelity (NCs completed training; plans uploaded to EHR; bedside copies used); penetration (multiple NCs to ensure 24/7 coverage); sustainability (ongoing rounds and bi-weekly meetings)

Title	Study-focus classification / DFHC intervention (TIDieR elements)	Implementation strategy (Proctor et al.)	AACTT	Findings related to delivering or implementing DFHC
Dementia case-finding in hospitals: a qualitative study exploring the views of healthcare professionals in English primary care and secondary care	I — Dementia case-finding policy/process: provider=junior doctors; ward nurses; dementia specialist nurses; coordinators; materials=cognitive tests (AMTS; MMSE; MoCA); IT forms/mandatory fields; procedures=single admission assessment (some repeat pre-discharge); documentation to discharge summary; mode=embedded in admission; dedicated chaser staff; dose=per unplanned admission ≥75; tailoring=local hospital methods/tools; modifications=evolving IT templates; fidelity=variable completion; missing fields and non-compliance in some specialties	Top-down policy with financial incentives (CQUIN); local workflow design; dedicated staff to ensure completion; IT prompts/mandatory fields; awareness/training initiatives; ad hoc liaison psychiatry referral pathways; variable documentation standards and discharge summary processes	Action=perform cognitive case-finding and document/communicate results; Actor=Doctors; Nurses; Managers; Context=acute NHS hospitals, East of England; Target=older inpatients (≥75) with unplanned admissions; Time=on admission (sometimes repeated before discharge)	Acceptability (mixed across specialties); adoption (heterogeneous across hospitals/specialties); feasibility (hampered by IT limitations/staffing); fidelity (variable; missing mandatory fields); appropriateness (delirium vs dementia concerns; risk of mislabelling); penetration (implemented across 12 hospitals); sustainability (unclear; depends on incentives/IT)
An Action Research Approach to Implementing a Program for Sharing Meaningful Moments With Hospitalized Patients Living With Dementia	I — Meaningful Moments initiative (adapted key elements of DementiAbility): providers=therapeutic recreationists; nurses; interprofessional team; materials=laminated bedside "Getting to know me…" poster with attached "Meaningful Moments to share together" card; activity kits; procedures=gather personhood info; translate to individualized moments; share during care/rounding; document in EMR; mode=two-day workshops; bedside tools; iterative in vivo practice; dose=core staff trained; unit-wide rollout over ~2 years; tailoring=selected hospital-suitable elements (not full DementiAbility); modifications=added EMR dropdown for "engagement in a meaningful moment"; fidelity=100% beds with posters; documentation compliance low	Champions ("shining stars"); leadership with clear roles/accountability; persuasive education; audit & feedback; reminders/motivational messaging; implementation blueprint; integration into routine care; activity kits; progress reporting	Action=create, share, and document individualized meaningful moments; Actor=Nurses; AHPs; Managers; Context=specialized dementia care unit, post-acute hospital, Canada; Target=patients living with dementia; Time=per shift and ongoing over ~2 years	Acceptability (high staff support; 100% awareness of posters); adoption (posters at every bedside; moments shared); feasibility (embedded during personal care/rounding); fidelity (posters high; documentation low); penetration (unit-wide); sustainability (maintained >2 years with leadership/audit)
Disjunctures in practice: ethnographic observations of orthopaedic ward practices in the care of older adults with hip fracture and presumed cognitive impairment	R — No distinct DFHC intervention delivered. Routine-care focus: Manage interruptions and deliver routine care for hip-fracture patients with cognitive impairment.	No formal implementation strategy reported..	Action=manage interruptions and deliver routine care for hip-fracture patients with cognitive impairment; Actor=Nurses; Doctors; AHPs; Managers; Context=acute ortho-geriatric wards and EDs, NHS teaching hospitals, UK; Target=older adults with hip fracture and presumed cognitive impairment; Time=throughout 24-hour cycle during routine care	Appropriateness concerns about "fast-track"; feasibility constrained by understaffing and workload; fidelity gaps to hourly/2-hourly care rounds; adoption challenges and workflow friction with hybrid paper–ePrescribing; penetration/sustainability NR
Patient safety for people experiencing advanced dementia in hospital: A video reflexive ethnography	I — Unit-wide person-centred DFHC model; provider=multidisciplinary team; materials=training resources, VRE clips, environmental adaptations (carpeted quieter space); procedures=psychosocial/non-pharmacological care, individualized activities, de-escalation, daily huddles/case conferences, nurse-led code black; mode=face-to-face care, team meetings, video-based reflexive sessions; dose=5-day initial education for all staff plus periodic sessions; tailoring=to patient needs and staff roles; modifications=ongoing situated adaptations; fidelity=reinforced via reflexive sessions and leadership oversight	Training all staff; champions/role modelling by leaders; audit/feedback via VRE; workflow/policy changes (nurse-led code black; security training); interprofessional huddles/case conferences; reminders via routine reflection; inclusive education for clinical and non-clinical staff	Action=situated adaptation of dementia-safe practice with reflection and team decisions; Actor=Nurses; Doctors; AHPs; Managers; Context=GEM ward in an acute hospital, South Australia; Target=patients with advanced dementia; Time=daily per shift; regular huddles/case conferences; periodic reflexive sessions over 18 months	Acceptability (positive staff engagement; joy/meaning in work); Adoption (reflexive sessions became routine; nurse-led code black embedded); Appropriateness (fits advanced dementia needs); Feasibility (unit-wide training incl. non-clinical staff); Fidelity (shared ethos reinforced by leadership and VRE); Sustainability (transparent teamwork culture maintained)
Dementia, medication and transitions of care	R — No distinct DFHC intervention delivered. Routine-care focus: Medication reconciliation and structured discharge communication with electronic updating. Routine practices examined included: Content relates to medication reconciliation, discharge processes, DAAs, cognitive screening/identifiers, use of patients' own meds, pharmacist-led reviews, and eHealth	No formal strategy implemented; authors recommended: increase pharmacist involvement (transition coordinators; community liaison pharmacists); structured discharge communication; standardized transfer ("yellow/green bag"); nurse/OT roles in discharge support; cognitive identifiers/screening; self-administration/patients' own meds; IT linkage and mandatory e-record updates; staff training.	Action=medication reconciliation and structured discharge communication with electronic updating; Actor=AHPs (pharmacists; occupational therapists); Nurses; Doctors; Context=acute hospital wards/ED with transitions to community/RACFs; Target=PWD medication lists, discharge summaries, continuity of medicines; Time=on admission (within 24h), at discharge, and within days post-discharge	Medication reconciliation and discharge communication were hindered by fragmented IT systems, unclear responsibilities, short stays and inconsistent pharmacy involvement; structured transfer information, pharmacist support and targeted training were facilitators.
Using sense-making theory to aid understanding of the recognition, assessment and management of pain in patients with dementia in acute hospital settings	R — No distinct DFHC intervention delivered. Routine-care focus: Recognize and manage pain via sense-making and pattern-building and trial-and-error. Additional study focus: Study proposes a revised conceptual model: recognition–assessment–management as distributed, sense-making (Recognition-Primed Decision) process rather than linear sequence	No formal implementation strategy reported. Organisational elements discussed: ward routines and comfort rounds; surgical vs medical ward expectations; shared narratives/pattern-building; documentation practices; limited use of validated pain tools; trial-and-error titration of analgesia.	Action=recognize and manage pain via sense-making and pattern-building and trial-and-error; Actor=Nurses; HCAs; Doctors; Physiotherapists; Specialist nurses; Managers; Context=acute NHS wards (medicine, surgery, rehab, continuing care); Target=older inpatients with dementia (accurate pain detection and appropriate analgesia/management); Time=throughout shifts and over admission trajectory (distributed across staff/handovers)	Pain recognition and management depended on contextual sense-making, shared narratives and cumulative cues; staffing and ward routines constrained assessment, and tools alone did not reliably prompt action.
Exploring dementia care in acute care settings: Perspectives of nurses and social workers on caring for patients with behavioral and psychological symptoms	R — No distinct DFHC intervention delivered. Routine practices examined included person-centred, non-pharmacological approaches for BPSD; brief=name/brief: person-centered, non-pharmacological tactics (redirection, de-escalation, music/stretching groups, sitter/1:1, private room); provider=nurses, social workers; materials=room adjustments, music, group activities, family-provided information/photos; procedures=history-taking, family coordination, redirection, sitters, consults; mode=in-person on ward; dose=as needed per shift; tailoring=by patient story and triggers; modifications=NR; fidelity (planned/actual)=NR	No formal implementation strategy reported. Informal supports described: Training/education informally via team communication; family engagement; team-based coordination; consults (e.g., music/pet therapy); workflow adjustments (private rooms, 1:1 sitters); reminders via team handoffs; policy/structural supports limited by resources.	Action=manage BPSD using primarily non-pharmacological strategies with escalation to medication when needed; Actor=Nurses; AHPs; Context=ED and inpatient units, academic tertiary acute hospital, USA; Target=patients with dementia exhibiting BPSD; Time=throughout hospitalization and per shift	Acceptability=person-centered, team-based strategies viewed positively; Appropriateness=non-pharmacological strategies appropriate for BPSD; Feasibility=limited by time, staffing, space, and resources; Adoption=variable (some nurses default to medications); Fidelity=NR; Penetration=NR; Sustainability=NR
Using Signs and Symbols to Label Hospital Patients with a Dementia Diagnosis: Help or Hindrance to Care?	I — "Technologies of attention" for dementia: bedside/board symbols (blue flower, butterfly, dragonfly); red trays for mealtime support; "This is me" profile form; dementia-friendly noticeboards and labeled bays; provider=ward staff; materials=laminated signs, magnets, whiteboards/digital boards, red trays, "This is me" forms; procedures=apply symbols, complete/file profiles, use colored crockery; mode=visual signage/documentation at bedside/boards; dose=continuous during admission; tailoring=rare; modifications=ad-hoc; fidelity=often inaccurate or not used	Whole-hospital labeling schemes; environmental cues (boards, trays, crockery); administrative "dementia-friendly" designation; routine handovers referencing symbols; minimal systematic training or audit; poor maintenance/ownership of signage	Action=use signage and one-page profiles to signal dementia status and needs; Actor=Nurses; Doctors; AHPs; Managers; Context=acute wards (MAU, Trauma & Orthopaedics) in teaching hospitals, UK; Target=patients with dementia; Time=throughout admission and per shift	Acceptability=institutionally promoted; Appropriateness=questioned due to stereotyping; Feasibility=high to deploy but low to maintain accurately; Fidelity=frequently poor (mislabeling, nonuse); Adoption=widespread signage, variable staff use; Penetration=whole-ward/bay labeling; Sustainability=maintained as administrative status rather than practice change
Developing a Life Story Intervention for Older Adults With Dementia or at Risk of Delirium Who Were Hospitalized: Multistage, Stakeholder-Engaged Co-Design Study	R — Pre-implementation co-design; pilot pending. Proposed intervention: MemoryWell life story adapted for acute care; MemoryWell life story (Short-Form bullets + 400–500-word Narrative); provider=trained interviewers/writers (external) with clinical team readers; materials=interview guide, audio/transcripts, EHR wireframes (Daily Rounds; Social History); procedures=30–45 min interview with patient and/or care partner, writer drafts, editor reviews, story shared to team; mode=in-person or virtual; dose=one per admission; tailoring=topics/time per capacity and workflow; modifications=co-designed content and EHR placement; fidelity (planned/actual)=NR (pilot pending)	Planned implementation strategies: co-design with the interdisciplinary team; planned team training and awareness; EHR integration using a Daily Rounds quick-view and Social History full text; identification of champions; workflow alignment to rounds; privacy and consent procedures; usability testing via survey.	Intended action=review and use Life Story Short-Form during rounds to tailor care; intended actors=Doctors; Nurses; AHPs; Managers; intended context=ACE unit, teaching hospital, USA; intended target=older inpatients with dementia or at risk of delirium; intended time=daily rounds and throughout admission	Anticipated acceptability was high among the interdisciplinary team; anticipated appropriateness for person-centred care was high; perceived feasibility was limited by time and short hospital stays; adoption reflected intention to use the intervention; fidelity, penetration and sustainability were not reported because the pilot was pending.
Issues experienced while administering care to patients with dementia in acute care hospitals: A study based on focus group interviews	R — No distinct DFHC intervention delivered. Routine-care focus: Early detection and management of problematic behaviors and falls. Study details: provider=NR, materials=NR, procedures=NR, mode=focus groups about practice, dose=1-1.5 h sessions, tailoring=NR, modifications=NR, fidelity=NR	No formal implementation strategy reported. Existing practices described: sensor mats and bringing patients to the station but no explicit strategy package.	Action=early detection and management of problematic behaviors and falls; Actor=Nurses; Context=medical and surgical wards, acute hospitals, Japan; Target=patients with dementia in acute care; Time=per shift and during admission	Feasibility, prevention and close observation difficult under staff and time constraints; Fidelity, fall prevention protocols not always adhered; Appropriateness, ethical concerns about restraints and intensive treatments; Adoption, limited interprofessional response and psychiatric consult uptake

Title	Study-focus classification / DFHC intervention (TIDieR elements)	Implementation strategy (Proctor et al.)	AACTT	Findings related to delivering or implementing DFHC
Effects of a Person Centered Dementia Training Program in Greek Hospital Staff: Implementation and Evaluation	I — two day classroom training, six interactive modules over roughly nine hours; provider: MG; materials: videos, scenarios, PCC tools including getting to know me card and screening tools for dementia, delirium, pain; procedures: interdepartmental cohorts of 13 to 20 staff, practice based activities; mode: face to face; dose: 14 workshops; tailoring: based on prior needs analysis and local context; modifications: NR; fidelity: pre, post, three month assessments and planned train the trainer	leadership engagement meetings with nurse directors and ward leads; interdepartmental scheduling; skills training with PCC emphasis; plan for champions and train the trainer; action plan to repeat twice yearly; informal audit via follow up questions on use in practice	Action=apply PCC communication and use dementia, delirium, pain tools in routine care; Actor=Nurses; AHPs; Context=acute wards in two Greek hospitals; Target=care processes and documentation for patients with dementia; Time=each shift and over three months	acceptability high: 85 percent rated training excellent; adoption: 50 percent reported using communication techniques, 22 percent PCC approaches, 8.8 percent getting to know me card, 14 percent screening tools; appropriateness: content and materials highly rated; feasibility: time and staffing constraints noted; fidelity: knowledge and confidence gains sustained with slight decline at three months; sustainability: train the trainer and leadership support planned
The Person, Interactions and Environment Programme to improve care of people with dementia in hospital: a multisite study	I — PIE: observation tool plus cyclical service improvement process: observe, reflect, action plan, act, review; providers: ward implementation team including dementia nurse specialist, practice development facilitator, ward leadership; materials: manual, tools, flow charts; procedures: paired observations, team meetings, action plans; mode: on ward workshops and meetings; dose: repeated cycles over 18 to 20 months in implementer wards; tailoring: local goals and actions; modifications: emergent, action plans amended or abandoned as needed; fidelity: variable, only two wards reached full adoption and sustainability	Interactive training workshop, manual, identification of drivers and facilitators, formation of implementation team, observation feedback, action planning with visible reminders, leadership engagement, alignment with trust dementia strategy, champions, periodic review and audit-style reflection	Action=implement cyclical PIE practice change; Actor=Nurses, AHPs, Managers; Context=acute wards in NHS hospitals, England; Target=ward practices for people with dementia; Time=cycles over 18 to 20 months.	Adoption, feasibility, fidelity, sustainability, acceptability: PIE fully adopted in 2 of 10 wards, partial or non adoption elsewhere; limited penetration beyond teams where drivers and facilitators were present; clinical outcomes collection incomplete
How do emergency department staff respond to behaviour that challenges displayed by people living with dementia? A mixed-methods study	R — No distinct DFHC intervention delivered. Routine-care focus: Prevent and de escalate behaviour that challenges via one to one care, rapid assessment, distraction, quieter area. Routine practices examined included: staff recalled ED dementia-friendly initiatives: music; box of distractions; reminders of the past; providing food and drink; This is Me; individual cubicles; family presence, with ED-specific limitations noted in Table 3	No formal strategy implemented; authors recommended: Embedding ED-specific training; checklist or escalation toolkit; stakeholder buy in; face to face study days; share This is Me electronically; workflow tweaks for quicker assessment.	Action=prevent and de escalate behaviour that challenges via one to one care, rapid assessment, distraction, quieter area; Actor=Doctors, Nurses; Context=EDs in three NHS Hospital Trusts, Southwest England; Target=patients with dementia with behaviour that challenges; Time=per shift including nights	Acceptability: strong support for ED-specific tool and separate area; Feasibility: constrained by staffing, space, time, ambulance delays; Appropriateness: prevention focus preferred; Fidelity: inconsistent responses and improvisation; Adoption: proposed only; Penetration: NR; Sustainability: NR
Balancing the needs of individuals and services in cancer treatment for people with dementia: A focused ethnographic study	R — No distinct DFHC intervention delivered. Routine-care focus: Deliver person centred oncology care with dementia identification, documentation, and continuity.	No formal implementation strategy reported.	Action=deliver person centred oncology care with dementia identification, documentation, and continuity; Actor=Doctors; Nurses; AHPs; Context=oncology and radiotherapy outpatient departments, two NHS trusts, UK; Target=patients with dementia receiving cancer diagnosis and treatment, workflows; Time=per appointment and across treatment pathway	Person-centred adaptations were acceptable and appropriate but constrained by time, staffing and target-driven workflows; proactive planning, flexible appointments, continuity and tailored communication facilitated care.
Supporting general hospital staff to provide dementia sensitive care: A realist evaluation	I — Dedicated dementia ward or mobile one-to-one team; providers: nurses, HCAs, dementia specialist nurses, clinical experts; materials: policies, care plans, biographical tools such as This is me, dementia-sensitive assessment tools such as Abbey Pain Scale; procedures: person-centred assessment, engagement activities, risk monitoring, documentation; mode: face-to-face at bedside and ward routines; dose: during shifts as needed; tailoring: to patient preferences, abilities, unmet needs; modifications: local ward workflows; fidelity: audited variably, documentation inconsistent for verbal agitation.	Training by clinical experts, audit and feedback on tools and care plans, defined roles for dementia care, senior staff endorsement, peer learning, supervision and proximity of experts, workforce allocation for one-to-one monitoring, policy standardisation.	Action=adapt and prioritise person-centred care to address unmet needs and manage risk; Actor=Nurses; AHPs; Doctors; Managers; Context=inpatient wards including a dedicated dementia ward and general medical wards in NHS acute hospitals, UK; Target=patients with dementia; Time=throughout each shift and at key care interactions.	Acceptability: mixed, influenced by valuing dementia care; Adoption: variable across wards; Appropriateness: endorsed by policies and experts; Feasibility: constrained by ward pressures and staffing; Fidelity: inconsistent documentation and practice; Penetration: stronger where experts had close contact; Sustainability: threatened by staff retention concerns and perceived deskilling.
Exploring ways to enhance pain management for older people with dementia in acute care settings using a Participatory Action Research approach	I — PAR facilitation plus local tools and prompts, providers: ward nurses, nursing assistants, pharmacists, AHPs, junior doctors, ward managers; materials: Purple Folder Toolkit, visual prompts such as purple dot, family or carer communication tool, pain assessment tools; procedures: early identification at triage, routine pain assessment including behavioral tools, family input, notes review, medication review; mode: face to face at triage and bedside with documentation aids; dose: brief 30 minute teaching sessions, per interaction during admission; tailoring: to dementia status, communication ability, family-reported signs; modifications: iterative actions across ED, AMU, SW; fidelity: variable, documentation and assimilation of information fragmented pre-intervention.	Implementation via facilitation and co-design, steering group oversight, unit focus groups for reflective practice, champions, visual reminders, protocol use, prompting at triage, training sessions, encouragement of family involvement, medication review processes.	Action=identify dementia promptly, assess and treat pain using dementia-appropriate methods and assimilated information; Actor=Nurses; Doctors; AHPs; Managers; Context=ED, AMU, Surgical Ward in an acute general hospital, UK; Target=older inpatients with dementia, pain management processes; Time=at triage, within first 72 hours, per shift contacts.	Acceptability: improved with visual prompts and concise training; Adoption: commitment to routine pain tool use and triage flags; Appropriateness: higher when family input and toolkits used; Feasibility: constrained by staff shortages and task pressure; Fidelity: inconsistent without prompts, improved with toolkit; Penetration: spread across ED, AMU, SW; Sustainability: dependent on ongoing facilitation and training.
Barriers and enablers to implementing 'DEALTS2' simulation-based train-the-trainer dementia training programme in hospital settings across England: a qualitative study	I — DEALTS2: simulation-based dementia training toolkit, providers: Trust trainers; materials: simulations, role plays, slides, debrief guides; procedures: experiential scenarios with structured debrief; mode: in-person workshops; dose: approximately 4 hours for three Tier 2 subjects; tailoring: adaptable to local needs; modifications: trainers amend scenarios for audience; fidelity: supported by flexible toolkit and debrief, variable where time or resources limited.	Train-the-trainer model, management approval and resourcing, scheduling protected time, low-tech simulations, peer networking, structured debriefs, local adaptation, integration with DSTF policies.	Action=deliver simulation-based Tier 2 dementia training with structured debrief and local adaptation; Actor=Managers; Trainers; Context=acute NHS hospital trusts across England; Target=hospital staff dementia knowledge and practice; Time=scheduled sessions of about 4 hours per course, ongoing rollout.	Acceptability: trainers valued toolkit and approach; Adoption: higher with management support and resources; Appropriateness: flexible simulations fit local needs; Feasibility: constrained by limited time, staffing, non-mandatory status; Fidelity: aided by debriefs and adaptable materials; Penetration: spread across 11 of 12 regions; Sustainability: dependent on protected time and organisational priority.
Person-centred oral hydration care for older people with dementia admitted to acute hospital wards: Empirical research qualitative	R — No distinct DFHC intervention delivered. Routine-care focus: Deliver person-centred oral hydration care.	No formal implementation strategy reported.	Action=deliver person-centred oral hydration care; Actor=Nurses, AHPs, Doctors, Managers; Context=acute wards in a teaching hospital, England; Target=older inpatients with dementia, ward processes and staff practice; Time=per shift and during medication, therapy, and drink rounds	Appropriateness: routines and outsourcing misaligned with PCC; Adoption: reliance on hosts for drink rounds; Feasibility: staff time, access to kitchens and supplies limited; Fidelity: fluid charts incomplete or inaccurate; Penetration: hydration not visible in ward metrics; Sustainability: dependent on organisational support
The experience of hospital staff in applying the Gentle Persuasive Approaches to dementia care	I — Gentle Persuasive Approaches, 1 day workshop, 4 modules; providers: certified GPA coaches with 2 day training; materials: small group exercises, story sharing, video vignettes, role play; procedures: experiential, communication and self protective techniques; mode: in person, groups of 12; dose: single day; tailoring: matched to staff learning needs; modifications: NR; fidelity planned: standardized curriculum, actual: NR	Joint interprofessional training; internal facilitation by a mental health nurse specialist; local educators organized staffing; team huddles and peer reflection; leadership engagement encouraged; protected time requested; no audit or reminders reported	Action=apply GPA person centred communication and de escalation in routine care; Actor=Nurses, Doctors, AHPs, Managers; Context=medicine and mental health units, teaching hospital, Vancouver; Target=patients with dementia; Time=post training during shifts	Acceptability: 95 percent would recommend; Appropriateness: useful and applicable; Adoption: staff reported using GPA techniques and reframing behavior; Feasibility: constrained by time and staffing; Fidelity: NR; Penetration: multiple units trained; Sustainability: uncertain
Nurses' knowledge and beliefs on pain management practices with hospitalised persons living with dementia: A qualitative descriptive study	R — No distinct DFHC intervention delivered. Routine-care focus: Assess and manage pain in PLWD.	No formal implementation strategy reported.	Action=assess and manage pain in PLWD; Actor=Nurses; Context=general medicine, postoperative surgery, telemetry units, acute hospital, USA; Target=persons living with dementia; Time=per shift during hospitalization	Feasibility: limited by time, staffing, communication gaps; Adoption: variable, reliance on vital signs and faces scales; Fidelity: low to best practice tools; Appropriateness: hesitancy due to perceived risks; Sustainability: NR; Penetration: single site
Living with dementia in hospital wards: a comparative study of staff perceptions of practice and observed patient experience	I — Post-evaluation feedback package: written report of recommendations; senior clinician-led lecture and Q&A; ongoing mentoring; environmental cues/signage; dose: single lecture plus ongoing mentorship; tailoring to two wards; fidelity: partial uptake (small EAT gains).	Feedback to managers, lecture and Q&A to all ward staff, ongoing senior clinician mentoring, small environmental tweaks and reminders about privacy and orientation	Action=embed person-centred routines and dementia-friendly cues; Actor=Nurses, AHPs, Managers; Context=two hospital wards, Malta; Target=patients with dementia; Time=per shift, ongoing T1 to T2	Adoption=partial; Feasibility=mixed; Fidelity=low to moderate; Appropriateness=raised awareness; Penetration=limited; Sustainability=NR

Title	Study-focus classification / DFHC intervention (TIDieR elements)	Implementation strategy (Proctor et al.)	AACTT	Findings related to delivering or implementing DFHC
Barriers and facilitators to caring for patients living with dementia in the emergency department: a qualitative study	R — No distinct DFHC intervention delivered. Routine-care focus: Collaborative dementia-informed ED assessment and discharge planning.	Collaborative teams with geriatrics and EMS; allied health pilot presence; reliance on care partner collateral; ad hoc tools and guidance, limited spread; day-shift geriatric consult availability	Action=collaborative dementia-informed ED assessment and discharge planning; Actor=Doctors, Nurses, AHPs; Context=adult EDs, Calgary hospitals; Target=people living with dementia; Time=per ED shift during visit	Acceptability=mixed; Adoption=not widespread; Appropriateness=high for person-centred approaches; Feasibility=constrained by time, staffing, space; Fidelity=variable; Penetration=limited; Sustainability=limited for pilots
Nurses' experiences of delivering acute orthopaedic care to patients with dementia	R — No distinct DFHC intervention delivered. Routine-care focus: Supplement electronic record with oral dissemination of person-specific cues.	No formal implementation strategy reported. Existing supports or practices described: Informal work-arounds: oral handover to supplement EHR; pocket notes with person cues; allocate calm nurses to dementia care; prioritize single rooms where possible.	Action=supplement electronic record with oral dissemination of person-specific cues; Actor=Nurses; Context=acute orthopaedic hip fracture unit, Denmark; Target=team communication and care plans for patients with dementia; Time=per shift handover and ad hoc during shifts	Acceptability=mixed sentiment; Adoption=work-arounds used; Appropriateness=EHR and ward environment not fit for purpose; Feasibility=time pressure and staffing mix limit tailoring; Fidelity=person-centred routines inconsistent
Health professional perspectives on systems failures in transitional care for patients with dementia and their carers: a qualitative descriptive study	R — No distinct DFHC intervention delivered. Routine-care focus: Prepare comprehensive discharge summaries with medication reconciliation and notify GP and RACF.	No formal implementation strategy reported. Existing supports or practices described: Standard processes: medical, nursing and allied health discharge summaries; referrals to community services; no routine pharmacy dose aids; limited dementia-specific discharge guidance.	Action=prepare comprehensive discharge summaries with medication reconciliation and notify GP and RACF; Actor=Doctors; Nurses; AHPs; Managers; Context=acute tertiary hospital, regional NSW; Target=GPs, RACF staff, community services for people with dementia; Time=at discharge	Acceptability=frustration across teams; Adoption=workarounds and selective cognition testing; Appropriateness=general discharge templates not dementia-suitable; Feasibility=service scarcity and time pressure; Fidelity=incomplete summaries; Penetration=variable across services; Sustainability=strained by demand
Nurses' perceptions in caring for people with dementia in Dutch acute hospitals	R — No distinct DFHC intervention delivered. Routine-care focus: Deliver dementia-related preventive interventions and manage challenging behaviour.	No formal implementation strategy reported.	Action=deliver dementia-related preventive interventions and manage challenging behaviour; Actor=Nurses; Context=acute hospital wards, university, teaching and general hospitals, Netherlands; Target=patients with dementia; Time=per shift, during admission and discharge	Acceptability and appropriateness mixed; feasibility constrained by time and staffing; adoption of restrictive measures high; appropriateness of dementia-specific preventive interventions low; training associated with more positive attitudes and perceived responsibility
Radiographers' experiences in working with patients living with dementia in Norway: A qualitative study	R — No distinct DFHC intervention delivered. Routine-care focus: Adapt imaging workflow and communication for PWD.	No formal implementation strategy reported. Existing supports or practices described: Departmental practices noted: scheduling extra time, quieter rooms, caregiver inclusion, use of fast/robust protocols, ad hoc record checks; limited or absent formal protocols.	Action=adapt imaging workflow and communication for PWD; Actor=AHPs; Context=imaging departments in Norwegian hospitals (MRI, CT, NM, general imaging); Target=patients with dementia; Time=per booked and acute examination	Appropriateness and acceptability generally positive for short, prebooked exams; feasibility hindered by missing referral info, time pressure, logistics; fidelity trade-offs when using fast protocols; perceived need for training and procedures
Perioperative Nurses' Experience of Working With Persons Living With Dementia	R — No distinct DFHC intervention delivered. Routine-care focus: Deliver perioperative dementia-sensitive nursing care and safety management.	No formal implementation strategy reported. Existing supports or practices described: Unit supports noted: staffing adjustments, in-person and virtual sitters, fall-risk precautions, proximity to nurses' station, alarms, environmental calming, ad hoc planning and observation routines.	Action=deliver perioperative dementia-sensitive nursing care and safety management; Actor=Nurses; Context=perioperative units, academic hospital, USA; Target=persons living with dementia; Time=preop, intraop, postop, per shift	Acceptability mixed; appropriateness concerns for constant reorientation; feasibility limited by time and staffing; adoption of sitters and restraints reported; fidelity trade-offs under competing priorities; perceived need for education and policies
Implementation of a model of care for hospitalised older persons with cognitive impairment (the Confused Hospitalised Older Persons program) in six New South Wales hospitals	I — CHOPs program, seven-principle bundle: cognitive screening within 24 h, delirium risk assessment with DRAT, assessment and management plans, person-centred communication, staff education, supportive environments; Providers: ward teams with clinical leads and executive sponsors; Materials: delirium alert stickers, whiteboard magnets, EMR icons, lanyard flowcharts, clocks/calendars, toilet/shower signage, DVDs, brochures, Sunflower profile; Procedures: routine screening, risk ID, documentation, family liaison, environmental adjustments; Mode: face-to-face in-services, knowledge cafés, e-learning, DVDs; Dose: site-tailored over ~6–12 months; Tailoring: priorities set from baseline diagnostics; Modifications: site-specific solutions; Fidelity: pre/post audits, surveys, environmental audits	Lead agency support and executive sponsorship; local clinical champions and ward clinical leads; AIM-guided action plans; staff training and mentoring; audit and feedback; workflow prompts and reminders (stickers, icons, magnets); guideline/policy development; environmental modifications; regular implementation teleconferences	Action=implement CHOPs bundle: screen within 24 h, assess risk, manage, communicate, adapt environment; Actor=Doctors, Nurses, AHPs, Managers; Context=acute wards in six NSW public hospitals; Target=older inpatients with or at risk of cognitive impairment; Time=on admission within 24 h and throughout admission per shift	Acceptability, high for education and practices; Adoption high for screening, risk ID, delirium coding; Appropriateness aligned with national standards; Feasibility constrained by staff time and competing priorities; Fidelity high to screening and assessment; Penetration across multiple wards; Sustainability actions via Expert Advisory Group and forums; Antipsychotic prescribing reduction not significant
Redesigning acute care for cognitively impaired older adults: Optimizing health care services	R — No distinct DFHC intervention delivered. Routine-care focus: Coordinate post-discharge clinic follow-up and reconcile plans and medications.	No formal implementation strategy reported. Existing clinic-facing supports and proposals described: EMR admission notifications to outpatient teams; ACE sends evaluation packets to hospital geriatrics clinic; clinic social work assists insurance and transport for appointments; staff education on delirium for clinic-facing nurses; proposals for discharge nurse role and NP continuity bridging to hospital clinics.	Action=coordinate post-discharge clinic follow-up and reconcile plans and medications; Actor=Doctors, Nurses, AHPs; Context=hospital geriatrics outpatient clinics within a university-affiliated safety-net hospital; Target=older adults with cognitive impairment after inpatient stay; Time=at discharge and early post-discharge visits	Feasibility constrained by clinic time and resources; Fidelity undermined by fragmented information and duplicated referrals; Adoption aided by strong geriatrics presence; Penetration supported by EMR alerts and standardized packets; Sustainability linked to staff buy-in and burnout mitigation
Dementia care across a tertiary-care health system: What exists now and what needs to change	R — No distinct DFHC intervention delivered. Routine-care focus: Standardize identification of dementia and coordinate referrals across ED, inpatient, and hospital clinics.	No formal implementation strategy reported. Existing supports or practices described: Existing hospital resources: Psychiatry Consultation Liaison Service, Geriatric Consult Service, Elder Life volunteers, No One Dies Alone; ED strengths include low nurse to patient ratio and a 24-hour crisis line; challenges noted with sitters, electronic sitters, antipsychotic overuse; recommendations include web-based dementia training, a dementia care management program to link services, outpatient education, and repurposing sitters as elder life specialists.	Action=standardize identification of dementia and coordinate referrals across ED, inpatient, and hospital clinics; Actor=Doctors, Nurses, AHPs, Managers; Context=tertiary hospital ED, inpatient wards, hospital-attached outpatient clinics; Target=persons living with dementia; Time=at triage, during admission, at discharge, and early follow-up	Feasibility limited by space constraints, staffing turnover, and time pressure; Fidelity threatened by inconsistent recognition of dementia and overreliance on sitters and antipsychotics; Appropriateness hindered by unclear service ownership; Adoption constrained by training gaps; Penetration reduced when dementia is not identified in records; Sustainability challenged by workload and resource strain
Understanding and integrating the needs and preferences of people living with dementia in the inpatient setting: a qualitative study	R — No distinct DFHC intervention delivered. Routine-care focus: Elicit, document, and apply individual needs and preferences in care.	No formal implementation strategy reported. Existing supports or practices described: Current supports described: bedside Sunflower prompt card; Behaviour Care Plans for complex cases; behaviour huddles; structured handover; proposed hospital admission kit for pre-emptive use.	Action=elicit, document, and apply individual needs and preferences in care; Actor=Nurses, Doctors, AHPs; Context=ACE inpatient ward, tertiary hospital, Australia; Target=inpatients with dementia; Time=on admission, during handover, per shift, at discharge planning	Feasibility limited by staffing and time; Fidelity reduced by unclear responsibility and inaccessible documentation; Acceptability high for MDT huddles and proposed admission kit; Appropriateness improved by bedside prompts and comprehensive behaviour plans; Sustainability contingent on accountable workflows and training
The assessment and management of pain in patients with dementia in hospital settings: a multi-case exploratory study from a decision making perspective	R — No distinct DFHC intervention delivered. Routine-care focus: Systematic pain assessment, documentation and timely reassessment.	No formal implementation strategy reported. Existing supports or practices described: Local pain documentation and tools variably present: Pain Care Plan, locally developed Generic Pain Assessment plus PACSLAC, Abbey Pain Scale trialed; routine post-surgical analgesia protocols and access to specialist pain team, but use inconsistent and often perceived as tick-box.	Action=systematic pain assessment, documentation and timely reassessment; Actor=Nurses, Doctors; Context=acute hospital wards, UK; Target=patients with dementia; Time=per interaction and per shift, with reassessment ideally within 30 minutes after analgesia	Acceptability: low for observational tools; Adoption: limited for Abbey and GPA; Appropriateness: challenged by paperwork perceptions; Feasibility: constrained by time pressure, shift patterns and fragmented records; Fidelity: low, completion without follow up; Penetration: partial across wards; Sustainability: NR

Title	Study-focus classification / DFHC intervention (TIDieR elements)	Implementation strategy (Proctor et al.)	AACTT	Findings related to delivering or implementing DFHC
What influences decisions about ongoing stroke rehabilitation for patients with pre-existing dementia or cognitive impairment: a qualitative study	R — No distinct DFHC intervention delivered. Routine-care focus: Decide on and adapt ongoing stroke rehabilitation.	No formal implementation strategy reported.	Action=decide on and adapt ongoing stroke rehabilitation; Actor=Doctors, Nurses, AHPs; Context=acute stroke unit and inpatient rehab units, NHS UK; Target=stroke patients with pre-existing dementia or cognitive impairment; Time=early admission and over short service windows	Acceptability=constrained by role perceptions; Adoption=lower when rehabilitation framed as improvement-only; Appropriateness=questioned under improvement-first models; Feasibility=limited by time, staffing, short timescales; Fidelity=variable to person-centred approaches; Sustainability=challenged by service limits; Penetration=training inconsistent
Facilitators, Barriers, and Solutions in Pain Management for Older Adults with Dementia	R — No distinct DFHC intervention delivered. Routine-care focus: Assess, record, and manage pain.	No formal strategy implemented; authors recommended: targeted training program, three courses on pain evaluation and management, dementia and pain, and pharmacology; creation of a specific electronic nursing pain register to structure comprehensive assessment and planning.	Action=assess, record, and manage pain; Actor=Nurses; Context=Acute Geriatric Unit, university hospital, Spain; Target=inpatient older adults with dementia; Time=per encounter and per shift	Adoption=variable with experience and knowledge; Appropriateness=lower where pain not treated as fifth constant; Feasibility=limited by time, workload, shift structure; Fidelity=low for documentation and consistent nonpharmacological use; Penetration=NR; Sustainability=NR
Privileges, and Permissions: Theorising Intersectionality and Cultures of Control in the Care of People Living With Dementia in Acute Hospital Settings	R — No distinct DFHC intervention delivered. Routine-care focus: Contain or permit ward walking and apply supervision/alarms.	No formal implementation strategy reported.	Action=contain or permit ward walking and apply supervision/alarms; Actor=Nurses, Managers; Context=NHS acute general medical wards and medical assessment units, England and Wales; Target=inpatients with dementia; Time=per shift during routine timetabled care	Appropriateness questioned (differential control vs person-centred care); Acceptability low for marginalised groups (distress observed); Penetration high of control practices (one-to-one, security, alarms); Sustainability entrenched routines
When people living with dementia say 'no': Negotiating refusal in the acute hospital setting	R — No distinct DFHC intervention delivered. Routine-care focus: Design and deliver requests for care tasks, negotiate refusals and acceptance.	No formal implementation strategy reported.	Action=design and deliver requests for care tasks, negotiate refusals and acceptance; Actor=Doctors; Nurses; AHPs; Context=acute inpatient Healthcare of the Older Person wards, large acute teaching hospital, UK; Target=people living with dementia; Time=during routine bedside encounters and ward rounds	Acceptability=mixed for low-entitlement requests; Adoption=higher for announcements and proposals; Appropriateness=high-entitlement requests with contingency-lowering framed as respectful; Feasibility=low-entitlement formats made refusal more likely, feasibility ↓; Fidelity=task completion improved when entitlement ↑ and contingencies lowered, fidelity ↑; Penetration=NR; Sustainability=NR
"Getting to Know Them": Person-Centered Care for Patients With Dementia in Acute Care	R — No distinct DFHC intervention delivered. Routine-care focus: Deliver person-centered care with consistent communication and tailored calming/engagement.	No formal implementation strategy reported.	Action=deliver person-centered care with consistent communication and tailored calming/engagement; Actor=Nurses; Context=medicine, rehabilitation, neuroscience units, large county teaching hospital, USA; Target=patients with dementia; Time=per shift, during handoffs and routine tasks	Acceptability=high desire for PCC; Adoption=variable with team communication; Appropriateness=PCC seen as best practice; Feasibility=limited by task pace, staffing, environment; Fidelity=NR; Penetration=NR; Sustainability=NR
Improving emotional wellbeing for hospital-based patients with dementia	R — No distinct DFHC intervention delivered. Routine-care focus: Deliver emotion-focused care within routine tasks and interactions.	No formal implementation strategy reported. Practice-enabling approaches described: Leaders model permission to prioritise emotional wellbeing alongside routine tasks; team meetings and supervision to share approaches; peer observation; invite family input and personal information books; use low-stimulation spaces, music, sensory resources and community links; micro-time redistribution for brief check-ins.	Action=deliver emotion-focused care within routine tasks and interactions; Actor=Nurses; Context=two long-stay dementia wards, independent hospital, UK; Target=patients with dementia; Time=throughout shifts in brief, opportunistic moments	Acceptability=valued as dignity and human-rights based; Adoption=increases when leaders explicitly permit "sitting with" patients and teams share what works; Appropriateness=improves with personal information and tailoring; Feasibility=constrained by time pressure and competing tasks, aided by micro-time redistribution; Fidelity=hindered because emotion-focused actions are hard to specify and enforce; Sustainability=threatened by emotional toll on nurses; Penetration=supported by routine embedding within existing tasks and team norms
Nurses' perspectives on shared decision-making in the daily care of hospitalized patients with dementia: An exploratory qualitative study	R — No distinct DFHC intervention delivered. Routine-care focus: Apply shared decision-making in daily nursing care and in multidisciplinary treatment planning.	No formal implementation strategy reported.	Action=apply shared decision-making in daily nursing care and in multidisciplinary treatment planning; Actor=Nurses; Context=general hospital wards, Netherlands; Target=patients with dementia, with relatives; Time=throughout admission during routine care and scheduled family meetings	Acceptability=valued for dignity, adherence, satisfaction; Adoption=limited by physician-ownership norms and variable nurse involvement; Appropriateness=reduced when patient goals are omitted; Feasibility=constrained by time pressure and communication challenges; Fidelity=low to SDM models, steps applied unconsciously; Penetration=variable across wards and decisions
Co-development of BRAIN-TRK: Qualitative examination of acceptability, usability and feasibility of an App to support nurses' care for patients with behavioural and psychological symptoms of neurocognitive disorders in hospital	I — BRAIN-TRK point-of-care App for nurses on iPads at the bedside, combining 4AT cognitive screen, risk factor checklist, tailored evidence-based BPS strategies and effectiveness monitoring for one patient per device; ward nurses and facilitators enter demographics, preferences, behaviours, select strategies and rate effects; App prompts reassessment at least every 8 hours and visualises trends over the admission; content derived from delirium and BPSD best practice guidance; recommendations tailored to each patient's risk factors and previous responses; 26 iteratively updated versions released during development and a 3-month pilot, with fidelity variable and evaluation sections often incomplete and use observed with 44.7% of eligible patients	Integrated KT package with co-development workshops, online education and a peer facilitation model; ward-based nurse facilitators and NUMs championed use; dedicated training sessions and one-to-one coaching on wards; iPads secured at bedside and made available for unsupervised practice; researchers provided ongoing on-ward support, troubleshooting and iterative software updates; in-App prompts to reassess at least every 8 hours; social expectation on participating wards that nurses use the App for eligible patients, but implementation limited to two general medicine wards during the pilot	Action=screen cognition and BPS, select and evaluate tailored strategies using the BRAIN-TRK App at the bedside; Actor=Nurses; Context=general medicine inpatient wards in two acute public hospitals, Australia; Target=hospitalised older adults with neurocognitive disorders or behavioural and psychological symptoms; Time=at admission and at least every 8 hours, throughout shifts and at handover during the 3-month pilot	Acceptability: generally positive when nurses were familiar with the App and perceived clear benefits for tailoring care and monitoring; Adoption: used for 17 of 38 observed eligible patients and 32 patients overall, with partial completion of risk and strategy sections; Appropriateness: content and level of detail viewed as relevant, basic and practical for busy wards; Feasibility: supported by bedside availability and alignment with the nursing process, but constrained by workload, duplicate documentation, device logistics and time pressure; Fidelity: assessment sections used more consistently than evaluation components, many strategies lacked follow-up ratings; Penetration: implementation confined to two wards, with uptake influenced by peer norms and facilitators; Sustainability: short trial period, authors note need for longer-term evaluation and integration with routine records

Title	Study-focus classification / DFHC intervention (TIDieR elements)	Implementation strategy (Proctor et al.)	AACTT	Findings related to delivering or implementing DFHC
Working with a robot in hospital and long-term care homes: staff experience	I — Double telepresence robot for video calls between older adults with dementia and their families; wheeled device with adjustable screen driven remotely by family; in hospital unit robot docked at nursing station then moved to patient rooms before calls; staff ensure charging, Wi Fi connection and safe placement; families connect via browser or app using link or QR code; calls used for social visits and to support care such as therapy sessions and end of life presence; no resident operation required and calls typically ended by family members	Collaborative Action Research cycles with staff champions and research team; CFIR guided implementation planning; 20–30 minute in person or virtual staff training with demonstrations, safety remarks and interactive games; staff packages with posters, videos, newsletters and user guides; ongoing weekly and monthly check ins and field visits; recognition of staff in newsletters and special events; staff champions orienting new colleagues; family orientation sessions; robots kept powered on and connected to designated Wi Fi; staff asked to clean devices, keep them charged and coordinate scheduled calls	Action=prepare, position, monitor and maintain telepresence robots to support family–patient video calls; Actor=Nurses, AHPs, Managers; Context=dementia care unit in a publicly funded hospital in British Columbia, Canada; Target=older adults with dementia in hospital and their communication with families; Time=at scheduled and ad hoc call times, including during therapy sessions and end of life, throughout routine shifts	Acceptability: initial fear and privacy concerns reduced after training and positive experiences; Adoption: robots used by engaged staff champions and some colleagues but uptake limited by uneven training and resource constraints; Appropriateness: seen as aligning with goals of maintaining family connection and reducing staff burden as families can call directly; Feasibility: enabled quick set up and minimal staff time for calls but constrained by unstable Wi Fi, staffing shortages, and physical space; Fidelity: use depended on champions and sometimes missed scheduled calls or inconsistent charging; Penetration: implemented on one hospital dementia unit within a broader project across four LTC homes; Sustainability: ongoing technical support and structural investment identified as prerequisites for longer term use
Agitation near the end of life with dementia: An ethnographic study of care	R — No distinct DFHC intervention delivered. Routine-care focus: Assess and respond to agitation in people with dementia nearing end of life.	No formal implementation strategy reported. Existing supports described: Hospital B had a dementia lead, dementia training for staff, dedicated one to one specials, environmental cues such as forget me not magnets and five things about me boards; Hospital A reported minimal dementia training, high reliance on temporary staff and security staff for agitation, and little structured implementation support.	Action=assess and respond to agitation in people with dementia nearing end of life; Actor=Nurses, Doctors, AHPs, Managers; Context=older adult inpatient medical wards in a district general and a teaching hospital in England; Target=inpatients with moderate to severe dementia nearing end of life; Time=during each shift, particularly during personal care, night time and acute agitation episodes	In the moral judgement model, agitation management was seen as outside core nursing work, leading to low adoption and feasibility of proactive responses, high rates of non response such as looking but not intervening, and greater use of security staff; in the needs based model, agitation was framed as distress or unmet need, with higher adoption and fidelity of comfort based and investigative responses, more social time with patients, and better perceived appropriateness of person centred care; across settings, penetration and sustainability of needs based practice depended on dementia specific training, leadership, staffing levels, and organisational valuing of older peoples care
Dementia care in acute hospitals: A qualitative study on nurse managers' perceived challenges and solutions	R — No distinct DFHC intervention delivered. Routine-care focus: Organise and deliver dementia care within acute medical wards and propose system level improvements.	No formal implementation strategy reported.	Action=organise and deliver dementia care within acute medical wards and propose system level improvements; Actor=Nurses; Managers; Context=acute medical wards, single public general hospital, Malta; Target=inpatients with dementia or cognitive impairment and complex social needs; Time=throughout admission, discharge planning and ward transfers	Acceptability of current dementia care and ward culture described as low; appropriateness of an acute throughput focused hospital strategy for large numbers of patients with dementia was questioned; feasibility and fidelity of person centred practices and restraint guidelines were constrained by staffing shortages, fixed routines, ward transfers and disorientating environments; sustainability of improvements was seen as dependent on whole systems change, leadership support and better integration with community and long term care
Creating a toolkit with stakeholders for leveraging tablet computers to support person-centred dementia care in hospitals	I — Tablet based family video intervention to support person centred dementia care: family members self record one minute reassuring videos on their own devices and send them to the nurse researcher, videos are uploaded to ward tablets and played to patients with dementia during care tasks to reassure and engage them, content tailored to individual interests and specific ADLs, frequency and timing determined by ward staff, fidelity supported by a standard short video format and toolkit materials but actual use varies by unit	Participatory action research with monthly meetings; staff focus groups and stakeholder interviews to surface barriers and ideas; co production with patient, family and staff partners to design the intervention workflow; development of short user videos, a family brochure and a staff pocket card with simple instructions; nurse leader acting as on ward champion; local adaptation of hardware (mechanical arm, storage, charging) and privacy procedures to embed the intervention into routine care	Action=use ward tablets with pre recorded family videos to support communication, reduce distress and assist ADLs for patients with dementia; Actor=Nurses and AHPs, supported by Managers; Context=older adult mental health unit and acute medical wards in two large urban hospitals in Canada; Target=hospitalised older adults with dementia and their care interactions; Time=during daily care activities and at times of distress or resistance to care	Acceptability: staff viewed tablet family videos as a helpful way to reassure patients, reduce loneliness and responsive behaviours; Adoption: increased when staff had simple instructions, ergonomic hardware and visible leadership support, but was limited by user inexperience and technical issues; Appropriateness: perceived as consistent with person centred dementia care and as an alternative to restraints and medications, particularly valuable during infection control and visiting restrictions; Feasibility: initially constrained by skills gaps, privacy concerns, mechanical instability, Wi Fi and power issues, and workload but improved after toolkit and local adaptations; Fidelity: supported by standardised one minute video messages and prompts in the toolkit, although use remained opportunistic; Sustainability: toolkit, online resources and integration into ward routines aimed to support ongoing use beyond the study period
Implementing the PIE (Person, Interaction and Environment) programme to improve person-centred care for people with dementia admitted to hospital wards: a qualitative evaluation	I — PIE practice improvement programme: ward-based cycles of observation of Person, Interactions and Environment by PIE teams, followed by reflective team meetings, identification of goals and small-step action plans, implementation and review using structured observation and action-planning tools and a workbook, delivered face to face on wards by nurses, AHPs and dementia specialist staff, repeated periodically over up to 18 months with ward-specific tailoring, shortened observations and variable fidelity across wards	Introductory workshops to train PIE teams; formation of multi-professional ward implementation teams; external clinical leadership and facilitation by dementia nurse specialists and practice development leads; ongoing observation–action–review meetings; cross-ward workshop to clarify shared purpose; integration with trust dementia strategies; use of awaydays and local ward managers to support and communicate changes	Action=conduct PIE observation–reflection–action cycles to change ward routines and environments toward person-centred dementia care; Actor=Nurses, AHPs, Managers; Context=acute hospital wards in five NHS trusts in England caring for older people with dementia; Target=staff practices, interactions and modifiable ward environments affecting inpatients with dementia; Time=over approximately 18 months with periodic observations, action-planning meetings and reviews	Acceptability generally high where PIE was seen as meaningful and empowering; adoption full in two wards, partial in two and absent in six; appropriateness influenced by perceived fit with existing quality goals and other dementia initiatives; feasibility constrained by staffing levels, time, space and organisational turbulence; fidelity variable, with observation cycles and action plans only partly enacted on many wards; penetration limited beyond early enthusiasts; sustainability challenging even in full implementers

Title	Study-focus classification / DFHC intervention (TIDieR elements)	Implementation strategy (Proctor et al.)	AACTT	Findings related to delivering or implementing DFHC
The barriers and facilitators to implementing dementia education and training in health and social care services: a mixed-methods study	I — Multiple dementia education and training programmes across UK services, typically local dementia education or champion programmes; providers: dementia training leads, facilitators and clinical staff; materials: face to face teaching sessions, experiential simulations, case studies, talks from people with dementia, e learning modules, self directed workbooks; procedures: group teaching, discussion of case studies, experiential exercises and reflection; mode: mainly on site group workshops plus some online or workbook based learning; dose: varies by package from single sessions to multiday or repeated sessions; tailoring: in some organisations content adapted to service type and staff role; modifications: NR; fidelity: mainly monitored through attendance and staff self report about applying learning rather than formal fidelity checks	Strategies included appointing dementia training leads and facilitators, integrating dementia education into organisational training strategies, providing protected paid time and incentives to attend, offering mentorship, supervision and feedback to embed learning, using dementia champions, encouraging mixed group and peer to peer learning, securing visible executive leadership support, and aligning training messages with person centred care culture and organisational policies	Action=apply dementia training learning in routine practice to deliver person centred dementia care; Actor=Doctors, Nurses, AHPs and Managers who complete dementia training; Context=acute hospital wards and mental health inpatient units within UK health and social care organisations; Target=care processes, staff behaviours and ward culture affecting people with dementia; Time=after completion of training, during day to day work on every shift	High perceived adoption and acceptability of dementia training, with over 85 percent of surveyed staff reporting they had been able to apply learning to some extent and only about half feeling strongly supported by their organisation; feasibility and fidelity were constrained by time pressure, staffing shortages, financial limits and non dementia friendly environments, alongside competing demands; interactive, face to face and experiential training was viewed as more appropriate and effective than stand alone e learning or workbook based training; supportive leadership, dementia focused culture, mentorship, supervision, peer support and incentives improved uptake, embedding and sustainability of behaviour change
Components of impactful dementia training for general hospital staff: a collective case study	I — Interactive dementia education and training programmes for general hospital staff, including 1 day dementia awareness, multi day dementia champions and study days; delivered by dementia leads, nurse educators, matrons and external trainers; face to face small group sessions using didactic teaching, videos, scenarios, experiential and simulation activities with briefing and debrief; doses from short induction sessions to multi day courses spaced over months; content tailored to general hospital context and staff roles; materials iteratively refined based on training needs analysis and participant feedback; fidelity variable, with some staff not trained and inconsistent application of learning in practice	Training embedded via Trust wide dementia training plans, mandatory courses for new nurses, dementia champions cascading learning on wards, senior leadership endorsement from Chief Nurse and Trust management, supportive ward managers encouraging and rostering staff to attend, accessible on site training facilities and flexible delivery, but constrained by staffing shortages, vacancies, attrition and reliance on untrained agency staff	Action=attend dementia training and apply person centred dementia care behaviours; Actor=Nurses, AHPs, Managers, some Doctors; Context=general hospital wards in three NHS acute hospital Trusts in England; Target=inpatients with dementia and their family carers; Time=after training during routine shifts, ongoing	Training was acceptable and valued, especially interactive face to face sessions, with staff and managers reporting increased knowledge, empathy, confidence and changed communication and activity practices; adoption and penetration were incomplete because not all staff groups, especially medics and agency staff, attended and some wards still showed limited activity and negative interactions; appropriateness was high due to tailoring to roles and hospital context; feasibility was undermined by time pressure, staffing shortages and lack of protected study leave; fidelity of practice change was variable, with DCM observations showing both good person centred care and persistent task focused, restrictive or poorly communicative interactions; sustainability depended on ongoing leadership from dementia training leads and supportive organisational culture
Visual identifiers for people with dementia in hospitals: a qualitative study to unravel mechanisms of action for improving quality of care	I — Visual identifier systems for people with dementia in acute hospitals, using symbols such as butterflies and forget me not flowers on wristbands, bedside boards, whiteboards, notes and electronic record flags to mark patients with dementia or suspected dementia and link them to additional support; identifiers sometimes paired with personalised information tools (eg This is me booklets, bedside posters) describing preferences and needs; applied by ward and admissions staff with variable consent procedures (opt out vs explicit consent) and eligibility rules; intended to be used continuously during admission to prompt dementia aware care, but application and use were inconsistent across wards and sites	Implemented via local dementia strategies and dementia friendly initiatives, led by dementia leads or senior dementia nurses; use of hospital wide electronic alerts, bedside symbols and patient profile documents; training and awareness raising for staff about identifiers and dementia care, though depth and coverage (especially for locum and agency staff) were limited; organisational policies to track and reduce ward transfers for patients with dementia and to fast track them through some diagnostic pathways; embedding personalised information documents alongside identifiers, but with weak routines and accountability for completing and using them	Action=apply and respond to visual identifiers and linked personalised information when planning and delivering hospital care; Actor=Nurses, Doctors, AHPs, Managers; Context=acute hospital wards in four NHS acute hospitals in England; Target=inpatients with dementia and their carers; Time=at admission and throughout the hospital stay, at every ward contact	Identifiers were generally viewed as appropriate and potentially useful for raising awareness and coordinating care for people with dementia, supporting acceptability among staff; adoption and penetration were high at organisational level, with most hospitals operating identifier schemes, but fidelity and consistency of application at ward level were poor, leading to misclassification and some eligible patients not being flagged; feasibility was constrained by unclear responsibility for applying identifiers, lack of standardisation of symbols, incomplete or inaccessible personalised information booklets and staff time pressures; appropriateness was undermined when identifiers became labels without linked person specific information, encouraging assumptions rather than person centred care; potential paradoxical effects and harmful externalities included stigma, discrimination and depersonalisation when staff held negative beliefs about dementia or relied on the label instead of engaging with the individual
A qualitative focus group discussion study on the experiences of Certified Nurses in Dementia Nursing related to effective staff education	I — DCNs function as in-house dementia specialists who plan and deliver staff education in acute wards, including individual bedside coaching, participation in care for patients with BPSD, expanded multidisciplinary conferences from admission to discharge, briefing sessions, and sharing written care guidelines; education is mostly on-the-job, embedded in routine care, with DCNs modelling dementia care at the bedside and gradually delegating care to ward nurses to build independent practice	Implemented via the DCN role and, where present, dementia care teams, with DCNs liaising between ward staff and managers, negotiating relief staffing and support systems, organising expanded conferences with hospital and community providers, mixing on-the-job coaching with workshops, and using feedback sessions and videos to show care effects; implementation is constrained by DCNs often being the only specialist in each hospital and sometimes combining DCN and ward staff roles	Action=provide on-the-job dementia care education and continuous practical support to hospital staff; Actor=Certified Nurses in Dementia Nursing (Nurses); Context=acute wards in 10 general hospitals in central Japan; Target=nurses and other hospital staff caring for inpatients with dementia; Time=ongoing during hospitalisation, with repeated ward visits, early intensive support and later stepwise withdrawal to encourage independence	Education and support from DCNs were perceived as acceptable and useful by nurses and volunteers, improving confidence and practice, indicating good acceptability; tailored bedside coaching and role modelling were appropriate and feasible in busy acute settings, but feasibility was reduced by time pressure, DCNs' dual roles and limited numbers of specialists; the approach aimed to build sustainable, independent dementia care on wards through gradual delegation and supporting reflection, but sustainability depends on supportive managers and existence of a dementia care team
Deconstructing dementia and delirium hospital practice: using cultural historical activity theory to inform education approaches	R — No distinct DFHC intervention delivered. Routine-care focus: Learning about and sharing knowledge of the confused older patient to guide care.	No formal strategy implemented; authors recommended: restructure ward processes and environments to support confused older patients, reduce ward moves, relax visiting to enable carers to share knowledge, support formal and informal interprofessional education led by Liaison mental health teams, create spaces for learning about the patient, empower low status staff to share expertise, use contradictions in the ward activity system as foci for expansive learning; no specific dementia friendly hospital care intervention was implemented or evaluated within this study.	Action=learning about and sharing knowledge of the confused older patient to guide care; Actor=Doctors, Nurses, AHPs and Managers working in acute hospital wards and Liaison mental health teams; Context=acute non-psychiatric wards in a District General Hospital in North East England, with Liaison old age psychiatry input; Target=older inpatients with dementia or delirium and the ward systems caring for them; Time=throughout the admission, especially during ward rounds, handovers, clinical meetings, night shifts and episodes of agitation	Implementation outcomes are not formally measured but findings indicate limited adoption and feasibility of person centred dementia and delirium care in acute wards, low penetration of psychosocial approaches compared with medical task focused care, variable acceptability of confused patients within busy acute cultures, reduced fidelity to safe prescribing messages for psychotropics under ward pressure, and threats to sustainability of learning about the patient due to fragmentation, frequent ward moves and rigid role boundaries

Title	Study-focus classification / DFHC intervention (TIDieR elements)	Implementation strategy (Proctor et al.)	AACTT	Findings related to delivering or implementing DFHC
The impact of an education intervention on the approach of hospital staff to patients with dementia in a Danish general hospital setting: An explanatory sequential mixed-methods study	I — Dementia education intervention for entire workforce: mandatory e-learning on dementia in hospital settings; one day 6 hour interdisciplinary classroom course with group work on pathophysiology, delirium, person centered care, communication, ethical issues and staff specific self protective skills; educators were geriatricians, nurses and healthcare assistants from the dementia friendly hospital team; delivery via online module plus face to face teaching in mixed staff groups; single course dose per staff member with flexible timing; parallel problem based learning sessions in all departments facilitated by dementia specialists to translate learning into practice; dose and frequency of PBL varied by workload; fidelity planned as universal participation in the course and regular PBL, actual PBL attendance depended on department capacity	Large scale hospital wide training strategy embedded in a national dementia friendly hospital project; inclusion of all staffing groups rather than only clinical staff; interdisciplinary learning teams and peer learning methods; use of mandatory e-learning and scheduled one day course as adoption levers; dementia specialists as ongoing facilitators for department level problem based learning sessions; expectation from the project team that PBL would be held regularly with mixed professions when workload allowed; leadership and gatekeepers in departments assisted recruitment; education aligned with other dementia friendly initiatives at the hospital	Action=apply shared person centered dementia care and interdisciplinary cooperation in everyday hospital work; Actor=Doctors, Nurses, AHPs, Managers; Context=smaller general hospital within Odense University Hospital across surgical, medical, para clinic and technical administrative departments; Target=inpatients with dementia and their care pathways during hospital admission; Time=throughout shifts after completion of e learning and the one day course with ongoing problem based learning sessions	Knowledge, attitude and competence scores increased significantly from pre intervention to one and five months for all staff and for authorized and non authorized groups (adoption, appropriateness); qualitative data showed a committed interdisciplinary cooperation, shared knowledge base and common ethos prioritising person over task (acceptability, penetration); staff described more dialogue, mutual help and joint problem solving as embedded practice but expressed concern about maintaining focus over time and relying on single measurement points (sustainability)
CogChamps: impact of a project to educate nurses about delirium and improve the quality of care for hospitalized patients with cognitive impairment	I — CogChamps dementia and delirium practice change intervention: two day workshop education for RN champions on dementia, delirium, assessment, non pharmacological management, leadership and change skills; development of ward specific Action Plans with at least three measurable actions for delirium prevention, identification and management; use of bedside teaching, modelling, lanyards and checklists (e.g. PITCHED acronym), Cognition Corner resources, posters and patient or family information; delivery via group workshops plus on ward coaching by CogChamps and seconded facilitator CogChamps; five month implementation phase with champions working alongside ward nurses without protected time, facilitators in off line roles; fidelity monitored through mini audits of cognitive assessments and monitoring of Action Plan progress, but resource use not routinely audited	Distributed leadership and champion based implementation using self nominated RN Cognition Champions; executive support via hospital steering committee with Nurse Unit Managers and medical consultants; use of evidence, context and facilitation constructs with research team mentors; weekly meetings and email support, use of communication books; ward specific Action Plans with clear targets, timeframes and assigned responsibilities; seconded CogChamps as facilitators providing on ward mentorship, modelling and feedback; periodic chart and room audits with observational feedback; alignment with national Caring for Cognitive Impairment campaign and existing dementia initiatives	Action=perform structured cognitive assessments, identify delirium risk and implement multi component non pharmacological care guided by Action Plans; Actor=Nurses (CogChamps, facilitator CogChamps and ward nurses); Context=acute medical and surgical wards in a large tertiary referral hospital in South East Queensland, with comparison control hospital wards; Target=older inpatients aged ≥65 years with cognitive impairment including dementia, delirium, confusion or memory problems; Time=within 24 hours of admission for assessment and throughout routine nursing care during the five month implementation phase and three month follow up	Implementation outcomes included increased formal cognitive assessments within 24 hours of admission at the intervention hospital (from 35% at baseline to 69% post implementation, 72% at follow up) largely due to increased nurse initiated assessments, and increased informal documentation of cognition; analgesia administration increased and benzodiazepine use decreased post implementation but both changes waned by follow up; no consistent change in reorientation, assistance at meals or antipsychotic use, and nurse patient interaction behaviours remained similar to control wards; practice improvements were strongest where Action Plan goals were explicit, measurable and responsibility was clearly assigned (adoption, fidelity), while sustainability depended on continued organisational priority and facilitation (sustainability, feasibility, appropriateness)
Care of patients with dementia in an acute trauma and orthopaedics unit	I — dementia toolkit with purple folder; traffic-light sheet; pain scale; delirium flow chart; provided by nurses; paper materials; used during admission; tailored by family info; fidelity variable early on; refined over cycles	staff education; lunchtime and ad hoc training; managerial support; dementia champions; workflow cueing via coloured folders	Action=use dementia toolkit to support dementia-sensitive care; Actor=Nurses; Context=acute trauma and orthopaedics unit, teaching hospital, Northern Ireland; Target=patients with dementia; Time=each admission and each shift	Improved adoption; feasibility increased with flexible training; fidelity improved after education; penetration increased across ward; early low fidelity identified as barrier
Caring for people with dementia in hospital: findings from a survey to identify barriers and facilitators to implementing best practice dementia care	R — No distinct DFHC intervention delivered. Routine-care focus: Identify perceived barriers and facilitators to best practice dementia care.	No formal implementation strategy reported.	Action=identify perceived barriers and facilitators to best practice dementia care; Actor=Nurses;Doctors;AHPs;Managers; Context=emergency departments and medical wards in two Australian hospitals; Target=best practice dementia care; Time=single survey period	Feasibility was constrained by staffing, time, the physical environment and limited dementia knowledge; specialist services, appropriate equipment and orientation cues facilitated best-practice care.
The use of the truth and deception in dementia care amongst general hospital staff	R — No distinct DFHC intervention delivered. Routine-care focus: Deciding how to respond to dementia related questions and behaviours using truth, distraction, passing responsibility or lying.	No formal implementation strategy reported.	Action=deciding how to respond to dementia related questions and behaviours using truth, distraction, passing responsibility or lying; Actor=Doctors, Nurses, AHPs, Managers; Context=older people's wards in acute general hospitals in north west England; Target=patients with dementia admitted to hospital; Time=whenever dementia related triggers arise during routine shifts	No formal implementation outcomes were measured, but staff reported low clarity and inconsistent practices around truth and deception, with poor communication, limited guidance and role ambiguity undermining feasibility and fidelity of consistent dementia friendly communication, while framing responses as in the patient's best interests supported perceived appropriateness and acceptability of selective deception.
Nurses' Perspectives and Experiences of Using a Bed-Exit Information System in an Acute Hospital Setting: Mixed Methods Study	I — Bed-exit information system (BES) using a pressure sensitive sensor mat as a mattress topper with radio transmitter and receiver connected to the existing ward call system; intended for patients with cognitive impairment and motor agitation or bed leaving risk; placed under the bed mattress and left in situ during the admission when indicated; nurses assessed cognitive impairment and independently decided which patients should receive the mat, focusing on those cognitively impaired, mobile and lively; training on device handling and intended scenarios delivered by a study nurse in ward based sessions during early to late shift handover, with the option for additional training if needed; BES available during P1 for 126 days and actually used on 20% of survey days with cognitively impaired patients; no differentiation of BES alarms from other calls in the call system; use tailored to individual patient behaviours and ward routines, with occasional removal when alarm frequency or workload was perceived as too high; fidelity variable, with reliable technical function but inconsistent deployment and some non use despite perceived risk.	Training sessions for all ward nurses on each participating ward at shift handover covering handling, scenarios of use and manufacturer guidance; opportunity for extra training sessions on request; nurses given autonomy to adopt and adapt BES use in daily practice (deciding who gets a mat and when); integration of BES alarms into existing call system infrastructure without new hardware on staff side; preparatory workshops with nurses to co define adverse events of interest; routine prevalence documentation of cognitively impaired patients and adverse events across P0 and P1; no formal ongoing supervision, audit and feedback, or policy mandate reported, and implementation relied on local nurse champions and ward culture.	Action=using a bed-exit information system to monitor and respond to unsupervised bed leaving risk in cognitively impaired inpatients; Actor=Nurses; Context=neurosurgery, surgery and radiology wards in an acute university medical center in Freiburg; Target=patients with cognitive impairment and tendency to leave bed or wander unsupervised; Time=during the 126 day implementation phase P1, especially at night and whenever patients with cognitive impairment and bed exit risk were present on the ward.	Implementation outcomes: Acceptability - nurses generally positive about IT and saw BES as a useful technical aid in selected scenarios, but high pre implementation expectations about workload reduction and fall prevention were only partly met; Appropriateness - BES perceived as appropriate mainly for mobile, cognitively impaired patients at risk of unsupervised night time bed exits or wandering, and less helpful for very immobile patients or complex behavioural patterns; Adoption - nurses used the system for a subset of eligible patients, with BES deployed on 20% of survey days with cognitively impaired patients and integrated as one tool among several strategies; Feasibility - heterogeneity of patient conditions, undifferentiated call signals, information overload and alarm fatigue made operationalization challenging and led to occasional system removal; Fidelity - alarms were generally technically reliable but nursing responses varied, with some alarms treated as routine background noise and some patients not fitted with mats despite perceived risk; Penetration - implementation limited to three wards and a minority of patients with cognitive impairment; Effectiveness related outcomes - overall adverse event rate per cognitively impaired patient day decreased by a factor of about 0.61 between P0 and P1, but with substantial variation between wards, and nurses' perceived workload and strain remained high, with only a subset reporting reduced strain when using BES.

Title	Study-focus classification / DFHC intervention (TIDieR elements)	Implementation strategy (Proctor et al.)	AACTT	Findings related to delivering or implementing DFHC
Junior doctor dementia champions in a district general hospital (innovative practice)	I — Junior doctor dementia champions and dementia and delirium (DaD) team implementing dementia CQUIN and a multifactorial forget me not scheme in an acute hospital, including dementia screening questions embedded in clerking pro formas for patients aged 75 plus, routine use of AMTS, confusion screens, collateral history, investigations and referral pathways, and ward level forget me not components such as information leaflets for relatives, car parking permits, a hospital passport, patient identifying stickers on ward lists and an 8 things about me form completed with patients and families; delivered primarily by FY1 junior doctors supported by a consultant and registrar, initially on their own wards then handed over as they rotated every four months, with the champions setting up the scheme with new teams; intervention mode was face to face ward work, data collection and documentation changes rather than formal training sessions; dose was continuous across the year with champions active on each ward; tailored to ward context as champions adapted materials and processes; fidelity high for CQUIN documentation with almost 100 percent compliance achieved and sustained, but less formally measured for the wider relational components of the forget me not package.	Junior doctor led quality improvement approach using dementia champions as change agents, with senior consultant backing; embedding dementia CQUIN screening into admission clerking pro formas to hard wire case finding; ongoing audit of CQUIN achievement and feedback to drive improvement; peer to peer influence as champions recruited colleagues and handed over champion roles when rotating posts; structural support through recognition of champions and access to management and teaching forums; use of the forget me not scheme as a concrete ward level tool to focus staff attention on dementia care; informal supervision and mentoring from the consultant lead, plus opportunities to present and write up the project which reinforced engagement and sustainability.	Action=implementing and sustaining dementia screening, assessment and the forget me not dementia friendly care scheme on acute wards; Actor=Doctors (junior doctors as champions, supported by senior doctors); Context=acute district general hospitals in England, including elderly care, hip fracture, surgery and other wards; Target=older inpatients with dementia or at risk of cognitive impairment, plus ward teams responsible for their care; Time=within 72 hours of admission for screening and then throughout each four month junior doctor rotation and across the foundation year.	Implementation outcomes: Adoption, junior doctor dementia champions established on multiple wards, with peers joining through interest and snowballing; Fidelity, dementia CQUIN case finding, assessment and referral achieved almost 100 percent compliance and maintained over time, indicating high fidelity to screening processes, though fidelity of broader person centred components was less explicitly measured; Acceptability, champions described enthusiasm, sense of making a difference and personal and team satisfaction, while many non champions recognised dementia care gaps and a need for training; Appropriateness, junior doctor led dementia work and the forget me not scheme were seen as fitting the acute hospital context and the dementia CQUIN requirements; Feasibility, champions balanced project work with service duties, but questionnaire data highlighted perceived barriers such as being too busy or seeing some tasks as nursing roles; Sustainability, high CQUIN compliance and continued use of the scheme beyond the first year suggest sustained practice change, supported by rotational handover of champion roles and ongoing senior support; Secondary outcomes, champions reported improved communication skills, better understanding of holistic dementia care and greater engagement with families.
Nurses' Experiences in Caring for Older Adults With Responsive Behaviors of Dementia in Acute Care	R — No distinct DFHC intervention delivered. Routine-care focus: Provide person centred nonpharmacological and pharmacological responses to responsive behaviours while maintaining safety.	No formal implementation strategy reported. Existing supports or practices described: Existing supports include Behavioural Supports Ontario BSO clinicians and specialty teams providing assessments and care plans; prior dementia education such as Gentle Persuasive Approaches GPA and PIECES workshops; occasional behavioural rounds and huddles to discuss patients with responsive behaviours; ad hoc involvement of families and one to one staff such as personal support workers and Bartimaeus workers; recommendations for a designated safe dementia unit with trained staff and personal alert systems.	Action=provide person centred nonpharmacological and pharmacological responses to responsive behaviours while maintaining safety; Actor=Nurses and AHPs; Context=acute medical units in an urban teaching hospital in Southern Ontario Canada; Target=older adults with dementia experiencing responsive behaviours in hospital; Time=throughout admission when responsive behaviours occur during each shift	Limited time heavy workload acute care priorities and understimulating environments reduced feasibility and fidelity of person centred dementia care; inadequate staffing and variable access to one to one support constrained adoption of nonpharmacological strategies; reliance on chemical restraints in desperate situations reflected workarounds that may undermine appropriateness and acceptability; strong interprofessional collaboration continuity of care family involvement behavioural rounds and BSO input supported adoption appropriateness and perceived sustainability of more dementia friendly practices
Assessing the Attitudes of Greek Nurses Toward Computerized Dementia Screening	I — Computerized dementia screening tests on tablet PCs, brief 10–15 minute cognitive tasks covering major cognitive domains, with automated administration, scoring and interpretive report, designed for self administered or nurse supervised use with older hospital patients	Planned implementation through dementia and ICT training for nurses, use of digital dementia champions, and organizing a hospital level plan to integrate computerized screening into daily routines and workflows	Action=administer brief computerized dementia screening tests to older inpatients; Actor=Nurses; Context=urban public general hospitals in Thessaloniki, Greece; Target=older adults with possible cognitive impairment admitted to hospital; Time=approximately 10–20 minutes per patient during a shift when screening is indicated	Acceptability and feasibility of computerized dementia screening were high among nurses, but perceived feasibility was constrained by equipment costs, insufficient training, lack of an integration plan into daily hospital routines, and time required for staff training and per patient assessment
