# Supplementary material for: Advancing dementia-friendly hospital care: a systematic review of healthcare professionals’ reported barriers and facilitators using the theoretical domains framework
Source: Age Ageing. 2026 Sep 22;55(9):afag285. doi: 10.1093/ageing/afag285 (PMC13596736; doi:10.1093/ageing/afag285)
Supplement: Supplementary file 1 [file supplementary_material_afag285.zip › aa-26-1308-File004.pdf]

Advancing Dementia-Friendly Hospital Care: A Systematic Review of Healthcare Professionals' Reported Barriers and Facilitators Using the Theoretical Domains Framework

Appendix 3. Full coding matrix of barriers

Supplementary Data

Full coding matrix of barriers

Title	Author(s)	Barrier quotations and granular barrier descriptions	TDF Domains – Barriers	Barriers specific to profession	Barriers per profession → TDF
Facilitators and barriers to implementing a specialized care unit for persons with cognitive impairment in an acute geriatric hospital: a process evaluation	Adlbrecht L; Karrer M; Helfenberger N; Ziegler E; Zeller A	we have already had so many interruptions (p.6) : repeated uncontrollable contextual changes, especially the COVID 19 pandemic and staffing shifts, keep stopping and restarting the project and erode implementation momentum; "it is neither fair to the patients nor to the nursing staff" (p.8) : nurse staffing levels and workload are too tight to deliver individualized dementia friendly care safely and consistently on the specialized unit; "you reach the limit" (p.9) : when several very high need cognitively impaired patients are admitted at once, the team feels overwhelmed and becomes reluctant to admit more similar patients; "processes on the unit were designed around the workflow" (p.7) : tightly synchronized, efficiency and billing driven routines leave little flexibility to adapt care processes to the needs, tempo and preferences of people with cognitive impairment; "my question is, what is the task for me" (p.8) : unclear goals and expectations for therapists in the new late shift and specialized unit mean they are unsure whether to prioritise care, therapy or both, which weakens their engagement; "we never agreed upon the question, how should we inform the persons involved" (p.9) : absence of a clear communication plan about who informs whom, when and about what leaves staff confused about roles, timelines and decisions and fragments implementation; "I'm basically questioning it" (p.8) : ongoing scepticism about concentrating patients with dementia and delirium in one unit, and doubts about risks and benefits, reduce commitment to the specialized unit model	Knowledge; Skills; Social/Professional Role and Identity; Beliefs about Consequences; Intentions; Goals; Environmental Context and Resources; Social Influences; Emotion; Behavioral Regulation	Doctors=concerns about concentrating dementia and delirium cases in one unit and preference for leaving roll out to individual initiative; Nurses=experience of staff shortages, emotional and physical burden, fear of falls and not doing justice to all patients, and strain from efficiency driven routines; AHPs=uncertainty about targets and tasks for the specialized unit and late shift, discomfort with terminology and nursing focused framing of interventions, and resistance to changing working hours; Managers=ambivalent engagement from hospital management who agreed to the specialized unit but did not consistently use their authority to support structural changes or prioritise implementation needs	Doctors=[Knowledge; Social/Professional Role and Identity; Beliefs about Consequences; Intentions]; Nurses=[Knowledge; Skills; Beliefs about Capabilities; Goals; Environmental Context and Resources; Social Influences; Emotion; Behavioral Regulation; Intentions]; AHPs=[Knowledge; Social/Professional Role and Identity; Beliefs about Consequences; Goals; Environmental Context and Resources]; Managers=[Environmental Context and Resources; Social Influences; Goals; Intentions]
Person-Centred Dementia Care in an Acute Hospital: Experiences from Nurses Working in a Specialized Dementia Unit	Cheong CY; Tay FHE; Choo J; Yap PLK	we don't really get exposure to dementia patients (N5, p.252) : limited dementia specific education leaves nurses unprepared for person centred dementia care and managing aggressive behaviour; "we cried a lot of times" (N4, p.252) : repeated exposure to violence and emotional strain makes it harder to cope and sustain PCC ethos; "there's language barrier, so it's quite hard" (N1, p.252) : communication gaps between nurses and patients trigger agitation and limit use of dementia care strategies; "nobody wants to be in CAMIE" (N1, p.253) : negative perceptions of dementia work among outside nurses hinder staffing and threaten sustainability of the unit; "we don't feel like coming to work anymore" (N5, p.253) : cumulative tiredness and emotional exhaustion reduce motivation to continue demanding PCC work	Knowledge; Skills; Beliefs about Capabilities; Emotion; Environmental Context and Resources; Social Influences; Intentions	Doctors=[NR]; Nurses=initial lack of dementia specific education, fear and emotional burden when managing aggression in a restraint free unit, language barriers with patients, stigma from outside nurses and worries about sustaining work and staffing; AHPs=[NR]; Managers=[NR]	Doctors=[NR]; Nurses=[Knowledge; Skills; Beliefs about Capabilities; Emotion; Environmental Context and Resources; Social Influences; Intentions]; AHPs=[NR]; Managers=[NR]
Prevention and management of behavioural and psychological symptoms in patients with dementia in acute care: a best practice implementation project	Rachid Akrour; Catherine Courret-Gilgen; Beatrice Perrenoud	too long and poorly structured: CMAI e-format impeded use (Environmental Context and Resources); "documentation remained suboptimal": EHR limits reduced fidelity (Environmental Context and Resources); "mental workload was a burden": workload cut engagement (Emotion; Beliefs about Capabilities); "lack of communication between the nursing staff members": coordination gaps (Social Influences); "practice monitoring … not … a priority": weak monitoring culture (Goals; Environmental Context and Resources); "felt uncomfortable caring for patients with agitation/aggressive behavior": confidence barrier (Beliefs about Capabilities; Emotion).	Knowledge; Skills; Beliefs about Capabilities; Emotion; Environmental Context and Resources; Social Influences; Goals; Behavioral Regulation	Doctors → NR; Nurses → mental workload; discomfort with agitation/aggression; documentation/EHR constraints; communication gaps; Managers → time pressure; reliance on head nurse to reach agreement; practice monitoring not prioritized; AHPs → NR	Doctors=[NR]; Nurses=[Knowledge; Skills; Beliefs about Capabilities; Emotion; Environmental Context and Resources; Social Influences; Behavioral Regulation]; AHPs=[NR]; Managers=[Environmental Context and Resources; Social Influences; Goals; Behavioral Regulation]
Understanding and identifying ways to improve hospital-based cancer care and treatment for people with dementia: an ethnographic study	Laura Ashley; Rachael Kelley; Alys Griffiths; Fiona Cowdell; Ann Henry; Hayley Inman; June Hennell; Margaret Ogden; Maria Walsh; Liz Jones; Ellen Mason; Michelle Collinson; Amanda Farrin; Claire Surr	"We don't have any revalidated training" → Limited dementia training (Knowledge); "It's about targets, targets, targets" → Service pressure limits flexibility (Environmental Context and Resources; Goals); "System would say… needs a two hour slot… we don't" → Inflexible scheduling capacity (Environmental Context and Resources); "Had to search records to establish diagnosis" → Poor visibility of dementia status (Environmental Context and Resources; Behavioral Regulation); "Memory nurse… didn't see it was their role" → Role ambiguity for support tasks (Social/Professional Role and Identity)	Knowledge; Goals; Environmental Context and Resources; Behavioral Regulation; Social/Professional Role and Identity	Doctors → Inflexible booking systems; lack of automatic dementia flags; limited capacity for extended slots; Nurses → Target-driven workflows limit person-centred adjustments; AHPs → Limited dementia-specific training (radiography); unclear ownership of escort/support roles (social work); Managers → NR	Doctors=[Environmental Context and Resources; Behavioral Regulation]; Nurses=[Goals; Environmental Context and Resources]; AHPs=[Knowledge; Social/Professional Role and Identity; Environmental Context and Resources]; Managers=[NR]
Implementing an intervention to enhance care delivery and consistency for people with hip fracture and cognitive impairment in acute hospital wards: a mixed methods process evaluation of a randomised controlled feasibility trial (PERFECTED)	Backhouse T; Fox C; Hammond SP; Poland F; McDermott-Thompson V; Penhale B; Cross JL	"when the nursing numbers have been so diminished any extra… is really resisted": Beliefs about Consequences/Environmental Context and Resources; "lack of engagement from the other staff": Social Influences; "staff are struggling to carry out their required duties": Environmental Context and Resources; "no-one has told us it has come from that": Knowledge	Knowledge; Beliefs about Consequences; Environmental Context and Resources; Social Influences; Goals; Behavioral Regulation	Doctors → "anything… extra… is really resisted"; Nurses → "no-one has told us it has come from that"; AHPs → "not a 100% clear about the different check lists"; Managers → "This leaves the ward unsafe… staff are struggling"	Doctors=[Beliefs about Consequences]; Nurses=[Knowledge; Environmental Context and Resources]; AHPs=[Knowledge]; Managers=[Environmental Context and Resources]
Implementation of STAR-VA for behavioral symptoms of dementia in acute care: Lessons learned	Hannah M. Bashian; Julia T. Boyle; Seneca Correa; Jane Driver; Caroline Madrigal; Isabel Desroches; Mackenzie Farrell; Olivia Eiten; Katie Flanagan; Talya Shahal; Kelly A. O'Malley	"managing BPSD"; "time required to care"; "emotional drain"; limited flexibility to change noisy environment; less time to know patients; need "more support" from other services	Knowledge; Skills; Environmental Context and Resources; Social Influences; Emotion; Behavioral Regulation	Doctors → [NR]; Nurses → managing BPSD; time pressure; emotional drain; need for de-escalation skills; AHPs → BC availability limited off-hours; Managers → sustaining cross-discipline communication and scheduling constraints	Doctors=[NR]; Nurses=[Knowledge; Skills; Environmental Context and Resources; Emotion; Behavioral Regulation]; AHPs=[Environmental Context and Resources]; Managers=[Environmental Context and Resources; Social Influences]
Dementia case-finding in hospitals: a qualitative study exploring the views of healthcare professionals in English primary care and secondary care	Anne-Marie Burn; Jane Fleming; Carol Brayne; Chris Fox; Frances Bunn	"ridiculous, a waste of time": specialty buy-in barrier; "missing mandatory fields": IT capture gaps; "no room for professional judgement": inflexible process; "don't put the yellow flag … no evidence of dementia": mislabelling risk	Environmental Context and Resources; Social Influences; Knowledge; Skills; Behavioral Regulation	Doctors → specialty non-compliance; risk of mislabelling in ED; unclear referral ownership; Nurses → documentation burden; variable tools/process; Managers → inflexible/fragmented IT systems; mandatory field glitches; AHPs → [NR]	Doctors=[Social Influences; Knowledge; Skills; Behavioral Regulation]; Nurses=[Environmental Context and Resources; Behavioral Regulation]; Managers=[Environmental Context and Resources; Behavioral Regulation]; AHPs=[NR]
An Action Research Approach to Implementing a Program for Sharing Meaningful Moments With Hospitalized Patients Living With Dementia	Esther Coker; Joanna Sue; Liz Mersereau; Patricia Hewston; Caroline Marr; Sophia Matarazzo; Afeef Vehra; Alexandra Papaioannou	"initial resistance" among staff: culture/acceptance; "set in their ways": habit barriers; "not knowing what or how to implement"; "documentation … rarely used"; "no dedicated EMR place to document"; "availability of resources" for activities; "time pressure during care"; "difficult to know patients when family uninvolved"; "hard to engage very severe dementia"; "unclear roles/assumptions"	Knowledge; Skills; Social/Professional Role and Identity; Beliefs about Capabilities; Beliefs about Consequences; Memory, Attention and Decision Processes; Environmental Context and Resources; Social Influences; Behavioral Regulation	Doctors →[NR]; Nurses=not sure what/how to implement; documentation friction; time pressure; AHPs=difficult to know person without family; engagement hard in very severe dementia; Managers=role clarity/accountability gaps; limited resources for activities	Doctors=[NR]; Nurses=[Knowledge; Behavioral Regulation; Memory, Attention and Decision Processes; Environmental Context and Resources]; AHPs=[Knowledge; Skills; Environmental Context and Resources; Social Influences]; Managers=[Social/Professional Role and Identity; Environmental Context and Resources]
Disjunctures in practice: ethnographic observations of orthopaedic ward practices in the care of older adults with hip fracture and presumed cognitive impairment	Jane L. Cross; Tamara Backhouse; Simon P. Hammond; Bridget Penhale; Fiona Scheibl; Nigel Lambert; Anna Varley; Chris Fox; Fiona M. Poland	Fast-track is a bit of a joke really.: Beliefs about Consequences; "We don't have staff numbers. We'll just have to cope.": Environmental Context/Emotion; "There is a sequence … but it doesn't always happen.": Behavioral Regulation/Goals; "I don't know where your paperwork is – it's a mystery.": Environmental Context; "am I giving both? … 12,500 units": Memory, Attention and Decision Processes; "I don't bother with all the forms as I should.": Behavioral Regulation	Environmental Context and Resources; Social/Professional Role and Identity; Memory, Attention and Decision Processes; Behavioral Regulation; Social Influences; Emotion; Beliefs about Consequences; Goals	Doctors → "Fast-track is a bit of a joke really"; limited response to personal care requests amid role demarcation; NR counts; Nurses → workload/understaffing; paperwork burden; fidelity gaps to care rounds; AHPs → role boundaries (physiotherapists initially assist then defer; pharmacist declines toileting help); paperwork/system frictions; Managers → bed manager overrides moving sleeping patients; ward manager notes staff shortages	Doctors=[Beliefs about Consequences; Social/Professional Role and Identity]; Nurses=[Environmental Context and Resources; Behavioral Regulation; Goals; Emotion]; AHPs=[Social/Professional Role and Identity; Environmental Context and Resources; Memory, Attention and Decision Processes]; Managers=[Environmental Context and Resources; Goals]
Patient safety for people experiencing advanced dementia in hospital: A video reflexive ethnography	Ann Dadich; Jade Rodrigues; Anita De Bellis; Annmarie Hosie; Tamsin Symonds; Justin Prendergast; Alan Bevan; Aileen Collier	"they don't pick it up until they hit someone else": delayed recognition of escalation cues among newer nurses; "When they wee, it doesn't happen on one square": hygiene/cleaning workload concern; "task-focused and rushing": general acute ward culture barrier; "gung-ho and all-hands-on-deck pin the person down": external security response misaligned; "balancing the requests of one patient… disruption to others": competing ward needs	Knowledge; Skills; Memory, Attention and Decision Processes; Environmental Context and Resources; Social/Professional Role and Identity; Social Influences; Goals	Doctors →[NR]; Nurses=delayed recognition of early behavioural cues among newer nurses; environmental hygiene concern; tension between individual preferences and ward needs; AHPs=[NR]; Managers=carpet trade-offs and monitoring; need to align security response; coordinating competing priorities	Doctors=[NR]; Nurses=[Knowledge; Memory, Attention and Decision Processes; Environmental Context and Resources; Goals; Social Influences]; AHPs=[NR]; Managers=[Environmental Context and Resources; Social/Professional Role and Identity; Social Influences; Goals]
Dementia, medication and transitions of care	Louise S. Deeks; Gabrielle M. Cooper; Brian Draper; Susan Kurrle; Diane M. Gibson	"they are actually really unsafe discharges" (hospital pharmacist); "the person who knows the patient least well is writing the instruction manual" (GP); "in other parts of the hospital there's less awareness of dementia" (hospital doctor); "it probably in reality never happens" (hospital pharmacist on updating summaries); "you've got to sort it" (OT on discharge meds)	Knowledge; Skills; Memory, Attention and Decision Processes; Environmental Context and Resources; Social/Professional Role and Identity; Social Influences; Behavioral Regulation; Goals	Doctors → limited dementia awareness outside geriatric wards; junior doctors drafting discharge meds lacking context; coordination with multiple prescribers; Nurses → time-pressured counseling; after-hours discharges; taking on medication roles due to pharmacy access limits; training gaps (esp. in aged-care interface); AHPs → pharmacist access restrictions; inability to amend discharge summaries; need for reconciliations; OT tasked with sorting discharge meds; Managers → NR	Doctors=[Knowledge; Social/Professional Role and Identity; Social Influences; Goals]; Nurses=[Environmental Context and Resources; Skills; Behavioral Regulation; Goals]; AHPs=[Environmental Context and Resources; Social/Professional Role and Identity; Behavioral Regulation; Memory, Attention and Decision Processes]; Managers=[NR]
Using sense-making theory to aid understanding of the recognition, assessment and management of pain in patients with dementia in acute hospital settings	Dawn Dowding; Valentina Lichtner; Nick Allcock; Michelle Briggs; Kirstin James; John Keady; Reena Lasrado; Elizabeth L. Sampson; Caroline Swarbrick; S. José Closs	"you're creating a picture… trying to build a picture" (staff nurse); "if a patient's got dementia then it's not really much use asking… pain scale" (doctor in training); "there's less focus on considering pain… [in some medical wards]" (consultant)	Knowledge; Skills; Memory, Attention and Decision Processes; Environmental Context and Resources; Social/Professional Role and Identity; Social Influences; Behavioral Regulation; Beliefs about Consequences; Goals	Doctors → underuse/distrust of tools; normalize aches in elderly; fragmented documentation; Nurses → reliance on intuition; time/routine constraints; non-use of validated tools; HCAs → limited authority to act beyond escalate; AHPs/Physios → pain only evident on mobilization; Managers → routine design may limit noticing points	Doctors=[Knowledge; Social/Professional Role and Identity; Beliefs about Consequences; Goals; Environmental Context]; Nurses=[Memory/Attention; Skills; Environmental Context; Behavioral Regulation; Goals]; HCAs=[Social/Professional Role and Identity]; Environmental Context; Behavioral Regulation]; AHPs=[Environmental Context; Knowledge; Skills; Social Influences]; Managers=[Environmental Context; Social Influences]

Title	Author(s)	Barrier quotations and granular barrier descriptions	TDF Domains – Barriers	Barriers specific to profession	Barriers per profession → TDF
Exploring dementia care in acute care settings: Perspectives of nurses and social workers on caring for patients with behavioral and psychological symptoms	Ruth E. Dunkle; Katherine Cavignini; Joonyoung Cho; Laura Sutherland; Helen Kales; Cathleen Connell; Amanda Leggett	we often don't have time: time/resource constraints; "lack of protected open space": environmental constraint; "I need an order for Haldol": reliance on medication under pressure; "staff has very little education for alternative communication methods": training/skill gap; "resources just aren't plentiful": discharge resource limitation; "we sometimes feel kind of stuck": placement dilemma	Environmental Context and Resources; Knowledge; Skills; Goals; Behavioral Regulation; Beliefs about Consequences; Social/Professional Role and Identity; Social Influences; Emotion	Doctors → [NR]; Nurses → reliance on meds under time pressure; supervision needs; lack of protected space; limited training in alternative communication; AHPs → coordination burden; discharge resource scarcity; need for family cooperation; time constraints; Managers → [NR]	Doctors=[NR]; Nurses=[Environmental Context and Resources; Knowledge; Skills; Goals; Behavioral Regulation; Beliefs about Consequences; Emotion]; AHPs=[Environmental Context and Resources; Social Influences; Goals; Knowledge]; Managers=[NR]
Using Signs and Symbols to Label Hospital Patients with a Dementia Diagnosis: Help or Hindrance to Care?	Katie Featherstone; Paula Boddington; Andy Northcott	"flowers will be left up for nondementia patients": signage persistence; "We have three feeds, can you help?": task pressure; "He won't eat all that": presumptive dependency; "Signs are often inaccurate": fidelity concern; "not enough red trays": resource mismatch; "Those that get the attention are the really disruptive ones": biased attention	Environmental Context and Resources; Memory, Attention and Decision Processes; Knowledge; Social/Professional Role and Identity; Beliefs about Consequences; Goals; Behavioral Regulation; Social Influences; Emotion	Doctors → concern about signs remaining after transfers; potential misclassification; Nurses → reliance on red trays and "feeding" assumptions; task/time pressures; Managers → emphasis on "dementia-friendly" image over practice; AHPs → identification inconsistencies across specialist roles	Doctors=[Environmental Context and Resources; Memory, Attention and Decision Processes]; Nurses=[Environmental Context and Resources; Goals; Beliefs about Consequences; Behavioral Regulation; Social/Professional Role and Identity]; AHPs=[Knowledge; Memory, Attention and Decision Processes]; Managers=[Goals; Social Influences; Environmental Context and Resources]
Developing a Life Story Intervention for Older Adults With Dementia or at Risk of Delirium Who Were Hospitalized: Multistage, Stakeholder-Engaged Co-Design Study	Sarah J. Flessa; James D. Harrison; Roniela Turnigan; Megan Rathfon; Michael Chandler; Jay Newton-Small; Stephanie E. Rogers	"don't have the time to sit down and know them": time pressure; "increasingly busy care environment": workload; short length of stay: feasibility; "story may not be a priority": competing demands; variable specialty buy-in: engagement; privacy concerns: data sharing risk	Environmental Context and Resources; Memory, Attention and Decision Processes; Goals; Social Influences; Social/Professional Role and Identity; Knowledge; Skills; Beliefs about Consequences; Emotion	Doctors=[NR]; Nurses=[NR]; AHPs=[NR]; Managers=[NR]	Doctors=[NR]; Nurses=[NR]; AHPs=[NR]; Managers=[NR]
Issues experienced while administering care to patients with dementia in acute care hospitals: A study based on focus group interviews	Risa Fukuda; Yasuko Shimizu; Natsuko Seto	Therefore, they deal with problems as they occur: reactive practice; Time to discuss the issues during conferences could not be secured: limited opportunity for shared learning; Because the nurse call button is not connected to a personal walkie talkie system: infrastructure gap; Doctors understanding of patients with dementia is limited, refuse psychiatric consultation: interprofessional barrier; Especially in staff shortage situations during the night: capacity constraint	Knowledge; Skills; Social/Professional Role and Identity; Beliefs about Consequences; Goals; Memory, Attention and Decision Processes; Environmental Context and Resources; Social Influences; Emotion; Behavioral Regulation	Doctors=[NR]; Nurses=staff shortage and time pressure, limited dementia training and experience, infrastructure gaps such as call system, difficulty obtaining psychiatric consult, reactive practice, fear of blame and liability, ethical tension about restraints and treatment; AHPs=[NR]; Managers=[NR]	Doctors=[NR]; Nurses=[Knowledge; Skills; Environmental Context and Resources; Social Influences; Goals; Memory, Attention and Decision Processes; Beliefs about Consequences; Emotion; Behavioral Regulation]; AHPs=[NR]; Managers=[NR]
Effects of a Person Centered Dementia Training Program in Greek Hospital Staff: Implementation and Evaluation	Mara Gkioka; Birgit Teichmann; Despina Moraitou; Sotirios Papagiannopoulos; Magda Tsolaki	lack of time during the shift : time pressure limited practice; "lack of staff members" : staffing shortages constrain PCC; " physical environment disturbances such as the illumination and high temperature of the room" : environment hindered implementation; "nursing continues to be dependent on the medical profession's authority" : constrained autonomy; " hierarchical culture focused on internal stability" : resistant culture; "bureaucracy" : organizational rigidity; "lack of guidance, leadership and clear procedures" : unclear protocols impede change	Environmental Context and Resources; Social Influences; Social/Professional Role and Identity; Beliefs about Consequences; Behavioral Regulation; Emotion	Doctors=[NR]; Nurses="lack of time during the shift; lack of staff members; physical environment disturbances; constrained autonomy under medical authority; doubts about implementing in Greek hospital reality"; AHPs=[NR]; Managers="bureaucracy; hierarchical culture; lack of guidance leadership and clear procedures"	Doctors=[NR]; Nurses=[Environmental Context and Resources; Social/Professional Role and Identity; Social Influences; Beliefs about Consequences; Behavioral Regulation]; AHPs=[NR]; Managers=[Social Influences; Behavioral Regulation; Goals; Environmental Context and Resources]
The Person, Interactions and Environment Programme to improve care of people with dementia in hospital: a multisite study	Godfrey M; Young J; Shannon R; Skingley A; Woolley R; Arrojo F; Brooker D; Manley K; Surr C	"there was neither the time nor the headroom to do more than get through the daily routine"; "absence of drivers" to pursue change; "ward closures and reorganisation" disrupted planning; "winter pressures beds" reopened, increasing demand	Environmental Context and Resources; Social Influences; Goals; Behavioral Regulation; Knowledge	Doctors → NR; Nurses → time pressure, staffing instability, ward moves; AHPs → peripheral to leadership reduced leverage; Managers → competing operational demands limit facilitation	Doctors=[NR]; Nurses=[Environmental Context and Resources; Goals; Social Influences; Behavioral Regulation]; AHPs=[Social Influences; Environmental Context and Resources]; Managers=[Social Influences; Environmental Context and Resources; Goals]
How do emergency department staff respond to behaviour that challenges displayed by people living with dementia? A mixed-methods study	Goodwin L, Liddiard C, Manning S, Benger JR, Carlton E, Cheston R, Hoskins R, Taylor H, Voss S	everything in ED plays against dementia p.? P011: Environmental overload and unmet needs; "we do not have a dedicated member of staff to stay with them" P007: Staffing shortfall limits 1:1 care; "only supposed to be there for four hours... lack of beds" P006: Flow and bed pressure sustain exposure to triggers; "you just have to improvise with what you have got" P011: Lack of protocol leads to ad hoc practice; "another piece of paperwork to fill out" P012: Paperwork burden reduces uptake	Environmental Context and Resources; Knowledge; Skills; Goals; Memory, Attention and Decision Processes; Social Influences; Emotion; Behavioral Regulation	Doctors=[NR]; Nurses=[NR]; AHPs=[NR]; Managers=[NR]	Doctors=[NR]; Nurses=[NR]; AHPs=[NR]; Managers=[NR]
Balancing the needs of individuals and services in cancer treatment for people with dementia: A focused ethnographic study	Alys Wyn Griffiths; Laura Ashley; Rachael Kelley; Fiona Cowdell; Michelle Collinson; Ellen Mason; Hayley Inman; Ann Henry; Amanda Farrin; Claire Surr	How do you examine somebody if they're not able to give consent to that?: consent and agitation challenge, Nurses; " you could cause more upset than good. It's difficult to gauge": communication risk, AHPs; "it's that kind of awkward conversation": uncertainty adapting talk, AHPs; "they should go back to where they originated from as soon as possible": defaulting elsewhere, Nurses; "Sometimes... just getting them through treatment, not dealing with the reasons": process focus over needs, AHPs; "why wait to the point where they get to treatment?": reactive not proactive, AHPs; "with the resources in the NHS at the moment, that gets harder and harder": time and staffing constraints, Nurses; "logistical nightmare": scheduling and targets pressure, AHPs; "Some will just do as they're told": infantilizing attitudes, Staff; "We have to make time. It's very challenging": limited capacity to individualize, Nurses	Environmental Context and Resources; Behavioral Regulation; Memory, Attention and Decision Processes; Skills; Social/Professional Role and Identity; Social Influences; Goals; Beliefs about Consequences; Emotion	Doctors=[NR]; Nurses=consent when agitated is challenging; time and target pressures; reactive adjustments; AHPs=communication hard to gauge; process focus; logistical and ID constraints; continuity hard; Managers=[NR]	Doctors=[NR]; Nurses=[Environmental Context and Resources; Skills; Behavioral Regulation; Goals; Beliefs about Consequences]; AHPs=[Environmental Context and Resources; Behavioral Regulation; Memory, Attention and Decision Processes; Skills; Goals]; Managers=[NR]
Supporting general hospital staff to provide dementia sensitive care: A realist evaluation	Melanie Handley; Frances Bunn; Claire Goodman	"We don't know anything about her and all we see is this person constantly calling out" : lack of patient biography limits responses; "There is no way to show that we are giving good care" : weak feedback metrics hinder practice change; "Who is this person on the ward with a note pad" : low credibility of distant experts; "The nurses are put upon" : workload pressure limits dementia care; "I don't think it is a skill, you just ensure that you don't let them fall" : dementia care devalued; "She is going to put the bedrails up for safety" : risk aversion overrides preferences; "Trying to keep all the patients in their bed" : restrictive routines increase agitation.	Knowledge; Skills; Social/Professional Role and Identity; Beliefs about Consequences; Goals; Environmental Context and Resources; Social Influences; Behavioral Regulation	Nurses → workload pressure, restrictive routines, inconsistent documentation, risk-averse practices; Doctors → NR; AHPs → NR; Managers → NR	Doctors=[NR]; Nurses=[Knowledge; Goals; Environmental Context and Resources; Beliefs about Consequences; Social/Professional Role and Identity; Behavioral Regulation]; AHPs=[NR]; Managers=[NR]
Exploring ways to enhance pain management for older people with dementia in acute care settings using a Participatory Action Research approach	Deirdre Harkin; Vivien Coates; Donna Brown	"We don't do well looking at other things around that" : acute focus crowds out person context; "They move on so quickly" : short stays hinder knowing the patient; "They are not able to vocalise and...they get forgot about" : non verbal pain overlooked; "Targets driving practice" : task culture deprioritises pain; "Serious staff shortages" : time pressure barrier; "Tick box and policy driven culture" : procedures over person; "Not joining the dots" : fragmented information blocks decisions.	Knowledge; Skills; Goals; Environmental Context and Resources; Memory, Attention and Decision Processes; Social/Professional Role and Identity; Behavioral Regulation	Nurses → target and task culture, staff shortages and time pressure, uncertainty assessing non verbal pain, fragmented information; Doctors → junior prescribing decisions defaulting to PRN, limited senior input; AHPs → uncertainty scoring pain, missed opportunities to integrate family input; Managers → policy or target emphasis over person-centred priorities	Doctors=[Beliefs about Consequences; Environmental Context and Resources]; Nurses=[Knowledge; Environmental Context and Resources; Goals; Memory, Attention and Decision Processes; Behavioral Regulation]; AHPs=[Knowledge; Memory, Attention and Decision Processes]; Managers=[Goals; Environmental Context and Resources]
Barriers and enablers to implementing 'DEALTS2' simulation-based train-the-trainer dementia training programme in hospital settings across England: a qualitative study	Michelle Heward; Michele Board; Ashley Spriggs; Dina Blagden; Jane Murphy	"I had to wait for management to say that we could go ahead with the training" : lack of managerial approval delays implementation; "Tricky to find time to fit it in... only 1.5 hours" : insufficient time allocation undermines delivery; "It is hard to get staff off the ward to train them" : workload and release barriers; "As I did not have the full background of the staff... unsure of what they were expecting" : audience fit uncertainty; "Only covers 3 areas" : scope misalignment with local expectations; "Moving away from face-to-face" : delivery model mismatch.	Environmental Context and Resources; Goals; Social Influences; Knowledge; Skills; Beliefs about Capabilities; Behavioral Regulation	Doctors → NR; Nurses → NR; AHPs → NR; Managers → approval delays, non-mandatory status deprioritises dementia training, insufficient time and resources	Doctors=[NR]; Nurses=[NR]; AHPs=[NR]; Managers=[Environmental Context and Resources; Goals; Social Influences]
Person-centred oral hydration care for older people with dementia admitted to acute hospital wards: Empirical research qualitative	Shanlee Higgins; Lesley Baillie; Calvin Moorley; Fiona Nolan	We have fluid balance charts but a lot of the time they're not filled in accurately : Memory, Attention and Decision Processes; "before, ward sisters could get into their own kitchen... it's a private firm" : Environmental Context and Resources; "Put on the back burner" : Goals; "They do not have any responsibility" [re: hosts reported by staff] : Social Influences and Environmental Context and Resources; "most staff–patient interactions related to physical care, hydration was less evident" : Goals and Environmental Context and Resources	Knowledge; Skills; Goals; Memory, Attention and Decision Processes; Environmental Context and Resources; Social Influences; Behavioral Regulation; Beliefs about Consequences	Doctors → minimal interaction, focus on acute risks and documentation only; Nurses → charts inaccurate, hydration secondary to other tasks, rely on routines; AHPs → unclear recording, advise rather than provide, hydration secondary to therapy aims; Managers → kitchen access removed, policy and metrics emphasise other priorities	Doctors=[Beliefs about Consequences; Environmental Context and Resources; Social/Professional Role and Identity]; Nurses=[Memory, Attention and Decision Processes; Goals; Behavioral Regulation; Environmental Context and Resources; Social Influences]; AHPs=[Social/Professional Role and Identity; Environmental Context and Resources]; Managers=[Environmental Context and Resources; Goals; Social Influences]
The experience of hospital staff in applying the Gentle Persuasive Approaches to dementia care	Lillian Hung; Cathy Son; Rebecca Hung	Everything is a matter of time : time pressure; "If you don't have time, you can't do it" : workload constraint; "I can't get my work done" : competing demands; "Time, so that we're not so pushed in terms of rushing a task" : rushing tasks; " Before the GPA, we didn't know what to do" : knowledge gap; "Once management understands... what our needs are" : leadership understanding gap	Environmental Context and Resources; Goals; Knowledge; Social Influences; Behavioral Regulation	Doctors=[NR]; Nurses= time constraints, rushing tasks, competing demands, need leadership support; AHPs=[NR]; Managers=[NR]	Doctors=[NR]; Nurses=[Environmental Context and Resources; Goals; Behavioral Regulation; Social Influences]; AHPs=[NR]; Managers=[NR]
Nurses' knowledge and beliefs on pain management practices with hospitalised persons living with dementia: A qualitative descriptive study	Beverly Ingelson; Sherry Dahlke; Hannah O'Rourke; Kathleen F. Hunter	It is twice as much as time because you have to observe them : time burden; "Sometimes numerically isn't possible to do" : self report limits; "Older patients with dementia are under medicated" : undertreatment; "They are afraid of giving them pain medication because they're afraid of respiratory distress and more confusion" : safety concerns; "It's hard to have frequent monitoring when you have three or four other patients" : staffing; "Give them medication to calm down before you can do the assessment" : antipsychotic first; "It may not be forefront in my mind" : competing priorities; "I don't think there's enough education" : knowledge gap	Knowledge; Skills; Beliefs about Consequences; Environmental Context and Resources; Goals; Memory, Attention and Decision Processes; Behavioral Regulation; Social Influences	Doctors=[NR]; Nurses= time burden; self report limits; safety concerns about analgesics; staffing limits; antipsychotic first; competing priorities; education gaps; AHPs=[NR]; Managers=[NR]	Doctors=[NR]; Nurses=[Environmental Context and Resources; Knowledge; Skills; Beliefs about Consequences; Goals; Memory, Attention and Decision Processes; Behavioral Regulation; Social Influences]; AHPs=[NR]; Managers=[NR]
Living with dementia in hospital wards: a comparative study of staff perceptions of practice and observed patient experience	Innes A., Kelly F., Scerri C., Abela S.	tasks continued to be generalised and rushed : task-first ward culture and time pressure; "patients continued to be disempowered or ignored" : limited reflective practice and low awareness; "routine use of incontinence pads" : workflow convenience over continence support; "inappropriate use of restraint continued" : risk-avoidance norms; "insufficient positive interaction between staff and patients" : competing priorities reduce engagement; "insufficient staff able to communicate" : staffing mix and language barriers; "ward sizes remained larger than recommended" : large ward layout stretches attention; "very loud buzzer systems" : noisy environment disrupts care	Environmental Context and Resources; Knowledge; Skills; Goals; Social Influences; Beliefs about Consequences; Behavioral Regulation	Doctors=[NR]; Nurses=[NR]; AHPs=[NR]; Managers=[NR]	Doctors=[NR]; Nurses=[NR]; AHPs=[NR]; Managers=[NR]

Title	Author(s)	Barrier quotations and granular barrier descriptions	TDF Domains – Barriers	Barriers specific to profession	Barriers per profession → TDF
Barriers and facilitators to caring for patients living with dementia in the emergency department: a qualitative study	Jelinski D., Reich K., Lang E., Holroyd-Leduc J., Goodarzi Z.	it's an environment that's not well set up for them. : overstimulating ED environment and layout; "lack of resources, lack of staff" : resource constraints and understaffing; "we don't always kind of have the best approach" : limited dementia-specific guidance and skills; "it's always hard to get a little bit of the buy in from the collective" : variable team norms and buy-in; "not all older adults that come in confused have dementia." : diagnostic uncertainty and knowledge gaps	Environmental Context and Resources; Skills; Knowledge; Social/Professional Role and Identity; Social Influences	Doctors → differentiating delirium and dementia skills vary; Nurses → time pressure and staffing hinder person-centred care; AHPs → buy-in and sustainability of roles are limited; Managers → NR	Doctors=[Knowledge; Skills]; Nurses=[Environmental Context and Resources; Goals; Social Influences]; AHPs=[Social/Professional Role and Identity; Social Influences; Environmental Context and Resources]; Managers=[NR]
Nurses' experiences of delivering acute orthopaedic care to patients with dementia	Jensen AM; Pedersen BD; Wilson RL; Olsen RB; Hounsgaard L	information drowning in the electronic patient record : fragmented information and poor retrieval; "The priority is on giving the patient the right medication" : task-first ward culture prioritises somatic goals; "We stress them with all our questions" : communication approach mismatched to cognitive capacity; "Oh no, I hope it is not one of them that calls out" : negative preconceptions shape allocation and effort; "Sometimes I get frustrated as they are so unpredictable" : emotional strain under uncertainty; "what if the patient catches pneumonia" : fear of consequences when varying pathways; "It is not ideal, and I feel both for the patient, and my colleagues" : allocation by personality under constraints	Knowledge; Skills; Goals; Memory, Attention and Decision Processes; Environmental Context and Resources; Social Influences; Emotion; Beliefs about Consequences; Behavioral Regulation	Doctors=[NR]; Nurses=["information drowning in the electronic record"; somatic priority over psychosocial needs; busy noisy ward; negative preconceptions and uncertainty; fear of adverse outcomes when varying pathways]; AHPs=[NR]; Managers=[allocation by personality under constraints; limited suitable rooms; standard pathways inflexible]	Doctors=[NR]; Nurses=[Knowledge; Skills; Goals; Memory, Attention and Decision Processes; Environmental Context and Resources; Social Influences; Emotion; Beliefs about Consequences; Behavioral Regulation]; AHPs=[NR]; Managers=[Environmental Context and Resources; Social Influences; Goals; Behavioral Regulation]
Health professional perspectives on systems failures in transitional care for patients with dementia and their carers: a qualitative descriptive study	Kable A; Chenoweth L; Pond D; Hullick C	push, push, push : discharge targets drive early discharge; "people doing the discharge summaries are the ones that have the least comprehensive overview" : limited situational awareness in documentation; "we don't have guidelines for discharge planning of PWD" : lack of guidance; "getting services is becoming really hard" : service scarcity and restrictive criteria; "notes aren't always amazing" : fragmented information; "we rarely sit down and explain the plan" : insufficient discharge conversation time; "we've never once received a Webster pack" : missing dose aids; "should the discharge summary be written by the most inexperienced person" : role misalignment	Knowledge; Skills; Memory, Attention and Decision Processes; Goals; Environmental Context and Resources; Social Influences; Beliefs about Consequences; Behavioral Regulation; Emotion	Doctors → limited overview when writing summaries; lack of dementia-specific guidance; med reconciliation uncertainty; discharge pressure; Nurses → time pressure, incomplete cross-discipline summaries, difficulty securing services; AHPs → access criteria and long waits for rehab and community services; Managers → discharge targets and late-after-hours transfer expectations	Doctors=[Knowledge; Memory, Attention and Decision Processes; Goals; Environmental Context and Resources; Beliefs about Consequences]; Nurses=[Environmental Context and Resources; Memory, Attention and Decision Processes; Goals; Behavioral Regulation]; AHPs=[Environmental Context and Resources; Goals]; Managers=[Goals; Social Influences; Environmental Context and Resources]
Nurses' perceptions in caring for people with dementia in Dutch acute hospitals	Annette Keuning-Plantinga; Petrie F. Roodbol; Wim P. Krijnen; Evelyn J. Finnema	time is often too short for the other duties : time pressure and workload; "Our department is not suitable for patients with dementia" : ward suitability and workflow constraints; "not always used in a timely and effective matter" : skills gap in restrictive measure alternatives; "they do not know how the bell works" : environmental cues and wayfinding limits	Knowledge; Skills; Environmental Context and Resources; Goals	Doctors → NR; Nurses → time pressure, ward not suitable, limited training on alternatives, reliance on restrictive measures; AHPs → NR; Managers → NR	Doctors=[NR]; Nurses=[Knowledge; Skills; Environmental Context and Resources; Goals]; AHPs=[NR]; Managers=[NR]
Radiographers' experiences in working with patients living with dementia in Norway: A qualitative study	E. Kjelle; C. Eikefet; C.C. Chilanga	The referrer must write if the patient is living with dementia : incomplete referral information; "There are generally no protocols to follow" : absence of departmental procedures; "as we close at three, the patient has not yet been picked up" : transport and scheduling logistics; "They don't always remember if they have a stent or a hip implant" : safety information reliability; "after 3 seconds they start, they don't remember or don't understand" : communication barriers under time pressure	Knowledge; Skills; Environmental Context and Resources; Behavioral Regulation; Goals	Doctors → NR; Nurses → NR; AHPs → missing dementia flags in referrals, no protocols, acute time pressure, safety screening difficult, transport logistics; Managers → NR	Doctors=[NR]; Nurses=[NR]; AHPs=[Knowledge; Skills; Environmental Context and Resources; Behavioral Regulation; Goals]; Managers=[NR]
Perioperative Nurses' Experience of Working With Persons Living With Dementia	Mariya Kovaleva; Abigail C. Jones; Cornelia Frances Garrard; Jana Lauderdale; Mary S. Dietrich; Peace Johnson; Cathy Maxwell	They definitely require more care : time pressure and workload; "helping keep the patient safe with dementia" : continuous monitoring burden; "We reorient these patients constantly" : limited effectiveness of reorientation routines; " PHYSICALLY HARM THEMSELVES, pulling at leads or their wires" : risk management habits trigger restraint use; "the longer they go without getting to sleep, the worse the symptoms get" : environmental sleep disruption	Knowledge; Skills; Environmental Context and Resources; Behavioral Regulation; Goals	Doctors → NR; Nurses → time-consuming workload, communication barriers, safety risks with lines and monitoring, sleep disruption, reliance on sitters and restraints; AHPs → NR; Managers → NR	Doctors=[NR]; Nurses=[Knowledge; Skills; Environmental Context and Resources; Behavioral Regulation; Goals]; AHPs=[NR]; Managers=[NR]
Implementation of a model of care for hospitalised older persons with cognitive impairment (the Confused Hospitalised Older Persons program) in six New South Wales hospitals	Susan Kurrle; Sigrid Patterson; Cath Bateman; Anthea Temple; Anne Cumming; Glen Pang	competing program priorities : competing priorities constrain focus; "project team member attrition" : staffing continuity limits momentum; "poor engagement by some staff" : variable buy-in; "time demands for project lead" : limited lead capacity; "low profile of delirium and dementia" : awareness and prioritisation gap; "information technology system changes" : workflow disruption	Knowledge; Skills; Environmental Context and Resources; Social Influences; Goals; Memory, Attention and Decision Processes	Doctors → NR; Nurses → NR; AHPs → NR; Managers → NR	Doctors=[NR]; Nurses=[NR]; AHPs=[NR]; Managers=[NR]
Redesigning acute care for cognitively impaired older adults: Optimizing health care services	Michael A LaMantia; Malaz A Boustani; Shola Jhanji; Mungai Maina; Arif Nazir; Frank C Messina; Amie Frame; Catherine Alder; Joshua Chodosh	complete disconnect between outpatient and inpatient care : fragmented cross-setting coordination, p.920; "maybe it was already done, and I did not know it" : duplicated referrals from poor visibility, p.921; "Somebody in the hospital told them to throw all their medicines away" : conflicting instructions create clinic reconciliation burden, p.921; "We are getting stretched, as far as our resources" : clinic workload and resource limits, p.919	Environmental Context and Resources; Social Influences; Knowledge; Memory, Attention and Decision Processes; Emotion	Doctors → "complete disconnect between outpatient and inpatient care"; "Somebody in the hospital told them to throw all their medicines away" ; Nurses → "We are getting stretched, as far as our resources"; "Everything starts with education" ; AHPs → "maybe it was already done, and I did not know it"; "we help with transportation and figure out their insurance" ; Managers → NR	Doctors=[Environmental Context and Resources; Social Influences; Memory, Attention and Decision Processes]; Nurses=[Environmental Context and Resources; Knowledge; Emotion]; AHPs=[Environmental Context and Resources; Memory, Attention and Decision Processes; Social Influences]; Managers=[NR]
Dementia care across a tertiary-care health system: What exists now and what needs to change	Amanda Leggett; Cathleen Connell; Leslie Dubin; Ruth Dunkle; Kenneth M. Langa; Donovan T. Maust; J. Scott Roberts; Beth Spencer; Helen C. Kales	exceptionally unfriendly place for anybody with dementia : large, noisy, disorienting ED environment; "dementia not being identified on the medical record" : missing diagnostic flags impede care; "we often do not have time" : competing demands and time pressure; "pulling the trigger on using antipsychotics quickly" : quick-fix habit under pressure; "hot potatoes" and "there's no place to send them" : disposition pathways unclear and constrained	Environmental Context and Resources; Knowledge; Social/Professional Role and Identity; Goals; Emotion; Memory, Attention and Decision Processes; Behavioral Regulation; Beliefs about Consequences	Doctors=[NR]; Nurses=[NR]; AHPs=[NR]; Managers=[NR]	Doctors=[NR]; Nurses=[NR]; AHPs=[NR]; Managers=[NR]
Understanding and integrating the needs and preferences of people living with dementia in the inpatient setting: a qualitative study	Alyse Lennox; Denise Goodwin; Felicity Leavold; Renae Nicol; Velandai Srikanth; Darshini Ayton; Madeleine Berends; Myra Thiessen; Daphne Flynn; Chris Moran	there's no specific teaching : limited formal dementia care training, Table 1; "we just don't have that time" : time pressure and workload, Table 1; "responsibility is on everyone, then it gets murky" : unclear ownership for gathering needs, Table 1; "current tools are surface-level" : prompts lack actionable strategies, Table 1; "people don't read them, they don't know they're there" : documentation buried and not used, Table 1	Knowledge; Skills; Environmental Context and Resources; Social/Professional Role and Identity; Memory, Attention and Decision Processes; Behavioral Regulation	Doctors → training gaps for practical dementia care; behaviour plans helpful but impractical to deploy routinely; buried in notes; Nurses → time pressure limits elicitation and documentation; unclear responsibility; reliance on reactive tools; AHPs → need shared, accessible tool across staff; online training available; Managers → NR	Doctors=[Knowledge; Environmental Context and Resources; Memory, Attention and Decision Processes]; Nurses=[Environmental Context and Resources; Behavioral Regulation; Social/Professional Role and Identity; Knowledge]; AHPs=[Knowledge; Environmental Context and Resources; Memory, Attention and Decision Processes]; Managers=[NR]
The assessment and management of pain in patients with dementia in hospital settings: a multi-case exploratory study from a decision making perspective	Valentina Lichtner; Dawn Dowding; Nick Allcock; John Keady; Elizabeth L. Sampson; Michelle Briggs; Anne Corbett; Kirstin James; Reena Lasrado; Caroline Swarbrick; S. José Closs	there's not enough of us, and we just haven't that time : Time pressure limits assessment and reassessment; "I'm only seeing the patients for a few minutes" : Brief doctor encounters constrain cue gathering; "paper... quite siloed risk assessments" : Documentation fragmentation across forms; "it's just seen as a form and a tick box exercise" : Procedural completion without clinical integration; "patients shouldn't go a long period without... reassessed" : Recommended timings unmet under workload; "not well used on the ward" : Unclear criteria reduce tool adoption; "I would assume... if there's nothing written" : Absence of documentation misread as absence of pain; "I don't have the time... to ferret out the information" : Limited time for diagnostic information seeking; "PRN... could not be used effectively" : Workflow reliance on patient-initiated requests; "nurses have so many assessments now" : Competing paperwork dilutes attention to pain cues	Environmental Context and Resources; Memory, Attention and Decision Processes; Behavioral Regulation; Goals; Knowledge; Skills; Social/Professional Role and Identity; Beliefs about Consequences	Doctors → brief patient contact windows, focus on investigations and organising tasks, assumption that no entry means no pain, limited time to elicit relevant history, reliance on routine pharmacology and PRN systems; Nurses → workload, competing documentation, tool use as tick-box, reliance on routines and brief interactions; AHPs → limited involvement in pain assessment on observed wards; Managers → fragmented forms and parallel paperwork noted, unclear criteria for tool use	Doctors=[Environmental Context and Resources; Memory, Attention and Decision Processes; Behavioral Regulation; Beliefs about Consequences; Goals]; Nurses=[Environmental Context and Resources; Behavioral Regulation; Memory, Attention and Decision Processes; Goals]; AHPs=[NR]; Managers=[Environmental Context and Resources; Behavioral Regulation]
What influences decisions about ongoing stroke rehabilitation for patients with pre-existing dementia or cognitive impairment: a qualitative study	Longley V; Bowen A; Peters S; Swarbrick C	have to make a decision pretty quickly (p.1138) : short timescales drive early judgments; "I wouldn't expect [them] to change" (p.1137) : low expectations reduce adoption; "it definitely takes more time" (p.1138) : additional time needed conflicts with capacity; "we're a service that's very much based around potential to improve" (p.1138) : improvement-first service model constrains options; "do you think they've got dementia?" (p.1139) : role ambiguity limits assessment confidence	Knowledge; Skills; Environmental Context and Resources; Social/Professional Role and Identity; Beliefs about Consequences; Goals; Social Influences	Doctors → quick discharge judgments under improvement framing; Nurses → staffing pressure and prioritization reduce rehab time; AHPs → difficulty predicting potential, reliance on gut instinct, limited dementia training; Managers → [NR]	Doctors=[Beliefs about Consequences; Goals]; Nurses=[Environmental Context and Resources; Goals]; AHPs=[Knowledge; Skills; Environmental Context and Resources; Social Influences]; Managers=[NR]
Facilitators, Barriers, and Solutions in Pain Management for Older Adults with Dementia	Alícia Minaya-Freire; Anna Ramon-Aribau; Gemma Pou-Pujol; Meritxell Fajula-Bonet; Mireia Subirana-Casacuberta	I think the conversation doesn't last more than 3 minutes : lack of time constrains assessment (p.498); "You don't have enough time to be able to see the patients and then be able to record everything" : time pressure and documentation burden (p.498); "We don't give it the importance we give to blood pressure" : pain not treated as fifth constant in records (p.498); "it's difficult for me to ask the question about pain and to be answered correctly" : cognitive impairment limits direct questioning (p.498); "if they're in a nursing home, the family doesn't want to know anything" : institutionalization reduces family collaboration (p.498–499); "In dementia we are light years away" : knowledge and skills gap (p.499); "We won't give the acetaminophen" : uncertainty about coadjuvant use reflects pharmacology knowledge gap (p.499); "I've never been to a meeting at night" : shift structure limits multidisciplinary coordination (p.499); "lack of communication interferes in this teamwork" : team communication barriers reduce coordination (p.499)	Knowledge; Skills; Environmental Context and Resources; Social Influences; Goals; Beliefs about Consequences; Memory, Attention and Decision Processes	Doctors=[NR]; Nurses=limited time and workload; under-recording; pain not treated as fifth constant; gaps in dementia and pharmacology knowledge; variable team communication by shift; difficulty directly questioning due to cognitive impairment; reduced family collaboration when institutionalized; AHPs=[NR]; Managers=[NR]	Doctors=[NR]; Nurses=[Knowledge; Skills; Environmental Context and Resources; Social Influences; Goals; Beliefs about Consequences; Memory, Attention and Decision Processes]; AHPs=[NR]; Managers=[NR]
Privileges, and Permissions: Theorising Intersectionality and Cultures of Control in the Care of People Living With Dementia in Acute Hospital Settings	Mwale S; Northcott A; Featherstone K	immediately called him back into his room : containment response when a patient with dementia walks away from bedside, p.5; "security guard shadowed him, arms folded" : security-based surveillance replaces therapeutic engagement, p.5; "one-to-one HCA shadowed at a distance" : differential supervision allows another patient to roam, p.5; "reading a patient folder at the nurses' desk" : privileged rule-bending permitted under supervision, p.6; "the chair alarm blared when she stood" : alarm device enforces bedside containment for walking attempts, p.8; "I can't leave you alone" : directive communication used to stop movement, p.8; "turn around" : command-style instruction to restrict walking, p.8; "labelled 'aggressive' and a 'big man'" : racialised categorisation heightens control measures, p.5	Social/Professional Role and Identity; Beliefs about Consequences; Goals; Environmental Context and Resources; Social Influences; Memory, Attention and Decision Processes; Emotion; Behavioral Regulation	Nurses → application of one-to-one observation, chair alarms, directive communication, containment; Doctors → [NR]; AHPs → [NR] for barriers; Managers → local rules/bedside routines emphasised (NR for specifics)	Doctors=[NR]; Nurses=[Social/Professional Role and Identity; Beliefs about Consequences; Goals; Environmental Context and Resources; Social Influences; Memory, Attention and Decision Processes; Emotion; Behavioral Regulation]; AHPs=[NR]; Managers=[Environmental Context and Resources; Goals]

Title	Author(s)	Barrier quotations and granular barrier descriptions	TDF Domains – Barriers	Barriers specific to profession	Barriers per profession → TDF
When people living with dementia say 'no': Negotiating refusal in the acute hospital setting	O'Brien R; Beeke S; Pilnick A; Goldberg SE; Harwood RH	I was just wondering if... (Findings, 3.5, Extract 5): Low-entitlement permission-seeking during nursing care invites refusal; "Is it okay if I listen to your chest?" (Findings, 3.5, Extract 6): Permission-seeking during doctor exam frames optionality; "Would you like another spoonful?" (Findings, 3.5, Extract 7): Question format highlights willingness contingency during feeding; "Can you take a deep breath?" (Findings, 3.5, Extract 8): Ability-framed respiratory request opens refusal space	Skills; Social/Professional Role and Identity; Beliefs about Consequences; Memory, Attention and Decision Processes; Behavioral Regulation	Doctors → "Is it okay if I listen to your chest?" highlights optionality; "Can you take a deep breath?" ability-framed request invites refusal; Nurses → "I was just wondering if..." low entitlement invites refusal; "Would you like another spoonful?" willingness contingency; AHPs → "Can you stand for me?" ability-framed request invites refusal; Managers → NR	Doctors=[Skills; Social/Professional Role and Identity; Beliefs about Consequences; Memory, Attention and Decision Processes; Behavioral Regulation]; Nurses=[Skills; Social/Professional Role and Identity; Beliefs about Consequences; Memory, Attention and Decision Processes; Behavioral Regulation]; AHPs=[Skills; Social/Professional Role and Identity; Beliefs about Consequences; Memory, Attention and Decision Processes; Behavioral Regulation]; Managers=[NR]
"Getting to Know Them": Person-Centered Care for Patients With Dementia in Acute Care	Osuoha P; Masoud SS; Leibas M; Cleveland LM; Reed CC; Piernik-Yoder B; White CL	"We just don't have enough hours in the day" : time pressure and task-first routines constrain PCC, p.39; "Hand-off is important" : fragmented information requires stronger handoff routines, p.39; "Sometimes when the medicine team is not on board" : interprofessional misalignment undermines consistent plans, p.39; "You have other patients you won't be able to reach" : staffing ratios and competing priorities limit attention, p.40; "They're in unfamiliar territory… unfamiliar faces" : unfamiliar ward environment elevates fall-risk management workload, p.40; "There's never one on dementia or Alzheimer's" : limited dementia-specific training leaves knowledge gaps, p.41	Knowledge; Skills; Memory, Attention and Decision Processes; Environmental Context and Resources; Social Influences; Goals	Doctors → NR; Nurses → time pressure and task-first routines; fragmented handoff/plan; interprofessional misalignment; staffing ratios and fall-risk workload; limited dementia-specific training; AHPs → NR; Managers → NR	Doctors=[NR]; Nurses=[Knowledge; Skills; Memory, Attention and Decision Processes; Environmental Context and Resources; Social Influences; Goals]; AHPs=[NR]; Managers=[NR]
Improving emotional wellbeing for hospital-based patients with dementia	Petty, S., Griffiths, A., Coleston, D.M. & Dening, T.	you can't just say 'Right this is a great way' (p.6, Interpersonal factors, tailoring) : need for individual tailoring increases decision complexity; "I can't possibly fit that in, I'm too busy" (p.10, Service design, competing tasks) : time pressure and workload; "busy, undignified and regimented" (p.12, Ward context) : high-stimulation environment; "hard to calm when distress is high" (p.6–7, Interpersonal factors) : escalation reduces effectiveness of responses	Environmental Context and Resources; Emotion; Goals; Knowledge	Doctors → NR; Nurses → time pressure and competing tasks, emotional toll, overstimulating environment, need for person knowledge; AHPs → NR; Managers → NR	Doctors=[NR]; Nurses=[Environmental Context and Resources; Emotion; Goals; Knowledge]; AHPs=[NR]; Managers=[NR]
Nurses' perspectives on shared decision-making in the daily care of hospitalized patients with dementia: An exploratory qualitative study	Plantinga A; Roodbol PF; van Munster BC; Finnema EJ	We don't ask about goals... this is the goal we have set (p.4659, multidisciplinary SDM context) : physician-led framing crowds out goal-talk; "making decisions together... often unconscious and part of my work routine" (p.4660, monodisciplinary care moments) : SDM steps not explicit; "patients are very inconsistent in their decision-making" (p.4661, communication with dementia) : fluctuating preferences complicate decisions; "not enough time to reflect on relatives' and patients' decisions" (p.4660, post-discharge planning) : time pressure constrains deliberation; "about half felt insufficiently competent" (p.4660, self-assessed capability) : perceived skills and knowledge gap; "treatment is continued for too long" (p.4660, escalation and palliation) : delayed palliative conversations	Knowledge; Skills; Social/Professional Role and Identity; Beliefs about Capabilities; Environmental Context and Resources; Social Influences; Memory, Attention and Decision Processes	Doctors → NR; Nurses → time pressure, physician-ownership norms, omitted goal-talk, communication inconsistency, perceived low competence, delayed palliative discussions; AHPs → NR; Managers → NR	Doctors=[NR]; Nurses=[Knowledge; Skills; Social/Professional Role and Identity; Beliefs about Capabilities; Environmental Context and Resources; Social Influences; Memory, Attention and Decision Processes]; AHPs=[NR]; Managers=[NR]
Co-development of BRAIN-TRK: Qualitative examination of acceptability, usability and feasibility of an App to support nurses' care for patients with behavioural and psychological symptoms of neurocognitive disorders in hospital	Redley B; Richardson B; Peel C; Ockerby C; Rawson H; Tomlinson E; Hutchinson A	hard to get people to add on to a full day : Existing heavy workload on general medicine wards makes additional App documentation low priority for nurses, p.2875; "lacking continuity so you're not going to see any trends" : Inconsistent data entry between shifts prevents trend monitoring and reduces perceived usefulness of the App, p.2875; "use very, very minimal technology as nurses" : Low baseline familiarity with digital tools and reliance on handwritten notes reduce comfort using a bedside App, p.2875; "we don't even have time to attend the app" : Managing acute agitation and routine tasks leaves little time to open or update the App during episodes, p.2876; "I was here three days and I asked someone and they didn't know" : Unclear local information about passwords and support contacts makes access difficult for new staff, p.2876; "it did crash a few times" : Occasional App crashes and data loss discourage re-entering information and undermine trust in the system, p.2876	Knowledge; Skills; Beliefs about Consequences; Memory, Attention and Decision Processes; Environmental Context and Resources; Social Influences; Behavioral Regulation	Doctors=[NR]; Nurses=perceived extra workload and time pressure, duplicate documentation, inconsistent use across shifts, low technology familiarity, unclear expectations, device and password issues, peer norms and feeling pressured to use the App; AHPs=[NR]; Managers=[NR]	Doctors=[NR]; Nurses=[Knowledge; Skills; Beliefs about Consequences; Memory, Attention and Decision Processes; Environmental Context and Resources; Social Influences; Behavioral Regulation]; AHPs=[NR]; Managers=[NR]
Working with a robot in hospital and long-term care homes: staff experience	Ren LH; Wong KLY; Wong J; Kleiss S; Berndt A; Mann J; Hussein A; Hu G; Wong L; Khong R; Fu J; Ahmed N; Nolte J; Hung L	I have not touched it because I am not sure (p.8) : Ward nurses on the dementia unit describe avoiding the robot before training because they are unsure how it works or what might happen during calls; "because of the pandemic, you were not able to come and do in person training" (p.10) : Social worker explains that COVID-19 restrictions limited early face to face teaching on the unit, slowing staff confidence and uptake; "If you just send them emails, packages, or a video" (p.10) : Staff note that email and online materials do not reach bedside nurses and care workers who rarely use computers during shifts, so key information about the robot is missed; "sometimes it gets missed, we get calls" (p.15) : Total care worker reports that, with shared devices and busy workloads, scheduled robot sessions can be overlooked and families must phone the unit to prompt staff; "I am one of those apprehensive people with technology" (p.8) : Staff member describes low digital confidence that makes them reluctant to experiment with the robot without support	Knowledge; Skills; Beliefs about Capabilities; Beliefs about Consequences; Emotion; Memory, Attention and Decision Processes; Environmental Context and Resources; Social Influences; Behavioral Regulation	Doctors=[NR]; Nurses=fear of damaging or misusing the robot, limited training access during pandemic, email only information that does not reach direct care staff, time pressure and coordination demands leading to missed calls, discomfort with technology; AHPs=rehabilitation assistants and social worker relied on a few champions to manage scheduling and device set up, shared responsibility for moving and charging robots added to busy caseloads, occasional missed bookings; Managers=patient care coordinators and clinical leaders struggled with inadequate Wi Fi, staffing shortages, and limited technical support when robots disconnected or were not charged; AHPs includes rehabilitation assistants and social worker; Managers includes clinical operations supervisors, clinical educators and patient care coordinators	Doctors=[NR]; Nurses=[Knowledge; Skills; Beliefs about Capabilities; Beliefs about Consequences; Emotion; Memory, Attention and Decision Processes; Environmental Context and Resources; Social Influences; Behavioral Regulation]; AHPs=[Knowledge; Skills; Environmental Context and Resources; Memory, Attention and Decision Processes; Behavioral Regulation]; Managers=[Beliefs about Consequences; Goals; Environmental Context and Resources; Social Influences; Behavioral Regulation]
Agitation near the end of life with dementia: An ethnographic study of care	Sampson EL; Stringer A; La Frenais F; Higgins S; Doyle MJ; Laybourne A; Livingston G; Leavey G	we really need more staff in our care of elderly wards because we have a lot to do : staff nurse in an understaffed older persons ward describes chronic staffing shortages and heavy workload that limit time for agitation support, p.8; "this patient is being too violent, everything, we can't stop him from leaving" : healthcare assistant in Hospital A interprets agitation as deliberate aggression and looks to security rather than exploring underlying distress or unmet need, p.10; " they become very violent towards the nurses as well so we have to be very careful as well" : staff fear of unpredictable aggression from agitated patients encourages defensive distancing and avoidance of close contact, p.10; "I don't think you should bother forcing people like that" : nurse unsure how to respond to refusals during distress defaults to non intervention rather than adapting care, p.10; "people say oh, care of elderly, I can't work there, no" : colleagues describe older peoples wards as undesirable, reflecting stigma that undermines morale, recruitment and investment in dementia care, p.8; "agitation behavior tended to be ignored" : observations on an older adult ward show that visible distress is routinely not responded to unless it blocks tasks such as personal care, p.6; "looking but not intervening was a typical response" : observers note that staff often watch agitated patients without engaging, so opportunities for comfort or de escalation are missed, p.9; unsuitable and unqualified staff are "dumped on older people's wards" : pooled recruitment and low organisational priority place under prepared staff into dementia care roles, reinforcing poor quality responses to agitation, p.8	Knowledge; Skills; Social/Professional Role and Identity; Beliefs about Consequences; Goals; Memory, Attention and Decision Processes; Environmental Context and Resources; Social Influences; Emotion; Behavioral Regulation	Doctors: limited engagement with agitation during ward rounds, focus on other clinical tasks and documentation while ignoring distress calls; Nurses: understaffing, stigma around care of elderly wards, fear of violence, seeing agitation as not core nursing work, reliance on security and typical responses of looking but not intervening; AHPs: earlier tendency to treat everybody the same rather than as individuals, constrained by task focused therapy sessions within busy wards; Managers: pooled recruitment practices that dump unsuitable staff on older peoples wards, minimal investment in dementia training and limited emotional support for frontline staff	Doctors=[Social/Professional Role and Identity; Environmental Context and Resources; Goals; Memory, Attention and Decision Processes]; Nurses=[Knowledge; Skills; Social/Professional Role and Identity; Beliefs about Consequences; Goals; Environmental Context and Resources; Social Influences; Emotion; Behavioral Regulation]; AHPs=[Knowledge; Social/Professional Role and Identity; Environmental Context and Resources]; Managers=[Social/Professional Role and Identity; Goals; Environmental Context and Resources; Social Influences]
Dementia care in acute hospitals: A qualitative study on nurse managers' perceived challenges and solutions	Scerri A; Innes A; Scerri C	the environment is not so conducive to persons with dementia : physical environment not designed with dementia friendly features, p.400 to 402; "the hospital was not built with the purpose to admit patients with dementia" : hospital infrastructure and mission prioritise acute episodes over dementia care, p.400 to 402; "there are no colour coding, so it is very difficult to orientate" : lack of colour cues and wayfinding makes wards disorientating, p.402; "some medical specialists won't listen that there are social issues" : medically dominated hierarchy limits responsiveness to nurses' social and dementia concerns, p.402 to 403; "most of the nurses they just label the patient as confused" : ageist labelling culture normalises cognitive symptoms and undermines personhood, p.403; "we have a huge issue about the nurse:patient ratio" : chronic nurse staffing shortages restrict time for dementia focused care, p.403; "we have a lot of transfers in the afternoon" : workflow driven ward transfers overload staff and destabilise patients with dementia, p.403	Knowledge; Skills; Social/Professional Role and Identity; Beliefs about Consequences; Goals; Environmental Context and Resources; Social Influences; Emotion; Behavioral Regulation	Doctors → medical dominance and variable decisions on restraints, limited attention to social issues and dementia assessment; Nurses → ageist labelling of patients as confused or social cases, low interest in dementia training, task and staff centred ward routines; AHPs → NR; Managers → constrained by rigid hospital hierarchy, lack of strategic focus on dementia and chronic staffing and resource limitations	Doctors=[Knowledge; Beliefs about Consequences; Goals; Environmental Context and Resources; Social Influences]; Nurses=[Knowledge; Skills; Social/Professional Role and Identity; Beliefs about Consequences; Goals; Environmental Context and Resources; Social Influences; Emotion; Behavioral Regulation]; AHPs=[NR]; Managers=[Knowledge; Social/Professional Role and Identity; Beliefs about Consequences; Goals; Environmental Context and Resources; Social Influences; Emotion; Behavioral Regulation]
Creating a toolkit with stakeholders for leveraging tablet computers to support person-centred dementia care in hospitals	Shadarevian J; Chan C; Berndt A; Son C; Gregorio M; Horne N; Mann J; Wallsworth C; Chow B; O'Neill R; Hung L	I use a different phone, so how does this iPad work? We need to know how to find the family video, what is the password (p.4) : staff lack clear procedural knowledge and login details for accessing and using family videos during care; "yes, we need education and some things that we can use to help" (p.4) : nurses and care staff perceive a training gap and need practical supports to feel confident using tablets; "not familiar with electronics" (p.5, Table 2) : limited digital literacy among staff and some patients reduces confidence and willingness to use tablets; "Nurses were fearful about dropping the tablet while juggling medications" (p.5, Table 2) : mechanical instability and competing tasks make device use feel risky in routine medication rounds; "it was hard to get to the right angle for the patient to watch" (p.5, Table 2) : ergonomics of holding and positioning the tablet hinder sustained use at the bedside; "privacy was raised as a concern by staff" (p.5) : uncertainty about information governance and who may access stored family videos limits uptake; "All users need detailed knowledge about the process of how to implement the tablet intervention" (p.5, Table 2) : absence of a clear stepwise process for creating, storing and playing videos is a barrier; "Wi-Fi connectivity, Power demand, Malfunctioning applications" (p.5, Table 2) : unstable connectivity, power limitations and unreliable apps undermine trust in the technology; "lack of person-centred care" (p.5, Table 2) : existing task driven routines mean personalising tablet activities is not automatic without deliberate planning; "Health organizations need to invest resources on acquiring touchscreens materials and increasing human capacity" (p.7) : limited organisational resources and staff time constrain wider implementation of tablet based dementia care	Knowledge; Skills; Environmental Context and Resources; Emotion; Beliefs about Consequences	Doctors=[NR]; Nurses=limited familiarity with electronics and tablet workflows, fear of dropping devices while juggling medications, difficulty positioning tablets and managing technical issues within busy routines; AHPs=[NR]; Managers=concerns about privacy, data access and organisational policies for storing family videos, awareness that current resources and staffing are insufficient for wider rollout	Doctors=[NR]; Nurses=[Knowledge; Skills; Environmental Context and Resources; Emotion; Beliefs about Consequences]; AHPs=[NR]; Managers=[Knowledge; Environmental Context and Resources; Beliefs about Consequences]
Implementing the PIE (Person, Interaction and Environment) programme to improve person-centred care for people with dementia admitted to hospital wards: a qualitative evaluation	Ann Skingley; Mary Godfrey; Rosemary Henderson; Kim Manley; Rosie Shannon; John Young	both observers and observed initially felt out of their comfort zone (p.6) : New observation role felt unfamiliar so staff were uneasy about watching and being watched; "staff being removed to cover absence on other wards" (p.12) : Staffing redeployment across wards reduced stable ward staffing, limiting time for PIE observations and meetings; "staff appeared stressed by simply trying to maintain responsive care" (p.9) : High patient acuity and winter pressures meant energy went to basic care, leaving little capacity for improvement work; "little interaction between staff and patients for long periods in the evening" (p.9) : Low evening staffing and routine task focus normalised minimal engagement, so interaction gaps were not prioritised for change; "they were powerless to act on it" (p.9) : Staff believed they could not influence staffing levels, weakening motivation to use PIE findings for action planning; "demand pressures and organisational uncertainty dampened both" (p.11) : Ongoing restructuring and performance pressures eroded staff confidence and willingness to invest effort in PIE; "found it too much to take on" (p.12) : Combining ward management with facilitation responsibilities overloaded local leaders and undermined sustained implementation	Knowledge; Skills; Social/Professional Role and Identity; Beliefs about Capabilities; Beliefs about Consequences; Goals; Environmental Context and Resources; Social Influences; Emotion; Behavioral Regulation	Doctors=[NR]; Nurses=[NR]; AHPs=[NR]; Managers=[NR]	Doctors=[NR]; Nurses=[NR]; AHPs=[NR]; Managers=[NR]

Title	Author(s)	Barrier quotations and granular barrier descriptions	TDF Domains – Barriers	Barriers specific to profession	Barriers per profession → TDF
The barriers and facilitators to implementing dementia education and training in health and social care services: a mixed-methods study	Surr CA; Parveen S; Smith SJ; Drury M; Sass C; Burden S; Oyebode J	maybe it did not recognise that the service has moved on in the last years : ward nurse felt training did not acknowledge recent service changes, so content seemed outdated, p.5; "the ward environment is not as practical as it needs to be for dementia patients" : hospital staff member described busy ward layout as not dementia friendly, limiting implementation of training ideas, p.6; "that training stays with them but does not get shared and therefore the culture remains" : trainer explained that without supportive ward culture and sharing mechanisms, individual training remains personal and does not shift norms, p.6; "Not all learners are confident with e-learning" : audit respondent noted some staff lack confidence with online systems, reducing engagement with self directed e learning, p.5; "one Doctor fell asleep, it is demoralising really" : dementia lead reported a doctor sleeping in a mandatory session, illustrating low motivation and demoralising trainers, p.7; "Why do I have to go on the dementia training, I went ten years ago" : ward manager described long serving staff who question repeated dementia training and see no added benefit, p.7; "Family can sometimes create barriers, if they have their own thoughts on what is best" : survey respondent noted that relatives preferences can conflict with training informed dementia care plans, p.6	Beliefs about Consequences; Environmental Context and Resources; Social Influences; Emotion; Beliefs about Capabilities	Doctors=[NR]; Nurses=[NR]; AHPs=[NR]; Managers=[NR]	Doctors=[NR]; Nurses=[NR]; AHPs=[NR]; Managers=[NR]
Components of impactful dementia training for general hospital staff: a collective case study	Surr CA; Sass C; Burnley N; Drury M; Smith SJ; Parveen S; Burden S; Oyebode J	their study leave gets cancelled : clinical demand on busy acute wards overrides nurses study leave, leaving no protected time for dementia training, p.518; "you'd like to send 3 people but you can't" : nurse vacancies and low staffing make it unsafe to release several staff for dementia courses, p.518; "we've had to withdraw staff from training" : short staffed wards pull nurses back from study days, disrupting implementation of dementia training, p.518; "there's no one around to delegate the task" : understaffing limits capacity for basic relational care such as offering drinks or conversation, p.518; "that's to do with not having enough staff" : staff attribute missed dementia care to chronic understaffing rather than lack of will, p.518; "They're very negative towards dementia" : agency staff without dementia training bring task first attitudes and uncertainty about responding to distress, p.518; "getting Medics interested and to attend training" : doctors show low engagement with dementia sessions so the medical workforce remains undertrained, p.518; "the ward environment isn't as practical as it probably needs to be" : standard acute ward layout and limited dementia friendly space restrict activity and orientation, p.518	Knowledge; Skills; Intentions; Environmental Context and Resources; Emotion	Doctors=[difficulty engaging medics in dementia training and inconsistent attendance]; Nurses=[lack of protected study leave, staffing shortages and workload limit training and practice change]; AHPs=[NR]; Managers=[part time dementia training lead and competing operational demands reduce visible leadership and flexibility for dementia work]	Doctors=[Intentions]; Nurses=[Environmental Context and Resources]; AHPs=[NR]; Managers=[Environmental Context and Resources]
Visual identifiers for people with dementia in hospitals: a qualitative study to unravel mechanisms of action for improving quality of care	Sutton E; Armstrong N; Locock L; Conroy S; Tarrant C	completely different ones, it can be confusing : non standardised symbols across hospitals confuse staff and weaken identifiers as quick cues, p.605, Table 3; "they may or may not remember to use it" : inconsistent application on some wards means eligible patients are not always flagged for dementia support, p.605, Table 3; "all it does is an identifier that they have dementia" : label alone gives no person specific information to guide person centred care, p.605, Table 3; " if people base assumptions about the diagnosis" : staff rely on dementia category assumptions instead of assessing individual abilities and needs, p.605, Table 3; "why have staff not read that" : personalised booklets exist but are rarely read or used in busy ward routines, p.605, Table 3; "because of the staffing issues" : limited staffing and lack of one to one cover restrict extra supervision for people with dementia, p.605, Table 3; "it's just horrible, they just think there's nothing more you can do" : negative staff attitudes and low expectations foster stigma and increase neglect risk, p.605, Table 3	Knowledge; Skills; Beliefs about Consequences; Environmental Context and Resources; Social Influences; Behavioral Regulation	Doctors=[NR]; Nurses=[time pressure and staffing shortages limit completion and use of personalised documents, risk of relying on the dementia label rather than knowing the patient]; AHPs=[NR]; Managers=[non standardised symbols, unclear eligibility rules and responsibility for applying identifiers lead to inconsistent implementation across wards]	Doctors=[NR]; Nurses=[Environmental Context and Resources; Knowledge; Skills; Beliefs about Consequences]; AHPs=[NR]; Managers=[Environmental Context and Resources; Social Influences; Behavioral Regulation]
A qualitative focus group discussion study on the experiences of Certified Nurses in Dementia Nursing related to effective staff education	Taneichi H; Rokkaku R	I have no power to control personnel placement : DCNs have no formal authority over staffing or relief systems for busy acute wards, so they must lobby managers to create supportive conditions for dementia care education, p.550; "nurses were sometimes uneasy about dementia care" : ward nurses feel uncertain and anxious about managing BPSD in acute settings, which reduces confidence to apply training without DCN support, p.551; "some DCNs had two roles: staff and DCN" : holding both ward nurse and specialist roles leaves DCNs short of time and energy for sustained educational work with staff, p.551; "overworked nurses are generally desperate to end even basic patient care" : high workload and time pressure in Japanese acute wards crowd out person centred dementia care and limit attention for learning during shifts, p.552; "the staff considers the care of patients with dementia to be troublesome" : when dementia care is seen as disrupting the routine of acute treatment, staff are less motivated to engage with DCN led education or change practice, p.552	Knowledge; Skills; Beliefs about Capabilities; Beliefs about Consequences; Environmental Context and Resources; Social Influences; Emotion	Doctors=[NR]; Nurses=[overworked, time pressured nurses feel uneasy about dementia care and may see it as troublesome, relying on DCNs and struggling to integrate person centred dementia care into acute routines]; AHPs=[NR]; Managers=[managers are under pressure to maintain efficiency in acute hospitals, which can limit support and relief staffing for dementia care initiatives]	Doctors=[NR]; Nurses=[Knowledge; Skills; Beliefs about Capabilities; Beliefs about Consequences; Environmental Context and Resources; Emotion]; AHPs=[NR]; Managers=[Environmental Context and Resources; Goals]
Deconstructing dementia and delirium hospital practice: using cultural historical activity theory to inform education approaches	Teodorczuk A; Mukaetova-Ladinska E; Corbett S; Welfare M	they cease to look at it as a person, it is somebody with chest that needs x, y and z : task and systems orientated ward culture focuses on medical problems over understanding the person, Int 04, line 402; if it is busy in the hospital, the quality of care goes down because staff do not have the time : time pressure and workload crowd out getting to know confused patients, Int 11, line 437; I do really feel pressure from staff to try and get me to do something : social pressure from ward staff pushes junior doctors towards quick pharmacological fixes for agitation, Int 02, line 126; I'm capable of feeding someone soup, I'm not allowed to do it : rigid role boundaries prevent support staff from acting on obvious care needs, Int 12, line 909; if they're on Donepezil, perhaps that'll make you think they must have a dementia : fragmented information and limited use of existing dementia cues across services, FG 04, line 256; even if I knew the answer I wouldn't say it : low status staff withhold patient knowledge because they feel their input is not valued, Int 12, line 422	Knowledge; Skills; Social/Professional Role and Identity; Beliefs about Capabilities; Beliefs about Consequences; Goals; Memory, Attention and Decision Processes; Environmental Context and Resources; Social Influences; Emotion; Behavioral Regulation	Doctors: feel pressured by ward staff to prescribe sedatives for challenging behaviour, have limited dementia and delirium specific training and find it difficult to resist expectations during night calls; Nurses: some adopt task based coping to avoid emotional distress, prioritise medical tasks and sedation over person centred dementia care and strongly influence ward responses to agitation; AHPs: occupational therapists, social workers and other staff often have detailed psychosocial knowledge about patients but are unheard in hierarchical meetings and are constrained by time pressure; Managers: NR	Doctors=[Knowledge; Skills; Beliefs about Consequences; Social Influences; Emotion; Goals]; Nurses=[Knowledge; Skills; Social/Professional Role and Identity; Goals; Social Influences; Emotion; Environmental Context and Resources]; AHPs=[Knowledge; Social/Professional Role and Identity; Beliefs about Capabilities; Social Influences; Environmental Context and Resources; Emotion]; Managers=[NR]
The impact of an education intervention on the approach of hospital staff to patients with dementia in a Danish general hospital setting: An explanatory sequential mixed-methods study	Toubøl A; Moestrup L; Thomsen K; Ryg J; Hansen DL; Foldager M; Jakobsen S; Nielsen DS	Previously, you could feel quite alone with these patients (p.36) : limited collegial support and shared responsibility for dementia care, staff managed challenging dementia situations alone, reducing confidence and resilience; "I think that there is a lot more work to be done" (p.36) : dementia friendly routines need ongoing reinforcement and are not yet fully embedded, with risk that focus on dementia care fades over time	Environmental Context and Resources; Social Influences; Behavioral Regulation	Doctors=[NR]; Nurses=[NR]; AHPs=[NR]; Managers=[NR]	Doctors=[NR]; Nurses=[NR]; AHPs=[NR]; Managers=[NR]
CogChamps: impact of a project to educate nurses about delirium and improve the quality of care for hospitalized patients with cognitive impairment	Travers C; Henderson A; Graham F; Beattie E	tasks that are viewed as everyone's responsibility but for which no one is directly accountable (p.10) : dementia and delirium care activities are shared across staff without clear ownership, so non urgent tasks like orientation, nutrition support and engagement are easily left undone; "delirium will continue to be under recognized and under treated without a concerted effort by hospital leaders (p.10)" : limited organisational priority and leadership focus on delirium makes it harder for nurses to prioritise and sustain cognitive impairment care practices	Environmental Context and Resources; Social Influences; Goals; Behavioral Regulation	Doctors=[NR]; Nurses=[Heavy workloads and competing demands make it harder to complete discretionary cognitive impairment care tasks; diffuse responsibility means some delirium related care activities are not consistently performed; limited hospital leadership priority for delirium limits nurses' ability to sustain new practices]; AHPs=[NR]; Managers=[NR]	Doctors=[NR]; Nurses=[Environmental Context and Resources; Social Influences; Goals; Behavioral Regulation]; AHPs=[NR]; Managers=[NR]
Care of patients with dementia in an acute trauma and orthopaedics unit	McCorkell G; Harkin D; McCrory V; Lafferty M; Coates V	staff on the unit felt stressed when caring for such patients (p.46) : workload and cognitive-care burden limit adoption; " it can be challenging for staff to identify and provide additional assistance on a busy ward" (p.46) : competing priorities hinder implementation; "the toolkit was not applied appropriately by some nurses" (p.49) : inconsistent fidelity; "staff required further education on the use of the dementia toolkit" (p.49) : knowledge and skills gap; "staff on the unit required information about what practices were to change" (p.49–50) : unclear behavioural expectations; "the ward environment is dynamic" (p.49–50) : constant change disrupts sustainability; "staff often have little opportunity to leave the clinical environment for training" (p.52) : time pressure limits training exposure; "meeting dementia-related needs may be considered a low priority that requires additional time" (p.51) : task-first culture deprioritises dementia care; " staff... may have an acute care mindset that does not consider specific needs of cognitive impairment" (p.51) : professional norms misaligned with DFHC	Environmental Context and Resources; Knowledge; Social/Professional Role and Identity; Goals; Memory, Attention and Decision Processes	Doctors=[NR]; Nurses=[NR]; AHPs=[NR]; Managers=[NR]	Doctors=[NR]; Nurses=[NR]; AHPs=[NR]; Managers=[NR]
Caring for people with dementia in hospital: findings from a survey to identify barriers and facilitators to implementing best practice dementia care	Tropea J; LoGiudice D; Liew D; Roberts C; Brand C	very noise busy environment can worsen symptoms (p.472, ED noise) : overstimulating environment; "difficult to have room appear familiar" (p.472, unfamiliar setting) : unfamiliar surroundings increase confusion; "poorly designed cubicles not age friendly" (p.472, layout) : inadequate physical layout; "unable to provide calm quiet area" (p.472, no quiet space) : limited calming spaces; "not enough staff to ensure safety" (p.472, staffing) : staffing shortages; "workload competing priorities" (p.472, workload) : competing task demands; "not enough time for one-on-one care" (p.472, time) : time pressure; "staff not sufficiently trained" (p.472, training) : limited dementia knowledge; "some staff not aware best way to communicate" (p.472, comms) : communication skills gap	Environmental Context and Resources; Knowledge; Skills; Memory, Attention and Decision Processes; Social/Professional Role and Identity; Social Influences	Doctors=[NR]; Nurses=[NR]; AHPs=[NR]; Managers=[NR]	Doctors=[NR]; Nurses=[NR]; AHPs=[NR]; Managers=[NR]
The use of the truth and deception in dementia care amongst general hospital staff	Turner A; Eccles F; Keady J; Simpson J; Elvish R	we spend most of the time with them, we don't really get to hear anything about them : Support worker caring for patients with dementia lacks access to personal background information needed to judge truthful or deceptive responses, p.865; "I don't have time to sit with them and make them feel better" : Staff nurse describes time pressure and workload limiting capacity to support patients after difficult conversations about truth or deception, p.865; "I'm here for ward round, then I'm off somewhere else" : Doctor's limited ward presence leads them to hand over dementia related questions to nurses rather than respond directly, p.866	Knowledge; Social/Professional Role and Identity; Beliefs about Consequences; Goals; Memory, Attention and Decision Processes; Environmental Context and Resources; Social Influences; Emotion; Behavioral Regulation	Doctors: Limited time on the ward and task focused ward rounds mean doctors often defer difficult dementia related conversations to nurses; Nurses: Time pressure and heavy workload, being the endpoint for passed on questions, fear of causing distress or complaints, and conflicting duties to be truthful yet avoid harm; AHPs: Sporadic ward presence and lack of access to personal information limit confidence in responding to dementia related questions; Managers: Concern about professional accountability and blame discourages explicit guidance about when deception is acceptable	Doctors=[Social/Professional Role and Identity; Environmental Context and Resources]; Nurses=[Knowledge; Social/Professional Role and Identity; Beliefs about Consequences; Environmental Context and Resources; Emotion; Memory, Attention and Decision Processes]; AHPs=[Social/Professional Role and Identity; Environmental Context and Resources]; Managers=[Social/Professional Role and Identity; Beliefs about Consequences; Social Influences]
Nurses' Perspectives and Experiences of Using a Bed-Exit Information System in an Acute Hospital Setting: Mixed Methods Study	Walzer S; Schön I; Pfeil J; Klemm S; Ziegler S; Schmoor C; Kunze C	the BES is the wrong way, because we actually need more people in the clinic : Nurses perceive technology as an inadequate substitute for low staffing and lack of sitters, p.8; "who gets a BES and for what reasons" : Difficulty defining clear indications makes it hard to operationalise consistent BES use, p.8; "that'll be the mat again" : Frequent alarms create information overload and habituation, increasing risk that alerts are downplayed or delayed, p.8	Knowledge; Social/Professional Role and Identity; Beliefs about Consequences; Goals; Memory, Attention and Decision Processes; Environmental Context and Resources; Emotion; Behavioral Regulation	Doctors: [NR]; Nurses: Challenges identifying suitable patients and scenarios for BES, high expectations not fully met, alarm fatigue and undifferentiated call signals, workload and staffing pressures limiting responses, and difficulty reconciling BES alerts with other fall prevention strategies; AHPs: [NR]; Managers: [NR]	Doctors=[NR]; Nurses=[Knowledge; Beliefs about Consequences; Goals; Memory, Attention and Decision Processes; Environmental Context and Resources; Emotion; Behavioral Regulation]; AHPs=[NR]; Managers=[NR]

Title	Author(s)	Barrier quotations and granular barrier descriptions	TDF Domains – Barriers	Barriers specific to profession	Barriers per profession → TDF
Junior doctor dementia champions in a district general hospital (innovative practice)	Wilkinson I; Coates A; Merrick S; Lee C	did not have the practical knowledge required : limited practical dementia care knowledge among some junior doctors created uncertainty in looking after patients with dementia, p.4; "we had so many people suffering from dementia on the wards" : high dementia caseload made junior doctors aware of large unmet needs and workload pressure, p.5; "we needed to know how to do everything" : multiple dementia screening, investigation and referral steps plus audit requirements made implementation feel demanding, p.5; "you have to really like patients to want to be a dementia champion" : variable intrinsic motivation and preferences for patient contact influenced willingness to take on champion roles, p.4; "Being too busy was seen as a barrier" : time pressure and workload on busy wards reduced feasibility of providing more active hands on dementia care, p.6; "more active care would not be supported" : perception that the rest of the team would not back extra dementia care time limited behaviour change, p.6; "part of the nurse’s role" : belief that practical dementia care tasks belong to nurses reinforced professional role boundaries, p.6; "standard of care delivered to patients with dementia was less good" : perception that ward care for dementia is poorer than for other conditions lowered expectations of care quality, p.5	Knowledge; Skills; Social/Professional Role and Identity; Beliefs about Consequences; Environmental Context and Resources; Social Influences; Emotion	Doctors : limited practical dementia care knowledge; high dementia caseload and workload pressures; multiple dementia screening and investigation tasks to complete; variable intrinsic motivation for hands on dementia care; perceptions that ward dementia care is poorer than care for other conditions; time pressure, short rotations and busy ward routines; belief that practical dementia care tasks are part of the nurse’s role; concern that colleagues or seniors may not support more active dementia care; Nurses : NR; AHPs : NR; Managers : NR	Doctors=[Knowledge; Skills; Social/Professional Role and Identity; Beliefs about Consequences; Environmental Context and Resources; Social Influences; Emotion]; Nurses=[NR]; AHPs=[NR]; Managers=[NR]
Nurses’ Experiences in Caring for Older Adults With Responsive Behaviors of Dementia in Acute Care	Yous ML; Ploeg J; Kaasalainen S; Schindel Martin L	basic nursing care, it is difficult : Routine procedures such as blood pressure measurement can trigger distress and escalate behaviours making essential tasks hard to complete, p.6; "you get a little frustrated, it is a medicine floor, it is very busy" : High workload and busyness on the medical floor limit time and emotional capacity for dementia care, p.7; " Due to acuity in patients, it is priority over chronic" : Acute medical emergencies and investigations are prioritised so dementia related needs drop down the nursing priority list, p.8; "it is an active environment so it is even harder" : Constant movement for tests and multiple staff in and out create a noisy overstimulating ward that hinders calm dementia care, p.8; "We cannot look after these patients the way they should be looked after" : Inadequate nurse and PSW staffing constrains person centred interaction and comfort for patients with dementia, p.9; "I think they are very bored, I think they are just sitting" : Lack of meaningful activity and social stimulation on the ward contributes to confusion and responsive behaviours, p.9; "the only form before physical restraints, it is usually PRN Haldol" : Limited time and few nonpharmacological options lead nurses to rely on sedating medication as a last resort for safety, p.7; " limited time, limited resources, competing priorities" : System pressures and scarce resources push staff towards quicker tranquillising solutions instead of labour intensive dementia care, p.7; "I find we do not really have in services" : Few accessible refresher sessions in dementia care reduce opportunities to reinforce skills and best practice on the unit, p.11	Environmental Context and Resources; Knowledge; Skills; Goals; Beliefs about Consequences; Emotion	Doctors=[NR]; Nurses=[limited time and acute priorities reduce attention to dementia care, inadequate staffing and heavy workload constrain therapeutic contact, understimulating ward environments contribute to responsive behaviours, reliance on chemical restraints in busy periods, limited in services and follow up after episodes of aggression]; AHPs=[limited time resources and competing priorities steer practice towards quicker sedating solutions, discharge pressures and throughput targets challenge consistent dementia focused care]; Managers=[NR]	Doctors=[NR]; Nurses=[Environmental Context and Resources; Knowledge; Skills; Goals; Beliefs about Consequences; Emotion]; AHPs=[Environmental Context and Resources; Goals]; Managers=[NR]
Assessing the Attitudes of Greek Nurses Toward Computerized Dementia Screening	Stelios Zygouris; Mara Gkioka; Despina Moraitou; Birgit Teichmann; Thrasyvoulos Tsiatsos; Sotirios Papagiannopoulos; Magda Tsolaki	the main reported barriers were cost of equipment : high costs of buying and maintaining computerized testing equipment are seen as a primary obstacle in public hospitals, p.1581; "insufficient training" : nurses anticipate that limited dementia and ICT training will leave staff unprepared to use computerized screening confidently, p.1581; "lack of a plan for the integration of computerized dementia screening in the daily routine of the hospital" : absence of clear workflows and procedures for when and how to screen undermines routine implementation, p.1581; "time needed for staff training" : nurses expect it will be difficult to free enough time from routine duties to train staff on computerized screening, p.1581; "length of administration, including time needed for interpretation of test results can be a significant barrier to adoption" : perceived extra time for running tests and interpreting reports reduces feasibility in already busy services, p.1577; "Underutilization after initial implementation remains an issue" : prior experience with other hospital technologies makes nurses wary that tools may be installed but not regularly used, p.1577; "Healthcare professionals accept change at different rates" : variation in how quickly staff adapt to new technologies creates uneven uptake and slows consistent adoption, p.1577; "the overall culture and work environment of the hospital also has an effect" : task focused, pressured ward cultures can limit experimentation with new tools like computerized screening, p.1577	Knowledge; Skills; Goals; Environmental Context and Resources; Social Influences; Beliefs about Consequences	Doctors=[NR]; Nurses=cost of equipment, insufficient training and ICT skills, lack of an integration plan into daily hospital routines, limited time for staff training and testing; AHPs=[NR]; Managers=[NR]	Doctors=[NR]; Nurses=[Knowledge; Skills; Goals; Environmental Context and Resources; Social Influences; Beliefs about Consequences]; AHPs=[NR]; Managers=[NR]
