# Supplementary material for: Advancing dementia-friendly hospital care: a systematic review of healthcare professionals’ reported barriers and facilitators using the theoretical domains framework
Source: Age Ageing. 2026 Sep 22;55(9):afag285. doi: 10.1093/ageing/afag285 (PMC13596736; doi:10.1093/ageing/afag285)
Supplement: Supplementary file 1 [file supplementary_material_afag285.zip › aa-26-1308-File005.pdf]

Advancing Dementia-Friendly Hospital Care: A Systematic Review of Healthcare Professionals’ Reported Barriers and Facilitators Using the Theoretical Domains Framework

Appendix 4. Full coding matrix of facilitators

Supplementary Data

Full coding matrix of facilitators

Title	Author(s)	Facilitator quotations and granular facilitator descriptions	TDF Domains – Facilitators	Facilitators specific to profession	Facilitators per profession → TDF
Facilitators and barriers to implementing a specialized care unit for persons with cognitive impairment in an acute geriatric hospital: a process evaluation	Adlbrecht L; Karrer M; Helfenberger N; Ziegler E; Zeller A	this project was the first big interprofessional project (p.10) : an interprofessionally composed project group gives each profession a voice in designing the specialized unit and supports shared ownership of implementation decisions; "we treat nine patients and, depending on the necessity, one patient a little longer" (p.7) : some therapists are willing to flex treatment duration between patients, which supports more individualized, dementia sensitive therapy scheduling within systemic constraints; "we all have self responsibility" (p.10) : emphasising individual professional responsibility encourages some clinicians to adopt elements of the specialized unit model in their own practice without waiting for formal roll out; "I would like to see togetherness" (p.11) : explicit valuing of working with, rather than alongside, other professions motivates efforts to strengthen interprofessional collaboration around persons with cognitive impairment	Knowledge; Skills; Social/Professional Role and Identity; Beliefs about Consequences; Goals; Environmental Context and Resources; Social Influences; Emotion; Reinforcement; Behavioral Regulation; Optimism	Doctors=[NR]; Nurses=motivation to improve stressful baseline situation on the ward, willingness to act as change agents, and strong engagement in the interprofessional project group and workshops; AHPs=participation of therapists and social workers in the project group and focus groups, bringing their perspectives into interprofessional discussions; Managers=initial decision by hospital leadership in favour of introducing the specialized unit and some visibility of the project in management committees	Doctors=[NR]; Nurses=[Knowledge; Skills; Social/Professional Role and Identity; Beliefs about Consequences; Goals; Environmental Context and Resources; Social Influences; Emotion; Reinforcement; Behavioral Regulation; Optimism]; AHPs=[Social/Professional Role and Identity; Goals; Environmental Context and Resources; Social Influences]; Managers=[Goals; Environmental Context and Resources; Social Influences]
Person-Centred Dementia Care in an Acute Hospital: Experiences from Nurses Working in a Specialized Dementia Unit	Cheong CY; Tay FHE; Choo J; Yap PLK	we value the person as an individual (N2, p.252) : shared PCC values orient nurses towards respecting autonomy and preferences; "it's more of a deeper relationship" (N8, p.252) : knowing patients histories helps nurses tailor interactions and strengthens commitment to PCC; "it literally brings you to a whole new dementia world" (N5, p.252) : experiential PCC training builds understanding, empathy, skills and confidence; "it's the friendship that keeps us together" (N2, p.253) : strong camaraderie and peer support sustain teamwork and resilience in challenging care; "the support that we get from the doctors" (N3, p.253) : supportive leadership and medical involvement provide backup and validation for nurses PCC efforts	Knowledge; Skills; Social/Professional Role and Identity; Beliefs about Capabilities; Beliefs about Consequences; Goals; Environmental Context and Resources; Social Influences; Emotion; Reinforcement; Behavioral Regulation; Optimism	Doctors=[NR]; Nurses=mandatory PCC training and experiential simulation, on the job learning and role modelling, adequate staffing and a social nurse role, strong teamwork and friendship, peer support sessions and social outings, supportive doctors and nurse leaders, and PCC aligned infrastructure and protocols; AHPs=[NR]; Managers=[NR]	Doctors=[NR]; Nurses=[Knowledge; Skills; Social/Professional Role and Identity; Beliefs about Capabilities; Beliefs about Consequences; Goals; Environmental Context and Resources; Social Influences; Emotion; Reinforcement; Behavioral Regulation; Optimism]; AHPs=[NR]; Managers=[NR]
Prevention and management of behavioural and psychological symptoms in patients with dementia in acute care: a best practice implementation project	Rachid Akrour; Catherine Courret-Gilgen; Beatrice Perrenoud	a formalized process … is in place: standardized workflow (Behavioral Regulation); "training … 79%": skills/knowledge uplift (Knowledge; Skills); "systematically reported as adverse events": monitoring/learning (Reinforcement; Behavioral Regulation); "interprofessional collaboration … strengthened": team support (Social Influences); "calm and hypostimulating environment": environmental enablement (Environmental Context and Resources); "encouraged … to ask for expert referral": accessible expertise (Social Influences).	Knowledge; Skills; Social Influences; Environmental Context and Resources; Behavioral Regulation; Reinforcement; Goals	Doctors → NR; Nurses → training uptake; formalized processes; routine adverse-event reporting; Managers → leadership/planning; ensured implementation; encouraged reporting; AHPs → geriatric psychologist support in demanding cases	Doctors=[NR]; Nurses=[Knowledge; Skills; Behavioral Regulation; Reinforcement; Social Influences]; AHPs=[Social Influences; Skills]; Managers=[Social Influences; Behavioral Regulation; Goals]
Understanding and identifying ways to improve hospital-based cancer care and treatment for people with dementia: an ethnographic study	Laura Ashley; Rachael Kelley; Alys Griffiths; Fiona Cowdell; Ann Henry; Hayley Inman; June Hennell; Margaret Ogden; Maria Walsh; Liz Jones; Ellen Mason; Michelle Collinson; Amanda Farrin; Claire Surr	"We do a lot over the phone" → Flexible review modality (Environmental Context and Resources; Behavioral Regulation); "We did things later in the day" → Tailored timing reduces agitation (Environmental Context and Resources); "Get one of us or a student to sit with her" → Ad hoc supportive staffing (Social Influences; Environmental Context and Resources)	Environmental Context and Resources; Behavioral Regulation; Social Influences	Doctors → NR; Nurses → Use of telephone reviews; later scheduling for distressed patients; AHPs → Ad hoc accompaniment/supervision by radiography/support staff; Managers → NR	Doctors=[NR]; Nurses=[Environmental Context and Resources; Behavioral Regulation]; AHPs=[Social Influences; Environmental Context and Resources]; Managers=[NR]
Implementing an intervention to enhance care delivery and consistency for people with hip fracture and cognitive impairment in acute hospital wards: a mixed methods process evaluation of a randomised controlled feasibility trial (PERFECTED)	Backhouse T; Fox C; Hammond SP; Poland F; McDermott-Thompson V; Penhale B; Cross JL	"Good networking with other staff who are committed": Social Influences; "ward sister has introduced this to the staff": Social Influences; "she just nagged us all the time": Behavioral Regulation; "PERFECT-ER is our ward philosophy": Goals/Social/Professional Role and Identity	Knowledge; Social Influences; Behavioral Regulation; Environmental Context and Resources; Beliefs about Capabilities; Goals	Doctors → "orthogeriatrician… we did a bit of a brainstorm"; Nurses → "tools… are certainly useful to help appropriately nurse"; AHPs → "it's brought an awareness of delirium"; Managers → "ward sister has introduced this to the staff"	Doctors=[Social Influences]; Nurses=[Knowledge; Behavioral Regulation]; AHPs=[Knowledge]; Managers=[Social Influences; Behavioral Regulation]
Implementation of STAR-VA for behavioral symptoms of dementia in acute care: Lessons learned	Hannah M. Bashian; Julia T. Boyle; Seneca Correa; Jane Driver; Caroline Madrigal; Isabel Desroches; Mackenzie Farrell; Olivia Eiten; Katie Flanagan; Talya Shahal; Kelly A. O'Malley	"protected time"; "nurse-to-nurse education"; in vivo education; multiple Nurse Champions (NCs) ensured "24-hour access"; "highly responsive" BC (Behavioral Coordinator); education rounds and brief in-services	Knowledge; Skills; Social/Professional Role and Identity; Social Influences; Environmental Context and Resources; Behavioral Regulation	Doctors → [NR]; Nurses → protected time; in vivo education; NC leadership; AHPs → embedded BC consultation; Managers → leadership buy-in; shared governance	Doctors=[NR]; Nurses=[Knowledge; Skills; Social/Professional Role and Identity; Social Influences; Behavioral Regulation; Environmental Context and Resources]; AHPs=[Social Influences; Environmental Context and Resources]; Managers=[Social Influences; Environmental Context and Resources]
Dementia case-finding in hospitals: a qualitative study exploring the views of healthcare professionals in English primary care and secondary care	Anne-Marie Burn; Jane Fleming; Carol Brayne; Chris Fox; Frances Bunn	"member of staff … daily goes to the ward … documentation … completed": dedicated chaser role; "departments are now making commitments … appropriate steps are taken": awareness and engagement	Knowledge; Skills; Social Influences; Environmental Context and Resources; Behavioral Regulation	Doctors → use prompts/direct referral pathways via liaison psychiatry; Nurses → dedicated dementia nurses; awareness/training; Managers → dedicated coordinator/chaser; IT prompts/mandatory fields; AHPs → [NR]	Doctors=[Social Influences; Behavioral Regulation]; Nurses=[Knowledge; Skills; Social Influences; Behavioral Regulation]; Managers=[Environmental Context and Resources; Social Influences; Behavioral Regulation]; AHPs=[NR]
An Action Research Approach to Implementing a Program for Sharing Meaningful Moments With Hospitalized Patients Living With Dementia	Esther Coker; Joanna Sue; Liz Mersereau; Patricia Hewston; Caroline Marr; Sophia Matarazzo; Afeef Vehra; Alexandra Papaioannou	"shining stars" champions; "seeing is believing"; clear role expectations & leadership accountability; reminders to document; audit & feedback; posters at every bedside; bedside posters more accessible than binders; everyone encouraged to contribute	Knowledge; Social/Professional Role and Identity; Beliefs about Consequences; Goals; Memory, Attention and Decision Processes; Environmental Context and Resources; Social Influences; Reinforcement; Behavioral Regulation	Nurses →posters at bedside helpful; reminders & audits support documentation; AHPs=TRs lead posters and activity kits; peer modeling; Managers=leadership sets clear roles and accountability; progress reporting; Doctors=[NR]	Doctors=[NR]; Nurses=[Environmental Context and Resources; Goals; Behavioral Regulation; Reinforcement; Memory, Attention and Decision Processes]; AHPs=[Knowledge; Social Influences; Environmental Context and Resources; Beliefs about Consequences]; Managers=[Social/Professional Role and Identity; Behavioral Regulation; Reinforcement]
Disjunctures in practice: ethnographic observations of orthopaedic ward practices in the care of older adults with hip fracture and presumed cognitive impairment	Jane L. Cross; Tamara Backhouse; Simon P. Hammond; Bridget Penhale; Fiona Scheibl; Nigel Lambert; Anna Varley; Chris Fox; Fiona M. Poland	fold it up nicely: Tailoring/Person-centred adjustment; "You're doing ever so well": Social support/reassurance; "Do you like dancing?": Distraction technique; "It's great, it will make a big difference.": Optimism about e-prescribing; Doctor writes notes to communicate with hard-of-hearing patient: Communication accommodation	Knowledge; Skills; Social Influences; Environmental Context and Resources; Beliefs about Capabilities; Behavioral Regulation; Optimism	Doctors → adapting communication (writing notes; fetching towel); Nurses → reassurance/distraction; small tailoring acts (e.g., folding clothes) to settle distress; AHPs → physiotherapists assist toileting; pharmacist optimism for e-prescribing; Managers → [NR]	Doctors=[Knowledge; Beliefs about Capabilities; Social Influences]; Nurses=[Skills; Behavioral Regulation; Social Influences]; AHPs=[Skills; Optimism; Social Influences]; Managers=[NR]
Patient safety for people experiencing advanced dementia in hospital: A video reflexive ethnography	Ann Dadich; Jade Rodrigues; Anita De Bellis; Annmarie Hosie; Tamsin Symonds; Justin Prendergast; Alan Bevan; Aileen Collier	"everyone has a voice": transparent teamwork; "Staff underwent a 5-day training program": comprehensive unit-wide education; "our perceptive skills have been enhanced": situated learning; "we have time": protected pace enabling person-centred care; "nurse is in charge of the code black": clear role identity; "you can get help anytime": supportive team climate	Knowledge; Skills; Social/Professional Role and Identity; Social Influences; Environmental Context and Resources; Behavioral Regulation; Goals; Beliefs about Capabilities	Doctors →flexible clinical approach supports de-escalation; Nurses=evolved perceptive skills; time to individualize care; easy access to help; AHPs=unit-wide understanding and adaptive practices highlighted by physiotherapist; Managers=leadership transparency; inclusive training; role modelling	Doctors=[Social/Professional Role and Identity; Goals]; Nurses=[Knowledge; Skills; Behavioral Regulation; Beliefs about Capabilities; Environmental Context and Resources; Social Influences; Goals]; AHPs=[Skills; Social Influences; Environmental Context and Resources]; Managers=[Social/Professional Role and Identity; Social Influences; Environmental Context and Resources; Behavioral Regulation; Goals]
Dementia, medication and transitions of care	Louise S. Deeks; Gabrielle M. Cooper; Brian Draper; Susan Kurrle; Diane M. Gibson	"[Community Pharmacist is] Most accurate" (hospital pharmacist); "use… green plastic bags" (hospital pharmacist); "start to self medicate in the hospital" (specialist aged care nurse); "everybody has an electronic record… automatically… alters it" (hospital doctor); "pharmacists do the reviews… put the pressure on the doctors" (RACF nurse)	Knowledge; Skills; Environmental Context and Resources; Social/Professional Role and Identity; Social Influences; Behavioral Regulation; Goals; Beliefs about Capabilities	Doctors → support for unified e-record updates; engagement in structured handover; Nurses → champions (CNC/ASET) supporting medication continuity and dementia-aware discharge; AHPs → pharmacist transition coordinators/community liaison pharmacists; standardized transfer tools; OTs contributing to safe discharge organization; Managers → NR	Doctors=[Goals; Social/Professional Role and Identity]; Nurses=[Environmental Context and Resources; Skills; Behavioral Regulation; Goals; Beliefs about Capabilities]; AHPs=[Environmental Context and Resources; Social/Professional Role and Identity; Behavioral Regulation; Knowledge]; Managers=[NR]
Using sense-making theory to aid understanding of the recognition, assessment and management of pain in patients with dementia in acute hospital settings	Dawn Dowding; Valentina Lichtner; Nick Allcock; Michelle Briggs; Kirstin James; John Keady; Reena Lasrado; Elizabeth L. Sampson; Caroline Swarbrick; S. José Closs	"they have always painkillers prescribed at every six hours" (surgical ward nurse); "we'll go to the next one… ask the pain team" (deputy ward manager); "comfort rounds" prompt asking about pain	Knowledge; Skills; Environmental Context and Resources; Social/Professional Role and Identity; Social Influences; Behavioral Regulation; Goals; Beliefs about Capabilities	Doctors → support structured routines/standing orders; Nurses → comfort rounds; building patient narratives; Specialist/pain teams consultation; HCAs → cue noticing and escalation; AHPs → trigger recognition during therapy sessions; Managers → foster transparency, shared sense-making	Doctors=[Goals; Social/Professional Role and Identity; Environmental Context]; Nurses=[Skills; Environmental Context; Behavioral Regulation; Goals; Beliefs about Capabilities]; HCAs=[Social Influences; Behavioral Regulation]; AHPs=[Knowledge; Skills; Environmental Context]; Managers=[Environmental Context; Social Influences; Goals]
Exploring dementia care in acute care settings: Perspectives of nurses and social workers on caring for patients with behavioral and psychological symptoms	Ruth E. Dunkle; Katherine Cavignini; Joonyoung Cho; Laura Sutherland; Helen Kales; Cathleen Connell; Amanda Leggett	try to redirect and to maintain them safely: non-pharmacological strategy; "try them on a 1 to 1": sitter as support; "family ... able to intervene and calm the patient": family engagement; "we do stretching and movement groups": activity-based calming; "guide them back to their room": de-escalation/wandering management	Environmental Context and Resources; Social Influences; Knowledge; Skills; Behavioral Regulation; Goals	Doctors → [NR]; Nurses → redirection; 1:1 sitters; private rooms; group activities; AHPs → family coordination; de-escalation exercises; therapy consults; guiding wanderers; Managers → [NR]	Doctors=[NR]; Nurses=[Environmental Context and Resources; Behavioral Regulation; Goals; Knowledge; Skills; Social Influences]; AHPs=[Social Influences; Knowledge; Behavioral Regulation; Environmental Context and Resources; Goals]; Managers=[NR]
Using Signs and Symbols to Label Hospital Patients with a Dementia Diagnosis: Help or Hindrance to Care?	Katie Featherstone; Paula Boddington; Andy Northcott	"colored crockery helps the food on the plate stand out": visibility aid; "fantastic people who engage them with music and the telly": engagement; "We are introducing colored crockery … to help people with dementia": environmental support	Environmental Context and Resources; Social Influences; Knowledge; Skills; Behavioral Regulation; Goals	Doctors → [NR]; Nurses → engage with environmental cues (colored crockery); some staff actively use music/TV; Managers → promote visibility initiatives; AHPs → specialist workers verify who actually has dementia	Doctors=[NR]; Nurses=[Environmental Context and Resources; Social Influences; Goals]; AHPs=[Knowledge; Behavioral Regulation]; Managers=[Social Influences; Goals]

Title	Author(s)	Facilitator quotations and granular facilitator descriptions	TDF Domains – Facilitators	Facilitators specific to profession	Facilitators per profession → TDF
Developing a Life Story Intervention for Older Adults With Dementia or at Risk of Delirium Who Were Hospitalized: Multistage, Stakeholder-Engaged Co-Design Study	Sarah J. Flessa; James D. Harrison; Roniela Turnigan; Megan Rathfon; Michael Chandler; Jay Newton-Small; Stephanie E. Rogers	“the humanizing element is really important”: person-centered value; “a champion to inform everybody in the team”: social influence; “make the story easy to find”: EHR accessibility; “an icon next to the patient’s name”: workflow cue; “distill information into an easily digestible format”: usability	Environmental Context and Resources; Social Influences; Knowledge; Skills; Behavioral Regulation; Goals	Doctors=[NR]; Nurses=[NR]; AHPs=[NR]; Managers=[NR]	Doctors=[NR]; Nurses=[NR]; AHPs=[NR]; Managers=[NR]
Issues experienced while administering care to patients with dementia in acute care hospitals: A study based on focus group interviews	Risa Fukuda; Yasuko Shimizu; Natsuko Seto	Nurses noted observation provides the opportunity to notice minute changes: clinical vigilance; Indicators of effort include placing sensor mats and taking patients on rounds: pragmatic adaptations; If nurses and other medical staff could work together, patients would benefit: teamwork as facilitator	Knowledge; Goals; Behavioral Regulation; Environmental Context and Resources; Social Influences	Doctors=[NR]; Nurses=observing minute changes, sensor mats, bringing patients to station, taking patients on rounds, seeking conference discussion and teamwork; AHPs=[NR]; Managers=[NR]	Doctors=[NR]; Nurses=[Knowledge; Environmental Context and Resources; Social Influences; Goals; Behavioral Regulation]; AHPs=[NR]; Managers=[NR]
Effects of a Person Centered Dementia Training Program in Greek Hospital Staff: Implementation and Evaluation	Mara Gkioka; Birgit Teichmann; Despina Moraitou; Sotirios Papagiannopoulos; Magda Tsolaki	supported by hospital management : managerial backing; "champions or mentors are important" : local leadership; "train the trainers" : capacity building; "teamwork and continuous action" : collective focus; "redesigning the ward environment" : enabling context; "delivery twice per year" : ongoing reinforcement; "videos, scenarios and practice based learning" : practical skills building	Knowledge; Skills; Social Influences; Environmental Context and Resources; Goals; Behavioral Regulation; Reinforcement	Doctors=[NR]; Nurses="supported by hospital management; champions or mentors; teamwork and continuous action; train the trainers; delivery twice per year; practice based learning"; AHPs=[NR]; Managers="leadership backing; action plans; resource reallocation and environment redesign"	Doctors=[NR]; Nurses=[Social Influences; Behavioral Regulation; Goals; Environmental Context and Resources; Knowledge; Skills; Reinforcement]; AHPs=[NR]; Managers=[Social Influences; Behavioral Regulation; Environmental Context and Resources; Reinforcement]
The Person, Interactions and Environment Programme to improve care of people with dementia in hospital: a multisite study	Godfrey M; Young J; Shannon R; Skingley A; Woolley R; Arrojo F; Brooker D; Manley K; Surr C	“practice development facilitator acted as a PIE driver”; “made everyone aware of dementia”; “clear and consistent focus on care quality”; “fit between practice change and strategic priorities”	Knowledge; Social Influences; Environmental Context and Resources; Goals	Doctors → NR; Nurses → champions and dementia specialists increased buy in; AHPs → OT leadership supported action planning; Managers → leadership visibility legitimated time for PIE	Doctors=[NR]; Nurses=[Knowledge; Social Influences; Goals; Environmental Context and Resources]; AHPs=[Social Influences; Knowledge]; Managers=[Social Influences; Goals; Environmental Context and Resources]
How do emergency department staff respond to behaviour that challenges displayed by people living with dementia? A mixed-methods study	Goodwin L, Liddiard C, Manning S, Benger JR, Carlton E, Cheston R, Hoskins R, Taylor H, Voss S	music... particularly helpful Table 3: Sensory distraction aids; "a stepwise approach that will guide you" P004: Checklist facilitates consistency; "tick box... make sure they are not in pain" P002: Structured assessment prompt; "make our department a place that prevents things escalating" P012: Prevention framing; "team of Dementia Nurses... sit with the patients" P009: Dedicated staffing enables 1:1 engagement	Knowledge; Behavioral Regulation; Social Influences; Environmental Context and Resources; Goals; Memory, Attention and Decision Processes	Doctors=[NR]; Nurses=[NR]; AHPs=[NR]; Managers=[NR]	Doctors=[NR]; Nurses=[NR]; AHPs=[NR]; Managers=[NR]
Balancing the needs of individuals and services in cancer treatment for people with dementia: A focused ethnographic study	Alys Wyn Griffiths; Laura Ashley; Rachael Kelley; Fiona Cowdell; Michelle Collinson; Ellen Mason; Hayley Inman; Ann Henry; Amanda Farrin; Claire Surr	treating every patient as a person and making sure that you speak to the person: person centred communication, Doctors; "start with what works for you already": tailoring to routines, Nurses; "really check their understanding and write things down more": communication support, Nurses; "keep their routine as much as they can": routine continuity aids treatment, AHPs; "put a hospital wristband on": adapted ID process, AHPs; "photo on the screen": alternative ID check, AHPs; "We got her longer appointments and the same people treated her": continuity and time, AHPs; "We might bring them back to another assessment clinic": staged decision making, Nurses; "otherwise, you make poor judgements": rationale for extra time, Nurses	Knowledge; Skills; Environmental Context and Resources; Behavioral Regulation; Goals; Social Influences; Memory, Attention and Decision Processes; Beliefs about Consequences	Doctors=speak to the person first; Nurses=tailor to routines; check understanding; write things down; AHPs=adapt ID with wristbands or photos; longer appointments; same staff; maintain routines; Managers=[NR]	Doctors=[Social/Professional Role and Identity; Skills]; Nurses=[Knowledge; Skills; Behavioral Regulation; Environmental Context and Resources; Beliefs about Consequences]; AHPs=[Environmental Context and Resources; Behavioral Regulation; Memory, Attention and Decision Processes; Goals]; Managers=[NR]
Supporting general hospital staff to provide dementia sensitive care: A realist evaluation	Melanie Handley; Frances Bunn; Claire Goodman	“Since he has had the pain relief he has been much calmer” : symptom relief reduces distress; “Let’s find this word together” : engaging activity calms and orients; “Let’s look for the edges first” : structured activity supports attention; “I’m going to get her some finger food for breakfast” : tailoring to preferences supports intake; “When she tells you to do X she is doing it from experience” : credible expertise guides practice; “These things you need to teach people and reinforce” : training consolidates skills.	Knowledge; Skills; Social Influences; Environmental Context and Resources; Goals; Beliefs about Consequences	Nurses → engagement activities, symptom management, tailored routines, peer learning; Doctors → NR; AHPs → training that builds empathy and practical skills; Managers → NR	Doctors=[NR]; Nurses=[Knowledge; Skills; Goals; Environmental Context and Resources; Social Influences; Beliefs about Consequences]; AHPs=[Knowledge; Skills; Social Influences]; Managers=[NR]
Exploring ways to enhance pain management for older people with dementia in acute care settings using a Participatory Action Research approach	Deirdre Harkin; Vivien Coates; Donna Brown	“Ask the family what the signs are, if they are normally in pain, what they are, how they would know?” : family input improves detection; “Since the Purple Folder toolkit…more focus on assessing the pain” : toolkit supports assessment; “Visual prompts…purple dot” : cues aid prioritisation; “Identify at triage” : early flagging enables action; “Use a recognised pain assessment tool routinely” : standardised assessment; “To be real and delivered in no more than 30 minutes” : brief training feasible.	Knowledge; Skills; Social Influences; Environmental Context and Resources; Goals; Beliefs about Consequences	Nurses → routine pain tools, family engagement, use of visual prompts and toolkits, reflective practice; Doctors → review and regularise analgesia, respond to flags; AHPs → gather pain signs during drug history, contribute to holistic assessment; Managers → support triage flags, endorse toolkit use, enable 30 minute training	Nurses=[Knowledge; Skills; Social Influences; Environmental Context and Resources; Goals]; Doctors=[Beliefs about Consequences; Environmental Context and Resources]; AHPs=[Knowledge; Skills; Social Influences]; Managers=[Goals; Environmental Context and Resources]
Barriers and enablers to implementing ‘DEALTS2’ simulation-based train-the–trainer dementia training programme in hospital settings across England: a qualitative study	Michelle Heward; Michele Board; Ashley Spriggs; Dina Blagden; Jane Murphy	“Attending the DEALTS2 training and getting the slides or resources was useful” : toolkit access enables delivery; “Given time to attend the workshop and prepare for the training sessions” : protected time facilitates rollout; “The role play simulations got good feedback” : simulations engage learners; “I have adapted them all” : flexibility supports local fit; “It has given me more practicals to use” : concrete methods increase trainer confidence; “We were able to start delivering the 4-hour sessions” : adequate session length supports depth.	Knowledge; Skills; Social Influences; Environmental Context and Resources; Goals; Beliefs about Consequences	Doctors → NR; Nurses → NR; AHPs → NR; Managers → allocate protected time and space, endorse simulations and debriefs, provide equipment and co-facilitators	Doctors=[NR]; Nurses=[NR]; AHPs=[NR]; Managers=[Social Influences; Environmental Context and Resources; Goals]
Person-centred oral hydration care for older people with dementia admitted to acute hospital wards: Empirical research qualitative	Shanlee Higgins; Lesley Baillie; Calvin Moorley; Fiona Nolan	Just trying to encourage and prompt them : Skills and Behavioral Regulation; "anybody going past can offer a drink, it's everybody's job" : Social/Professional Role and Identity and Social Influences; "we can proactively do [oral hygiene] to avoid that building up" : Knowledge and Beliefs about Consequences; "offers him a drink on the walk" : Action integration and Environmental Context and Resources	Knowledge; Skills; Social/Professional Role and Identity; Social Influences; Environmental Context and Resources; Behavioral Regulation; Beliefs about Consequences	Doctors → promote oral hygiene relevance; Nurses → person-centred prompting and integrating hydration with activities; AHPs → occasional direct assist during sessions; Managers → champion hydration as everybody's job and convene hospital-wide strategy	Doctors=[Knowledge; Beliefs about Consequences; Social/Professional Role and Identity]; Nurses=[Skills; Behavioral Regulation; Environmental Context and Resources; Social Influences]; AHPs=[Skills; Environmental Context and Resources]; Managers=[Social/Professional Role and Identity; Social Influences; Goals]
The experience of hospital staff in applying the Gentle Persuasive Approaches to dementia care	Lillian Hung; Cathy Son; Rebecca Hung	Teamwork provides a sense you’re not alone : peer support; "Using a framework provides a common language" : shared understanding; "Now we feel empowered" : confidence; "There was no fuss, and it worked" : cross departmental cooperation; "Protected times for regular team conversation… are key" : structural support	Knowledge; Skills; Social Influences; Social/Professional Role and Identity; Beliefs about Capabilities; Goals; Environmental Context and Resources; Behavioral Regulation	Doctors= shared language for partnership; Nurses= teamwork and empowerment, tailored routines; AHPs= reframing behaviors via GPA principles; Managers=[NR]	Doctors=[Social Influences; Goals]; Nurses=[Beliefs about Capabilities; Skills; Social Influences; Behavioral Regulation; Environmental Context and Resources]; AHPs=[Knowledge; Social Influences]; Managers=[NR]
Nurses' knowledge and beliefs on pain management practices with hospitalised persons living with dementia: A qualitative descriptive study	Beverly Ingelson; Sherry Dahlke; Hannah O'Rourke; Kathleen F. Hunter	I think that the handoff is important : team process; "Families know the patient better than I do" : collateral input; "Simply documenting in the notes that there are certain experiences that are causing pain" : documentation aid; "I assess pain by their vital signs and by their non verbal gestures" : multimodal assessment; "You can just talk to them and then they feel like you're a familiar person" : rapport	Skills; Knowledge; Social Influences; Behavioral Regulation; Beliefs about Capabilities	Doctors=[NR]; Nurses= handoff processes; family input; documentation to guide care; multimodal assessment; rapport building; AHPs=[NR]; Managers=[NR]	Doctors=[NR]; Nurses=[Skills; Knowledge; Social Influences; Behavioral Regulation; Beliefs about Capabilities]; AHPs=[NR]; Managers=[NR]
Living with dementia in hospital wards: a comparative study of staff perceptions of practice and observed patient experience	Innes A., Kelly F., Scerri C., Abela S.	pulling curtains around beds when carrying out personal care : simple dignity cue reinforced by reminders; "signs on toilet, shower and bathroom doors" : wayfinding cues support independence; "pictures of local landmarks and religious objects" : familiar cues aid orientation; "quiet seating area in one ward" : environmental adaptation enables calm interaction; "verandas equipped with more comfortable seating" : improved rest areas support visits; "more positive interactions between staff and patients" : mentoring encouraged engagement; "improved awareness of patient experience" : feedback loop raised awareness	Environmental Context and Resources; Social Influences; Knowledge; Behavioral Regulation	Doctors=[NR]; Nurses=[NR]; AHPs=[NR]; Managers=[NR]	Doctors=[NR]; Nurses=[NR]; AHPs=[NR]; Managers=[NR]
Barriers and facilitators to caring for patients living with dementia in the emergency department: a qualitative study	Jelinski D., Reich K., Lang E., Holroyd-Leduc J., Goodarzi Z.	They have a calming influence often : care partner collateral and comfort; "get them in, get them worked up, get them moving" : streamlined workflow and prioritization; "We have made an incredible difference." : allied health presence and mobilization support; "set up with community resources" : discharge coordination and linkage	Social Influences; Environmental Context and Resources; Knowledge; Skills; Goals	Doctors → person-centred assessment and using care partner collateral; Nurses → prioritizing non-medical needs and efficient flow to reduce harms; AHPs → discharge planning, mobility, community linkage; Managers → NR	Doctors=[Knowledge; Social Influences]; Nurses=[Goals; Environmental Context and Resources]; AHPs=[Environmental Context and Resources; Social Influences; Skills]; Managers=[NR]
Nurses’ experiences of delivering acute orthopaedic care to patients with dementia	Jensen AM; Pedersen BD; Wilson RL; Olsen RB; Hounsgaard L	areas of concern in care : structured EHR cueing prompts relevant dementia info; "there is someone taking care of the children, it is a solution" : validation technique reduces distress; "personality is also taken into consideration" : allocating calm nurses supports de-escalation; "reading up on a patient" : routine pre-shift review supports planning	Knowledge; Skills; Goals; Environmental Context and Resources; Social Influences; Behavioral Regulation	Doctors=[NR]; Nurses=[validation communication used; oral handover and pocket notes; prioritising single rooms]; AHPs=[NR]; Managers=[assign calm nurses to dementia patients; enable single-room placement when feasible]	Doctors=[NR]; Nurses=[Knowledge; Skills; Goals; Environmental Context and Resources; Social Influences; Behavioral Regulation]; AHPs=[NR]; Managers=[Environmental Context and Resources; Social Influences; Goals; Behavioral Regulation]
Health professional perspectives on systems failures in transitional care for patients with dementia and their carers: a qualitative descriptive study	Kable A; Chenoweth L; Pond D; Hullick C	sit down and say this is the plan when you leave : structured discharge conversation aids understanding; "ACAT assessment sent to the GP" : timely information transfer supports continuity; "home medicine review" : post discharge medication support; "dose administration aids for medicines" : dispensing aids support adherence	Knowledge; Environmental Context and Resources; Social Influences; Behavioral Regulation; Goals	Doctors → seeking registrar and nurse input to improve summaries; revising meds post discharge; Nurses → aiming to notify and include nursing summaries; AHPs → arranging referrals and advocating for services; Managers → aligning discharge timing with RACF policy and earlier notification	Doctors=[Knowledge; Social Influences; Behavioral Regulation]; Nurses=[Behavioral Regulation; Goals]; AHPs=[Environmental Context and Resources; Social Influences]; Managers=[Goals; Environmental Context and Resources; Social Influences]
Nurses’ perceptions in caring for people with dementia in Dutch acute hospitals	Annette Keuning-Plantinga; Petrie F. Roodbol; Wim P. Krijnen; Evelyn J. Finnema	being present : caring reaction routine; "ask what is wrong" : problem-solving communication; "negotiate with colleagues or family" : collaborative support; "organise an expert consultation" : accessible specialist input	Knowledge; Skills; Social Influences; Behavioral Regulation	Doctors → NR; Nurses → caring reactions, problem-solving, collegial and family negotiation, expert consultation; AHPs → NR; Managers → NR	Doctors=[NR]; Nurses=[Knowledge; Skills; Social Influences; Behavioral Regulation]; AHPs=[NR]; Managers=[NR]
Radiographers' experiences in working with patients living with dementia in Norway: A qualitative study	E. Kjelle; C. Eikefet; C.C. Chilanga	Music can be useful for many patients : calming sensory cue; "bring them into a quieter environment and minimise their wait time" : environmental modification and scheduling; "Caregivers can provide additional information, we rely heavily on family members" : caregiver collaboration; "We can use fast sequences and more robust protocols" : technology-enabled shorter scans; "set aside time or distribute patients over different days" : protected scheduling	Skills; Social Influences; Environmental Context and Resources; Behavioral Regulation	Doctors → NR; Nurses → NR; AHPs → caregiver involvement, quiet rooms, music, fast protocols, extra time; Managers → NR	Doctors=[NR]; Nurses=[NR]; AHPs=[Skills; Social Influences; Environmental Context and Resources; Behavioral Regulation]; Managers=[NR]
Perioperative Nurses’ Experience of Working With Persons Living With Dementia	Mariya Kovaleva; Abigail C. Jones; Cornelia Frances Garrard; Jana Lauderdale; Mary S. Dietrich; Peace Johnson; Cathy Maxwell	Family members can help keep them calm : caregiver collaboration; "play music" : calming sensory cue; "place rooms near nurses’ station" : proximity supports monitoring; "chart in the room" : observation embedded in workflow; "raise the blinds for daylight" : environmental orientation	Skills; Social Influences; Environmental Context and Resources; Behavioral Regulation	Doctors → NR; Nurses → family presence, environmental calming and daylight, proximity and observation, music and activities, planning ahead; AHPs → NR; Managers → NR	Doctors=[NR]; Nurses=[Skills; Social Influences; Environmental Context and Resources; Behavioral Regulation]; AHPs=[NR]; Managers=[NR]

Title	Author(s)	Facilitator quotations and granular facilitator descriptions	TDF Domains – Facilitators	Facilitators specific to profession	Facilitators per profession → TDF
Implementation of a model of care for hospitalised older persons with cognitive impairment (the Confused Hospitalised Older Persons program) in six New South Wales hospitals	Susan Kurrle; Sigrid Patterson; Cath Bateman; Anthea Temple; Anne Cumming; Glen Pang	executive sponsorship : leadership backing; "clinical lead and clinical champions" : local championing; "medical staff and geriatric team involvement" : interdisciplinary collaboration; "cohesive project team and clear action plan" : structured implementation; "synergy with other programs" : integration into existing workflows; "delirium alert stickers and icons" : visual prompts; "education using DVDs and e-learning" : targeted training; "environmental improvements, clocks and signage" : orientation cues	Knowledge; Skills; Social Influences; Environmental Context and Resources; Behavioral Regulation; Memory, Attention and Decision Processes; Reinforcement	Doctors → NR; Nurses → NR; AHPs → NR; Managers → NR	Doctors=[NR]; Nurses=[NR]; AHPs=[NR]; Managers=[NR]
Redesigning acute care for cognitively impaired older adults: Optimizing health care services	Michael A LaMantia; Malaz A Boustani; Shola Jhanji; Mungai Maina; Arif Nazir; Frank C Messina; Amie Frame; Catherine Alder; Joshua Chodosh	I get updates when our patients go inpatient automatically : EMR alerts cue clinic follow-up, p.920; "I often get a packet with their full evaluation" : standardized handover supports clinic planning, p.920; "Everything starts with education" : clinic staff training prioritization, p.922; "we help with transportation and figure out their insurance" : clinic social work logistics enable attendance, p.921	Knowledge; Environmental Context and Resources; Social Influences; Memory, Attention and Decision Processes; Behavioral Regulation	Doctors → "I get updates when our patients go inpatient automatically"; "I often get a packet with their full evaluation" ; Nurses → "Everything starts with education"; "wrap-around services while our patients are waiting" ; AHPs → "we help with transportation and figure out their insurance"; "I get updates when our patients go inpatient automatically" ; Managers → NR	Doctors=[Knowledge; Environmental Context and Resources; Social Influences]; Nurses=[Knowledge; Environmental Context and Resources; Behavioral Regulation]; AHPs=[Environmental Context and Resources; Social Influences; Memory, Attention and Decision Processes]; Managers=[NR]
Dementia care across a tertiary-care health system: What exists now and what needs to change	Amanda Leggett; Cathleen Connell; Leslie Dubin; Ruth Dunkle; Kenneth M. Langa; Donovan T. Maust; J. Scott Roberts; Beth Spencer; Helen C. Kales	Elder Life volunteers spend 30 minutes twice daily : structured engagement routine supports orientation; "24-hour crisis line" : immediate access enables timely support; "link between psychiatry and the medical/surgical services" : liaison structure facilitates coordination; "repurpose sitter resources to elder life specialists" : role redesign institutionalizes engagement	Social Influences; Knowledge; Environmental Context and Resources; Behavioral Regulation; Memory, Attention and Decision Processes	Doctors=[NR]; Nurses=[NR]; AHPs=[NR]; Managers=[NR]	Doctors=[NR]; Nurses=[NR]; AHPs=[NR]; Managers=[NR]
Understanding and integrating the needs and preferences of people living with dementia in the inpatient setting: a qualitative study	Alyse Lennox; Denise Goodwin; Felicity Leavold; Renae Nicol; Velandai Srikanth; Darshini Ayton; Madeleine Berends; Myra Thiessen; Daphne Flynn; Chris Moran	behaviour huddles we have found are amazing : MDT huddles enable shared understanding, Table 1; "handover is the priority number one" : routine handover supports consistent practice, Table 1; "right there in front of you" : bedside prompts cue actions, Table 1; "role modelling those as well" : on-shift mentoring builds staff capability, Table 1	Knowledge; Social Influences; Environmental Context and Resources; Behavioral Regulation; Memory, Attention and Decision Processes	Doctors → advocate clearer access to behaviour plans and pre-emptive tools; Nurses → value behaviour huddles and structured handover; bedside prompts aid cueing; AHPs → support shared, readily accessible tool and training; Managers → NR	Doctors=[Environmental Context and Resources; Behavioral Regulation]; Nurses=[Social Influences; Behavioral Regulation; Environmental Context and Resources; Knowledge]; AHPs=[Knowledge; Environmental Context and Resources; Memory, Attention and Decision Processes]; Managers=[NR]
The assessment and management of pain in patients with dementia in hospital settings: a multi-case exploratory study from a decision making perspective	Valentina Lichtner; Dawn Dowding; Nick Allcock; John Keady; Elizabeth L. Sampson; Michelle Briggs; Anne Corbett; Kirstin James; Reena Lasrado; Caroline Swarbrick; S. José Closs	knowledge of what is happening within the body : Clinical knowledge guides analgesic titration; "pain relief already regularly on their charts" : Protocolised baseline analgesia supports coverage; "contact with a specialist pain team" : Escalation pathway supports complex cases; "it does recommend half an hour" : Guideline prompts structure reassessment; "drug chart… used to assess and review" : Shared artefact supports team coordination; "we're trying to build a picture" : Interdisciplinary sense-making routine	Knowledge; Skills; Environmental Context and Resources; Behavioral Regulation; Social Influences	Doctors → clinical knowledge of surgery and conditions informs dosing, drug charts centralise information, specialist pain team access; Nurses → standard protocols, drug chart routines, local care plans, reassessment prompts; AHPs → NR; Managers → attempts to introduce tools and electronic integration	Doctors=[Knowledge; Skills; Environmental Context and Resources; Behavioral Regulation]; Nurses=[Knowledge; Behavioral Regulation; Environmental Context and Resources; Social Influences]; AHPs=[NR]; Managers=[Environmental Context and Resources; Social Influences]
What influences decisions about ongoing stroke rehabilitation for patients with pre-existing dementia or cognitive impairment: a qualitative study	Longley V; Bowen A; Peters S; Swarbrick C	give all patients a fair chance (p.1137) : equitable default prompts trial of rehab; "clear, concise language" (p.1140, Table 3) : structured communication aids engagement; "use quiet rooms and spaces" (p.1140, Table 3) : environmental cues reduce overload; "engage family with rehabilitation" (p.1140, Table 3) : family information supports tailoring; "organise an expert consultation" (p.1139) : specialist access supports decision-making	Knowledge; Skills; Environmental Context and Resources; Social Influences; Behavioral Regulation	Doctors → [NR]; Nurses → MDT collaboration supports decisions; AHPs → tailored communication, quiet spaces, family engagement, compensatory strategies, expert consultation; Managers → [NR]	Doctors=[NR]; Nurses=[Social Influences]; AHPs=[Skills; Behavioral Regulation; Environmental Context and Resources; Social Influences; Knowledge]; Managers=[NR]
Facilitators, Barriers, and Solutions in Pain Management for Older Adults with Dementia	Alícia Minaya-Freire; Anna Ramon-Aribau; Gemma Pou-Pujol; Meritxell Fajula-Bonet; Mireia Subirana-Casacuberta	I point to the part of the body I'm referring to : adapted nonverbal communication supports assessment (p.498); "I always try to maintain a soft tone of voice" : calming communication enables assessment (p.498); "it works, but it's a matter of doing" : experience informs timely analgesia decisions (p.498); "They more than anyone know the patient" : caregiver knowledge informs detection (p.498); "in the event that they see... to let me know" : caregiver-initiated alerts integrate into workflow (p.498)	Skills; Knowledge; Social Influences; Beliefs about Capabilities	Doctors=[NR]; Nurses=professional experience; adapted nonverbal and tone adjustments; consulting available caregiver; caregiver-initiated alerts; AHPs=[NR]; Managers=[NR]	Doctors=[NR]; Nurses=[Skills; Knowledge; Social Influences; Beliefs about Capabilities]; AHPs=[NR]; Managers=[NR]
Privileges, and Permissions: Theorising Intersectionality and Cultures of Control in the Care of People Living With Dementia in Acute Hospital Settings	Mwale S; Northcott A; Featherstone K	gently guide him back : supportive guidance used instead of physical stopping, p.6; "the team all chatted and laughed with her" : positive social engagement permits walking around the ward, p.8; "smiled and said hello" : brief relational contact helps de-escalate distress, p.7; "activities coordinator led him by the hand" : purposeful activity offers an alternative to security containment, p.5	Skills; Social Influences; Environmental Context and Resources	Nurses → reassurance and conversational engagement enabling walking; AHPs → activities-based engagement supports safe movement; Doctors → [NR]; Managers → [NR]	Doctors=[NR]; Nurses=[Skills; Social Influences; Environmental Context and Resources]; AHPs=[Environmental Context and Resources; Social Influences]; Managers=[NR]
When people living with dementia say 'no': Negotiating refusal in the acute hospital setting	O'Brien R; Beeke S; Pilnick A; Goldberg SE; Harwood RH	We're just gonna get you a quick shave, is that alright? (Findings, 3.6, Extract 10): Announcement with minimisers plus permission check, reframes task as brief; "Let's have another go" (Findings, 3.6, Extract 11): Collaborative proposal presents drinking as shared activity; "I need to check your mouth, is that alright?" (Findings, 3.6, Extract 12): Statement of HCP need plus permission check signals importance; "deep breath in" (Findings, 3.6, imperative sequence): Bald imperative once activity underway limits refusal space; "just try another sip" (Findings, 3.6, Extract 14): Minimiser plus try-framing lowers task demand	Skills; Social/Professional Role and Identity; Beliefs about Consequences; Memory, Attention and Decision Processes; Behavioral Regulation	Doctors → "I need to…" plus "is that alright?" gains assent; Nurses → "We're just gonna…" announcement with minimisers; AHPs → "Let's have another go" collaborative proposal; "deep breath in" imperative once activity underway; Managers → NR	Doctors=[Skills; Social/Professional Role and Identity; Beliefs about Consequences; Memory, Attention and Decision Processes; Behavioral Regulation]; Nurses=[Skills; Social/Professional Role and Identity; Beliefs about Consequences; Memory, Attention and Decision Processes; Behavioral Regulation]; AHPs=[Skills; Social/Professional Role and Identity; Beliefs about Consequences; Memory, Attention and Decision Processes; Behavioral Regulation]; Managers=[NR]
"Getting to Know Them": Person-Centered Care for Patients With Dementia in Acute Care	Osuoha P; Masoud SS; Leibas M; Cleveland LM; Reed CC; Piernik-Yoder B; White CL	"Gave her a pile of towels to fold, and she sat" : familiar activity cue supports de-escalation, p.39; "We're here to… give them as much comfort as you can" : calm tone and trust-building aid cooperation, p.39; "Go with them… validation" : redirection and validation routines reduce distress, p.39; "Puzzles… coloring pages" : ready-to-hand activity materials provide engagement options, p.40; "Have someone there as a constant is help enough" : family presence offers stabilizing cue in unfamiliar setting, p.40; "Hand-off is important" : standardized handoff shares person info for consistent care, p.39	Knowledge; Skills; Memory, Attention and Decision Processes; Environmental Context and Resources; Social Influences; Behavioral Regulation	Doctors → NR; Nurses → familiar activity cues; calm tone and trust; validation/redirection; activity materials to hand; family presence; stronger handoff routines; AHPs → NR; Managers → NR	Doctors=[NR]; Nurses=[Knowledge; Skills; Memory, Attention and Decision Processes; Environmental Context and Resources; Social Influences; Behavioral Regulation]; AHPs=[NR]; Managers=[NR]
Improving emotional wellbeing for hospital-based patients with dementia	Petty, S., Griffiths, A., Coleston, D.M. & Dening, T.	speaking just to the actual person... tiny little thing (p.7, Routine interactions) : direct inquiry routine elicits personal cues; "having it engrained... ok to just sit... hold their hand" (p.10, Leadership modelling) : permission structure from leaders; "we do make time... fit that extra bit in" (p.10, Micro-time) : teamwork and micro-time redistribution; "watching how colleagues... learning what works" (p.11, Peer learning) : experiential learning and sharing; "get the family involved... ring her daughter" (p.12, Family input) : family as information and comfort resource; "exchanges... can normalise a situation" (p.12, Environment shaping) : conversational presence reduces institutional feel	Social Influences; Skills; Knowledge; Environmental Context and Resources; Social/Professional Role and Identity	Doctors → NR; Nurses → leaders' permission to sit-with, micro-time help, peer learning, family input, conversational presence, tailored inquiry; AHPs → NR; Managers → NR	Doctors=[NR]; Nurses=[Social Influences; Skills; Knowledge; Environmental Context and Resources; Social/Professional Role and Identity]; AHPs=[NR]; Managers=[NR]
Nurses' perspectives on shared decision-making in the daily care of hospitalized patients with dementia: An exploratory qualitative study	Plantinga A; Roodbol PF; van Munster BC; Finnema EJ	family meetings are often scheduled (p.4658–4659, routine workflow) : structured encounters support shared deliberation; "You must take your role... include this in the overall decision" (p.4660, advocacy in MDT) : nurse advocacy and information integration; "reduce options and provide more guidance" (p.4661, ADL decisions) : simplified choices fit cognitive load; "adapt communication, check understanding" (p.4661, tailored interaction) : communication adaptation supports participation; "choose a calm time and low-stimulus setting" (p.4661, timing) : environment and timing improve engagement; "there is a meeting with the family... figure out what is a good option" (p.4661, triadic consult) : relatives' involvement sustains SDM	Skills; Knowledge; Social Influences; Environmental Context and Resources; Social/Professional Role and Identity; Goals	Doctors → NR; Nurses → schedule family meetings, triadic consultations, communication adaptation, option simplification, timing in calm settings, advocacy and coordination; AHPs → NR; Managers → NR	Doctors=[NR]; Nurses=[Skills; Knowledge; Social Influences; Environmental Context and Resources; Social/Professional Role and Identity; Goals]; AHPs=[NR]; Managers=[NR]
Co-development of BRAIN-TRK: Qualitative examination of acceptability, usability and feasibility of an App to support nurses' care for patients with behavioural and psychological symptoms of neurocognitive disorders in hospital	Redley B; Richardson B; Peel C; Ockerby C; Rawson H; Tomlinson E; Hutchinson A	once they started getting into it, they realised how easy it is : Repeated exposure builds familiarity, showing nurses that navigation and basic functions fit daily practice, p.2875; "it does help the staff and remind them which care suits the patient" : On-screen prompts refresh knowledge and link strategies to individual patient needs and responses, p.2875; "putting the things the patient's like and dislike is really important" : Structured fields for likes and dislikes help nurses quickly individualise care for confused patients, p.2876; "you want to get some suggestions and information from the iPad" : Immediate access to tailored strategy lists at the bedside encourages nurses to consult the App for ideas, p.2876; "that's what we needed, we need basic strategies" : Content pitched at a basic practical level matches nurses' expectations and reduces perceived complexity, p.2876; "I saw it on the app, music can settle them" : Examples in the App normalise trying simple non pharmacological approaches like music for unsettled patients, p.2876	Knowledge; Skills; Beliefs about Consequences; Goals; Memory, Attention and Decision Processes; Environmental Context and Resources; Social Influences; Behavioral Regulation	Doctors=[NR]; Nurses=training and preparatory information, bedside availability of iPads, clear and basic content, tailored strategy suggestions, recording likes and dislikes, peer and facilitator encouragement, visible benefits for managing behavioural and psychological symptoms; AHPs=[NR]; Managers=[NR]	Doctors=[NR]; Nurses=[Knowledge; Skills; Beliefs about Consequences; Goals; Memory, Attention and Decision Processes; Environmental Context and Resources; Social Influences; Behavioural Regulation]; AHPs=[NR]; Managers=[NR]
Working with a robot in hospital and long-term care homes: staff experience	Ren LH; Wong KLY; Wong J; Kleiss S; Berndt A; Mann J; Hussein A; Hu G; Wong L; Khong R; Fu J; Ahmed N; Nolte J; Hung L	After your training, we understand it more (p.8) : Nurses in the hospital focus group say that hands on demonstrations which address privacy and technical worries improve their understanding and willingness to use the robot; "today, I feel better, more comfortable" (p.8) : The same nurse reports feeling more confident to operate the robot safely with patients immediately after trying it during training; "Within 5 minutes you can set it up" (p.12) : Rehabilitation assistants highlight that the quick setup process for calls fits into busy ward workflows and makes it realistic to use robots alongside other tasks; "it is easier than, say, FaceTime" (p.12) : Patient care coordinator compares the simple link or QR based login with more complex tablet logins, describing the robot as the easier option for both staff and families; "staff members can just put it in a room" (p.11) : Staff emphasise that once the robot is positioned in a patient's room, families can drive it and connect directly, so bedside nurses' role is limited to docking and basic checks; "Once staff know that there is something for them to gain" (p.8) : Unit lead describes how gamified training sessions, small rewards and public appreciation make learning enjoyable and motivate staff to engage with and keep using the robot	Knowledge; Skills; Beliefs about Consequences; Goals; Reinforcement; Memory, Attention and Decision Processes; Environmental Context and Resources; Social Influences; Behavioral Regulation	Doctors=[NR]; Nurses=hands on demonstrations, peer champions, easy to follow steps and seeing residents' and families' positive reactions encouraged adoption; AHPs=rehabilitation assistants and social worker used the robot to support therapy sessions and social calls, experienced reduced set up workload compared with tablets and acted as local experts; Managers=clinical leaders recognised alignment with goals of maintaining family connections and endorsed integrating robots into care plans, encouraging staff to participate and helping resolve practical issues	Doctors=[NR]; Nurses=[Knowledge; Skills; Beliefs about Consequences; Goals; Reinforcement; Environmental Context and Resources; Social Influences; Behavioral Regulation]; AHPs=[Knowledge; Skills; Beliefs about Consequences; Environmental Context and Resources; Social Influences]; Managers=[Beliefs about Consequences; Goals; Environmental Context and Resources; Social Influences; Reinforcement]
Agitation near the end of life with dementia: An ethnographic study of care	Sampson EL; Stringer A; La Frenais F; Higgins S; Doyle MJ; Laybourne A; Livingston G; Leavey G	our training tends to enlighten us and equip us with the skills : staff nurse in the teaching hospital describes dementia training as building concrete skills and confidence to calm and support distressed patients, p.7; "our job as a nurse is to give them that assurance, that support, empathy" : nurse frames reassurance and empathy as core professional duties when responding to agitation near the end of life, p.11; agitation was referred to as "distress behaviors" : staff language in Hospital B reframes agitation as signs of anxiety or unmet need, encouraging investigative and compassionate responses instead of blame, p.7; "patients with dementia and agitation often had a dedicated staff member, specials" : assigning one to one staff increases availability to notice triggers, provide company and respond quickly when agitation starts, p.7; "each patient had a forget-me-not magnet on their board" : visible cue on bed boards flags cognitive impairment so staff remember to adapt communication and anticipate distress, p.7; "can we cover you up? Is that comfortable?" : ward sister uses gentle questions, physical comfort and small talk during care to prevent escalation and keep the person settled, p.12	Knowledge; Skills; Social/Professional Role and Identity; Beliefs about Consequences; Goals; Environmental Context and Resources; Social Influences; Emotion; Behavioral Regulation	Doctors: friendly small talk and acknowledgement of agitated patients during ward visits contribute to a calmer atmosphere and support de escalation; Nurses: dementia specific training, reframing agitation as distress behaviour, strong professional identity around reassurance and empathy, and use of comfort, distraction and activity toys to respond to agitation; AHPs: dementia training encouraging an individualised understanding of patients stories and needs, integrating this into how therapy is delivered; Managers: dementia leadership roles, such as the hospital dementia lead and ward sisters who model person centred responses, implement forget me not magnets and five things about me boards, and arrange one to one specials for distressed patients	Doctors=[Social/Professional Role and Identity; Skills]; Nurses=[Knowledge; Skills; Social/Professional Role and Identity; Beliefs about Consequences; Goals; Environmental Context and Resources; Social Influences; Emotion; Behavioral Regulation]; AHPs=[Knowledge; Skills; Social/Professional Role and Identity]; Managers=[Social/Professional Role and Identity; Goals; Environmental Context and Resources; Social Influences; Behavioral Regulation]

Title	Author(s)	Facilitator quotations and granular facilitator descriptions	TDF Domains – Facilitators	Facilitators specific to profession	Facilitators per profession → TDF
Dementia care in acute hospitals: A qualitative study on nurse managers’ perceived challenges and solutions	Scerri A; Innes A; Scerri C	the first thing is to see at a point in time how many people with dementia we have in hospital : collecting local dementia prevalence data to demonstrate demand and support proposals, p.402; "you need numbers, you need statistics" : quantitative evidence seen as necessary to persuade senior management, p.402; "we can easily colour code the wards" : simple environmental colour coding proposed to support orientation and dementia friendliness, p.402; "they must go directly to a medical ward" : fast tracking people with dementia from admission areas to wards suggested to reduce transitions, p.403; "we need to become more empathic and increase in our values" : empathy focused training promoted as a way to shift staff attitudes towards dementia, p.403; "a practice nurse in dementia care" : creating a dementia practice nurse role proposed to support clinical teams and education, p.403 to 404; "Champions have to be identified among staff" : dementia champions drawn from experienced or motivated staff suggested to lead peer learning and change, p.404	Knowledge; Skills; Social/Professional Role and Identity; Beliefs about Consequences; Goals; Environmental Context and Resources; Social Influences; Behavioral Regulation	Doctors → shared care models with geriatricians in some specialties, potential for more collaborative decision making; Nurses → frontline observations about nutrition and risk, potential dementia champions and strong interest in practical training and environmental changes; AHPs → NR; Managers → motivated to act as catalysts for system wide change, advocate for evidence based guidelines, training, better environments and integrated pathways	Doctors=[Social/Professional Role and Identity; Beliefs about Consequences; Goals; Social Influences]; Nurses=[Knowledge; Skills; Social/Professional Role and Identity; Beliefs about Consequences; Goals; Environmental Context and Resources; Social Influences; Behavioral Regulation]; AHPs=[NR]; Managers=[Knowledge; Skills; Social/Professional Role and Identity; Beliefs about Consequences; Goals; Environmental Context and Resources; Social Influences; Behavioral Regulation]
Creating a toolkit with stakeholders for leveraging tablet computers to support person-centred dementia care in hospitals	Shadarevian J; Chan C; Berndt A; Son C; Gregorio M; Horne N; Mann J; Wallsworth C; Chow B; O’Neill R; Hung L	we worked together to set the opera shows for the patient to watch. It made a big difference (p.4) : collaboration with family to personalise tablet content to patient interests improved engagement and reduced loneliness; "I see the iPad can help to reduce the use of restraints and medications" (p.4) : nurse leader belief that tablets can substitute for restraints and sedatives supports adoption; "the key to our success here is we helped everyone understand the benefits, WIIFM" (p.4) : explaining concrete benefits for staff and patients encourages buy in and regular use; "a mechanical arm was introduced which alleviated the weight of the tablet" (p.5, Table 2) : ergonomic hardware solution made tablet use more convenient and safer for staff and patients; "The nurse leader also found that educating staff about the purpose, benefits, and method of operating the tablet reduced inexperience" (p.6) : on ward education by a leader addressed skills gaps and normalised the intervention; "Staff may explore with family to find additional application activities to optimize the tablet use based on their hobbies and interests" (p.5, Table 2) : structured collaboration with families supports meaningful personalisation of tablet activities	Knowledge; Skills; Environmental Context and Resources; Social Influences; Social/Professional Role and Identity; Goals; Beliefs about Consequences; Behavioral Regulation	Doctors=[NR]; Nurses=seeing calmer patients and reduced restraints or medications when tablets are used, valuing simple educational materials, ergonomic hardware and quick reference pocket cards; AHPs=[NR]; Managers=nurse leader championing the intervention, providing education, highlighting benefits and aligning tablet use with person centred care and safety goals	Doctors=[NR]; Nurses=[Knowledge; Skills; Environmental Context and Resources; Social Influences; Goals; Behavioral Regulation; Beliefs about Consequences]; AHPs=[NR]; Managers=[Knowledge; Social Influences; Goals; Environmental Context and Resources; Beliefs about Consequences]
Implementing the PIE (Person, Interaction and Environment) programme to improve person-centred care for people with dementia admitted to hospital wards: a qualitative evaluation	Ann Skingley; Mary Godfrey; Rosemary Henderson; Kim Manley; Rosie Shannon; John Young	We have found that’s really a big, big thing (p.7) : Seeing quiet patients talk and eat at shared mealtimes reinforced the value of changing routines around food and social contact; "the environment is better I think" (p.7) : Adjusting radio use and ward noise after observations showed how small environmental tweaks improved the ward atmosphere; "It’s allowed us to step back and look at what we’re doing" (p.7) : Dedicated observation time created structured reflection space, helping staff review everyday practice more critically; "considerable enthusiasm about being part of a process that actively involved staff" (p.8) : Participatory PIE workshops and cross-ward meetings fostered shared ownership of change and encouraged frontline initiative	Knowledge; Skills; Social/Professional Role and Identity; Beliefs about Capabilities; Beliefs about Consequences; Goals; Memory, Attention and Decision Processes; Environmental Context and Resources; Social Influences; Behavioral Regulation	Doctors=[NR]; Nurses=[NR]; AHPs=[NR]; Managers=[NR]	Doctors=[NR]; Nurses=[NR]; AHPs=[NR]; Managers=[NR]
The barriers and facilitators to implementing dementia education and training in health and social care services: a mixed-methods study	Surr CA; Parveen S; Smith SJ; Drury M; Sass C; Burden S; Oyebode J	everything he talked about made sense because he is going through it : staff in an acute focus group described hearing directly from a person with dementia, which made examples believable and relevant, p.5; "it was not really dry, there were lots of interactive parts to it" : mental health staff compared interactive case based exercises with didactic teaching and felt engagement increased with active methods, p.5; "I have glimpse of how it may be to experience dementia" : staff survey respondent reported experiential simulation gave personal insight into dementia, boosting empathy and practice change, p.5; "Support from Managers and colleagues to reinforce what was learned from training" : survey respondents highlighted that ongoing reminders and encouragement from managers and colleagues help sustain new behaviours, p.6; "it is embedded in supervision and the MDT and the way we work" : ward manager explained how linking dementia principles to routine supervision and multidisciplinary discussions embeds them in daily work, p.6; "The Chief Exec came himself and said this is fantastic what you are doing" : training lead described the chief executive attending dementia events and praising them, signalling organisational priority and support, p.6; "they are really keen to learn, they have a real thirst for knowledge" : ward manager observed that many nurses, especially overseas recruits, are eager to learn, which fuels training uptake, p.7	Knowledge; Skills; Social Influences; Environmental Context and Resources; Goals; Beliefs about Consequences; Reinforcement	Doctors=[NR]; Nurses=[NR]; AHPs=[NR]; Managers=[NR]	Doctors=[NR]; Nurses=[NR]; AHPs=[NR]; Managers=[NR]
Components of impactful dementia training for general hospital staff: a collective case study	Surr CA; Sass C; Burnley N; Drury M; Smith SJ; Parveen S; Burden S; Oyebode J	she gets you up and doing things : interactive exercises during dementia study days keep staff engaged and help them practise new approaches, p.515; "one of the more fun study days" : enjoyable, memorable training increases acceptability and positive talk about dementia education among ward staff, p.515; "you go away being rubbed off with a bit of that passion" : enthusiastic facilitators model valuing dementia care and motivate staff to change practice, p.515; "I’ve tried to like almost show them how I look after a patient with dementia" : ward managers role model person centred care on the ward to embed classroom learning, p.518; "I kept emailing people and telling them the dates" : proactive ward managers remind staff and reassure them about hours back so they prioritise attendance, p.518; "they have to attend a course, it’s mandatory that they attend" : Trust policy makes dementia training compulsory for new nurses, supporting consistent exposure to core content, p.518; "you’ve got to put yourself on people’s doorsteps" : a proactive dementia lead networks with leaders and wards so dementia training stays visible and prioritised, p.519	Knowledge; Skills; Beliefs about Capabilities; Goals; Social Influences; Environmental Context and Resources	Doctors=[NR]; Nurses=[interactive role tailored training and dementia champions support practice change on wards]; AHPs=[NR]; Managers=[supportive ward and unit managers, proactive dementia leads and senior endorsement promote training and implementation]	Doctors=[NR]; Nurses=[Knowledge; Skills; Beliefs about Capabilities]; AHPs=[NR]; Managers=[Social Influences; Environmental Context and Resources; Goals]
Visual identifiers for people with dementia in hospitals: a qualitative study to unravel mechanisms of action for improving quality of care	Sutton E; Armstrong N; Locock L; Conroy S; Tarrant C	could actually see where our patients with dementia were : electronic alerts help hospital and night teams coordinate locations and reduce unnecessary transfers, p.604, Table 2; "immediately assessed by the meaningful activity service" : ED identifiers trigger automatic referral to activity coordinators for additional engagement support, p.604, Table 2; "I’ll take that one first" : x ray staff use identifier cards to fast track people with dementia through imaging, reducing waiting time distress, p.604, Table 2; "this patient is probably going to need quite a lot of assistance" : bedside identifiers help nurses anticipate extra support needs and plan workload during handover, p.604, Table 2; "we have to have more patience with them" : seeing identifiers prompts staff to slow down, adjust communication and explore likes and dislikes, p.604, Table 2; "by engaging them in an activity" : dementia training encourages staff to manage falls risk through meaningful engagement instead of repeated sit down commands, p.605, Table 3; "we’re looking at like coping strategies with staff" : regular debriefing and reflection sessions support staff resilience and better responses to distressed patients, p.605, Table 3	Knowledge; Skills; Goals; Social Influences; Environmental Context and Resources	Doctors=[NR]; Nurses=[use of bedside and electronic identifiers to anticipate additional support needs and adapt communication with patients with dementia]; AHPs=[NR]; Managers=[use of alert systems and flags to coordinate pathways, trigger dementia specific services and embed dementia care within hospital strategies]	Doctors=[NR]; Nurses=[Knowledge; Skills; Environmental Context and Resources]; AHPs=[NR]; Managers=[Goals; Social Influences; Environmental Context and Resources]
A qualitative focus group discussion study on the experiences of Certified Nurses in Dementia Nursing related to effective staff education	Taneichi H; Rokkaku R	I joined patients care willingly to lighten the burden imposed on nurses : DCNs step into direct care during difficult BPSD episodes, reducing nurses immediate workload while modelling dementia sensitive approaches at the bedside, p.550; "I tried to talk with the staff personally or during work" : one to one conversations during shifts give nurses a safe space to share difficulties and receive tailored, case based guidance, p.550; "I planned expanded conferences four times until discharge" : repeated multidisciplinary meetings from admission to discharge build shared understanding and coordinated dementia care plans across hospital and community services, p.550; "At the beginning, I told a nurse to stay behind me and observe me" : DCNs explicitly use role modelling in real cases so nurses can watch concrete dementia care techniques before trying them themselves, p.551; "I carried out a briefing session indicating the effects of the activity program" : DCNs present qualitative and quantitative outcomes of dementia activities to show staff that changed approaches can reduce BPSD and improve engagement, p.551; "I asked a nurse questions such as what is the patients reason not to eat" : guided questioning in specific clinical situations prompts nurses to reflect on causes of behaviour and develop their own problem solving skills in dementia care, p.551	Knowledge; Skills; Beliefs about Consequences; Reinforcement; Environmental Context and Resources; Social Influences; Behavioral Regulation	Doctors=[NR]; Nurses=[respond well to on-the-job coaching, role modelling, structured reflection and visible feedback on dementia care outcomes, gradually gaining confidence to provide dementia care independently]; AHPs=[NR]; Managers=[when managers understand the benefits of dementia care, they support relief systems, staff placement and collaboration that enable DCNs educational work]	Doctors=[NR]; Nurses=[Knowledge; Skills; Beliefs about Consequences; Reinforcement; Social Influences; Behavioral Regulation; Environmental Context and Resources]; AHPs=[NR]; Managers=[Goals; Social Influences; Environmental Context and Resources]
Deconstructing dementia and delirium hospital practice: using cultural historical activity theory to inform education approaches	Teodorczuk A; Mukaetova-Ladinska E; Corbett S; Welfare M	if you have a good leader on that ward you get better quality of care : visible ward leadership sets expectations and supports better dementia and delirium care, FG 01, line 347; where care was good there was a high level of learning and sharing knowledge about the patient : team culture of sharing patient knowledge supports person centred dementia care, Findings activity theory section; I certainly would not allow a doctor to have the final say if I felt my information was as valuable : increased professional confidence enables social workers to contribute psychosocial knowledge in decision making, Int 07, line 718	Knowledge; Beliefs about Capabilities; Social/Professional Role and Identity; Goals; Environmental Context and Resources; Social Influences; Behavioral Regulation	Doctors: awareness of sedation risks and ability to explain reasons for not prescribing when supported can promote safer practice; Nurses: strong ward leadership by senior nurses can model good dementia care and set expectations for the team; AHPs: social workers and other allied staff can develop confidence to speak up, and domestics and occupational therapists hold valuable knowledge about what makes patients tick; Managers: NR	Doctors=[Knowledge; Beliefs about Consequences]; Nurses=[Social Influences; Knowledge; Goals; Behavioral Regulation]; AHPs=[Beliefs about Capabilities; Knowledge; Social/Professional Role and Identity; Social Influences]; Managers=[NR]
The impact of an education intervention on the approach of hospital staff to patients with dementia in a Danish general hospital setting: An explanatory sequential mixed-methods study	Toubøl A; Moestrup L; Thomsen K; Ryg J; Hansen DL; Foldager M; Jakobsen S; Nielsen DS	we now have a common frame of reference among the departments (p.34) : shared knowledge base across departments supports consistent dementia care, staff use the same concepts and language about dementia; "focal point becomes the patient rather than the specific task to be solved" (p.35) : shared values prioritise person over task in ward routines, staff adjust workflows to patient needs; "We are helping each other to avoid merely doing the usual routines" (p.35) : interdisciplinary mutual help and reflection encourage adapting care to individual patients instead of default routines	Knowledge; Social/Professional Role and Identity; Social Influences; Goals; Environmental Context and Resources; Behavioral Regulation	Doctors=[NR]; Nurses=[NR]; AHPs=[NR]; Managers=[NR]	Doctors=[NR]; Nurses=[NR]; AHPs=[NR]; Managers=[NR]
CogChamps: impact of a project to educate nurses about delirium and improve the quality of care for hospitalized patients with cognitive impairment	Travers C; Henderson A; Graham F; Beattie E	they were readily available to support the CogChamps and coach and mentor nurses (p.10) : off line facilitator CogChamps with dedicated time provide on ward support, modelling and opportunistic teaching that help nurses adopt new delirium care behaviours; "Action Plans provided a concrete guide and clear direction for the practice improvement efforts (p.10)" : ward specific Action Plans with explicit tasks, timelines and named leads organise change work and support consistent implementation of cognitive assessment and delirium prevention routines	Knowledge; Skills; Social/Professional Role and Identity; Social Influences; Goals; Environmental Context and Resources; Behavioral Regulation	Doctors=[NR]; Nurses=[CogChamps and facilitator CogChamps model best practice delirium care and provide bedside coaching; clear Action Plans with defined tasks, timelines and responsibility support nurses’ use of cognitive assessments and non pharmacological strategies; executive and Nurse Unit Manager support plus research team mentorship and national campaigns reinforce the importance of cognitive impairment care]; AHPs=[NR]; Managers=[NR]	Doctors=[NR]; Nurses=[Knowledge; Skills; Social/Professional Role and Identity; Social Influences; Goals; Environmental Context and Resources; Behavioral Regulation]; AHPs=[NR]; Managers=[NR]
Care of patients with dementia in an acute trauma and orthopaedics unit	McCorkell G; Harkin D; McCrory V; Lafferty M; Coates V	the manager… supported the research project and encouraged staff (p.46) : leadership endorsement facilitated adoption; "the nursing team made suggestions about what would assist them" (p.46–47) : co-design increased buy-in; "a dementia toolkit was developed to increase awareness and communicate discreetly that a patient has dementia" (p.47) : environmental cueing supported consistent behaviour; "sessions were held at lunchtime or on an ad hoc basis" (p.49–50) : flexible training improved feasibility; "dementia champions… facilitated development of further champions" (p.51) : peer mentoring supported sustainability	Environmental Context and Resources; Social Influences; Knowledge	Doctors=[NR]; Nurses=[NR]; AHPs=[NR]; Managers=[NR]	Doctors=[NR]; Nurses=[NR]; AHPs=[NR]; Managers=[NR]
Caring for people with dementia in hospital: findings from a survey to identify barriers and facilitators to implementing best practice dementia care	Tropea J; LoGiudice D; Liew D; Roberts C; Brand C	supportive environment quiet places natural light (p.473, environment) : orienting environmental cues; "older person friendly cubicles clocks signage" (p.473, cues) : wayfinding aids; "access to expert medical and nursing staff" (p.473, specialists) : specialist support; "staff trained consistent approach" (p.473, consistency) : shared practice norms; "ability to special patients as required" (p.473, staffing) : additional staffing capacity	Environmental Context and Resources; Skills; Social Influences	Doctors=[NR]; Nurses=[NR]; AHPs=[NR]; Managers=[NR]	Doctors=[NR]; Nurses=[NR]; AHPs=[NR]; Managers=[NR]
The use of the truth and deception in dementia care amongst general hospital staff	Turner A; Eccles F; Keady J; Simpson J; Elvish R	I would lie through my teeth and drop it in their tea : Ward sister explains using deception to ensure essential medication is taken in line with perceived patient benefit and professional duty, p.865; "remembering why we are telling these porkies" : Reflecting on reasons for deception helps staff align their communication with what they see as best interests of the person with dementia, p.866; "maintain those boundaries of when to tell that little b" : Informal team boundaries around acceptable deception provide a shared guide for consistent decision making, p.866	Social/Professional Role and Identity; Beliefs about Consequences; Goals; Behavioral Regulation	Doctors: NR; Nurses: Use of professional ethical principles and informal boundaries, plus pragmatic use of deception for medication and risk reduction, provides some decision clarity; AHPs: NR; Managers: NR	Doctors=[NR]; Nurses=[Social/Professional Role and Identity; Beliefs about Consequences; Goals; Behavioral Regulation]; AHPs=[NR]; Managers=[NR]

Title	Author(s)	Facilitator quotations and granular facilitator descriptions	TDF Domains – Facilitators	Facilitators specific to profession	Facilitators per profession → TDF
Nurses' Perspectives and Experiences of Using a Bed-Exit Information System in an Acute Hospital Setting: Mixed Methods Study	Walzer S; Schön I; Pfeil J; Klemm S; Ziegler S; Schmoor C; Kunze C	if this [BES] is of any help, great : Nurses are open to using BES as partial support when sitters or extra staff are unavailable, p.8; "still very lively, very mobile, agile, he gets a mat" : Nurses target BES to mobile, cognitively impaired patients with higher unsupervised bed exit risk, p.8; "they also went into the room more quickly at night because they knew there was a mat" : Bed exit alerts prompt earlier checks on at risk patients at night, p.8; "I put that in once, plug it in and then it's there" : Simple setup lets nurses incorporate BES alongside other technical devices in routine care, p.8; "it does prevent falls in some cases" : Observed fall prevention reinforces perceived usefulness of BES for selected patients, p.8; "it rings and I run and it doesn't rumble" : Early warning allows nurses to respond before a fall occurs, increasing sense of control and safety, p.8	Social/Professional Role and Identity; Beliefs about Consequences; Goals; Environmental Context and Resources; Behavioral Regulation	Doctors: [NR]; Nurses: See BES as one useful tool to improve safety for specific mobile, cognitively impaired patients, especially at night; value earlier warnings, physical and psychological relief, and relatively easy integration into routine; AHPs: [NR]; Managers: [NR]	Doctors=[NR]; Nurses=[Social/Professional Role and Identity; Beliefs about Consequences; Goals; Environmental Context and Resources; Behavioral Regulation]; AHPs=[NR]; Managers=[NR]
Junior doctor dementia champions in a district general hospital (innovative practice)	Wilkinson I; Coates A; Merrick S; Lee C	the initial doctors' enthusiasm and the sense of a group : peer enthusiasm and sense of a group coming together encouraged others to join the champion scheme, p.4; "it would be nice to have a bit more knowledge" : desire to gain dementia knowledge and skills motivated engagement in the champion role, p.5; "becoming a dementia champion made us enthusiastic about doing it" : having a defined champion role and responsibility for audit increased motivation to complete dementia assessments consistently, p.5; "with every single patient you keep exactly the same routine" : embedding cognitive assessment into a standard clerking routine supported consistent dementia screening, p.5; "I think it has made me a better doctor" : perceived professional growth reinforced commitment to dementia friendly practices, p.6; "I am much more confident in talking to patients' families now" : improved communication skills with relatives encouraged ongoing involvement in dementia care, p.6; "I can almost not imagine going to another hospital where they don't have this sort of set-up" : champions came to view dementia schemes as part of normal good practice, raising expectations in future workplaces, p.6; "we want to try and present this, we want to try and write it up" : opportunities for presentation and publication provided career incentives that supported ongoing engagement, p.7; "We have founded a Junior Doctors Forum with great connections with the Trust management" : a junior doctors forum with management connections enabled doctors to influence wider hospital practice and support service improvement work, p.4; "Dr XX's influence has been huge" : visible consultant support and framing dementia assessment as what every patient should have normalised dementia friendly routines, p.6	Knowledge; Skills; Social/Professional Role and Identity; Beliefs about Capabilities; Goals; Social Influences; Environmental Context and Resources; Reinforcement; Behavioral Regulation; Optimism	Doctors : prior personal experiences with dementia motivating improvement; peer led dementia champion group and sense of community; supportive consultant lead who validates dementia friendly work; dementia assessment embedded into routine clerking; opportunities to develop communication skills, leadership and quality improvement experience through the champion role, junior doctor forums and conference presentations; Nurses : NR; AHPs : NR; Managers : NR	Doctors=[Knowledge; Skills; Social/Professional Role and Identity; Beliefs about Capabilities; Goals; Social Influences; Environmental Context and Resources; Reinforcement; Behavioral Regulation; Optimism]; Nurses=[NR]; AHPs=[NR]; Managers=[NR]
Nurses' Experiences in Caring for Older Adults With Responsive Behaviors of Dementia in Acute Care	Yous ML; Ploeg J; Kaasalainen S; Schindel Martin L	they share a different way about the patient : Input from physiotherapists and occupational therapists broadens understanding of the patient and supports joint care planning, p.8; "talking to their family or friends is the easiest thing" : Family knowledge about the person provides quick practical information that helps staff tailor responses to behaviours, p.8; "I love playing music for patients" : Simple person centred activities like preferred music create a calming familiar atmosphere that can ease responsive behaviours, p.8; "We all help him, it is much easier for me" : Shared ownership of patients with dementia makes challenging situations more manageable for individual nurses, p.9; "these patients belonging to everyone" : A shared team view that all staff are responsible for dementia care supports collective action and mutual support, p.9; "they are used to my familiar face" : Continuity with familiar nurses helps patients follow routines and accept care more easily, p.9; "the behavioural rounds might help more" : Structured team meetings focused on behaviours allow staff to share ideas and agree consistent care plans, p.9; "having them more frequently on the floor might be more helpful" : Increased BSO presence on the unit would give nurses timely specialist advice for managing responsive behaviours, p.10; "Add additional staffing, educated staffing" : Having more staff who are specifically trained in dementia care is seen as central to improving support for these patients, p.10; "maybe the opportunity for everyone to take PIECES and or GPA" : Wider access to dementia specific education is expected to strengthen staff knowledge and skills in managing behaviours, p.11	Social Influences; Environmental Context and Resources; Knowledge; Skills	Doctors=[NR]; Nurses=[strong interprofessional collaboration and shared ownership lighten the load of complex dementia care, family involvement continuity of care and behavioural rounds help staff coordinate person centred responses, increased BSO presence more dementia trained staff and regular refresher education are viewed as key supports]; AHPs=[family history and life story work help tailor nonpharmacological approaches, interdisciplinary meetings and recommending GPA or PIECES training are seen as ways to strengthen ward dementia care]; Managers=[NR]	Doctors=[NR]; Nurses=[Social Influences; Environmental Context and Resources; Knowledge; Skills; Goals]; AHPs=[Social Influences; Environmental Context and Resources; Knowledge]; Managers=[NR]
Assessing the Attitudes of Greek Nurses Toward Computerized Dementia Screening	Stelios Zygouris; Mara Gkioka; Despina Moraitou; Birgit Teichmann; Thrasyvoulos Tsiatsos; Sotirios Papagiannopoulos; Magda Tsolaki	Computer literacy and familiarity lead to better attitudes toward technology and support technology acceptance : existing ICT skills and regular computer use among nurses encourage openness and confidence about computerized dementia screening, p.1577; "The attitudes of nurses toward computerized dementia screening, in our study sample, are overall positive" : strong baseline support among experienced nurses increases likelihood that computerized screening will be welcomed if offered, p.1580; "Both feasibility and acceptability scores are high" : nurses judge computerized dementia screening as both workable in principle and acceptable in their clinical role, p.1580; "The most common answer to Question 4 was '10–20 minutes'" : many nurses feel they can realistically allocate 10–20 minutes per older patient for computerized screening during a shift, p.1579; "The most common answer to Question 3 was '1–2 days'" : nurses report they can devote one to two full days to initial training in computerized dementia screening, p.1579; "The findings of this study support the implementation of computerized dementia screening in public hospitals" : positive attitudes plus manageable time commitments suggest implementation is feasible once equipment and training barriers are addressed, p.1582	Knowledge; Skills; Goals; Environmental Context and Resources; Beliefs about Consequences; Optimism	Doctors=[NR]; Nurses=positive attitudes toward computerized dementia screening, perceived feasibility and acceptability, willingness to devote time for training and 10–20 minute tests, recognition that ICT training and computer literacy can improve acceptance; AHPs=[NR]; Managers=[NR]	Doctors=[NR]; Nurses=[Knowledge; Skills; Goals; Environmental Context and Resources; Beliefs about Consequences; Optimism]; AHPs=[NR]; Managers=[NR]
