# Supplementary material for: Advancing dementia-friendly hospital care: a systematic review of healthcare professionals’ reported barriers and facilitators using the theoretical domains framework
Source: Age Ageing. 2026 Sep 22;55(9):afag285. doi: 10.1093/ageing/afag285 (PMC13596736; doi:10.1093/ageing/afag285)
Supplement: Supplementary file 1 [file supplementary_material_afag285.zip › aa-26-1308-File006.pdf]

Advancing Dementia-Friendly Hospital Care: A Systematic Review of Healthcare Professionals' Reported Barriers and Facilitators Using the Theoretical Domains Framework

Appendix 5. The Theoretical Domains Framework (TDF) with definitions and component constructs

TDF domain	Definition	Component constructs
**1. Knowledge**	An awareness of the existence of something	Knowledge (including knowledge of conditions/scientific rationale)<br>Procedural knowledge<br>Knowledge of task environment
**2. Skills**	An ability or proficiency acquired through practice	Skills<br>Skills development<br>Competence<br>Ability<br>Interpersonal skills<br>Practice<br>Skill assessment
**3. Social/Professional Role and Identity**	A coherent set of behaviors and displayed personal qualities of an individual in a social or work setting	Professional identity<br>Professional role<br>Social identity<br>Identity<br>Professional boundaries<br>Professional confidence<br>Group identity<br>Leadership

		Organizational commitment
**4. Beliefs about Capabilities**	Acceptance of the truth, reality or validity about an ability, talent or facility that a person can put to constructive use	Self-confidence<br>Perceived competence<br>Self-efficacy<br>Perceived behavioral control<br>Beliefs<br>Self-esteem<br>Empowerment<br>Professional confidence
**5. Optimism**	The confidence that things will happen for the best or that desired goals will be attained	Optimism<br>Pessimism<br>Unrealistic optimism<br>Identity
**6. Beliefs about Consequences**	Acceptance of the truth, reality or validity about outcomes of a behavior in a given situation	Beliefs<br>Outcome expectancies<br>Characteristics of outcome expectancies<br>Anticipated regret<br>Consequents
**7. Reinforcement**	Increasing the probability of a response by arranging a dependent relationship, or contingency, between the response and a given stimulus	Rewards (proximal/distal, valued/not valued, probable/improbable)<br>Incentives<br>Punishment<br>Consequents<br>Reinforcement

		Contingencies<br>Sanctions
**8. Intentions**	A conscious decision to perform a behavior or a resolve to act in a certain way	Stability of intentions<br>Stages of change model<br>Trans-theoretical model and stages of change
**9. Goals**	Mental representations of outcomes or end states that an individual wants to achieve	Goals (distal/proximal)<br>Goal priority<br>Goal/target setting<br>Goals (autonomous/controlled)<br>Action planning<br>Implementation intention
**10. Memory, Attention and Decision Process**	The ability to retain information, focus selectively on aspects of the environment and choose between two or more alternatives	Memory<br>Attention<br>Attention control<br>Decision making<br>Cognitive overload/tiredness
**11. Environmental Context & Resources**	Any circumstance of a person's situation or environment that discourages or encourages the development of skills and abilities, independence, social competence and adaptive behavior	Environmental stressors<br>**Resources**/material resources<br>Organizational **culture**/climate<br>Salient events/**critical incidents**<br>Person × environment **interaction**<br>Barriers and facilitators
**12. Social Influences**	Those interpersonal processes that can cause individuals to change their thoughts, feelings or behaviors	Social pressure<br>Social norms<br>Group conformity<br>Social comparisons

		Group norms<br>Social support<br>Power<br>Intergroup conflict<br>Alienation<br>Group identity<br>Modelling
**13. Emotion**	A complex reaction pattern, involving experiential, behavioral, and physiological elements, by which the individual attempts to deal with a personally significant matter or event	Fear<br>Anxiety<br>Affect<br>Stress<br>Depression<br>Positive/negative affect<br>Burn-out
**14. Behavioral Regulation**	Anything aimed at managing or changing objectively observed or measured actions.	Self-monitoring<br>Breaking habit<br>Action planning

TDF = Theoretical Domains Framework

Reference:

Atkins L, Francis J, Islam R, O'Connor D, Patey A, Ivers N, et al. A guide to using the Theoretical Domains Framework of behaviour change to investigate implementation problems. Implement Sci. 2017;12(1):77
