# Supplementary material for: Advancing dementia-friendly hospital care: a systematic review of healthcare professionals’ reported barriers and facilitators using the theoretical domains framework
Source: Age Ageing. 2026 Sep 22;55(9):afag285. doi: 10.1093/ageing/afag285 (PMC13596736; doi:10.1093/ageing/afag285)
Supplement: Supplementary file 1 [file supplementary_material_afag285.zip › aa-26-1308-File007.pdf]

Advancing Dementia-Friendly Hospital Care: A Systematic Review of Healthcare Professionals’ Reported Barriers and Facilitators Using the Theoretical Domains Framework

Appendix 6. Study-level Mixed Methods Appraisal Tool assessments

Supplementary Data

Study-level Mixed Methods Appraisal Tool assessments

Title	Author(s)	Year	1.1_ QualApproach	1.2_ QualDataCollection	1.3_ QualFindings	1.4_ QualInterpretation	1.5_ QualCoherence	3.1_ NonRandRepresentative	3.2_ NonRandMeasurements	3.3_ NonRandCompleteData	3.4_ NonRandConfounders	3.5_ NonRandIntervention	4.1_ Descriptive Sampling	4.2_ Descriptive Representativeness	4.3_ Descriptive Measurements	4.4_ Descriptive NonresponseBias	4.5_ Descriptive Statistics	5.1_ MM_ Rationale	5.2_ MM_ Integration	5.3_ MM_ Interpretation	5.4_ MM_ Divergences	5.5_ MM_ QualityAdherence	MMAT descriptive rating (0-5 stars)
Facilitators and barriers to implementing a specialized care unit for persons with cognitive impairment in an acute geriatric hospital: a process evaluation	Adlbrecht L; Karrer M; Helfenberger N; Ziegler E; Zeller A	2024	1	1	1	1	1	NA	NA	NA	NA	NA	NA	NA	NA	NA	NA	NA	NA	NA	NA	NA	★★★★★
Person-Centred Dementia Care in an Acute Hospital: Experiences from Nurses Working in a Specialized Dementia Unit	Cheong CY; Tay FHE; Choo J; Yap PLK	2021	1	1	1	1	1	NA	NA	NA	NA	NA	NA	NA	NA	NA	NA	NA	NA	NA	NA	NA	★★★★★
Prevention and management of behavioural and psychological symptoms in patients with dementia in acute care: a best practice implementation project	Rachid Akrour; Catherine Courret-Gilgen; Beatrice Perrenoud	2022	NA	NA	NA	NA	NA	0	1	1	0	0	NA	NA	NA	NA	NA	NA	NA	NA	NA	NA	★★
Understanding and identifying ways to improve hospital-based cancer care and treatment for people with dementia: an ethnographic study	Laura Ashley; Rachael Kelley; Alys Griffiths; Fiona Cowdell; Ann Henry; Hayley Inman; June Hennell; Margaret Ogden; Maria Walsh; Liz Jones; Ellen Mason; Michelle Collinson; Amanda Farrin; Claire Surr	2021	1	1	1	1	1	NA	NA	NA	NA	NA	NA	NA	NA	NA	NA	NA	NA	NA	NA	NA	★★★★★
Implementing an intervention to enhance care delivery and consistency for people with hip fracture and cognitive impairment in acute hospital wards: a mixed methods process evaluation of a randomised controlled feasibility trial (PERFECTED)	Backhouse T; Fox C; Hammond SP; Poland F; McDermott-Thompson V; Penhale B; Cross JL	2023	1	1	1	1	1	NA	NA	NA	NA	NA	0	0	0	0	1	1	1	1	1	0	★★★
Implementation of STAR-VA for behavioral symptoms of dementia in acute care: Lessons learned	Hannah M. Bashian; Julia T. Boyle; Seneca Correa; Jane Driver; Caroline Madrigal; Isabel Desroches; Mackenzie Farrell; Olivia Eiten; Katie Flanagan; Talya Shahal; Kelly A. O'Malley	2024	NA	NA	NA	NA	NA	NA	NA	NA	NA	NA	1	0	1	0	1	NA	NA	NA	NA	NA	★★★
Dementia case-finding in hospitals: a qualitative study exploring the views of healthcare professionals in English primary care and secondary care	Anne-Marie Burn; Jane Fleming; Carol Brayne; Chris Fox; Frances Bunn	2018	1	1	1	1	1	NA	NA	NA	NA	NA	NA	NA	NA	NA	NA	NA	NA	NA	NA	NA	★★★★★
An Action Research Approach to Implementing a Program for Sharing Meaningful Moments With Hospitalized Patients Living With Dementia	Esther Coker; Joanna Sue; Liz Mersereau; Patricia Hewston; Caroline Marr; Sophia Matarazzo; Afeef Vehra; Alexandra Papaioannou	2025	1	1	1	1	1	NA	NA	NA	NA	NA	1	0	1	0	1	1	1	1	1	0	★★★★
Disjunctures in practice: ethnographic observations of orthopaedic ward practices in the care of older adults with hip fracture and presumed cognitive impairment	Jane L. Cross; Tamara Backhouse; Simon P. Hammond; Bridget Penhale; Fiona Scheibl; Nigel Lambert; Anna Varley; Chris Fox; Fiona M. Poland	2022	1	1	1	1	1	NA	NA	NA	NA	NA	NA	NA	NA	NA	NA	NA	NA	NA	NA	NA	★★★★★
Patient safety for people experiencing advanced dementia in hospital: A video reflexive ethnography	Ann Dadich; Jade Rodrigues; Anita De Bellis; Annmarie Hosie; Tamsin Symonds; Justin Prendergast; Alan Bevan; Aileen Collier	2023	1	1	1	1	1	NA	NA	NA	NA	NA	NA	NA	NA	NA	NA	NA	NA	NA	NA	NA	★★★★★
Dementia, medication and transitions of care	Louise S. Deeks; Gabrielle M. Cooper; Brian Draper; Susan Kurrle; Diane M. Gibson	2016	1	1	1	1	1	NA	NA	NA	NA	NA	NA	NA	NA	NA	NA	NA	NA	NA	NA	NA	★★★★★
Using sense-making theory to aid understanding of the recognition, assessment and management of pain in patients with dementia in acute hospital settings	Dawn Dowding; Valentina Lichtner; Nick Allcock; Michelle Briggs; Kirstin James; John Keady; Reena Lasrado; Elizabeth L. Sampson; Caroline Swarbrick; S. José Closs	2016	1	1	1	1	1	NA	NA	NA	NA	NA	NA	NA	NA	NA	NA	NA	NA	NA	NA	NA	★★★★★
Exploring dementia care in acute care settings: Perspectives of nurses and social workers on caring for patients with behavioral and psychological symptoms	Ruth E. Dunkle; Katherine Cavignini; Joonyoung Cho; Laura Sutherland; Helen Kales; Cathleen Connell; Amanda Leggett	2022	1	1	1	1	1	NA	NA	NA	NA	NA	NA	NA	NA	NA	NA	NA	NA	NA	NA	NA	★★★★★
Using Signs and Symbols to Label Hospital Patients with a Dementia Diagnosis: Help or Hindrance to Care?	Katie Featherstone; Paula Boddington; Andy Northcott	2020	1	1	1	1	1	NA	NA	NA	NA	NA	NA	NA	NA	NA	NA	NA	NA	NA	NA	NA	★★★★★
Developing a Life Story Intervention for Older Adults With Dementia or at Risk of Delirium Who Were Hospitalized: Multistage, Stakeholder-Engaged Co-Design Study	Sarah J. Flessa; James D. Harrison; Roniela Turnigan; Megan Rathfon; Michael Chandler; Jay Newton-Small; Stephanie E. Rogers	2024	1	1	1	1	1	NA	NA	NA	NA	NA	1	1	1	1	1	1	1	1	1	1	★★★★★
Issues experienced while administering care to patients with dementia in acute care hospitals: A study based on focus group interviews	Risa Fukuda; Yasuko Shimizu; Natsuko Seto	2015	1	1	1	1	1	NA	NA	NA	NA	NA	NA	NA	NA	NA	NA	NA	NA	NA	NA	NA	★★★★★
Effects of a Person Centered Dementia Training Program in Greek Hospital Staff: Implementation and Evaluation	Mara Gkioka; Birgit Teichmann; Despina Moraitou; Sotirios Papagiannopoulos; Magda Tsolaki	2020	1	1	1	1	1	0	1	0	0	1	NA	NA	NA	NA	NA	1	1	1	0	0	★★★
The Person, Interactions and Environment Programme to improve care of people with dementia in hospital: a multisite study	Godfrey M; Young J; Shannon R; Skingley A; Woolley R; Arrojo F; Brooker D; Manley K; Surr C	2018	1	1	1	1	1	NA	NA	NA	NA	NA	1	0	1	0	1	1	1	1	0	0	★★★★
How do emergency department staff respond to behaviour that challenges displayed by people living with dementia? A mixed-methods study	Goodwin L, Liddiard C, Manning S, Benger JR, Carlton E, Cheston R, Hoskins R, Taylor H, Voss S	2023	1	1	1	1	1	NA	NA	NA	NA	NA	1	0	1	0	1	1	1	1	1	0	★★★★
Balancing the needs of individuals and services in cancer treatment for people with dementia: A focused ethnographic study	Alys Wyn Griffiths; Laura Ashley; Rachael Kelley; Fiona Cowdell; Michelle Collinson; Ellen Mason; Hayley Inman; Ann Henry; Amanda Farrin; Claire Surr	2021	1	1	1	1	1	NA	NA	NA	NA	NA	NA	NA	NA	NA	NA	NA	NA	NA	NA	NA	★★★★★
Supporting general hospital staff to provide dementia sensitive care: A realist evaluation	Melanie Handley; Frances Bunn; Claire Goodman	2019	1	1	1	1	1	NA	NA	NA	NA	NA	NA	NA	NA	NA	NA	NA	NA	NA	NA	NA	★★★★★
Exploring ways to enhance pain management for older people with dementia in acute care settings using a Participatory Action Research approach	Deirdre Harkin; Vivien Coates; Donna Brown	2022	1	1	1	1	1	NA	NA	NA	NA	NA	NA	NA	NA	NA	NA	NA	NA	NA	NA	NA	★★★★★
Barriers and enablers to implementing 'DEALTS2' simulation-based train-the-trainer dementia training programme in hospital settings across England: a qualitative study	Michelle Heward; Michele Board; Ashley Spriggs; Dina Blagden; Jane Murphy	2021	1	1	1	1	1	NA	NA	NA	NA	NA	NA	NA	NA	NA	NA	NA	NA	NA	NA	NA	★★★★★
Person-centred oral hydration care for older people with dementia admitted to acute hospital wards: Empirical research qualitative	Shanlee Higgins; Lesley Baillie; Calvin Moorley; Fiona Nolan	2023	1	1	1	1	1	NA	NA	NA	NA	NA	NA	NA	NA	NA	NA	NA	NA	NA	NA	NA	★★★★★
The experience of hospital staff in applying the Gentle Persuasive Approaches to dementia care	Lillian Hung; Cathy Son; Rebecca Hung	2019	1	1	1	1	1	NA	NA	NA	NA	NA	1	0	1	1	1	1	1	1	1	0	★★★★
Nurses' knowledge and beliefs on pain management practices with hospitalised persons living with dementia: A qualitative descriptive study	Beverly Ingelson; Sherry Dahlke; Hannah O'Rourke; Kathleen F. Hunter	2024	1	1	1	1	1	NA	NA	NA	NA	NA	NA	NA	NA	NA	NA	NA	NA	NA	NA	NA	★★★★★
Living with dementia in hospital wards: a comparative study of staff perceptions of practice and observed patient experience	Innes A., Kelly F., Scerri C., Abela S.	2016	1	1	1	1	1	NA	NA	NA	NA	NA	1	0	1	0	1	1	1	1	1	0	★★★★
Barriers and facilitators to caring for patients living with dementia in the emergency department: a qualitative study	Jelinski D., Reich K., Lang E., Holroyd-Leduc J., Goodarzi Z.	2025	1	1	1	1	1	NA	NA	NA	NA	NA	NA	NA	NA	NA	NA	NA	NA	NA	NA	NA	★★★★★
Nurses' experiences of delivering acute orthopaedic care to patients with dementia	Jensen AM; Pedersen BD; Wilson RL; Olsen RB; Hounsgaard L	2019	1	1	1	1	1	NA	NA	NA	NA	NA	NA	NA	NA	NA	NA	NA	NA	NA	NA	NA	★★★★★
Health professional perspectives on systems failures in transitional care for patients with dementia and their carers: a qualitative descriptive study	Kable A; Chenoweth L; Pond D; Hullick C	2015	1	1	1	1	1	NA	NA	NA	NA	NA	NA	NA	NA	NA	NA	NA	NA	NA	NA	NA	★★★★★
Nurses' perceptions in caring for people with dementia in Dutch acute hospitals	Annette Keuning-Plantinga; Petrie F. Roodbol; Wim P. Krijnen; Evelyn J. Finnema	2022	NA	NA	NA	NA	NA	NA	NA	NA	NA	NA	0	0	1	0	1	NA	NA	NA	NA	NA	★★
Radiographers' experiences in working with patients living with dementia in Norway: A qualitative study	E. Kjelle; C. Eikefet; C.C. Chilanga	2024	1	1	1	1	1	NA	NA	NA	NA	NA	NA	NA	NA	NA	NA	NA	NA	NA	NA	NA	★★★★★
Perioperative Nurses' Experience of Working With Persons Living With Dementia	Mariya Kovaleva; Abigail C. Jones; Cornelia Frances Garrard; Jana Lauderdale; Mary S. Dietrich; Peace Johnson; Cathy Maxwell	2025	1	1	1	1	1	NA	NA	NA	NA	NA	NA	NA	NA	NA	NA	NA	NA	NA	NA	NA	★★★★★
Implementation of a model of care for hospitalised older persons with cognitive impairment (the Confused Hospitalised Older Persons program) in six New South Wales hospitals	Susan Kurrle; Sigrid Patterson; Cath Bateman; Anthea Temple; Anne Cumming; Glen Pang	2019	1	0	0	0	1	1	1	1	0	0	NA	NA	NA	NA	NA	1	0	0	0	0	★★
Redesigning acute care for cognitively impaired older adults: Optimizing health care services	Michael A LaMantia; Malaz A Boustani; Shola Jhanji; Mungai Maina; Arif Nazir; Frank C Messina; Amie Frame; Catherine Alder; Joshua Chodosh	2016	1	1	1	1	1	NA	NA	NA	NA	NA	NA	NA	NA	NA	NA	NA	NA	NA	NA	NA	★★★★★

Title	Author(s)	Year	1.1_ QualApproach	1.2_ QualDataCollection	1.3_ QualFindings	1.4_ QualInterpretation	1.5_ QualCoherence	3.1_ NonRandRepresentative	3.2_ NonRandMeasurements	3.3_ NonRandCompleteData	3.4_ NonRandConfounders	3.5_ NonRandIntervention	4.1_ DescriptiveSampling	4.2_ DescriptiveRepresentativeness	4.3_ DescriptiveMeasurements	4.4_ DescriptiveNonresponseBias	4.5_ DescriptiveStatistics	5.1_ MM_ Rationale	5.2_ MM_ Integration	5.3_ MM_ Interpretation	5.4_ MM_ Divergences	5.5_ MM_ QualityAdherence	MMAT descriptive rating (0-5 stars)
Dementia care across a tertiary-care health system: What exists now and what needs to change	Amanda Leggett; Cathleen Connell; Leslie Dubin; Ruth Dunkle; Kenneth M. Langa; Donovan T. Maust; J. Scott Roberts; Beth Spencer; Helen C. Kales	2020	1	1	1	1	1	NA	NA	NA	NA	NA	NA	NA	NA	NA	NA	NA	NA	NA	NA	NA	★★★★★
Understanding and integrating the needs and preferences of people living with dementia in the inpatient setting: a qualitative study	Alyse Lennox; Denise Goodwin; Felicity Leavold; Renae Nicol; Velandai Srikanth; Darshini Ayton; Madeleine Berends; Myra Thiessen; Daphne Flynn; Chris Moran	2025	1	1	1	1	1	NA	NA	NA	NA	NA	NA	NA	NA	NA	NA	NA	NA	NA	NA	NA	★★★★★
The assessment and management of pain in patients with dementia in hospital settings: a multi-case exploratory study from a decision making perspective	Valentina Lichtner; Dawn Dowding; Nick Allcock; John Keady; Elizabeth L. Sampson; Michelle Briggs; Anne Corbett; Kirstin James; Reena Lasrado; Caroline Swarbrick; S. José Closs	2016	1	1	1	1	1	NA	NA	NA	NA	NA	NA	NA	NA	NA	NA	NA	NA	NA	NA	NA	★★★★★
What influences decisions about ongoing stroke rehabilitation for patients with pre-existing dementia or cognitive impairment: a qualitative study	Longley V; Bowen A; Peters S; Swarbrick C	2018	1	1	1	1	1	NA	NA	NA	NA	NA	NA	NA	NA	NA	NA	NA	NA	NA	NA	NA	★★★★★
Facilitators, Barriers, and Solutions in Pain Management for Older Adults with Dementia	Alícia Minaya-Freire; Anna Ramon-Aribau; Gemma Pou-Pujol; Meritxell Fajula-Bonet; Mireia Subirana-Casacuberta	2020	1	1	1	1	1	NA	NA	NA	NA	NA	NA	NA	NA	NA	NA	NA	NA	NA	NA	NA	★★★★★
Privileges, and Permissions: Theorising Intersectionality and Cultures of Control in the Care of People Living With Dementia in Acute Hospital Settings	Mwale S; Northcott A; Featherstone K	2025	1	1	1	1	1	NA	NA	NA	NA	NA	NA	NA	NA	NA	NA	NA	NA	NA	NA	NA	★★★★★
When people living with dementia say 'no': Negotiating refusal in the acute hospital setting	O'Brien R; Beeke S; Pilnick A; Goldberg SE; Harwood RH	2020	1	1	1	1	1	NA	NA	NA	NA	NA	NA	NA	NA	NA	NA	NA	NA	NA	NA	NA	★★★★★
"Getting to Know Them": Person-Centered Care for Patients With Dementia in Acute Care	Osuoha P; Masoud SS; Leibas M; Cleveland LM; Reed CC; Piernik-Yoder B; White CL	2021	1	1	1	1	1	NA	NA	NA	NA	NA	NA	NA	NA	NA	NA	NA	NA	NA	NA	NA	★★★★★
Improving emotional wellbeing for hospital-based patients with dementia	Petty, S., Griffiths, A., Coleston, D.M. & Dening, T.	2021	1	1	1	1	1	NA	NA	NA	NA	NA	NA	NA	NA	NA	NA	NA	NA	NA	NA	NA	★★★★★
Nurses' perspectives on shared decision-making in the daily care of hospitalized patients with dementia: An exploratory qualitative study	Plantinga A; Roodbol PF; van Munster BC; Finnema EJ	2024	1	1	1	1	1	NA	NA	NA	NA	NA	NA	NA	NA	NA	NA	NA	NA	NA	NA	NA	★★★★★
Co-development of BRAIN-TRK: Qualitative examination of acceptability, usability and feasibility of an App to support nurses' care for patients with behavioural and psychological symptoms of neurocognitive disorders in hospital	Redley B; Richardson B; Peel C; Ockerby C; Rawson H; Tomlinson E; Hutchinson A	2019	1	1	1	1	1	NA	NA	NA	NA	NA	1	0	1	0	1	1	1	1	1	0	★★★★
Working with a robot in hospital and long-term care homes: staff experience	Ren LH; Wong KLY; Wong J; Kleiss S; Berndt A; Mann J; Hussein A; Hu G; Wong L; Khong R; Fu J; Ahmed N; Nolte J; Hung L	2024	1	1	1	1	1	NA	NA	NA	NA	NA	NA	NA	NA	NA	NA	NA	NA	NA	NA	NA	★★★★★
Agitation near the end of life with dementia: An ethnographic study of care	Sampson EL; Stringer A; La Frenais F; Higgins S; Doyle MJ; Laybourne A; Livingston G; Leavey G	2019	1	1	1	1	1	NA	NA	NA	NA	NA	1	0	1	0	1	1	1	1	0	0	★★★★
Dementia care in acute hospitals: A qualitative study on nurse managers' perceived challenges and solutions	Scerri A; Innes A; Scerri C	2020	1	1	1	1	1	NA	NA	NA	NA	NA	NA	NA	NA	NA	NA	NA	NA	NA	NA	NA	★★★★★
Creating a toolkit with stakeholders for leveraging tablet computers to support person-centred dementia care in hospitals	Shadarevian J; Chan C; Berndt A; Son C; Gregorio M; Horne N; Mann J; Wallsworth C; Chow B; O'Neill R; Hung L	2020	1	1	1	1	1	NA	NA	NA	NA	NA	NA	NA	NA	NA	NA	NA	NA	NA	NA	NA	★★★★★
Implementing the PIE (Person, Interaction and Environment) programme to improve person-centred care for people with dementia admitted to hospital wards: a qualitative evaluation	Ann Skingley; Mary Godfrey; Rosemary Henderson; Kim Manley; Rosie Shannon; John Young	2021	1	1	1	1	1	NA	NA	NA	NA	NA	NA	NA	NA	NA	NA	NA	NA	NA	NA	NA	★★★★★
The barriers and facilitators to implementing dementia education and training in health and social care services: a mixed-methods study	Surr CA; Parveen S; Smith SJ; Drury M; Sass C; Burden S; Oyebode J	2020	1	1	1	1	1	NA	NA	NA	NA	NA	1	0	1	0	1	1	1	1	1	0	★★★★
Components of impactful dementia training for general hospital staff: a collective case study	Surr CA; Sass C; Burnley N; Drury M; Smith SJ; Parveen S; Burden S; Oyebode J	2018	1	1	1	1	1	NA	NA	NA	NA	NA	1	0	1	0	1	1	1	1	1	0	★★★★
Visual identifiers for people with dementia in hospitals: a qualitative study to unravel mechanisms of action for improving quality of care	Sutton E; Armstrong N; Locock L; Conroy S; Tarrant C	2023	1	1	1	1	1	NA	NA	NA	NA	NA	NA	NA	NA	NA	NA	NA	NA	NA	NA	NA	★★★★★
A qualitative focus group discussion study on the experiences of Certified Nurses in Dementia Nursing related to effective staff education	Taneichi H; Rokkaku R	2020	1	1	1	1	1	NA	NA	NA	NA	NA	NA	NA	NA	NA	NA	NA	NA	NA	NA	NA	★★★★★
Deconstructing dementia and delirium hospital practice: using cultural historical activity theory to inform education approaches	Teodorczuk A; Mukaetova-Ladinska E; Corbett S; Welfare M	2015	1	1	1	1	1	NA	NA	NA	NA	NA	NA	NA	NA	NA	NA	NA	NA	NA	NA	NA	★★★★★
The impact of an education intervention on the approach of hospital staff to patients with dementia in a Danish general hospital setting: An explanatory sequential mixed-methods study	Toubøl A; Moestrup L; Thomsen K; Ryg J; Hansen DL; Foldager M; Jakobsen S; Nielsen DS	2022	1	1	1	1	1	1	1	0	0	1	NA	NA	NA	NA	NA	1	1	1	1	0	★★★★
CogChamps: impact of a project to educate nurses about delirium and improve the quality of care for hospitalized patients with cognitive impairment	Travers C; Henderson A; Graham F; Beattie E	2018	NA	NA	NA	NA	NA	1	1	1	0	1	NA	NA	NA	NA	NA	NA	NA	NA	NA	NA	★★★★
Care of patients with dementia in an acute trauma and orthopaedics unit	McCorkell G; Harkin D; McCrory V; Lafferty M; Coates V	2017	NA	NA	NA	NA	NA	1	1	1	0	0	NA	NA	NA	NA	NA	NA	NA	NA	NA	NA	★★★
Caring for people with dementia in hospital: findings from a survey to identify barriers and facilitators to implementing best practice dementia care	Tropea J; LoGiudice D; Liew D; Roberts C; Brand C	2017	NA	NA	NA	NA	NA	NA	NA	NA	NA	NA	1	0	0	0	1	NA	NA	NA	NA	NA	★★
The use of the truth and deception in dementia care amongst general hospital staff	Turner A; Eccles F; Keady J; Simpson J; Elvish R	2016	1	1	1	1	1	NA	NA	NA	NA	NA	NA	NA	NA	NA	NA	NA	NA	NA	NA	NA	★★★★★
Nurses' Perspectives and Experiences of Using a Bed-Exit Information System in an Acute Hospital Setting: Mixed Methods Study	Walzer S; Schön I; Pfeil J; Klemm S; Ziegler S; Schmoor C; Kunze C	2025	1	1	1	1	1	1	1	1	0	1	NA	NA	NA	NA	NA	1	1	1	1	0	★★★★
Junior doctor dementia champions in a district general hospital (innovative practice)	Wilkinson I; Coates A; Merrick S; Lee C	2016	1	1	1	1	1	NA	NA	NA	NA	NA	1	0	1	0	1	1	1	1	1	0	★★★★
Nurses' Experiences in Caring for Older Adults With Responsive Behaviors of Dementia in Acute Care	Yous ML; Ploeg J; Kaasalainen S; Schindel Martin L	2019	1	1	1	1	1	NA	NA	NA	NA	NA	NA	NA	NA	NA	NA	NA	NA	NA	NA	NA	★★★★★
Assessing the Attitudes of Greek Nurses Toward Computerized Dementia Screening	Stelios Zygouris; Mara Gkioka; Despina Moraitou; Birgit Teichmann; Thrasyvoulos Tsiatsos; Sotirios Papagiannopoulos; Magda Tsolaki	2020	NA	NA	NA	NA	NA	NA	NA	NA	NA	NA	1	0	1	0	1	NA	NA	NA	NA	NA	★★★

Note. MMAT criteria 2.1-2.5 are absent because no included report was appraised as a quantitative randomised controlled trial. For single-design studies, the rating represents the proportion of "Yes" judgements across the five applicable design-specific criteria. For mixed-methods studies, "Yes" judgements were summed across the 15 applicable qualitative, quantitative and mixed-methods criteria. The proportion was normalised to a five-point scale and rounded to the nearest whole star. This author-defined rating was used only as a compact descriptive summary and not to exclude, weight or rank studies.

Summary of MMAT criteria

Criterion	Studies meeting criterion (%)	Studies not meeting criterion (%)
1.1	100.0	0.0
1.2	98.3	1.7
1.3	98.3	1.7
1.4	98.3	1.7
1.5	100.0	0.0
3.1	71.4	28.6
3.2	100.0	0.0
3.3	71.4	28.6
3.4	0.0	100.0
3.5	57.1	42.9
4.1	87.5	12.5
4.2	6.3	93.8
4.3	87.5	12.5
4.4	12.5	87.5
4.5	100.0	0.0
5.1	100.0	0.0
5.2	93.8	6.3
5.3	93.8	6.3
5.4	75.0	25.0
5.5	6.3	93.8
