# Supplementary material for: Advancing dementia-friendly hospital care: a systematic review of healthcare professionals’ reported barriers and facilitators using the theoretical domains framework
Source: Age Ageing. 2026 Sep 22;55(9):afag285. doi: 10.1093/ageing/afag285 (PMC13596736; doi:10.1093/ageing/afag285)
Supplement: Supplementary file 1 [file supplementary_material_afag285.zip › aa-26-1308-File002.pdf]

Advancing Dementia-Friendly Hospital Care: A Systematic Review of Healthcare Professionals' Reported Barriers and Facilitators Using the Theoretical Domains Framework

Appendix 1

Search strategies, extraction form, coding rules, and supplementary results

Table A1.1: PubMed Search Strategy and Limits

Theme	Search Terms
**1. Dementia Concept**	("Dementia"[Mesh] OR "Dementia*"[tiab] OR "Cognitive Impairment*"[tiab] OR Alzheimer*[tiab] OR "Lewy body disease*"[tiab])<br>AND
**2. Setting (Hospital and Health Care Contexts)**	("Hospitals"[Mesh] OR "Hospital*"[tiab] OR "Inpatient*"[tiab] OR "Acute Care"[tiab] OR "Emergency Department*"[tiab] OR "Outpatient*"[tiab] OR "Geriatric Unit*"[tiab] OR "Healthcare Setting*"[tiab] OR "Health care setting*"[tiab] OR "Clinical Environment*"[tiab])<br>AND
**3. Health Professionals and Care Practice**	("Healthcare Provider*"[tiab] OR "Health Personnel"[tiab] OR "Health Professional*"[tiab] OR "Clinician*"[tiab] OR "Nurs*"[tiab] OR "Doctor*"[tiab] OR "Staff"[tiab] OR "Team*"[tiab] OR "Health Personnel"[Mesh] OR "Health Services for the Aged"[Mesh] OR "geriatric care" [tiab] OR "dementia care" [tiab] OR "Nursing"[Mesh] OR Physician*[tiab])<br>AND
**4. Barriers and Facilitators**	("Barrier*"[tiab] OR "Facilitator*"[tiab] OR "Enabler*"[tiab] OR "Obstacle*"[tiab] OR "Challeng*"[tiab] OR "Hindrance*"[tiab] OR "Constraint*"[tiab] OR "Limitation*"[tiab] OR "Impediment*"[tiab] OR "Inhibitor*"[tiab] OR "Supporting Factor*"[tiab] OR "Driving Factor*"[tiab] OR "Motivator*"[tiab] OR "Success Factor*"[tiab] OR "Opportunit*"[tiab] OR "Implement*"[tiab] OR "Adopt*"[tiab] OR "Train*"[tiab] OR "Attitude*"[tiab] OR "Confiden*"[tiab] OR "Competence*"[tiab] OR "Health Knowledge, Attitudes, Practice"[Mesh] OR "Experien*"[tiab] OR "Perception*"[tiab] OR "Belie*"[tiab] OR "Educat*"[tiab])
**Limits**	AND "2015/01/01"[PDat] : "2025/07/04"[PDat]

Table A1.2: EMBASE Search Strategy and Limits

Theme	Search Terms
**1. Dementia Concept**	('dementia'/exp OR 'dementia*':ti,ab,kw OR 'cognitive impairment*':ti,ab,kw OR 'alzheimer*':ti,ab,kw OR 'lewy body disease*':ti,ab,kw)<br><br>AND
**2. Setting (Hospital and Health Care Contexts)**	('hospital'/exp OR 'hospital*':ti,ab,kw OR 'inpatient*':ti,ab,kw OR 'acute care':ti,ab,kw OR 'emergency department*':ti,ab,kw OR 'outpatient*':ti,ab,kw OR 'geriatric unit*':ti,ab,kw OR 'healthcare setting*':ti,ab,kw OR 'health care setting*':ti,ab,kw OR 'clinical environment*':ti,ab,kw)<br><br>AND
**3. Health Professionals and Care Practice**	('healthcare provider*':ti,ab,kw OR 'health personnel':ti,ab,kw OR 'health professional*':ti,ab,kw OR 'clinician*':ti,ab,kw OR 'nurs*':ti,ab,kw OR 'doctor*':ti,ab,kw OR 'staff':ti,ab,kw OR 'team*':ti,ab,kw OR 'health care personnel'/exp OR 'elderly care'/exp OR 'geriatric care':ti,ab,kw OR 'dementia care':ti,ab,kw OR 'nursing'/exp OR 'physician*':ti,ab,kw)<br><br>AND
**4. Barriers and Facilitators**	('barrier*':ti,ab,kw OR 'facilitator*':ti,ab,kw OR 'enabler*':ti,ab,kw OR 'obstacle*':ti,ab,kw OR 'challeng*':ti,ab,kw OR 'hindrance*':ti,ab,kw OR 'constraint*':ti,ab,kw OR 'limitation*':ti,ab,kw OR 'impediment*':ti,ab,kw OR 'inhibitor*':ti,ab,kw OR 'supporting factor*':ti,ab,kw OR 'driving factor*':ti,ab,kw OR 'motivator*':ti,ab,kw OR 'success factor*':ti,ab,kw OR 'opportunit*':ti,ab,kw OR 'implement*':ti,ab,kw OR 'adopt*':ti,ab,kw OR 'train*':ti,ab,kw OR 'attitude*':ti,ab,kw OR 'confiden*':ti,ab,kw OR 'competence*':ti,ab,kw OR 'attitude to health'/exp OR 'experien*':ti,ab,kw OR 'perception*':ti,ab,kw OR 'belie*':ti,ab,kw OR 'educat*':ti,ab,kw)
**Limits**	AND [english]/lim<br>AND [humans]/lim<br>AND [2015-2025]/py<br>NOT ('editorial'/it OR 'letter'/it OR 'note'/it OR 'conference abstract'/it OR 'conference review'/it OR 'conference paper'/it)

Table A1.3: PsycINFO Search Strategy and Limits

Theme	Search Terms
**1. Dementia Concept**	((MH "Dementia+") OR TI Dementia* OR AB Dementia* OR TI "Cognitive Impairment*" OR AB "Cognitive Impairment*" OR TI Alzheimer* OR AB Alzheimer* OR TI "Lewy body disease*" OR AB "Lewy body disease*")<br><br>AND
**2. Setting (Hospital and Health Care Contexts)**	((MH "Hospitals+") OR TI Hospital* OR AB Hospital* OR TI Inpatient* OR AB Inpatient* OR TI "Acute Care" OR AB "Acute Care" OR TI "Emergency Department*" OR AB "Emergency Department*" OR TI Outpatient* OR AB Outpatient* OR TI "Geriatric Unit*" OR AB "Geriatric Unit*" OR TI "Healthcare Setting*" OR AB "Healthcare Setting*" OR TI "Health Care Setting*" OR AB "Health Care Setting*" OR TI "Clinical Environment*" OR AB "Clinical Environment*")<br><br>AND
**3. Health Professionals and Care Practice**	((MH "Health Personnel+") OR TI "Healthcare Provider*" OR AB "Healthcare Provider*" OR TI "Health Personnel" OR AB "Health Personnel" OR TI "Health Professional*" OR AB "Health Professional*" OR TI Clinician* OR AB Clinician* OR TI Nurs* OR AB Nurs* OR TI Doctor* OR AB Doctor* OR TI Staff OR AB Staff OR TI Team* OR AB Team* OR TI "Geriatric Care" OR AB "Geriatric Care" OR TI "Dementia Care" OR AB "Dementia Care" OR (MH "Nursing+") OR TI Physician* OR AB Physician*)<br><br>AND
**4. Barriers and Facilitators**	(TI Barrier* OR AB Barrier* OR TI Facilitator* OR AB Facilitator* OR TI Enabler* OR AB Enabler* OR TI Obstacle* OR AB Obstacle* OR TI Challeng* OR AB Challeng* OR TI Hindrance* OR AB Hindrance* OR TI Constraint* OR AB Constraint* OR TI Limitation* OR AB Limitation* OR TI Impediment* OR AB Impediment* OR TI Inhibitor* OR AB Inhibitor* OR TI "Supporting Factor*" OR AB "Supporting Factor*" OR TI "Driving Factor*" OR AB "Driving Factor*" OR TI Motivator* OR AB Motivator* OR TI "Success Factor*" OR AB "Success Factor*" OR TI Opportunit* OR AB Opportunit* OR TI Implement* OR AB Implement* OR TI Adopt* OR AB Adopt* OR TI Train* OR AB Train* OR TI Attitude* OR AB Attitude* OR TI Confiden* OR AB Confiden* OR TI Competence* OR AB Competence* OR (MH "Attitudes+") OR TI Experien* OR AB Experien* OR TI Perception* OR AB Perception* OR TI Belie* OR AB Belie* OR TI Educat* OR AB Educat*)
**Limits**	English language; journal articles; 1 January 2015 to 4 July 2025

Table A1.4: CINAHL Search Strategy and Limits

Theme	Search Terms
**1. Dementia Concept**	((MH "Dementia+") OR TI Dementia* OR AB Dementia* OR TI "Cognitive Impairment*" OR AB "Cognitive Impairment*" OR TI Alzheimer* OR AB Alzheimer* OR TI "Lewy body disease*" OR AB "Lewy body disease*")<br>AND
**2. Setting (Hospital and Health Care Contexts)**	((MH "Hospitals+") OR TI Hospital* OR AB Hospital* OR TI Inpatient* OR AB Inpatient* OR TI "Acute Care" OR AB "Acute Care" OR TI "Emergency Department*" OR AB "Emergency Department*" OR TI Outpatient* OR AB Outpatient* OR TI "Geriatric Unit*" OR AB "Geriatric Unit*" OR TI "Healthcare Setting*" OR AB "Healthcare Setting*" OR TI "Health Care Setting*" OR AB "Health Care Setting*" OR TI "Clinical Environment*" OR AB "Clinical Environment*")<br>AND
**3. Health Professionals and Care Practice**	((MH "Health Personnel+") OR (MH "Health Services for the Aged+") OR TI "Healthcare Provider*" OR AB "Healthcare Provider*" OR TI "Health Personnel" OR AB "Health Personnel" OR TI "Health Professional*" OR AB "Health Professional*" OR TI Clinician* OR AB Clinician* OR TI Nurs* OR AB Nurs* OR TI Doctor* OR AB Doctor* OR TI Staff OR AB Staff OR TI Team* OR AB Team* OR TI "Geriatric Care" OR AB "Geriatric Care" OR TI "Dementia Care" OR AB "Dementia Care" OR (MH "Nursing+") OR TI Physician* OR AB Physician*)<br>AND
**4. Barriers and Facilitators**	((MH "Attitude+") OR (MH "Health Knowledge, Attitudes, Practice+") OR TI Barrier* OR AB Barrier* OR TI Facilitator* OR AB Facilitator* OR TI Enabler* OR AB Enabler* OR TI Obstacle* OR AB Obstacle* OR TI Challeng* OR AB Challeng* OR TI Hindrance* OR AB Hindrance* OR TI Constraint* OR AB Constraint* OR TI Limitation* OR AB Limitation* OR TI Impediment* OR AB Impediment* OR TI Inhibitor* OR AB Inhibitor* OR TI "Supporting Factor*" OR AB "Supporting Factor*" OR TI "Driving Factor*" OR AB "Driving Factor*" OR TI Motivator* OR AB Motivator* OR TI "Success Factor*" OR AB "Success Factor*" OR TI Opportunit* OR AB Opportunit* OR TI Implement* OR AB Implement* OR TI Adopt* OR AB Adopt* OR TI Train* OR AB Train* OR TI Attitude* OR AB Attitude* OR TI Confiden* OR AB Confiden* OR TI Competence* OR AB Competence* OR TI Experien* OR AB Experien* OR TI Perception* OR AB Perception* OR TI Belie* OR AB Belie* OR TI Educat* OR AB Educat*)
**Limits**	English language; human studies; publication type and date range were applied in the interface (1 January 2015 to 4 July 2025)

Table A1.5: Data Extraction Form Template:

Section	Domains	Description

Study identification and setting	Title	Full study title.
	Author(s)	Authors as cited.
	Year	Publication year.
	Hospital Type (acute, teaching, geriatric, psychiatric, etc.)	Hospital classification.
	Country	Country/region.
	Setting details (e.g., outpatient, acute geriatric ward, ED, mixed wards)	Clinical environment/ward context.
	Sample size	Total N (and by group if available).
Study design and methods	Study Design	Qualitative, descriptive, cohort, mixed methods, etc.
	Participants (Professional groups)	Staff groups included (nurses, physicians, HCAs, managers, etc.).
	Theoretical Framework used (If any)	Framework(s) used (e.g., TDF, CFIR, COM-B, NPT).
	Data Collection Methods	Interviews, focus groups, observation, surveys, audit, routine data, etc.
	Study aim and Key Findings	Concise narrative of main DFHC messages (avoid long lists).
	Notes	Use sparingly for unusual/supplementary context.
Intervention and implementation specification	DFHC Intervention (TIDieR elements)	What the DFHC intervention is + how it was delivered (core components, mode, dose, who delivered, materials, tailoring/fidelity if reported).
	Implementation Strategy (Proctor et al.)	How it was implemented/embedded (strategy label, actor, actions, temporality, dose, resources).
	AACTT<br>– Action<br>– Actor<br>– Context<br>– Target<br>– Time	What behaviour is promoted?<br>Who performs the behaviour?<br>Where does it occur (ward/hospital/country)?<br>To whom/what is it directed (patients/teams/processes)?<br>When/how often/for how long?
	Outcomes related to implementation	Implementation outcomes (acceptability, adoption, feasibility, fidelity, reach, sustainability, costs), as reported.
Determinants and TDF mapping	Verbatim Barrier Phrases: Behavioural Determinant Description	Granular barriers (verbatim or close paraphrase).

	Verbatim Facilitator Phrasing: Behavioural Determinant Description	Granular facilitators (verbatim or close paraphrase).
	TDF Domains – Barriers	TDF coding for barriers.
	TDF Domains – Facilitators	TDF coding for facilitators.
	Barriers specific to profession	Profession-specific barriers.
	Facilitators specific to profession	Profession-specific facilitators.
	Barriers per profession → TDF	Profession-specific barrier → TDF mapping.
	Facilitators per profession → TDF	Profession-specific facilitator → TDF mapping.
	Extractor notes	Internal notes on coding decisions/uncertainties.
Quality appraisal (MMAT 2018)	1.1_QualApproach	Qualitative: approach appropriate for the question?
	1.2_QualDataCollection	Qualitative: data collection adequate?
	1.3_QualFindings	Qualitative: findings derived from data?
	1.4_QualInterpretation	Qualitative: interpretation supported by data?
	1.5_QualCoherence	Qualitative: coherence across data/analysis/interpretation?
	3.1_NonRandRepresentative	Non-randomized: participants representative?
	3.2_NonRandMeasurements	Non-randomized: measurements appropriate?
	3.3_NonRandCompleteData	Non-randomized: complete outcome data?
	3.4_NonRandConfounders	Non-randomized: confounders accounted for?
	3.5_NonRandIntervention	Non-randomized: intervention/exposure as intended?
	4.1_DescriptiveSampling	Descriptive: sampling strategy relevant?
	4.2_DescriptiveRepresentativeness	Descriptive: sample representative?
	4.3_DescriptiveMeasurements	Descriptive: measurements appropriate?
	4.4_DescriptiveNonresponseBias	Descriptive: nonresponse bias low/handled?
	4.5_DescriptiveStatistics	Descriptive: analysis appropriate?
	5.1_MM_Rationale	Mixed methods: rationale for MM design?
	5.2_MM_Integration	Mixed methods: components integrated?

	5.3_MM_Interpretation	Mixed methods: integration interpreted?
	5.4_MM_Divergences	Mixed methods: divergences addressed?
	5.5_MM_QualityAdherence	Mixed methods: each component meets quality criteria?
	MMAT descriptive rating (0–5 stars)	Summary of the proportion of applicable criteria rated 'Yes', normalised to five and rounded to the nearest whole star.

***Note**: MMAT criteria 2.1–2.5 are absent because no included report was appraised as a quantitative randomised controlled trial. For single-design studies, the rating represents the proportion of "Yes" judgements across the five applicable design-specific criteria. For mixed-methods studies, "Yes" judgements were summed across the 15 applicable qualitative, quantitative and mixed-methods criteria. The proportion was normalised to a five-point scale and rounded to the nearest whole star. This author-defined rating was used only as a compact descriptive summary and not to exclude, weight or rank studies.*

Table A1.6. Operational definitions and decision rules for classifying implementation determinants

Item	Operational definition / decision rule
**Implementation determinant**	Any factor described by healthcare professionals as influencing whether DFHC practices are delivered in daily hospital care.
**Barrier**	A determinant described as hindering DFHC delivery or reducing its feasibility, consistency, or quality in routine care (e.g., insufficient time; noisy environment; unclear responsibility).
**Facilitator**	A determinant described as enabling DFHC delivery or increasing its feasibility, consistency, or quality (e.g., leadership support; dementia champion; quiet space).
**Qualitative studies: identifying barriers/facilitators**	Barriers/facilitators were identified from participant quotations and/or authors' interpretations, based on the direction of influence expressed in context.
**Quantitative studies: identifying barriers/facilitators**	Barriers/facilitators were identified from the wording and direction of items and results (e.g., agreement with negatively framed items = barrier; agreement with positively framed items = facilitator). Open-ended survey responses were treated as qualitative text.

Note: Decision rules were applied consistently across studies; uncertainties were resolved by discussion between reviewers.

Table A1.7. Frequency of TDF-coded barriers and facilitators by professional group (nurses, doctors, allied health professionals, and managers).

TDF Count	**Nurse**		**Doctors**		**AHPs**		**Managers**	
	Barriers	Facilitators	Barriers	Facilitators	Barriers	Facilitators	Barriers	Facilitators
Knowledge	33	36	10	8	14	16	2	2
Skills	27	37	5	5	7	13	0	1
Social/Professional Role and Identity	11	11	9	9	10	5	5	5
Beliefs about Capabilities	6	8	0	2	1	1	0	0
Optimism	0	3	0	1	0	1	0	0
Beliefs about Consequences	23	14	13	5	2	3	3	3
Reinforcement	0	7	0	1	0	0	0	3
Intentions	2	0	2	0	0	0	1	0
Goals	34	28	8	6	6	3	17	18
Memory, Attention, and Decision Processes	18	4	7	1	9	4	0	0
Environmental Context and Resources	50	38	12	5	21	18	25	18
Social Influences	23	37	6	9	8	21	17	22
Emotion	20	3	2	0	1	0	1	0
Behavioural Regulation	32	38	4	6	5	7	8	9
Total	**279**	**264**	**78**	**58**	**84**	**92**	**79**	**81**

Abbreviations: TDF *= Theoretical Domains Framework;* ***AHPs*** *= allied health professionals.*

Table A1.8. Cross-cutting themes of linked determinants in barriers and facilitators to implementing dementia-friendly hospital care, mapped to the Theoretical Domains Framework (TDF)

Type	Cross-cutting theme bundles	Brief description (linked bundles)	Typical contexts / professions	Dominant TDF domains
Barrier	**System pressures and feasibility constraints**	Workload, time and staffing pressures, competing priorities, dementia-unfriendly environments, and fragmented documentation/information systems commonly co-occurred, reducing the practical doability of DFHC in routine care. These constraints often limited continuity, handover, and access to person-specific information.	High-pressure acute wards, ED, peri-operative, orthopaedics, oncology/outpatients, mixed wards; Nurses, Doctors, AHPs, Managers	Environmental Context and Resources; Goals; Behavioural Regulation; Memory, Attention and Decision Processes
Barrier	**Capability gaps in dementia-specific knowledge, skills, and confidence**	Limited dementia, BPSD, delirium, pain, and communication knowledge, alongside variable assessment, de-escalation, and tailoring skills, often co-occurred with low confidence and uncertainty. Under pressure, this was frequently linked to reactive, restrictive, or pharmacological responses.	ED, peri-operative, general wards, pain care, imaging/diagnostics, orthopaedics; all groups, often most visible in Nurses and AHPs	Knowledge; Skills; Beliefs about Capabilities; Beliefs about Consequences
Barrier	**Task-first cultures, hierarchy, and unclear ownership**	Acute-illness-first norms, role ambiguity, hierarchy, weak interprofessional working, and low or uneven buy-in commonly interacted with stigma, emotional burden, and control-oriented responses. Relational and supportive dementia care was often seen as extra work, peripheral work, or someone else's responsibility.	Multidisciplinary wards, discharge pathways, older people's wards, oncology, stroke, mixed acute settings; all groups	Social Influences; Social/Professional Role and Identity; Emotion; Beliefs about Consequences; Goals
Facilitator	**Visible leadership and champions**	Executive sponsorship, ward leaders, dementia leads/champions, and implementation teams created legitimacy, prioritization, accountability, and follow-through, helping DFHC	Ward-level and hospital-wide programs; all groups, with Managers/champions prominent	Social Influences; Goals; Environmental Context and Resources

		remain visible amid competing pressures.		
Facilitator	**Experiential, role-relevant, and ongoing education**	Interactive, ongoing, role-tailored education, especially when paired with modelling, coaching, practice-based examples, or feedback, built capability and confidence and helped staff translate dementia principles into everyday practice.	Whole-workforce and specialty training; all groups	Knowledge; Skills; Beliefs about Capabilities; Reinforcement; Emotion
Facilitator	**Interprofessional teamwork and shared ownership**	MDT huddles, behavioural rounds, shared planning, handover, and collaborative routines improved consistency, reduced duplication, clarified roles, and supported staff when managing complexity under pressure.	ACE/dementia units, rehabilitation, acute wards, discharge and transition pathways; all groups	Social Influences; Social/Professional Role and Identity; Behavioural Regulation
Facilitator	**Workflow fit for person-centred practice**	Person-centred information, family input, continuity cues, and feasible workflow/environmental adaptations, often supported by prompts, identifiers, tools, or technology, helped staff tailor care in real time and reduced friction across shifts and settings.	Admission and ward routines, ED, outpatient care, imaging, transitions; Nurses, Doctors, AHPs, Managers	Environmental Context and Resources; Behavioural Regulation; Memory, Attention and Decision Processes; Social Influences

Note: Bundles were developed inductively by grouping determinants described as related within individual studies. A bundle was retained if it spanned ≥2 TDF domains, recurred in ≥3 studies, and was identified across ≥2 professional groups and/or ≥2 hospital settings. Bundles are not mutually exclusive.

Abbreviations: *AHP = allied health professionals; ED = emergency department; TDF = Theoretical Domains Framework.*

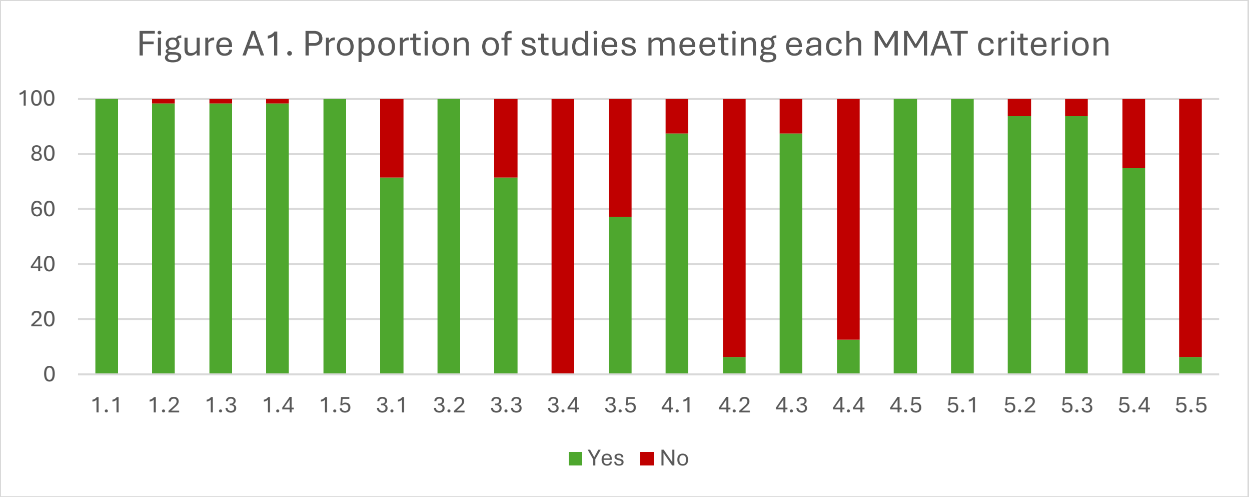

Figure A1. Proportion of studies meeting each MMAT criterion. Percentages are calculated among studies for which each criterion was applicable (NA excluded).

Note: X-axis codes are MMAT criteria by design: 1 = qualitative; 2 = quantitative randomised; 3 = quantitative nonrandomized; 4 = quantitative descriptive; 5 = mixed-methods. MMAT criteria 2.1–2.5 are absent because no included report was appraised as a quantitative randomised controlled trial.
